# Single-Cell Multi-Omics Analysis of *In Vitro* Post-Ovulatory–Aged Oocytes Revealed Aging-Dependent Protein Degradation

**DOI:** 10.1016/j.mcpro.2024.100882

**Published:** 2024-11-20

**Authors:** Yueshuai Guo, Mengmeng Gao, Xiaofei Liu, Haotian Zhang, Yue Wang, Tong Yan, Bing Wang, Xudong Han, Yaling Qi, Hui Zhu, Chenghao Situ, Yan Li, Xuejiang Guo

**Affiliations:** 1 State Key Laboratory of Reproductive Medicine and Offspring Health, Department of Histology and Embryology, Nanjing Medical University, Nanjing, China; 2 School of Medicine, Southeast University, Nanjing, China; 3 Department of Clinical Laboratory, Sir Run Run Hospital, Nanjing Medical University, Nanjing, China

**Keywords:** single cell, multi-omics, post-ovulation aging, oocyte, mouse, melatonin

## Abstract

Once ovulated, the oocyte has to be fertilized in a short time window or it will undergo post-ovulation aging (POA), whose underlying mechanisms are still not elucidated. Here, we optimized single-cell proteomics methods and performed single-cell transcriptomic, proteomic, and phosphoproteomic analysis of fresh, POA, and melatonin-treated POA oocytes. POA oocytes showed downregulation of most differentially expressed proteins, with little correlation with mRNA expression, and the protein changes can be rescued by melatonin treatment. MG132 treatment rescued the decreased fertilization and polyspermy rates and upregulated fragmentation and parthenogenesis rates of POA oocytes. MG132-treated oocytes displayed health status at proteome, phosphoproteome, and fertilization ability similar to fresh oocytes, suggesting that protein stabilization might be the underlying mechanism for melatonin to rescue POA. The important roles of proteasome-mediated protein degradation during oocyte POA revealed by single-cell multi-omics analyses offer new perspectives for increasing oocyte quality during POA and improving assisted reproduction technologies.

## Highlights

• Single-cell multi-omics analysis of fresh, POA and melatonin-treated mouse oocytes.
• Melatonin improved the quality of POA oocytes at protein but not mRNA level.
• Proteasome-mediated protein degradation is important during oocyte POA.
• MG132 improved fertilization ability and reduced polyspermy rate of POA oocytes.

## Graphical Abstract

**Figure:**
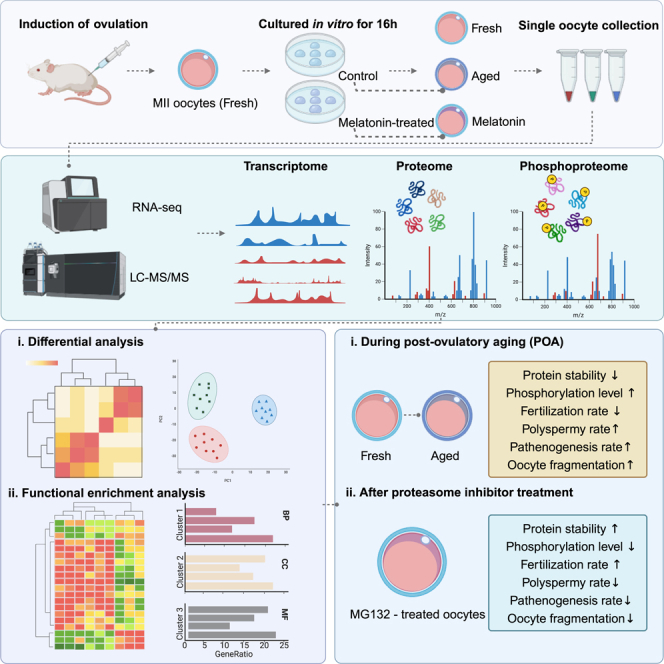

## In Brief

Here, we performed single-cell transcriptomic, proteomic, and phosphoproteomic analysis of fresh, post-ovulation aging (POA), and melatonin-treated POA oocytes and found that most differentially expressed proteins were downregulated in POA oocytes which could be rescued by melatonin treatment. Oocytes treated with MG132 exhibited comparable health conditions at fertilization capacity, polyspermy rate, proteome, and phosphoproteome to fresh oocytes, hinting protein stabilization might be a potential mechanism for melatonin to rescue POA.

In mammals, the development of a new organism initiates from the fertilization of oocytes by sperm (1). Once ovulated, the oocyte has to be fertilized in a short time window or it will undergo a time-dependent degeneration known as post-ovulatory aging (POA), which refers to the decline in oocyte quality that occurs with increasing time after ovulation (2). For example, human oocytes degenerate about 24 h after ovulation, while mouse oocytes degenerate 8 to 12 h after ovulation (3). The health and quality of oocytes significantly impact the success of fertilization and the subsequent embryo development (4, 5). Assisted reproduction technology (ART) has been widely used to treat infertile couples worldwide (6). And ART suffers from POA due to the ineluctable long-time culture of oocytes before *in vitro* fertilization (IVF) (7), especially when rescue intracytoplasmic sperm injection was performed due to fertilization failure during IVF (8, 9). During ART, POA can impair the fertilization ability of oocytes *in vitro* (10), lower the quality of the embryo (11), increase the risk of early miscarriage (12, 13), and raise the likelihood of abnormal offspring (14, 15, 16). Alleviation of the effects of POA is expected to help improve the success rate of ART and fertilization *in vivo*.

During POA, oocytes display various aberrations, such as mitochondrial dysfunction, accumulated reactive oxygen species, and downregulation of cell cycle factors including maturation-promoting factor and MAPK (17, 18, 19). Mitochondria produce ATPs and are important for oocyte fertilization (20, 21). Reactive oxygen species accumulation increases oxidative stress and affects the quality of metaphase II (MII) oocytes (22, 23). Maturation-promoting factor and MAPK maintain cell cycle arrest once oocytes reach the MII phase by phosphorylating downstream substrates, and their abnormal expression in POA oocytes indicates the involvement of protein phosphorylation in POA (24). Previous studies have shown that melatonin, which is synthesized and produced by the pineal gland (25, 26), can maintain oocyte quality after ovulation (27). It ameliorates oocyte quality decrease during POA by postponing apoptosis, resulting in an extended ideal window for fertilization, increased fertilization rates, and improved embryo quality (18, 27, 28). Nevertheless, the underlying molecular mechanisms of oocyte quality decrease during POA and quality maintenance by melatonin have not been elucidated.

The studies of oocytes are limited by their low availability of samples. In each ovulation, only a few dozen oocytes can be generated. The development of single-cell omics technologies has opened new avenues for understanding oocytes. And sequencing-based omics methods have been used to characterize oocyte development. However, these studies were mainly conducted at the transcriptional level, which could not accurately reflect intricate posttranscriptional and posttranslational regulations in oocytes (29). Single-cell mass spectrometry–based proteomics methods enabled us to characterize oocytes at a single-cell level. Recently, Jiang *et al.* developed a single-cell simultaneous transcriptome and proteome technology and quantified 2663 protein groups in single mouse oocytes (30). He *et al.* had developed an on-capillary alkylation microreactor for proteo-metabolome profiling in the same single cells and identified 3457 protein groups and 171 metabolites in single mouse oocytes (31). Our previous single-cell data-dependent acquisition (DDA)–based proteomic profiling of human oocyte maturation *in vitro* and *in vivo* revealed little correlation of protein changes between mRNA and protein levels (32). Compared with DDA, BoxCar (33) and data-independent acquisition (DIA) (34, 35) are also widely used. They have been used to improve the identification depth and quantification accuracy of proteins in the analysis of bulk samples and may have improved performance in single oocyte proteome analysis. Besides protein expression, protein posttranslational modifications, such as phosphorylation, also regulate oocyte functions (36). However, single-cell characterization of phosphoproteome remains challenging due to low abundance of phosphorylation. To characterize the mechanisms of POA, it is important to systemically characterize post-ovulatory oocyte aging and its reversal at mRNA, protein, and phosphorylation levels.

In this study, we optimized single-cell proteomics methods for single oocyte and performed multi-omics profiling of fresh, POA, and melatonin-treated oocytes and found that melatonin rescued the quality defects of oocytes caused by POA mainly at the protein level but not the transcriptional level. The maintenance of protein stability during POA could ameliorate the quality decrease of aged oocytes.

## Experimental Procedures

### Experimental Design and Statistical Rationale

To investigate the quality differences of fresh oocytes, POA oocytes, and melatonin-treated POA oocytes, three groups of mouse oocytes (fresh; aged: defined as POA oocytes, that oocytes aged for 16 h after ovulation *in vitro*; melatonin: melatonin-treated oocytes aged for 16 h after ovulation *in vitro*) were used. Nine biological replicates of each group were analyzed using our single-cell proteomic method (a total of 27 samples used).

The protein or phosphopeptide quantification data were filtered according to the following criteria: (i) proteins or phosphopeptides identified in no less than 50% of the samples were preserved for subsequent analysis; for phosphopeptides, only phosphopeptides with single phosphorylation site were preserved for subsequent analysis to better show the phosphorylation site changes. To avoid the potential effect of protein expression changes, the level of each phosphopeptide was further calibrated by dividing the level of the corresponding protein. If the corresponding proteins could not be quantified by proteomic analysis, the level of the phosphopeptides were used directly for statistical analysis (37); (ii) samples that identified less than 80% of the total proteins were considered of low quality and discarded. Expression levels of each protein were log2 transformed in the following analysis to avoid invalid values. To compensate for the missing quantitative value caused by low protein abundance, we imputed the data with quantile regression imputation of left-censored data algorithm (38). The statistical significance of the differences of RNA, protein expression, and phosphorylation levels among groups were determined using one-way ANOVA test, and false discovery rate (FDR)-corrected *p* value (FDR-*q*) cut-off of 0.05 was used based on the Benjamini–Hochberg method. Post-hoc Tukey test was performed for differences between groups. For other comparisons, independent Student’s *t* test or one-way ANOVA test followed by the post-hoc Tukey test was used for two or three groups, respectively. All experiments were repeated for three or more times, and data were given as mean ± SEM. *p* < 0.05 was considered significant.

### Animals

All animal experiments were approved by the Institutional Animal Care and Use Committee of Nanjing Medical University (No: IACUC-1810007). ICR female mice (6–8 weeks old) and ICR male mice (10–20 weeks old) used in the study were purchased and housed at the Animal Core Facility of Nanjing Medical University under specific pathogen-free environmental conditions with free access to water and food, a temperature range of 20 to 22 °C, a humidity range of 50 to 70%, and a 12-h light/dark cycle.

### Oocyte Collection

Female mice were superovulated by injecting 5 IU of pregnant mare’s serum gonadotropin (Ningbo Sansheng Pharmaceutical Corporation, 110914564), followed by 5 IU human chorionic gonadotropin (Ningbo Sansheng Pharmaceutical Corporation, 110911282) 48 h later. After 14 h, the manipulated mice were euthanized by cervical dislocation. Cumulus-oocyte complexes were isolated from oviduct ampullae and then cultured in 37 °C M2 medium (Sigma, M7167) containing 0.5 mg/ml hyaluronidase (Sigma, H3506) to remove the cumulus mass to obtain denuded MII oocytes.

### Oocyte Culture

To determine the effect of melatonin or MG132 on POA oocytes, melatonin and MG132 were prepared into 1 M and 5 mM stock solutions using dimethyl sulfoxide (DMSO) (MedChemExpress, HY-Y0320) and diluted into 1 mM and 5 μM work solutions using M2 culture medium at the time of use, respectively. And the final concentration of the DMSO solvent was 0.1% of the culture medium. Freshly collected MII oocytes were cultured for 16 h in a droplet of M2 medium containing 0.1% (v/v) DMSO, without (aged group, vehicle control) or with 1 mM melatonin (Sigma, M5250) (Melatonin group), 5 μM MG132 (MedChemExpress, HY-13259) (MG132 group) under mineral oil (Sigma, M8410) in accordance with previous studies (11, 39, 40), respectively. The freshly collected MII oocytes were directly used in the fresh group.

### *In vitro* Fertilization

IVF was assessed as previously described (41, 42). Sperm were released from dissected cauda epididymis and capacitated in human tubal fluid (HTF) (EasyCheck, M1130) for 1 h at 37 °C with 5% CO_2_. Subsequently, capacitated sperm were cocultured with denuded MII oocytes in HTF for 5 h at 37 °C to facilitate fertilization. After successful fertilization, zygotes were transferred into KSOM medium (EasyCheck, M1430) and cultured at 37 °C.

### Measurement of ATP Content

ATP content was measured using an Enhanced ATP Assay Kit (Beyotime Biotechnology, S0027). Briefly, 100 oocytes per group were pooled and processed according to the manufacturer’s protocol. A seven-point standard curve (0, 0.1, 0.2, 0.3, 0.4, 0.5, and 1.0 μmol of ATP) was generated in each assay, and ATP levels were calculated using the formula derived from the linear regression of the standard curve.

### Sperm Binding Assay

Mouse sperm were capacitated as described above and then added to different groups of oocytes in HTF for 1.5 h in the incubator (37 °C, 5% CO2). Samples were then fixed in 4% paraformaldehyde for 30 min and stained with Hoechst 33342. The number of fused sperm per oocyte was quantified by confocal microscopy (LSM 800, Zeiss).

### Immunostaining and Confocal Microscopic Imaging of Oocyte Staining

Immunofluorescence staining was performed as described previously (43). Oocytes were fixed in 4% paraformaldehyde (Sigma, P6148) for 30 min, followed by permeabilization with 0.5% Triton X-100 (Sigma, T8787) for 45 min at room temperature. Then, the oocytes were blocked in blocking buffer (2.5% bovine serum albumin (Sigma, A1933) and 0.2% Triton X-100) for 1 h at room temperature and incubated overnight at 4 °C with anti-α-tubulin-FITC antibody (Abcam, ab64503). After three washes, chromosomes were stained with propidium iodide (Sigma, P4864) for 10 min. The oocytes were mounted on glass slides and detected under a confocal laser scanning microscope (LSM 800, Zeiss).

### Single-Cell RNA-seq Library Construction

Single oocytes in different groups were chosen and aspirated using pipettes under a microscope. Using the Single Cell Full Length mRNA-Amplification Kit (Vazyme, N712) in accordance with the manual's instructions, each chosen oocyte was put to lysis buffer right away for single-cell RNA extraction, reverse transcription, and complementary DNA amplification. The TruePrep DNA Library Prep Kit V2 for Illumina (Vazyme, TD502) was used to create RNA-seq libraries from pure complementary DNA, which followed the manual's instructions. After that, a NovaSeq 6000 (Illumina) was used to sequence the libraries.

### Proteomic Sample Preparation

In this study, 1000 germinal vesicle oocytes, 1000 MII oocytes, and 1000 zygotes were collected for the construction of spectra library or DIA-based protein expression and phosphorylation level quantification; proteins in oocytes were extracted by protein extraction buffer (8 M urea, 75 mM NaCl, 50 mM Tris, pH 8.2, 1% (vol/vol) EDTA-free protease inhibitor, 1 mM NaF, 1 mM β-glycerophosphate, 1 mM sodium orthovanadate, 10 mM sodium pyrophosphate, 1 × cocktail), reduced, alkylated, trypsin digested, and desalted by SepPak column from Waters Co (Milford) as described previously (44, 45).

For single oocyte proteomic experiments, each oocyte was lysed in 0.1% N-Dodecyl-β-D-maltoside, 130 mM tetraethylammonium bromide, 10 mM Tris(2-carboxyethyl) phosphine, 25 mM cyanoacetamide, 10 ng/μl trypsin, for 5 h at 37 °C, after which the peptides were dried with a SpeedVac concentrator before MS analysis.

### High-pH Reverse Phase Fractionation

For construction of proteome spectral library, the peptides were fractionated at pH 10 by a high-pH reverse phase column using an XBridge BEH130 C18 column (BEH C18, 1.7 μm, 300 μm × 150 mm, Waters) using the ACQUITY UPLC M-Class system (Waters). Twelve fractions were collected using a nonadjacent pooling scheme with a 74 min gradient of 0 to 10% buffer B (100% ACN/20 mM ammonium formate, pH 10.0) for 14 min, 3 to 8% B for 1 min, 8 to 29% B for 24 min, 29 to 41% B for 4 min, and 41 to 100% B for 18 min, followed by 21 min at 3% B. Each peptide fraction was then dried using a Speedvac concentrator (Labconco).

### Phosphopeptide Enrichment

For construction of phosphoproteome spectral library, phosphopeptides were enriched by immobilized metal affinity chromatography (Ti^4+^-IMAC, JK Chemical) as previously described (46). Briefly, peptides were resuspended in a loading buffer (80% ACN, 6% TFA) and incubated for 20 min with IMAC beads at a ratio of 1:10 (w/w) at room temperature. The peptides were then loaded onto the equilibrated StageTips (Thermo Fisher Scientific, SP301) with the magnetic beads, and the phosphopeptides were eluted with elution buffer (10% NH4OH). Next, the supernatant was transferred to a clean microtube, dried by Speedvac concentrator, and stored at −20 °C until MS analysis.

### LC-MS/MS Analysis

The LC-MS/MS analysis was performed on an Orbitrap Fusion Lumos mass spectrometry (Thermo Fisher Scientific) coupled with the Easy-nLC 1200 (Thermo Fisher Scientific). The purified peptide samples in 0.1% FA were separated on an analytical column (75 μm × 150 mm, 1.7 μm, CoAnn Technologies) using a 95-min linear gradient (3% to 5% buffer B (80% acetonitrile, 0.1% FA) for 5 s, 5% to 15% buffer B for 40 min, 15% to 28% buffer B for 34 min 50 s, 28% to 38% buffer B for 12 min, 38% to 100% buffer B for 5 s, 100% buffer B for 8 min).

To construct proteome or phosphoproteome libraries for BoxCar and DIA, the peptides or enriched phosphopeptides were subjected to DDA acquisition analysis in an MS instrument with full MS scans from m/z 350–1500 at an MS1 resolution of 60 K with a standard automated gain control (AGC) target and a maximum injection time of 20 ms. For higher-energy collisional dissociation (HCD) MS/MS scans, the normalized collision energy was set to 30% with 15 K resolution, a standard AGC target, and a dynamic maximum injection time.

For comparison of single oocyte proteomic methods among DDA, DIA, and BoxCar, the DDA acquisition mode of MS instrument was set up as full MS scans from m/z 350–1500 at an MS1 resolution of 120 K with an AGC target of 250% and a maximum injection time of 50 ms. For HCD MS/MS scans, the normalized collision energy was set to 30% with 30 K resolution, an AGC target of 100%, and a dynamic maximum injection time.

The DIA acquisition mode was acquired using the parameters of scan range of 350-1500 m/z at an MS1 resolution of 120k with an AGC target of 250%, and the maximum injection time was 50 ms. The MS/MS scan was performed in HCD mode using a normalized collision energy of 30%, resolution at 30k, maximum injection time of dynamic, AGC target of 100% with a 50 Da isolation window over 350 to 450 m/z precursor, 15 Da isolation window over 450 to 960 m/z precursor, and 100 Da isolation window over 960–1500 m/z precursor according to the signal distribution of tryptic peptides in this experiment (Supplemental Table S1*A*).

For the BoxCar acquisition mode, we implemented the BoxCar quantitation method with some minor modifications (33, 47). The scan was set as 3 and the box was set as 10. The overlap of neighboring boxes was 1 Th (47). Isolation windows used are described in Supporting Information (Supplemental Table S1*B*). For the full scan, the resolution was 120k and AGC target was set as 250%. The scan range was set as 350-1500 m/z. The maximal ion injection time was 50 ms. The HCD mode had a normalized collision energy at 30%. For the MS/MS scan, fragment spectra were detected by Orbitrap analyzer with resolution of 120k (48). The AGC target was set as 100% for each injection step. The maximum injection time was set as dynamic.

### Single-Cell RNA-seq Data Analysis

Raw reads from Illumina sequencing were initially trimmed and filtered by Trim Galore, and then clean reads were mapped against GRCm39 using STAR (49), and uniquely mapped reads were counted using featureCounts (50). Gene expression levels were quantified with transcripts per million.

### Proteomic Data Analysis

The standard single cell DDA raw data were processed using MaxQuant software (1.6.5.0) with default settings (51), the single cell BoxCar raw data were processed using MaxQuant software (1.6.5.0) incorporating RAW data of the proteome spectral libraries as described in previous study (52), precursor mass ions tolerance was set to 20 ppm for the first search and 4.5 ppm for the main search, and fragment ions mass tolerance was set to 20 ppm (53). The reference FASTA files for mouse were downloaded from UniProtKB/SwissProt (version: 2024-10; 17,334 entries) (54). Carbamidomethyl (C) was set as fixed modifications. Oxidation (M), acetyl (Protein N-term), and phospho (STY) were set as variable modifications. Enzyme specificity was considered to be full cleavage by trypsin, and two maximum missed cleavage sites were permitted. The cut-off of FDR-*q* for identification of peptides, proteins, and sites was set to 0.01. The MBR feature with a match window of 0.7 min was used. Label-free quantitation was used to estimate protein abundance according to the previous procedure (55). All other parameters were the default settings of the Maxquant software.

Single-cell DIA data analysis together with proteome spectral library or phosphoproteome spectral library construction were performed using the DIA_DIA-Umpire_SpecLib_Quant (56) workflow in FragPipe computational platform (version 22.0) with MSFragger (57, 58) (version 4.1), IonQuant (version 1.10.27), Philosopher (59) (version 5.1.1), and EasyPQP (version 0.1.49) components. DIA_DIA-Umpire_SpecLib_Quant workflow takes proteome spectral library of DDA data files of 12 fractions for single-cell DIA-based protein expression analysis and phosphoproteome spectral library of DDA data file of one fraction for single-cell DIA-based phosphoproteome analysis. It generates pseudo-MS/MS spectra from the single-cell DIA files using DIA-Umpire (56), searched the DIA extracted pseudo-MS/MS spectra against the proteome spectral library or phosphoproteome spectral library using MSFragger, followed by quantification with DIA-NN (version 18.0) (60) for single-cell proteome or single-cell phosphoproteome analysis, respectively. For the DIA-Umpire analysis, precursor ions tolerance was set to 10 ppm and fragment mass tolerance was set to 20 ppm. For the MSFragger analysis, the precursor mass tolerance was set to 20 ppm and the fragment mass tolerance to 20 ppm. The reference FASTA files for mouse were UniProtKB/SwissProt (version: 2024-10; 17,334 entries) and combined with the standard contamination database built into the software. Reversed protein sequences were appended to the original databases as decoys. Carbamidomethyl (C) was set as fixed modification, oxidation (M) and protein N-term acetyl as variable modifications. At least one unique peptide was required for protein inference. Post-processing with PeptideProphet (61) and ProteinProphet (62) in Philosopher was performed with 1% FDR filtering at both the PSM and protein levels (sequential filtering). For phosphorylation site identification, phosphor (STY) was additionally set as a variable modification. The phosphorylation site localization probability cut-off was set as 0.75 for PTM Prophet (63). The remaining parameters are default parameters.

### Bioinformatics Analysis

Correlation analysis across samples was performed on stats package using the OmicStudio tools (64) at https://www.omicstudio.cn/tool. The Principal Component Analysis (PCA) was performed by R software using the stats package. Heatmaps were derived using the ComplexHeatmap (65) package, and Z-score was used for standardization between samples. ID conversion and annotation were conducted using clusterProfiler (66) and org.Mm.eg.db (67) packages. Gene ontology (GO) enrichment analysis of differentially expressed proteins was also performed using clusterProfiler package (66).

## Results

### DIA Performs Better than DDA and BoxCar for Single-Cell Proteomic Analysis of Oocytes

**Fig. 1:**
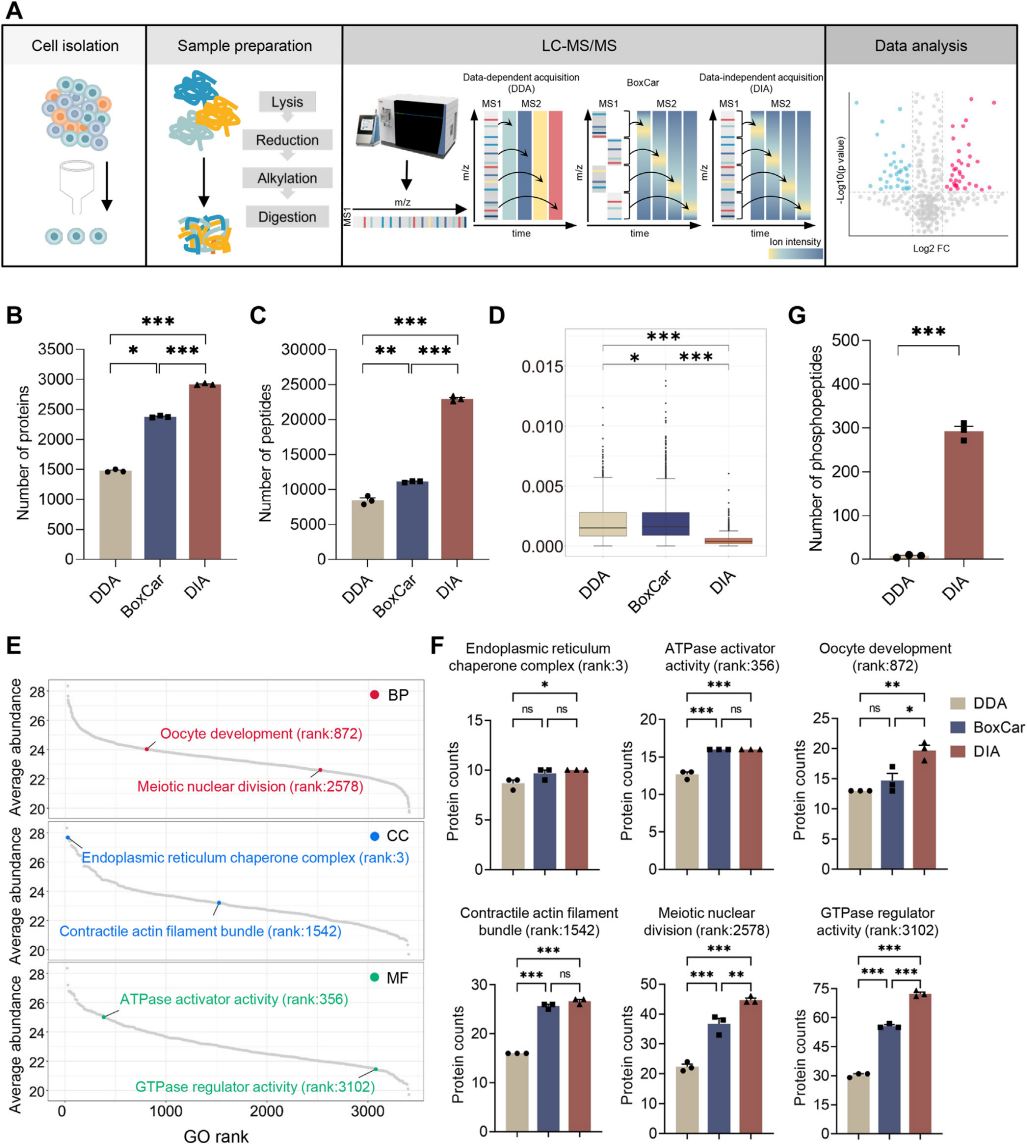
**Performance comparison of DDA, BoxCar, and DIA methods in single-cell proteomics.***A*, schematic overview of the workflow of single-cell proteomics analysis using DDA, BoxCar, or DIA methods. *B* and *C*, the number of proteins (*B*) and peptides (*C*) quantified (n = 3) using DDA, BoxCar, and DIA. *D*, coefficient of variation of log2-transformed quantification values of proteins commonly identified by DDA, BoxCar, and DIA (n = 3) (Independent Student’s *t* test). *E*, GO enrichment ranking of the proteome of mouse GV/MII oocyte and zygote spectra library, with the ranking order determined by the average abundance (log2 iBAQ) of proteins contained in each GO term. *F*, the number of proteins identified by DDA, BoxCar, and DIA in GO terms with proteins of different abundances (one-way ANOVA, Tukey’s multiple comparison test). *G*, the number of phosphopeptides identified by DDA and DIA in a single mouse MII oocytes (n = 3) (Two-tailed Student’s *t* test). ∗*p* < 0.05; ∗∗*p* < 0.01; ∗∗∗*p* < 0.001.

To improve single-cell proteomic analysis of oocytes, we evaluated the performance of BoxCar and DIA compared with DDA in protein identification and quantification using 10 ng mouse oocyte protein, whose protein amount is similar to one single mouse oocyte (68) (Fig. 1*A*). Using a DDA proteome spectral library containing 7132 proteins, 66,721 peptides, and 130,386 precursors (Supplemental Fig. S1*A*), we compared BoxCar and DDA and found that BoxCar identified more proteins than DDA, but the number of peptides was similar to DDA, resulting in a lower average number of peptides per protein. A comparison of DIA with DDA or BoxCar showed that DIA identified the most proteins and peptides (Fig. 1, *B* and *C*). To evaluate the quantification accuracy, the coefficient of variances (CVs) for protein quantification was analyzed, and the results showed that DIA had lower CVs than DDA and BoxCar (Fig. 1*D*). Therefore, DIA performs best in protein identification and quantification at single mouse oocyte level.

In single oocytes, we were able to identify highly abundant proteins in terms such as “endoplasmic reticulum chaperone complex (rank:3)” and “ATPase activator activity (rank:356),” medium abundant proteins in terms such as “oocyte development (rank:872)” and “contractile actin filament bundle (rank:1542)”, and low abundant proteins in terms such as “meiotic nuclear division (rank:2578)” and “GTPase regulator activity (rank:3102)” according to the ranking of average protein abundances quantified in the constructed mouse proteome spectral library (Fig. 1*E* and Supplemental Table S1*C*). We further analyzed the protein coverage in the above terms in single oocyte proteomes and found that the three methods are similar for terms of high abundance such as “endoplasmic reticulum chaperone complex.” However, DIA identified more proteins than BoxCar and DDA for terms containing lower abundant proteins, such as “meiotic nuclear division” and “GTPase regulator activity” (Fig. 1*F*).

Furthermore, we analyzed the performance of phosphorylation identification in single mouse oocytes. The results showed that DIA performed better than DDA in phosphopeptide identification in a single mouse MII oocytes without phosphopeptide enrichment using a DDA phosphoproteome library containing 1357 phosphoproteins, 11,481 phosphopeptides, and 39,128 precursors (Fig. 1*G* and, Supplemental Fig. S1*B*). BoxCar was not evaluated because BoxCar-based phosphorylation identification method was currently unavailable (33). DIA could identify more phosphorylation sites than the other methods (Supplemental Fig. S2, *A* and *B*). And DIA was pursued in further single-cell proteomics investigation of mouse oocytes.

### Single-Cell Multi-Omics Profiling of Fresh, POA, and Melatonin-Treated Oocytes at mRNA, Protein Expression, and Phosphorylation Levels

**Fig. 2:**
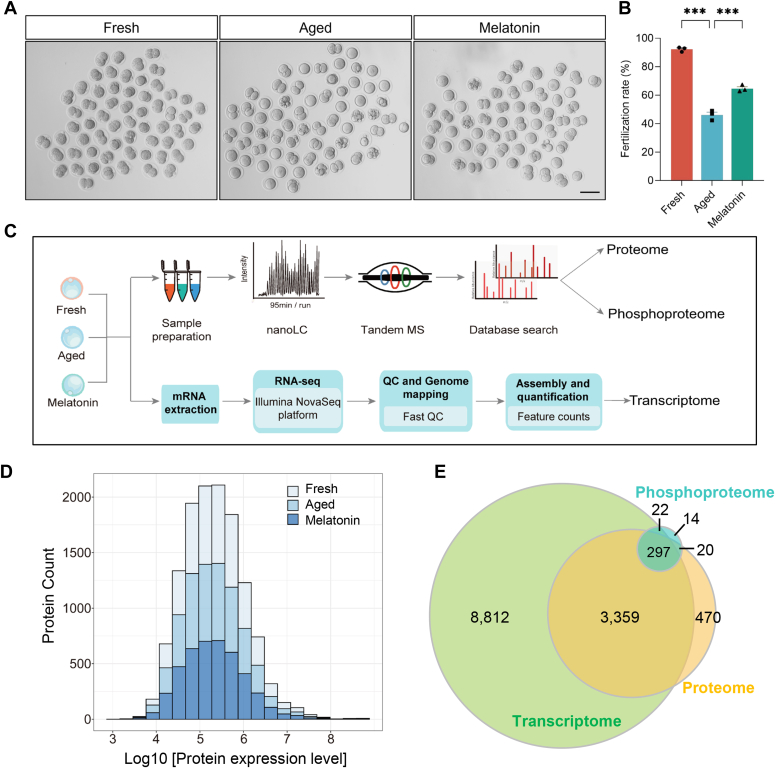
**Single-cell multi-omics profiling of fresh, POA, and melatonin-treated oocytes.***A*, representative images of *in vitro*–fertilized oocytes in fresh (fresh), POA (aged), and melatonin-treated (melatonin) oocytes. Scale bar represents 100 μm. *B*, *in vitro* fertilization rates of the oocytes in fresh (n = 169), aged (n = 162), and melatonin (n = 172) groups. Data are presented as mean ± SEM in three independent experiments. ∗∗∗*p* < 0.001 by one-way ANOVA, Tukey’s multiple comparison test. *C*, schematic workflow of the RNA-seq, proteomic, and phosphoproteomic profiling of fresh, aged, and melatonin-treated oocytes at single-cell level. *D*, dynamic range of protein quantification values without imputation in the oocytes of fresh, aged, and melatonin groups. *E*, Venn diagram showing the overlap among quantified genes in single-cell proteome, transcriptome, and phosphoproteome.

Melatonin is reported to be able to improve the quality of POA oocytes (18). Previous published dose-response analyses showed that 1 mM melatonin was most effective in increasing the fertilization rate and reducing the percentage of morphological abnormalities (11, 18) during post-ovulatory oocyte aging. To ensure optimal performance of melatonin treatment during post-ovulatory oocyte aging, we used 1 mM for subsequent experiments, and DMSO in a final concentration of 0.1% (v/v) was used as a co-solvent, which does not affect the quality of oocytes and was widely used in other studies (69). The aged group is cultured for 16 h in M2 medium also containing 0.1% DMSO as a control for melatonin-treatment. The results show that melatonin improved fertilization rate of the aged oocytes from 46.04 ± 3.49% to 64.53 ± 2.39% (Fig. 2, *A* and *B*). Melatonin treatment could also rescue the decreased ATP level in aged oocytes (Supplemental Fig. S3*A*) and meliorated the abnormal spindle assembly in aged oocytes from 48.95 ± 11.61% to 27.38 ± 5.19% (Supplemental Fig. S3, *B* and *C*). The analysis of molecular changes of melatonin-treated oocytes may help identify molecules important for oocyte quality improvement during POA.

With the optimization of single-cell proteomics method, we are able to profile the proteome and phosphoproteome of POA and melatonin-treated mouse oocytes in comparison with the transcriptome at single cell level. In order to investigate the mechanism of POA, nine fresh oocytes, nine aged oocytes, and nine melatonin-treated oocytes were collected to profile the single-cell DIA-based proteome and phosphoproteome. And seven MII oocytes of each group were subjected to Smart-seq2 single-cell RNA-seq analysis (Fig. 2*C* and Supplemental Table S2). Totally, 4704 oocyte proteins were identified in single-cell proteomics, and 4349 proteins (Supplemental Table S3) were quantified in at least half of the samples in one groups, with an average of 3837 proteins per cell and 3730 ± 17, 3959 ± 17, and 3822 ± 20 proteins in fresh, aged, and melatonin groups, respectively (Supplemental Fig. S4*A*). The overall protein expression levels spanned from 10ˆ3 to 10ˆ8 (Fig. 2D). Reproducibility analysis of protein quantification indicated high reproducibility, with pairwise Pearson’s correlation coefficients ranging from 0.951 to 0.984, 0.975 to 0.985, and 0.961 to 0.982 among oocytes in fresh, aged, and melatonin groups, respectively (Supplemental Fig. S4, *B*–*D*). Compared with previously published data on single mouse MII oocytes (30, 31) and that the vast majority of the 2335 proteins confidently identified by both Jiang *et al.* and He *et al.*, 2266 (97.0%) were also identified in our single mouse oocytes data, and 1074 proteins were not identified in these two studies, indicating the confidence and depth of our data (Supplemental Fig. S4*E*). Single-cell phosphoproteomics identified 1472 nonredundant phosphopeptides and quantified 620 phosphopeptides (Supplemental Table S4) containing a single phosphorylation site corresponding to 353 phosphoproteins without phosphopeptide enrichment in at least half of the samples in one group. Correlations analysis of phosphopeptide quantification indicated high reproducibility, with pairwise Pearson’s correlation coefficients ranging from 0.818 to 0.960, 0.915 to 0.968, and 0.861 to 0.956 among single oocytes in fresh, aged, and melatonin groups, respectively (Supplemental Fig. S5, *A*–*C*). Distribution analysis of phosphorylated amino acids indicated that the percentages of phosphoserine (pS), phosphothreonine (pT), and phosphotyrosine (pY) were 72.74%, 22.52%, and 4.74%, respectively (Supplemental Fig. S5*D*). Single-cell RNA-seq identified 12,633 genes in oocytes. And the majority of phosphoproteins (297/353, 84.14%) and proteins (3359/4,146, 81.02%) were identified in the transcriptome (Fig. 2*E*).

### Melatonin Improved the Quality of POA Oocytes at Protein but not mRNA Level

**Fig. 3:**
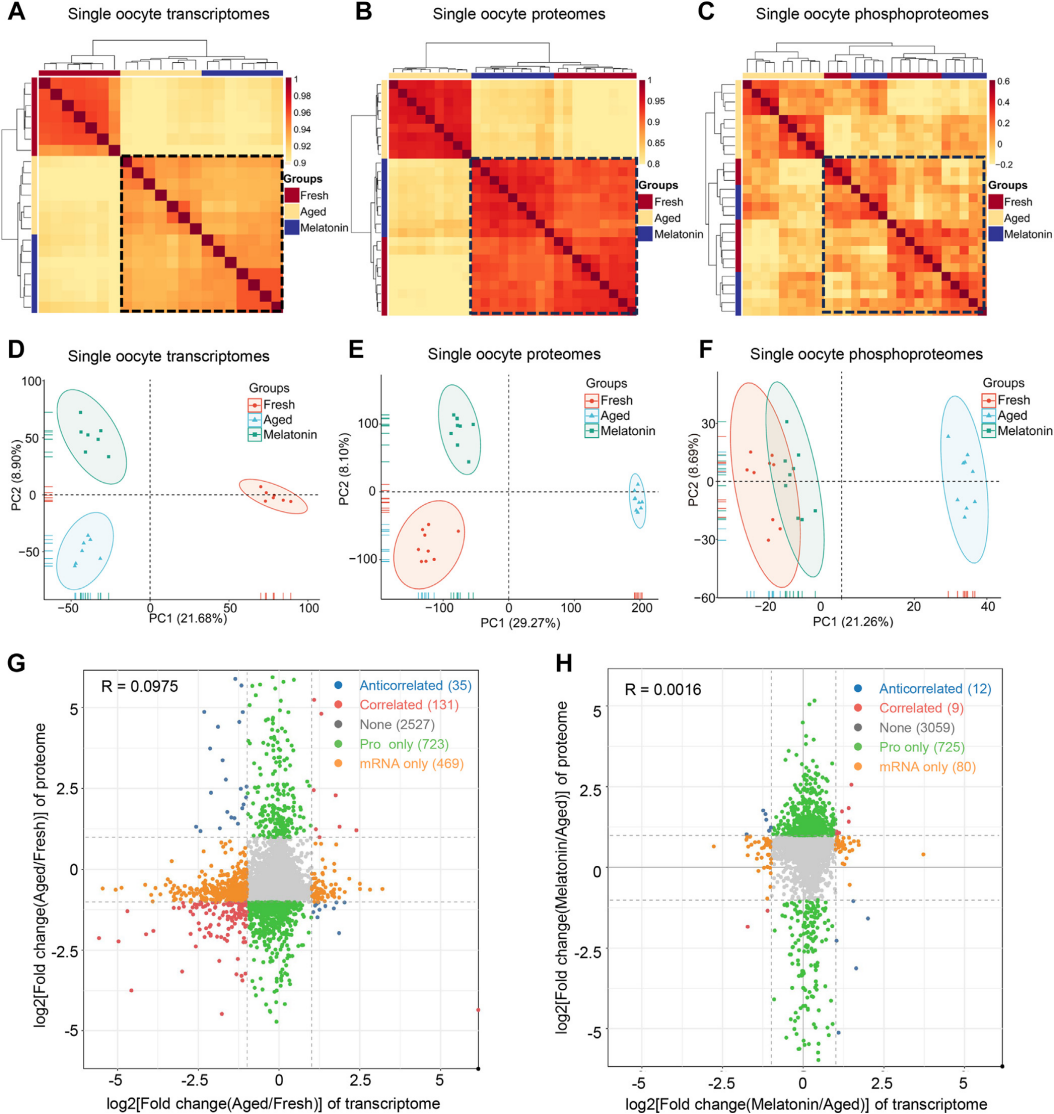
**Correlation analysis on fresh, POA, and melatonin-treated oocytes at different single-cell omics levels.***A*–*C*, hierarchical clustering and Pearson correlation analyses of quantitative transcriptomic (*A*), proteomic (*B*), and phosphoproteomic (*C*) data of single-cell oocytes in fresh, aged, and melatonin groups. *D*–*F*, PCA analyses of quantitative single-cell transcriptomic (*D*), proteomic (*E*), and phosphoproteomic (*F*) data of oocytes in fresh, aged, and melatonin groups. *G*, scatter plot of the log2-transformed fold changes (aged/fresh) between the single-cell proteome and transcriptome of oocytes. *H*, scatter plot of the log2-transformed fold changes (melatonin/aged) between the single-cell proteome and transcriptome of oocytes.

With the quantification of transcripts, proteins, and phosphorylation levels, we can evaluate the abnormalities of POA oocytes at molecular levels and characterize the molecular basis of quality improvement after melatonin treatment. We performed enhanced hierarchical clustering and Pearson’s correlation analysis to analyze the similarities of the transcriptome, proteome, and phosphoproteome among the fresh, aged, and melatonin-treated oocytes. For the transcriptome, the correlation coefficients between melatonin-treated oocytes and POA oocytes are higher than those between melatonin-treated oocytes and fresh oocytes, indicating a little effect of melatonin in rescuing the aged oocytes at the transcriptome level (Fig. 3*A*). For the proteome, melatonin-treated oocytes are more similar to the fresh oocytes other than aged oocytes (Fig. 3*B*). And for the phosphoproteome, melatonin-treated oocytes are also more similar to fresh oocytes than aged oocytes (Fig. 3*C*). Melatonin-treated oocytes are more similar to the fresh oocytes at protein expression and phosphorylation levels.

Additionally, we performed PCA analysis of the oocytes at different omics levels. Based on the transcriptome data, the three groups of oocytes formed three distinct clusters with the cluster of melatonin-treated oocytes closer to that of the aged oocytes (Fig. 3*D*). While at the proteome level, the cluster of melatonin-treated oocytes was closer to that of fresh oocytes (Fig. 3*E*). At the phosphorylation level, the clusters of melatonin-treated and fresh oocytes overlapped (Fig. 3*F*). These results are similar to those by correlation analysis, supporting the important roles of melatonin in improving oocyte quality at protein level rather than the transcript level.

Considering different effects of melatonin treatment on transcriptome and proteome or phosphoproteome levels, we analyzed correlations between the transcriptome and proteome across groups (aged *versus* fresh, melatonin *versus* aged). The correlation coefficient between the transcriptome and proteome during *in vitro* aging (aged *versus* fresh) was 0.0975. Among the genes identified in both transcriptome and proteome, only 18.67% of differential proteins (166/889) showed differential expression at the transcriptome level (Fig. 3*G*). And in the melatonin *versus* aged group, only 2.82% of differential proteins (21/746) have corresponding mRNA changes, with an overall correlation of 0.0016 between transcriptome and proteome (Fig. 3*H*). Little correlation between transcriptome and proteome during POA or melatonin treatment indicated complex posttranscriptional regulations, and it is important to directly study oocyte POA at the protein level. During POA, we still observed that 131 differentially expressed genes during POA have correlated expression between the transcriptome and proteome. GO enrichment analysis of these genes (Supplemental Fig. S6*A* and Supplemental Table S5*A*) was performed and revealed enrichment of "mitochondrial respiratory chain complex assembly," "mitotic spindle," " fertilization," and "protein processing." In addition, oxidative phosphorylation–related terms were also significantly enriched. In the term of “mitochondrial respiratory chain complex I assembly,” NDUFC2, NDUFAF3, NDUFAF5, and NDUFAF7 proteins, which are involved in NADH-ubiquinone oxidoreductase (complex I) assembly, were downregulated in the *in vitro* POA oocytes (Supplemental Fig. S6*B*). KEGG pathway analysis (Supplemental Table S5*B*) identified enriched pathways of "Oocyte meiosis" and "Progesterone-mediated oocyte maturation." The functions of genes in these terms or pathways are consistent with our observed phenotypes of POA oocytes including abnormal spindle of MII oocytes, reduced fertilization ability and abnormal embryo development, and the reported abnormal mitochondrial morphology (70). The genes with consistent expression changes at both transcriptome and proteome levels may also regulate the process of POA.

### Gene Ontology Analysis of Differential Oocyte Proteins during POA

**Fig. 4:**
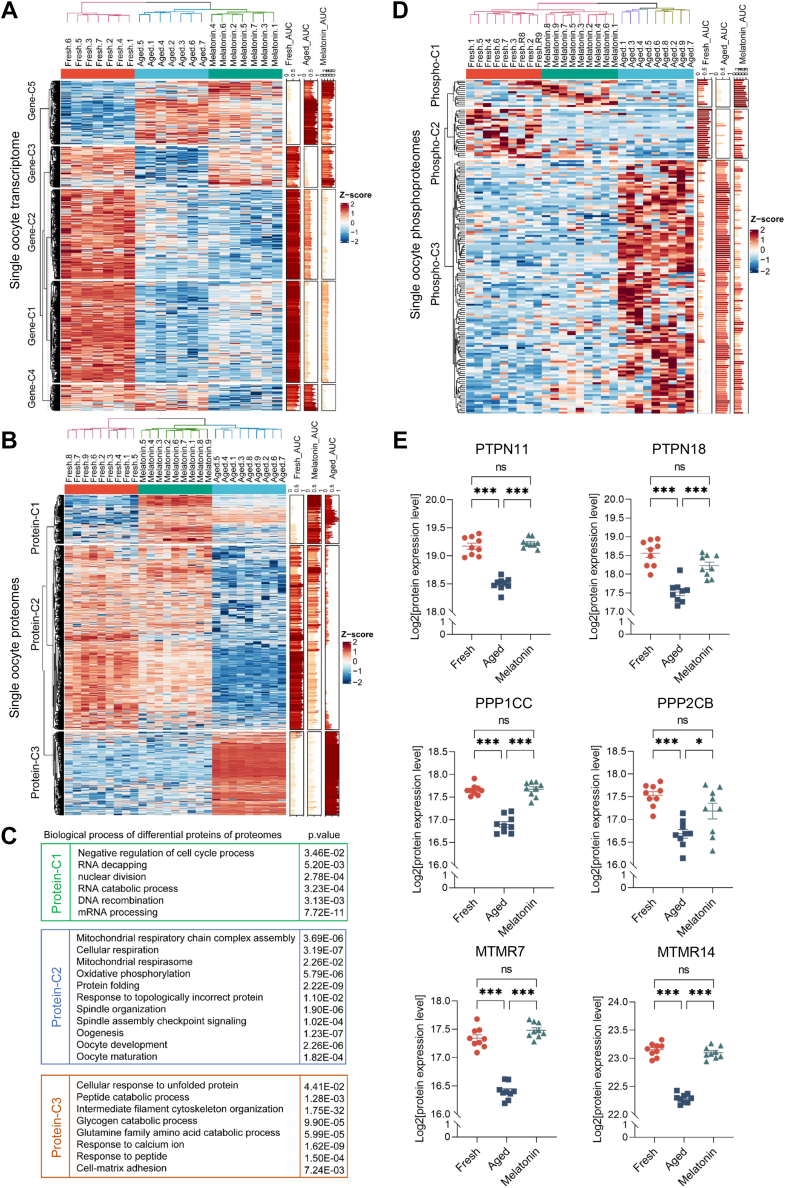
**Heatmap and functional enrichment analysis of differentially expressed oocyte proteins among groups.***A* and *B*, K-means clustering and heatmap of differentially expressed genes (*A*) and differentially expressed proteins (*B*) among fresh, aged, and melatonin groups. *C*, the enriched biological processes analysis of differentially expressed proteins from C1, C2, and C3 clusters. *D*, K-means clustering and heatmap of phosphopeptides with differential levels among fresh, aged, and melatonin groups. *E*, the protein expression levels of phosphatases PTPN11, PTPN18, PPP1CC, PPP2CB, MTMR7, and MTMR14 in oocytes from fresh, aged, and melatonin groups. ∗*p* < 0.05, ∗∗∗*p* < 0.001 by one-way ANOVA with Tukey’s multiple comparison test.

Among fresh, aged, and melatonin-treated oocyte groups, we identified 2350 differentially expressed genes (Supplemental Table S2), 1195 differential expressed proteins (Supplemental Table S3), and 168 differentially regulated phosphopeptides (One-way ANOVA, FDR-*q* < 0.05, fold change >2) (Supplemental Table S4). To analyze the functions of genes or proteins regulated after POA or melatonin treatment, we performed heatmap and GO enrichment analysis (Table S6). The differentially regulated genes could be classified into 5 clusters (Fig. 4*A*). Majority of differential genes (1694/2350, 72.09%%, Clusters gene-C1, gene-C2, and gene-C3) were downregulated in aged oocytes, indicating mRNA degradation during POA. Nevertheless, most of the downregulated genes were not rescued after melatonin treatment. Melatonin had little effect on rescuing the transcript level during POA.

We further analyzed the expression patterns of proteins regulated after POA or melatonin treatment. The differentially expressed proteins showed three distinct clusters (Fig. 4*B*). Proteins in Cluster protein-C3 (316/1,195, 26.44%) were abnormally upregulated in the aged oocytes and recovered to normal expression levels close to those in the fresh oocytes after melatonin treatment. Cluster protein-C2 constitutes majority of differentially expressed proteins (696/1,195, 58.24%). Proteins in Cluster protein-C1 were downregulated after *in vitro* aging and rescued to the level similar to the fresh oocytes by melatonin. However, we also observed a small proportion of proteins (Cluster protein-C1 183/1,195, 15.31%) that showed similar expression levels in the fresh and aged oocytes but were abnormally upregulated in the melatonin-treated oocytes. Thus, most of the differentially expressed proteins are downregulated during POA. Melatonin can rescue almost all the differentially expressed proteins to the level of fresh oocytes, with abnormal upregulation of a small proportion of proteins.

To characterize the functions of differentially expressed proteins, we performed GO analysis (Fig. 4*C*). The results revealed that proteins in Cluster protein-C2, the largest cluster, were enriched in terms of "mitochondrial respiratory chain complex assembly," "cellular respiration," and "mitochondrial respirasome," indicating abnormal mitochondrial functions in aged oocytes. They are also enriched in terms related to "protein folding," indicating possible decreased protein folding activity after POA. Additionally, we also observed enriched terms related to "spindle organization" and "oocyte development," consistent to our observation of abnormal spindle assembly and oocyte development in aged oocytes. Proteins in Cluster protein-C3 were enriched in protein quality-related terms, such as "cellular response to unfolded protein" and "peptide catabolic process." The downregulation of proteins involved in protein folding and upregulation of proteins in response to unfolded protein suggested that protein folding may be impaired during POA, resulting in active protein degradation and downregulation of the majority of differentially expressed proteins.

For the differentially regulated phosphopeptides, most of which (129/168, 76.79%) were abnormally elevated during aging and returned to the level of fresh oocytes in melatonin-treated oocytes (Fig. 4*D*). Phosphorylation is catalyzed by kinases and removed by phosphatases, indicating that the abnormal upregulation of phosphopeptides may be caused by decreased phosphatase activity during aging. Indeed, we identified phosphatases (71, 72), such as PTPN11, PTPN18, PPP1CC, PPP2CB, MTMR7, and MTMR14, to be downregulated in aged oocytes, which were successfully rescued to the level of fresh oocytes by melatonin treatment (Fig. 4*E*).

### Maintaining Protein Stability Improves the Quality of Oocytes during POA

**Fig. 5:**
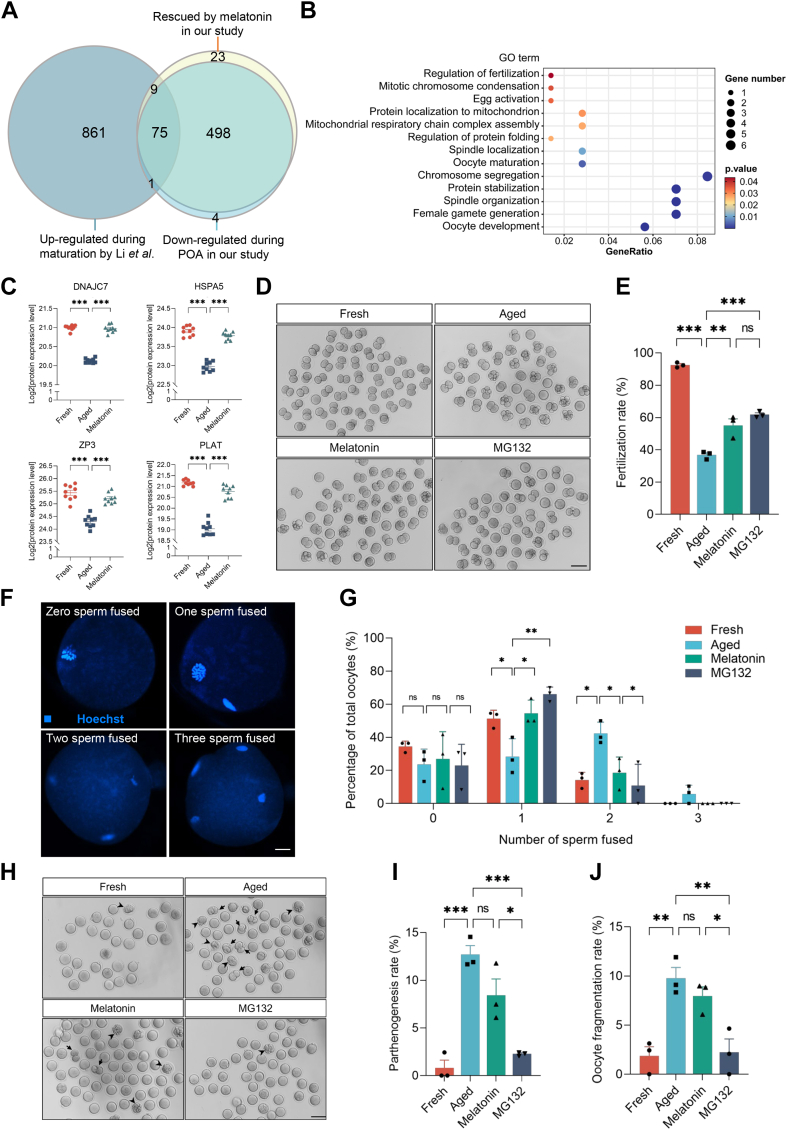
**MG132 treatment improved the quality of POA oocytes.***A*, numbers of rescued proteins by melatonin among proteins upregulated during meiotic maturation according to Li *et al*.’s study but downregulated during POA. *B*, column diagram of enriched GO terms of 75 rescued proteins by melatonin referred in (*A*). *C*, the protein expression levels of DNAJC7, HSPA5, ZP3, and PLAT in oocytes from fresh, aged, and melatonin groups. *D*, representative images of *in vitro*–fertilized oocytes in fresh, aged, melatonin and MG132 groups. Scale bar represents 100 μm. *E*, *in vitro* fertilization rates of the oocytes in fresh (n = 159), aged (n = 136), melatonin (n = 156), and MG132 (n = 152) groups. *F*, representative confocal images of *in vitro*–fertilized zona pellucida–free oocytes with zero, one, two, or three sperm fused. Scale bars represent 10 μm. *G*, percentage of oocytes with different numbers of fused sperm in fresh (n = 35), aged (n = 50), melatonin (n = 34), and MG132 (n = 35) groups. The x-axis indicates the number of sperm fused and the y-axis shows the percentage of oocytes with distinct numbers of fused sperm. *H*, representative images of parthenogenetic and fragmented oocytes in fresh, aged, melatonin, and MG132 groups. Arrows indicate the parthenogenetic oocytes and arrowheads indicate the fragmented oocytes. Scale bar represents 100 μm. *I* and *J*, the percentage of parthenogenetic (*I*) and fragmented (*J*) oocytes in fresh, aged, melatonin, and MG132 groups. Data are presented as mean ± SEM in three independent experiments. ∗*p* < 0.05, ∗∗*p* < 0.01, ∗∗∗*p* < 0.001 by one-way ANOVA with posthoc Tukey’s multiple comparison test.

As most proteins were downregulated after POA, we analyzed the expression of proteins actively translated during meiotic maturation from germinal vesicle to MII stages. Among 578 downregulated proteins during POA, 76 were reported to be upregulated and newly translated during oocyte meiotic maturation according to Li *et al*.’s study (73), and the expression of 75 (75/76, 98.68%) proteins were rescued by melatonin treatment (Fig. 5*A*). GO analysis revealed that these 75 proteins were enriched in terms related to oocyte development and protein processing, such as "oocyte maturation," "spindle organization," "regulation of fertilization," and "regulation of protein folding" (Fig. 5*B* and Supplemental Table S7). Among these rescued proteins, DNAJC7 and HSPA5 (74) are already known to be essential for protein folding and quality control. The defects of protein folding and quality control may lead to protein degradation (75). Besides, we also observed ZP3 (76), a protein critical for sperm-oocyte interaction during fertilization, and PLAT (77), a protein involved in polyspermy block during oocyte activation (Fig. 5*C*). The maintenance of these proteins’ stability may contribute to the effect of melatonin in rescuing the decreased fertilization rate and quality of POA oocytes.

Proteasome-mediated protein degradation is an important pathway for protein degradation. To test the role of proteasome-mediated protein degradation in POA, we utilized MG132, a reversible and cell-permeable proteasome inhibitor, to treat POA oocytes and assessed the fertilization rate of MG132-treated oocytes. The results showed that inhibition of proteasome activity by MG132 significantly improved fertilization rate of post-ovulatory oocytes (*p* < 0.01). And the fertilization rate of MG132-treated oocytes is similar to that of melatonin-treated oocytes (*p* > 0.05) (Fig. 5, *D* and *E*). We removed the zona pellucida and evaluated the effects of MG132 in reducing the polyspermy rate of aged oocytes. It indicated that MG132 could significantly reduce the polyspermy rate of aged oocytes compared with the POA group (*p* < 0.01), and the number of oocytes fertilized with two sperm was lower in the MG132-treated group than that in the melatonin-treated group (*p* < 0.05) (Fig. 5, *F* and *G*).

POA can induce high fragmentation and parthenogenesis rates, both of which were classical features of aged oocytes (78, 79). Thus, besides fertilization, we also analyzed parthenogenesis and fragmentation rates after POA. It indicated that MG132 could significantly downregulate both parthenogenesis (*p* < 0.001) and fragmentation (*p* < 0.01) rates, while melatonin had no effect in reducing parthenogenesis and fragmentation rates (*p* > 0.05) (Fig. 5, *H*–*J*). The parthenogenesis and fragmentation rates of MG132-treated oocytes were even similar to those of fresh oocytes (*p* > 0.05). Inhibition of proteasome-mediated protein degradation well improved oocyte fertilization rate and reduced polyspermy, parthenogenesis, and fragmentation rates.

**Fig. 6:**
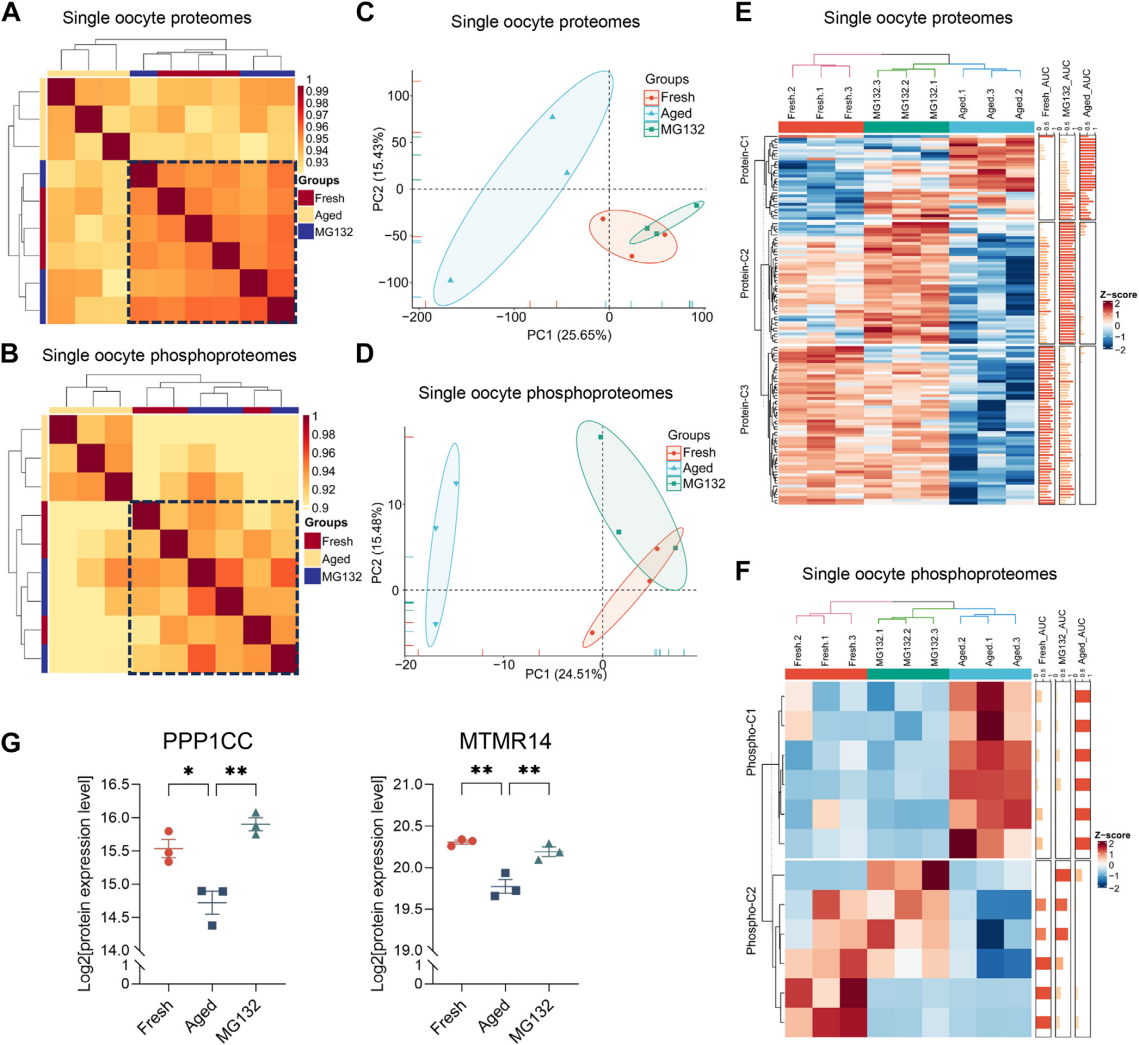
**Heatmap and functional enrichment analysis of differentially expressed oocyte proteins among fresh (fresh), post-ovulatory aged (aged), and MG132-treated (MG132) groups.***A* and *B*, hierarchical clustering and Pearson correlation analysis of quantitative proteomic (*A*) and phosphoproteomic (*B*) data of single-cell oocytes in fresh, aged, and MG132 groups. *C* and *D*, PCA analysis of quantitative proteomic (*C*) and phosphoproteomic (*D*) data of single-cell oocytes in fresh, aged, and MG132 groups. *E* and *F*, K-means clustering and heatmap of differentially expressed proteins (*E*) and phosphopeptides with differential levels among fresh, aged, and MG132 groups. *G*, the protein expression levels of phosphatases PPP1CC and MTMR14 in oocytes from fresh, aged, and MG132 groups. ∗*p* < 0.05, ∗∗*p* < 0.01 by one-way ANOVA with Tukey’s multiple comparison test.

Characterization of proteome and phosphoproteome can help us evaluate whether inhibition of proteasome using MG132 can restore the protein expression or even protein phosphorylation level during post-ovulatory culture. We performed single-cell proteomic analysis of oocytes from fresh, POA, and MG132-treated groups. Hierarchical clustering and Pearson’s correlation analysis showed that MG132-treated oocytes are more similar to the fresh oocytes with higher correlation coefficients at not only the proteome level but also the phosphoproteome level. POA oocytes showed a distinct cluster, while MG132-treated and fresh oocytes clustered together in a large cluster (Fig. 6, *A* and *B* and Supplemental Tables S8 and S9). PCA analysis also revealed the similarities between MG132-treated and fresh oocytes at both proteome and phosphoproteome levels (Fig. 6, *C* and *D*).

To further analyze the effects of MG132 on proteins with reduced expression during POA, we performed a heatmap analysis of differentially expressed proteins among groups. The results showed that MG132 could increase the expression level of most downregulated proteins in POA oocytes (Fig. 6*E*), indicating that the protein expression decrease during post-ovulatory culture is mainly due to protein degradation by proteasome. MG132 cannot directly regulate protein phosphorylation. To evaluate the phosphorylation changes after protein degradation perturbation, we further analyzed the levels of protein phosphopeptides among groups. For the phosphopeptides that were abnormally upregulated during post-ovulatory culture, their levels were also downregulated after MG132 treatment. Inhibition of proteasome can restore the phosphoproteome profile in post-ovulatory aged oocytes to the level of fresh oocytes (Fig. 6*F*). And similar to melatonin, MG132 treatment could rescue the decrease of PPP1CC and MTMR14 (Fig. 6*G*). Thus, the proteasome-mediated protein degradation plays important roles in the decreased quality of POA oocytes, and inhibition of proteasome-mediated protein degradation can improve the decreased quality of POA oocytes.

## Discussion

The molecular changes of POA have not been systemically investigated, especially at protein level (3). Using the optimized single-cell multi-omics methods, we characterized transcriptome, proteome, and phosphoproteome of POA oocytes and melatonin-treated oocytes. And the results showed the inconsistency between transcriptome and proteome. Protein degradation is an important mechanism of decreased quality during POA, and inhibition of the activity of proteasome using MG132 can well rescue the decreased oocyte quality by improving fertilization rate and inhibiting ratios of oocyte fragmentation, parthenogenetic activation, and occurrence of polyspermic fertilization during POA. MG132 treatment can also rescue the phosphoproteome disturbance during POA.

Comparison of different acquisition methods indicated that DIA performs better than BoxCar and DDA at both proteome coverage and CV of quantification. BoxCar method was reported to benefit for low-abundant protein identification, and it was demonstrated to perform better than DDA at both depth and reproducibility (52). As BoxCar can benefit the identification of low-abundant proteins for bulk samples, it seems that it might help improve single-cell proteomics analysis. Compared with DDA, BoxCar showed better depth in proteome coverage but higher CV. It seems that extremely low-abundant proteins at single-cell level will lead to a decrease of reproducibility of BoxCar. A direct comparison between BoxCar and DIA showed that DIA performs better than BoxCar at both depth and CV. Meanwhile, DIA can quantify low-abundant posttranslational modification, phosphorylation, at single-cell level. The improved algorithm for DIA data analysis might contribute to the better performance of DIA method (80). With the optimized single-cell DIA-based proteomics method, in total, we identified 4349 proteins and 1472 phosphopeptides and quantified 4349 proteins and 620 single-phosphosite phosphopeptides in single mouse oocytes.

Single-cell proteomics analysis showed that melatonin can restore the abnormal protein expression in oocytes during post-ovulatory culture. As a major pineal secretory hormone involved in the regulation of circadian rhythms and seasonal reproduction in animals (81), melatonin can improve oocyte quality and increase fertilization rates, ameliorate mitochondrial dysfunction, and maintain normal cellular energy supply (ATP) (82, 83) and reduce oxidative stress as a potent antioxidant (84). Consistently, we observed that melatonin supplementation improved oocyte quality, increased the fertilization rates, and facilitated the development of the early embryo during oocyte aging *in vitro*. Our single-cell proteomics analysis of oocytes showed that the majority of the differentially expressed proteins decreased during POA and were stabilized by melatonin treatment, while melatonin had little effect on the transcriptome level, indicating possible protein stabilization effect of melatonin on improving oocyte quality during POA. Proteins can be degraded by different pathways such as proteasome or autophagy, and melatonin may stabilize protein by inhibiting the proteasome pathway. Melatonin could induce the accumulation of p53 (85), JNK (86), and caspase-3 (87), which could also be induced by proteasome inhibitors (88). However, there is still lack of proteome scale evidence of proteins stability regulation by melatonin. Here the inhibition of proteasome using MG132 showed major roles of proteasome-mediated protein degradation in oocyte quality decrease during POA, because MG132 showed similar effects to melatonin in rescuing most of the proteome and phosphoproteome perturbations during POA and in reducing fragmented oocytes and solitary developing oocytes, decreasing incidence of polyspermia of oocytes without zona pellucida after POA. However, further studies are still needed to reveal whether the unstable proteins are selectively degraded by proteasome and which E3 ligases are involved in proteasome-mediated degradation during POA.

Single-cell proteomic analysis of the phosphorylation level revealed that most differentially phosphorylated proteins were abnormally upregulated during POA. Protein phosphorylation is essential in regulating oocyte meiotic maturation, fertilization, and mitosis (89, 90, 91). Our proteasome inhibition by MG132 treatment well rescued the abnormal upregulation of protein phosphorylation during POA. Protein phosphorylation is catalyzed by kinases and removed by phosphatases (92). Phosphatases including PTPN11, PTPN18, PPP1CC, PPP2CB, MTMR7, and MTMR14 were significantly downregulated in aged oocytes. The decreased activity of these phosphatases might lead to increased phosphorylation level of their substrate proteins during POA. Melatonin treatment and MG132 treatment both rescued the decrease of PPP1CC and MTMR14, and they both improved the phosphorylation level of aged oocytes to that of fresh oocytes.

Overall, DIA was proved to be more suitable for single-cell proteomic research. We performed single-cell multi-omics analysis on fresh, aged, and melatonin-treated oocytes and found that melatonin could improve aged oocyte qualities at the protein but not mRNA level. MG132-treated oocytes displayed health status similar to melatonin-treated oocytes at both proteome and phosphoproteome levels, suggesting protein stabilization could also rescue aged oocytes, which might be the underlying mechanism through which melatonin exerted its functions.

## Data Availability

The mass spectrometry proteomics data are available *via* ProteomeXchange with the identifier PXD045614 (Username: reviewer_pxd045614@ebi.ac.uk, Password: vj7tfArD).

The RNA seq data are available in the National Center for Biotechnology Information (NCBI) Short Read Archive database under project no. PRJNA1020598 (https://dataview.ncbi.nlm.nih.gov/object/PRJNA1020598?reviewer=dls0ggv07npt56c9r1u1qg251i).

## Supplemental data

This article contains supplemental data.

## Conflict of Interest

The authors declare no competing interests.
