# Supplementary material for: Single-Cell Multi-Omics Analysis of In Vitro Post-Ovulatory–Aged Oocytes Revealed Aging-Dependent Protein Degradation
Source: Mol Cell Proteomics. 2024 Nov 20;24(1):100882. doi: 10.1016/j.mcpro.2024.100882 (PMC11728983; doi:10.1016/j.mcpro.2024.100882)
Supplement: Supplemental Figures [file mmc1.pdf]

Supplemental Information for

Single-cell multi-omics analysis of in vitro post-ovulatory aged oocytes revealed aging-dependent protein degradation

Yueshuai Guo[1], Mengmeng Gao[1], Xiaofei Liu[1], Haotian Zhang[1], Yue Wang[1], Tong Yan[1], Bing Wang[1,2], Xudong Han[1,2], Yaling Qi[1], Hui Zhu[1], Chenghao Situ[1], Yan Li[3], Xuejiang Guo[1]

[1]State Key Laboratory of Reproductive Medicine and Offspring Health, Department of Histology and Embryology, Nanjing Medical University, Nanjing 211166, China

[2]School of Medicine, Southeast University, Nanjing 210009, China

[3]Department of Clinical Laboratory, Sir Run Run Hospital, Nanjing Medical University, Nanjing, 211166, China

Running title: Single-cell multi-omics analysis of mouse oocytes

Corresponding authors:

Xuejiang Guo, E-mail: guo_xuejiang@njmu.edu.cn;

Yan Li, E-mail: yanli@njmu.edu.cn;

Chenghao Situ, E-mail: situchenghao@njmu.edu.cn.

1. Supplementary Figures

Figure S1. Construction of spectral libraries for proteome and phosphoproteome of mouse oocytes and zygotes.

Figure S2. Identification of phosphorylation sites by DIA and DDA in single mouse oocytes.

Figure S3. Melatonin rescued the ATP decrease and meiotic spindle/chromosome abnormalities during POA.

Figure S4. Single-cell multi-omics profiling of fresh, aged and melatonin groups.

Figure S5. Single-cell phosphoproteomics analysis of fresh, aged and melatonin groups.

Figure S6. GO enrichment and KEGG pathway analysis of 131 genes with correlated expression between mRNA and protein levels in aged vs. fresh groups.

2. Supplementary Tables (separate files)

Supplementary Table S1a. Transmission windows for the DIA method.

Supplementary Table S1b. Isolation windows for the BoxCar method.

Supplementary Table S1c. Abundance ranking of GO enrichment of mouse oocyte proteins.

Supplementary Table S2. Quantification of mouse fresh, aged and melatonin-treated oocyte genes by single-cell RNA-seq.

Supplementary Table S3. Quantification of mouse fresh, aged and melatonin-treated oocytes proteins by single-cell proteomics.

Supplementary Table S4. Quantification of mouse fresh, aged and melatonin-treated oocyte phosphopeptides by single-cell phosphoproteomics.

Supplementary Table S5a. GO enrichment analysis of 131 differential expressed gene between fresh and aged in both the proteome and transcriptome.

Supplementary Table S5b. KEGG pathway analysis of 131 differential expressed gene between fresh and aged in both the proteome and transcriptome.

Supplementary Table S6. GO terms enriched in each of the 3 clusters of differentially expressed proteins among mouse fresh, aged and melatonin-treated oocytes.

Supplementary Table S7. GO terms enriched in 75 rescued proteins by melatonin in proteins newly translated during meiotic maturation but down-regulated during POA.

Supplementary Table S8. Quantification of mouse fresh, aged and MG132-treated oocyte proteins by single-cell proteomics.

Supplementary Table S9. Quantification of mouse fresh, aged and MG132-treated oocyte phosphopeptides by single-cell phosphoproteomic analysis.

3. Supplementary Data (separate files)

Supplementary Data 1. Annotated spectra for proteins identified by a single peptide in Table S3.

Supplementary Data 2. Annotated spectra for proteins identified by a single peptide in Table S8.

Supplemental Figure 1

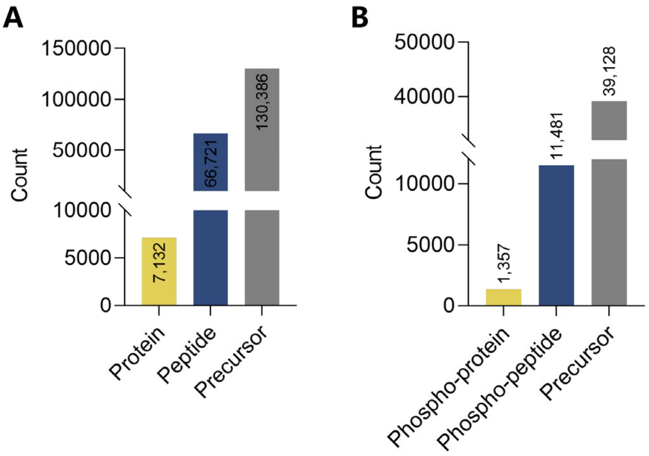

Figure S1. Construction of spectral libraries for proteome and phosphoproteome of mouse oocytes and zygotes. (A) The identification of proteins, peptides and precursors in the proteome spectral library of mouse oocytes and zygotes. **(B)** The identification of phosphoproteins, phosphopeptides and precursors in the phosphoproteome spectral library of mouse oocytes and zygotes.

Supplemental Figure 2

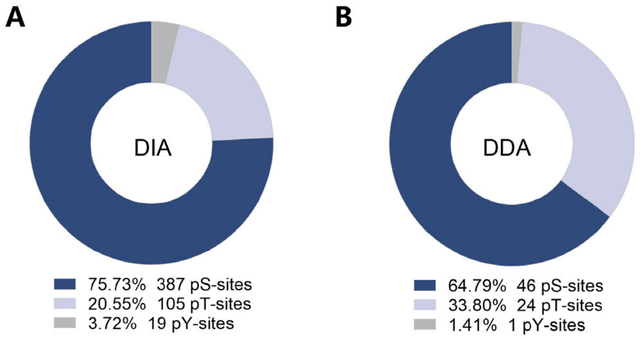

Figure S2. Identification of phosphorylation sites by DIA and DDA in single mouse oocytes. The number of phosphoserine sites (pS-sites), phosphothreonine sites (pT-sites), and phosphotyrosine sites (pY-sites) identified in single oocytes by DIA **(A)** and DDA methods **(B)** (n = 3).

Supplemental Figure 3

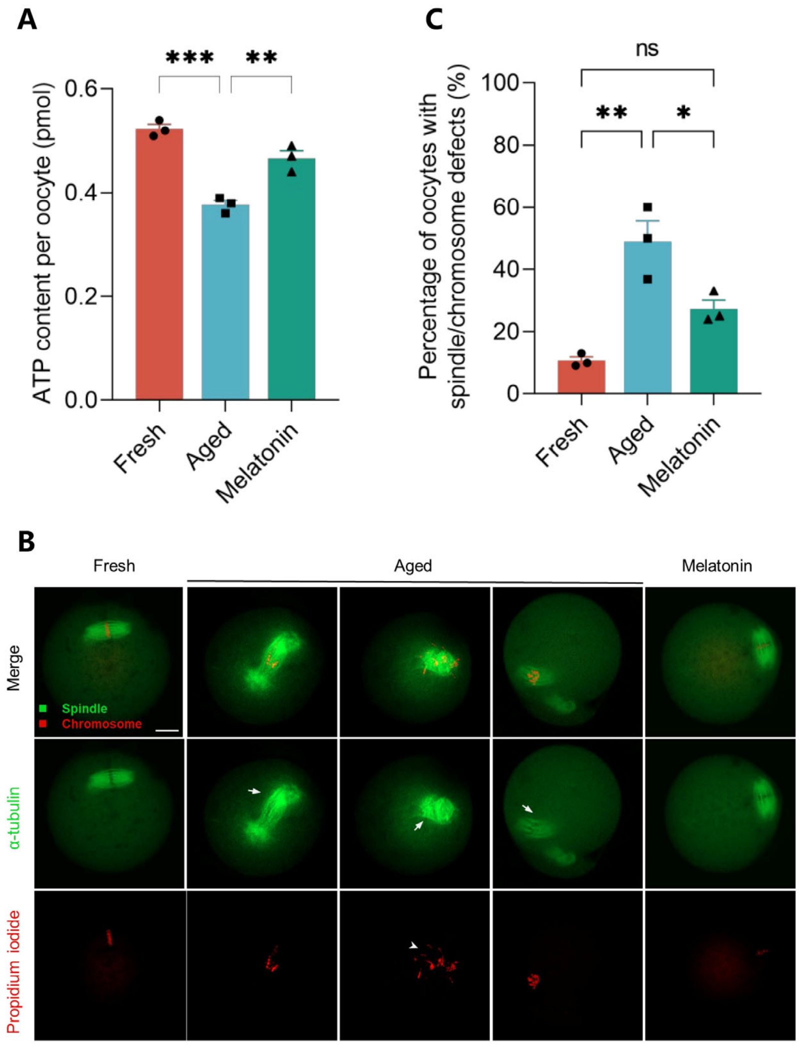

Figure S3. Melatonin rescued the ATP decrease and meiotic spindle/chromosome abnormalities during POA. (A) ATP content per oocyte in fresh, aged, and melatonin groups. **(B)** Representative confocal images of meiotic spindle and chromosomes at MII stage of oocytes in fresh, aged, and melatonin groups. The arrowhead indicates the misaligned chromosomes and arrows indicate the defect spindles. Scale bars, 10 µm. **(C)** Percentage of oocytes in Fresh (n=65), Aged (n=63), and Melatonin (n=72) groups with spindle/chromosome defects. Data are presented as mean ± SEM in three independent experiments. $*P < 0.05$, $**P < 0.01$, $***P < 0.001$ by one-way ANOVA with posthoc Tukey's multiple comparison test.

Supplemental Figure 4

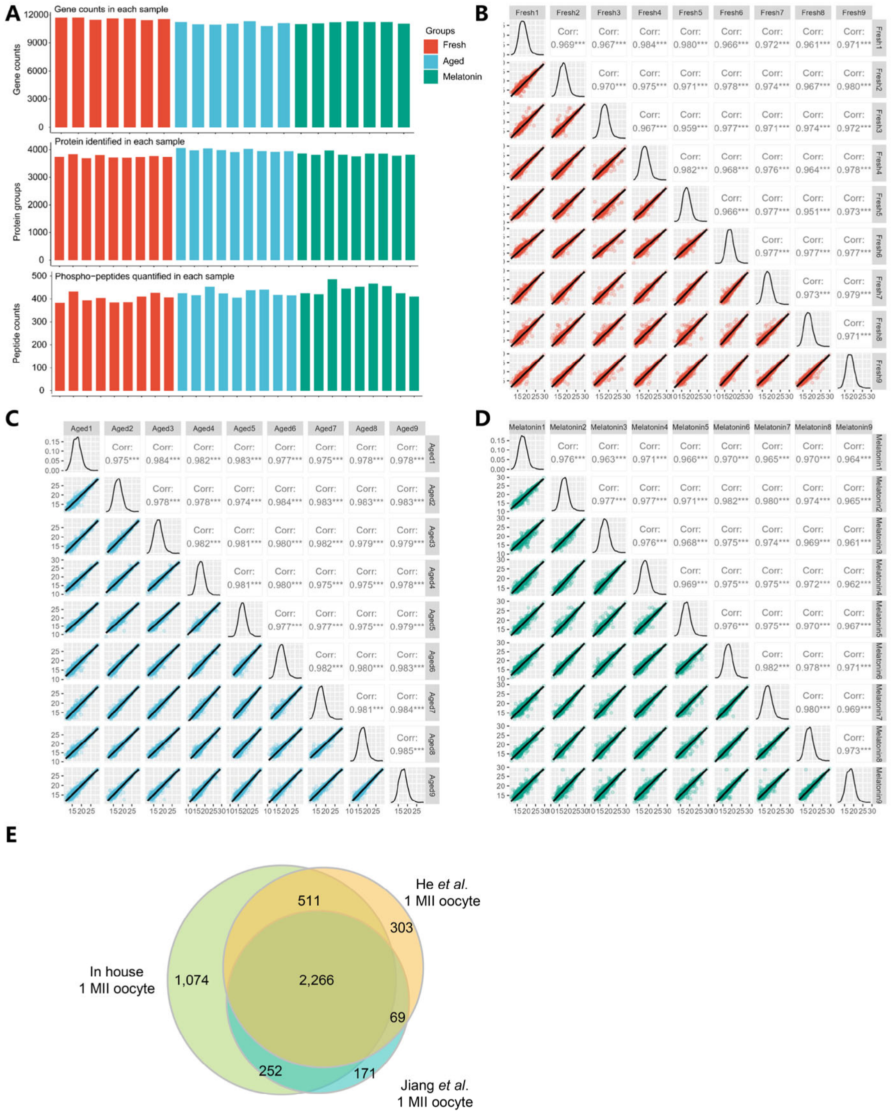

Figure S4. Single-cell multi-omics profiling of fresh, aged and melatonin groups. (A) The numbers of genes, proteins and phosphopeptides identified in fresh, aged, and melatonin groups at single-cell level. **(B-D)** Scatter plots and Pearson correlation analysis of single-cell oocyte proteomes among fresh **(B)**, aged **(C)**, melatonin **(D)** groups. The horizontal and vertical axes represent the log2-transformed protein quantification values without imputation. The scatter plot of protein quantification values of paired cells is shown in the lower left corner, and the Pearson correlation coefficient of paired cells is shown in the upper right corner. **(E)** Venn diagram showing the overlap between our data, He *et al.*'s and Jiang *et al.*'s single mouse MII oocyte data.

Supplemental Figure 5

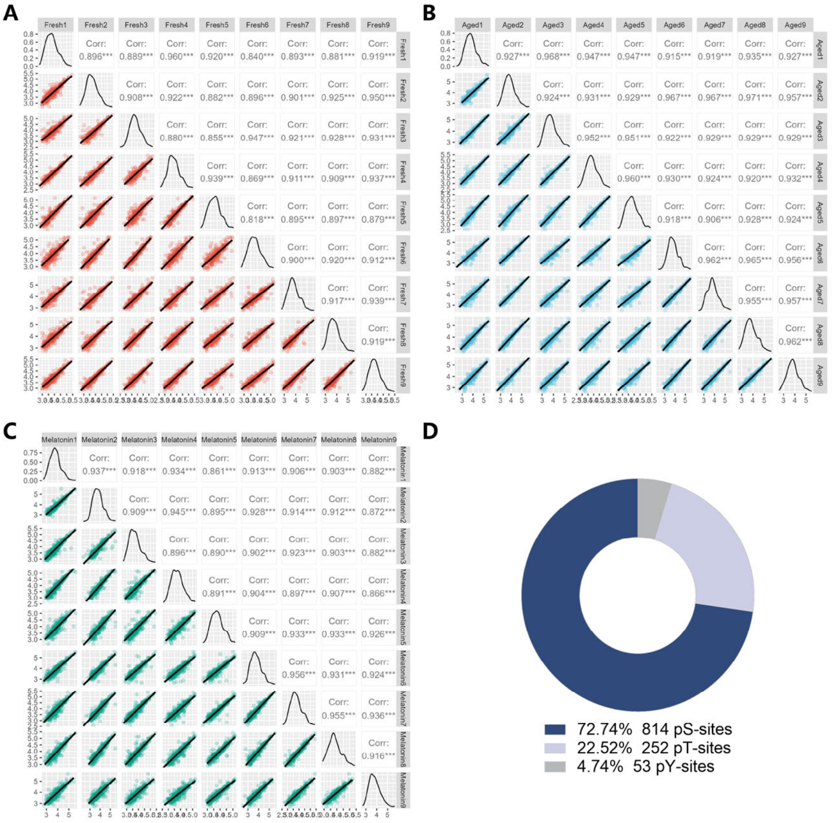

Figure S5. Single-cell phosphoproteomics analysis of fresh, aged and melatonin groups. (A-C) Scatter plots and Pearson correlation analysis of single-cell oocyte phosphoproteomes among fresh **(A)**, aged **(B)**, melatonin **(C)** groups. The horizontal and vertical axes represent the log2-transformed phosphopeptides quantification values without imputation. The scatter plot of phosphopeptides quantification values of paired cells is shown in the lower left corner, and the Pearson correlation coefficient of paired cells is shown in the upper right corner. **(D)** The total number of phosphoserine sites (pS-sites), phosphothreonine sites (pT-sites), and phosphotyrosine sites (pY-sites) identified in single oocytes of fresh, aged, and melatonin groups.

Supplemental Figure 6

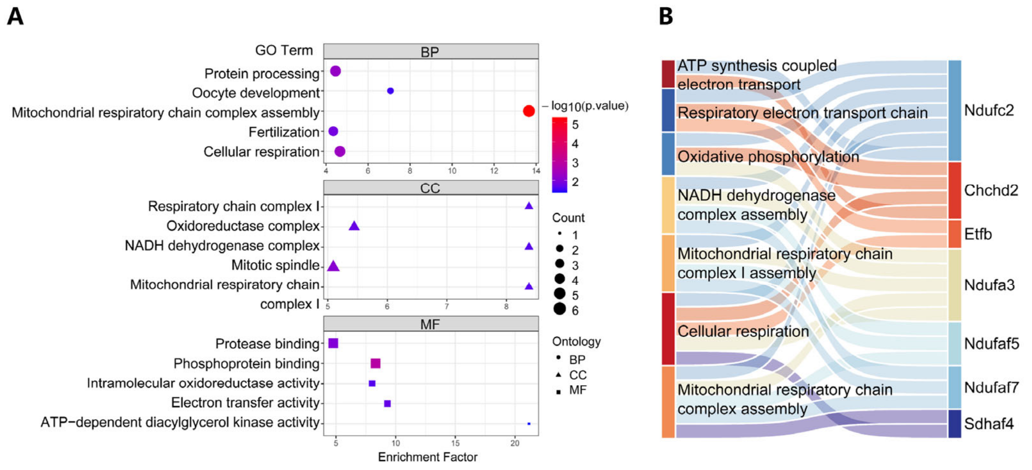

Figure S6. GO enrichment and KEGG pathway analysis of 131 genes with correlated expression between mRNA and protein levels in aged vs. fresh groups. GO enrichment analysis of 131 differential expressed proteins between fresh and aged in both the proteome and transcriptome **(A)** and the relationship between differential expressed proteins and GO terms related to oxidative phosphorylation **(B)**.
