# Supplementary material for: Single-Cell Multi-Omics Analysis of In Vitro Post-Ovulatory–Aged Oocytes Revealed Aging-Dependent Protein Degradation
Source: Mol Cell Proteomics. 2024 Nov 20;24(1):100882. doi: 10.1016/j.mcpro.2024.100882 (PMC11728983; doi:10.1016/j.mcpro.2024.100882)
Supplement: Supplementary Data 1 [file mmc2.pdf]

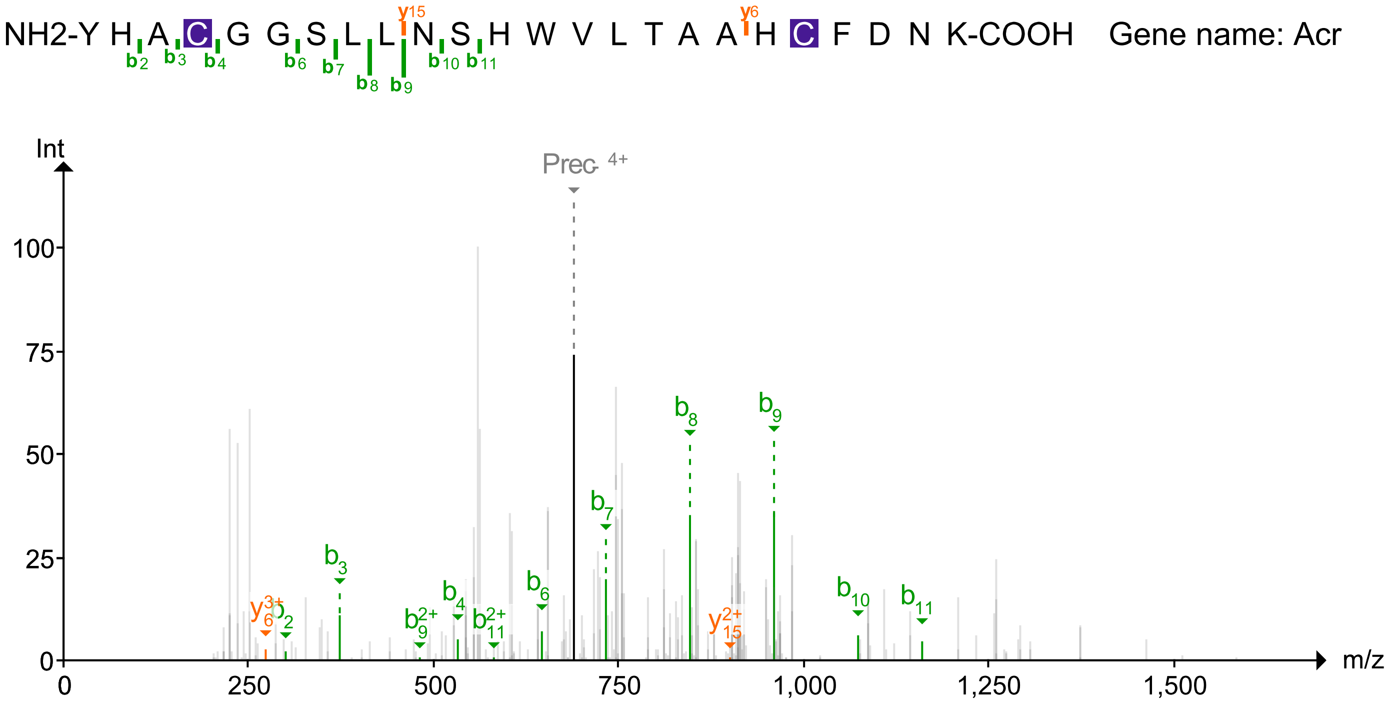

NH2-Y H A C G G S L L N S H W V L T A A H C F D N K-COOH
Gene name: Acr
b2 b3 b4 b6 b7 b8 b9 b10 b11
y15 y6
Int
Prec 4+
100
75
50
25
0
b8
b9
b7
b3
b6
b4
b10
b11
y6 3+
b2
b9 2+
b11 2+
y15 2+
0
250
500
750
1,000
1,250
1,500
m/z

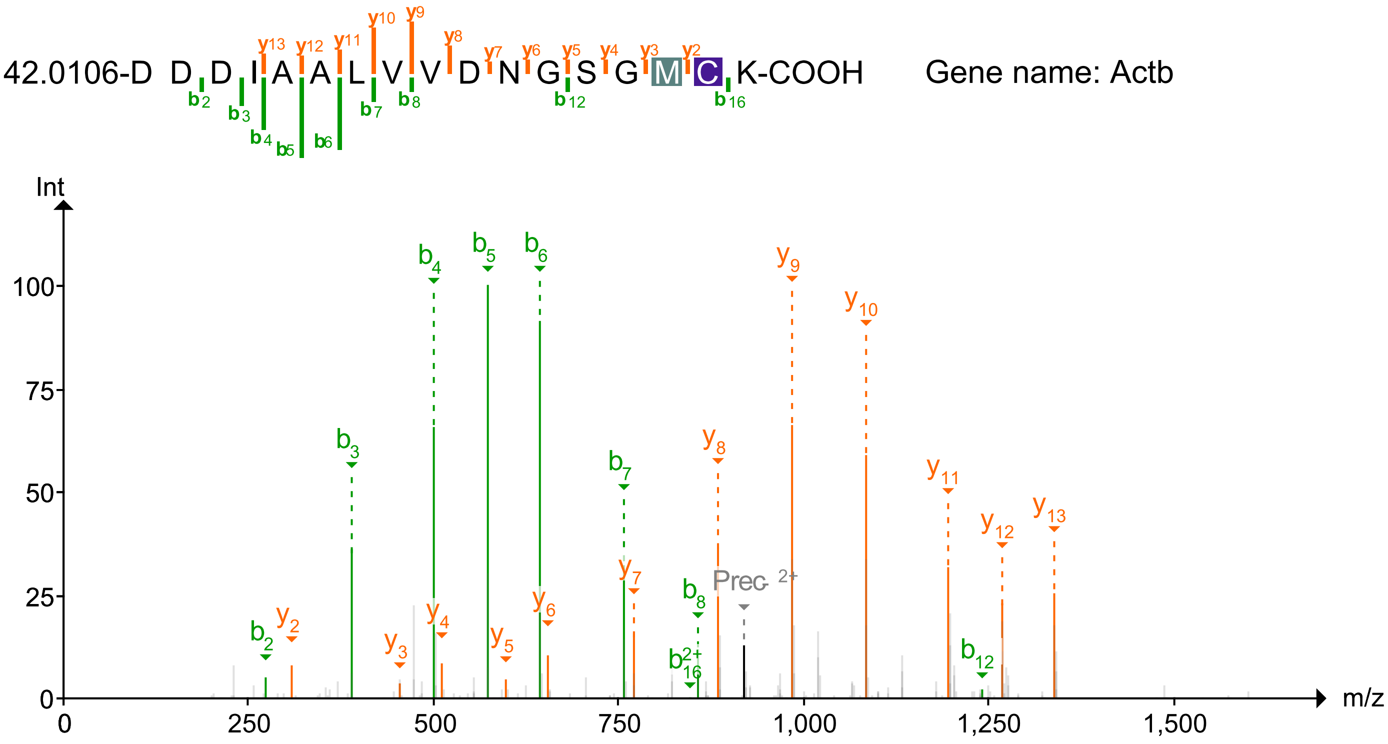

42.0106-D D D I A A L V V D N G S G M C K-COOH
Gene name: Actb
Int
m/z
Prec 2+

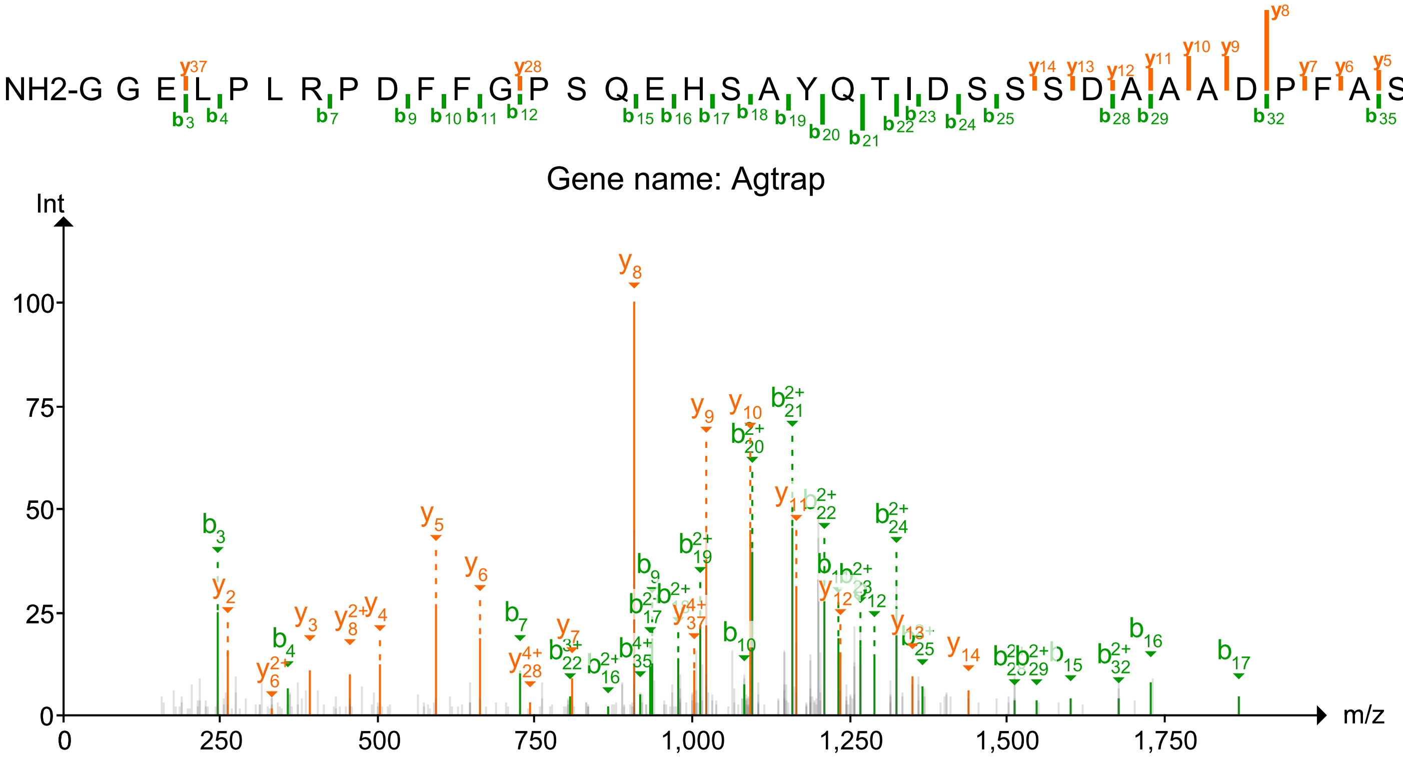
NH2-G G E L P L R P D F F G P S Q E H S A Y Q T I D S S S D A A A D P F A S
Gene name: Agtrap
Int
m/z
0
25
50
75
100
250
500
750
1,000
1,250
1,500
1,750

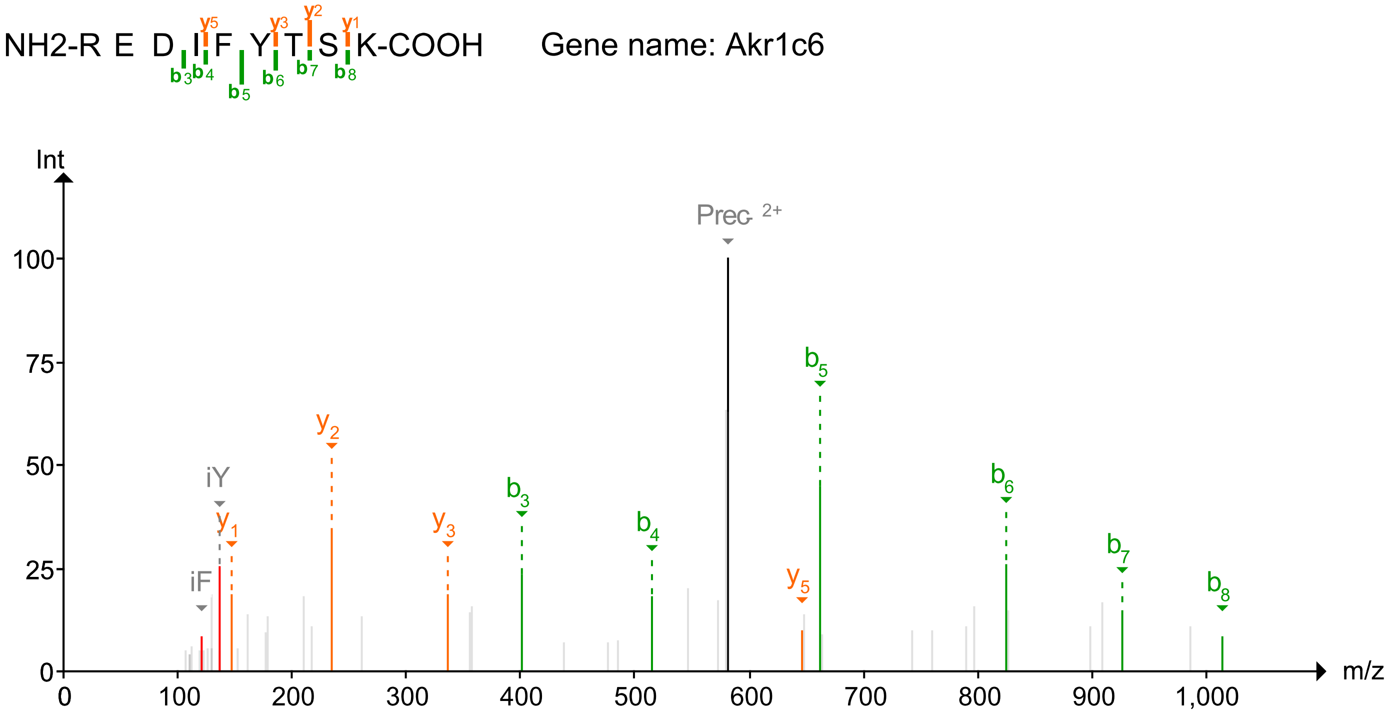
NH2-R E D I F Y T S K-COOH
Gene name: Akr1c6
Int
m/z
Prec 2+
iY
iF
y1
y2
y3
y5
b3
b4
b5
b6
b7
b8
0
25
50
75
100
0
100
200
300
400
500
600
700
800
900
1,000

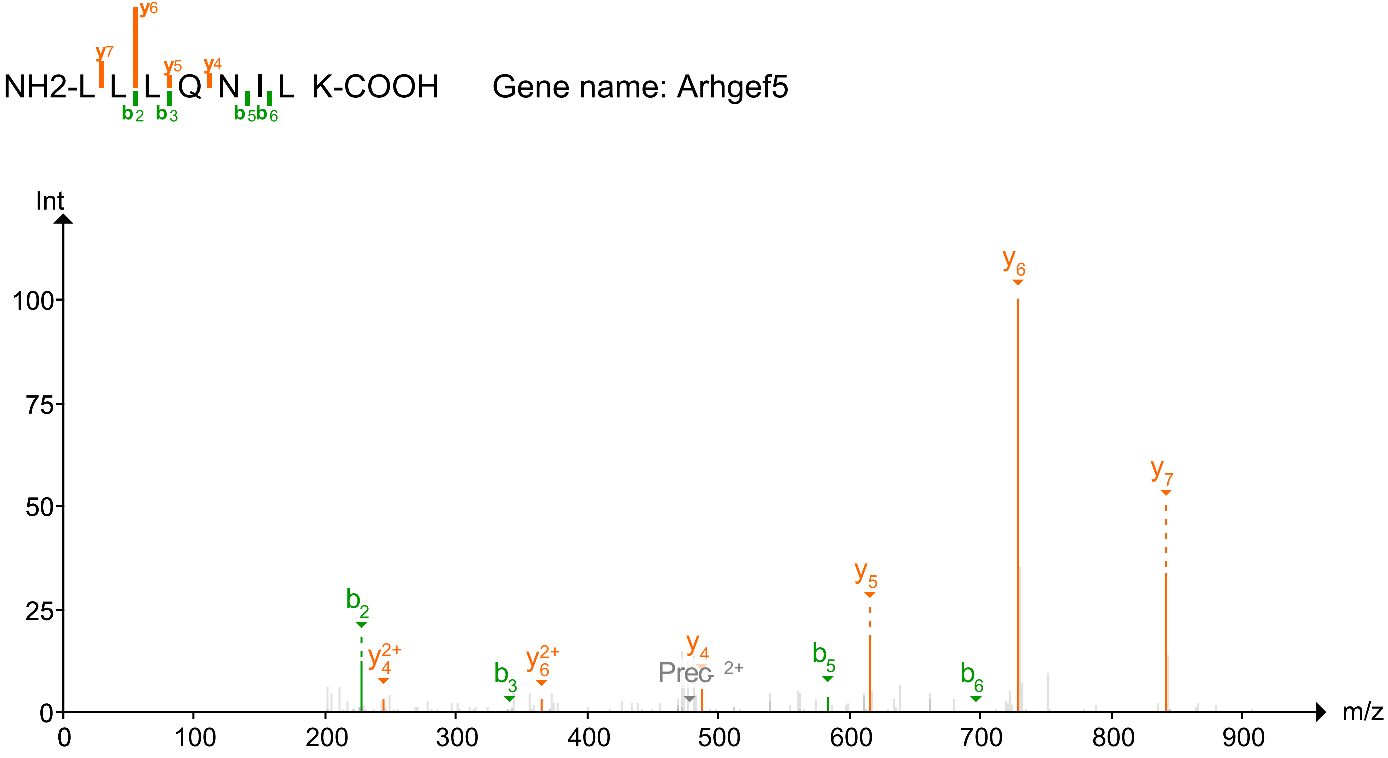

NH2-L L L Q N I L K-COOH
Gene name: Arhgef5
y7 y6 y5 y4
b2 b3 b5 b6
Int
100
75
50
25
0
m/z
0 100 200 300 400 500 600 700 800 900
b2
y4 2+
b3
y6 2+
y4
Prec 2+
b5
y5
b6
y6
y7

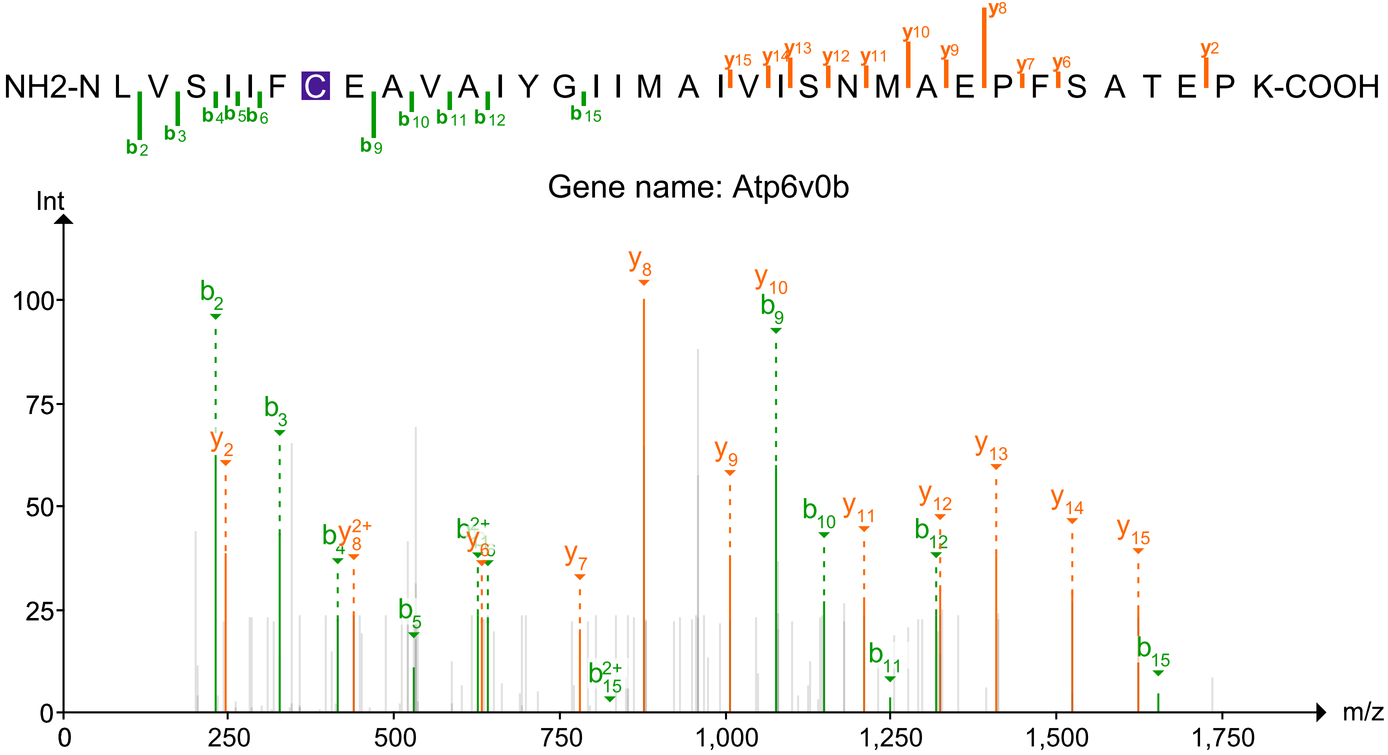
NH2-N L V S I I F C E A V A I Y G I I M A I V I S N M A E P F S A T E P K-COOH
Gene name: Atp6v0b
Int
m/z
0
25
50
75
100
0
250
500
750
1,000
1,250
1,500
1,750
b2
b3
b4
b5
b6
b9
b10
b11
b12
b15
y2
y6
y7
y8
y9
y10
y11
y12
y13
y14
y15

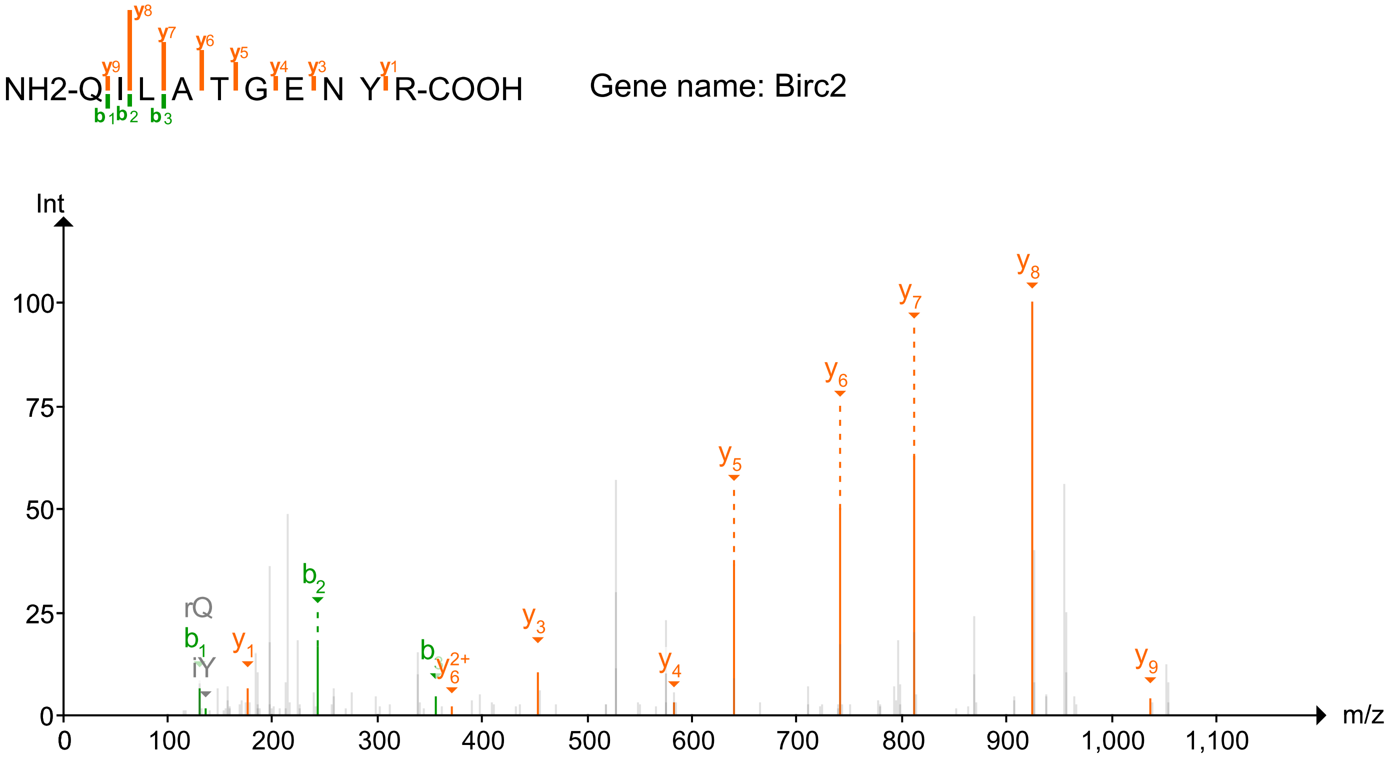

NH2-QILATGENYR-COOH
Gene name: Birc2
Int
m/z
100
75
50
25
0
0
100
200
300
400
500
600
700
800
900
1,000
1,100
y8
y7
y6
y5
y3
y4
y1
y9
b2
rQ
b1
iY
y2+ 6
y3
y4
y9

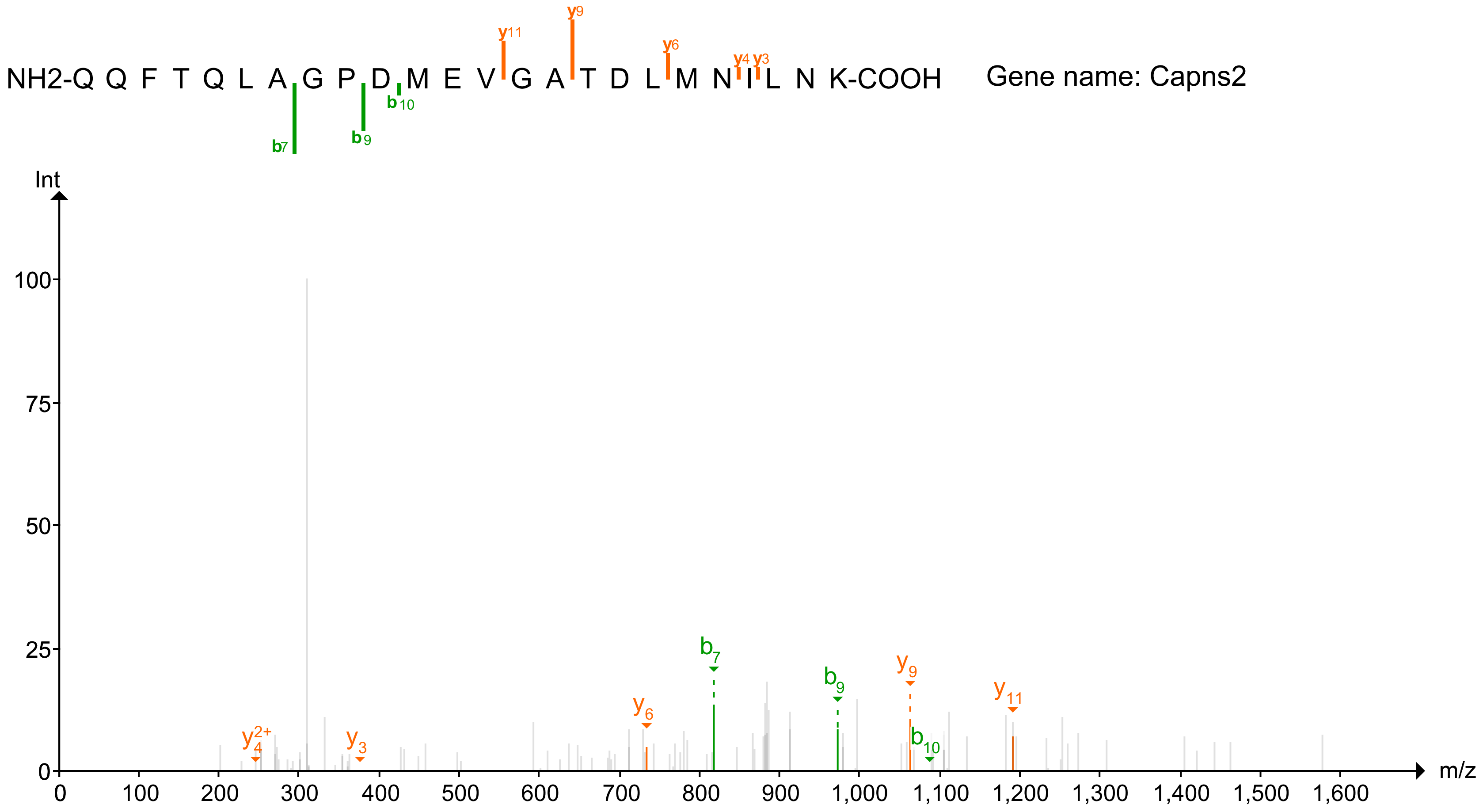
NH2-Q Q F T Q L A G P D M E V G A T D L M N I L N K-COOH
Gene name: Capns2
y11
y9
y6
y4
y3
b7
b9
b10
Int
100
75
50
25
0
y_4^{2+}
y_3
y_6
b_7
b_9
y_9
b_{10}
y_{11}
0
100
200
300
400
500
600
700
800
900
1,000
1,100
1,200
1,300
1,400
1,500
1,600
m/z

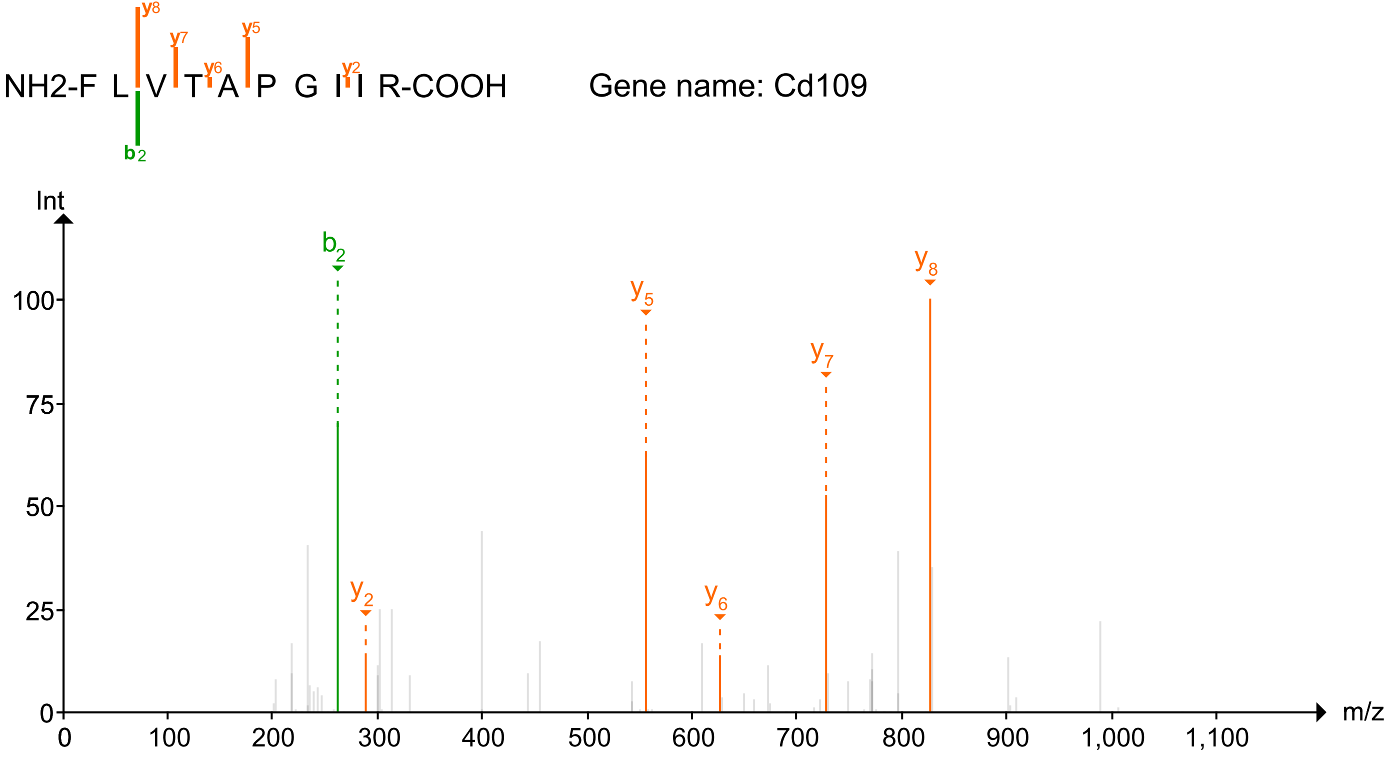

NH2-F L V T A P G I I R-COOH
Gene name: Cd109
y8
y7
y6
y5
y2
b2
Int
100
75
50
25
0
m/z
0
100
200
300
400
500
600
700
800
900
1,000
1,100
b2
y2
y5
y6
y7
y8

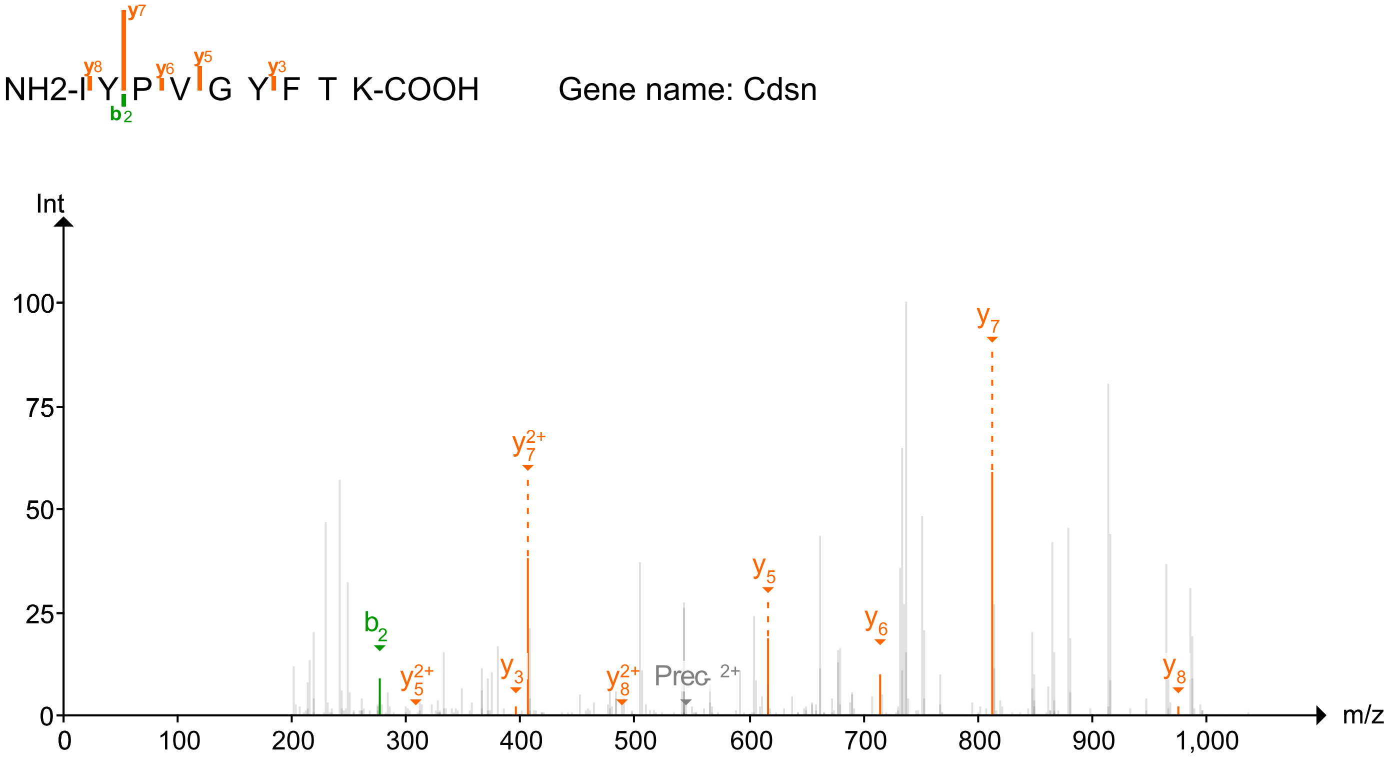

NH2-I Y P V G Y F T K-COOH
y8
y7
y6
y5
y3
b2
Gene name: Cdsn
Int
100
75
50
25
0
m/z
0
100
200
300
400
500
600
700
800
900
1,000
y7
y7 2+
y5
y6
b2
y5 2+
y3
y8 2+
Prec 2+
y8

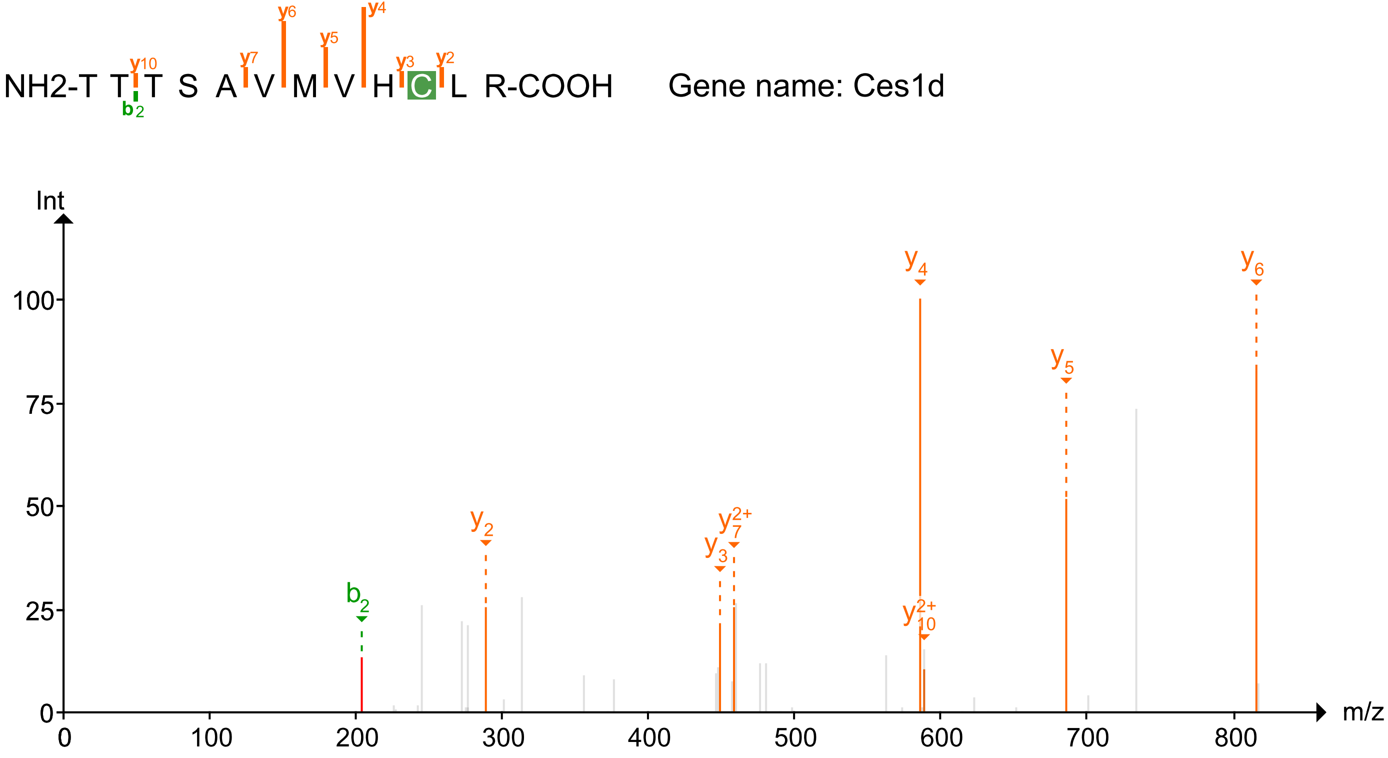

NH2-T T T S A V M V H C L R-COOH
Gene name: Ces1d
y10 y7 y6 y5 y4 y3 y2
b2
Int
100
75
50
25
0
m/z
0 100 200 300 400 500 600 700 800
y4 y6 y5 y2 y3 y7 2+ y10 2+ b2

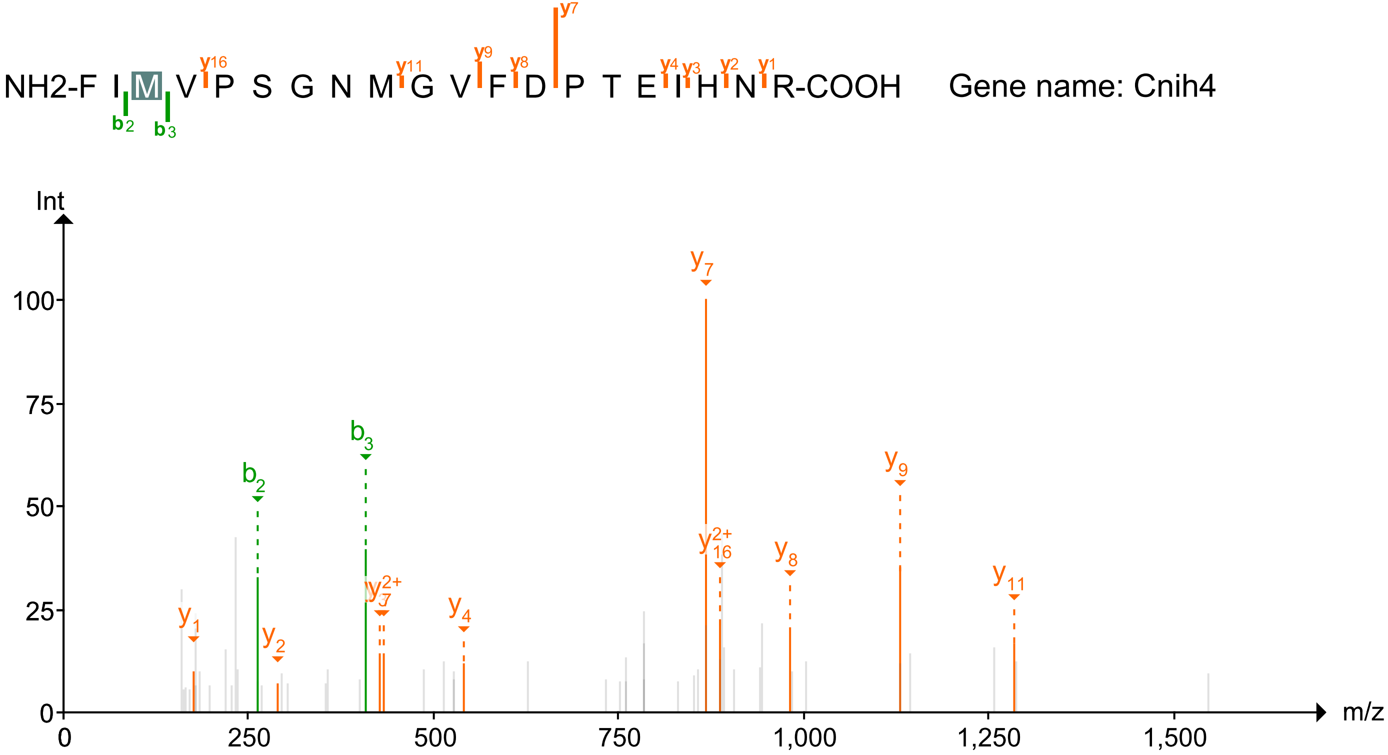
NH2-F I M V P S G N M G V F D P T E I H N R-COOH
Gene name: Cnih4
b2
b3
y16
y11
y9
y8
y7
y4
y3
y2
y1
Int
100
75
50
25
0
m/z
250
500
750
1,000
1,250
1,500

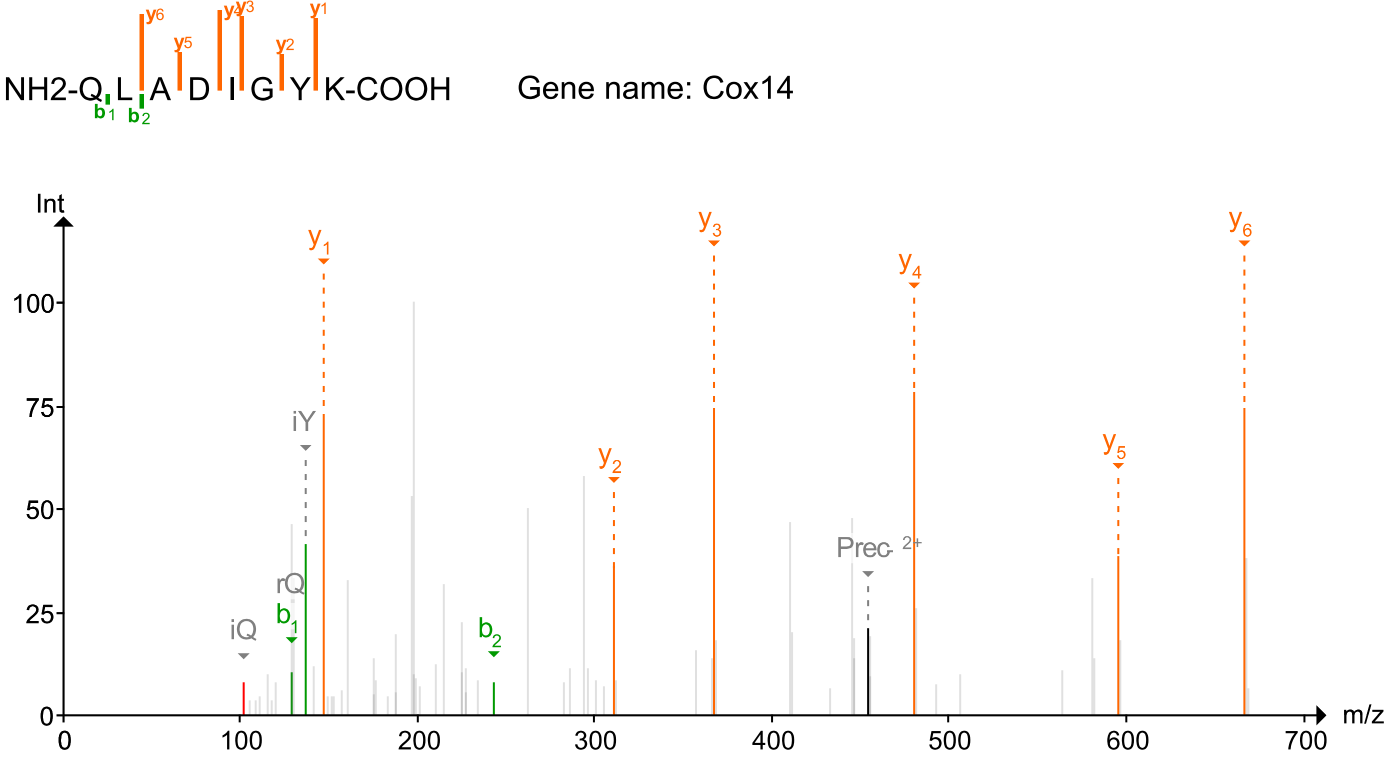
NH2-Q L A D I G Y K-COOH
Gene name: Cox14
b1 b2
y6 y5 y4 y3 y2 y1
Int
m/z
0
25
50
75
100
0
100
200
300
400
500
600
700
iQ
rQ
b1
iY
y1
b2
y2
y3
Prec 2+
y4
y5
y6

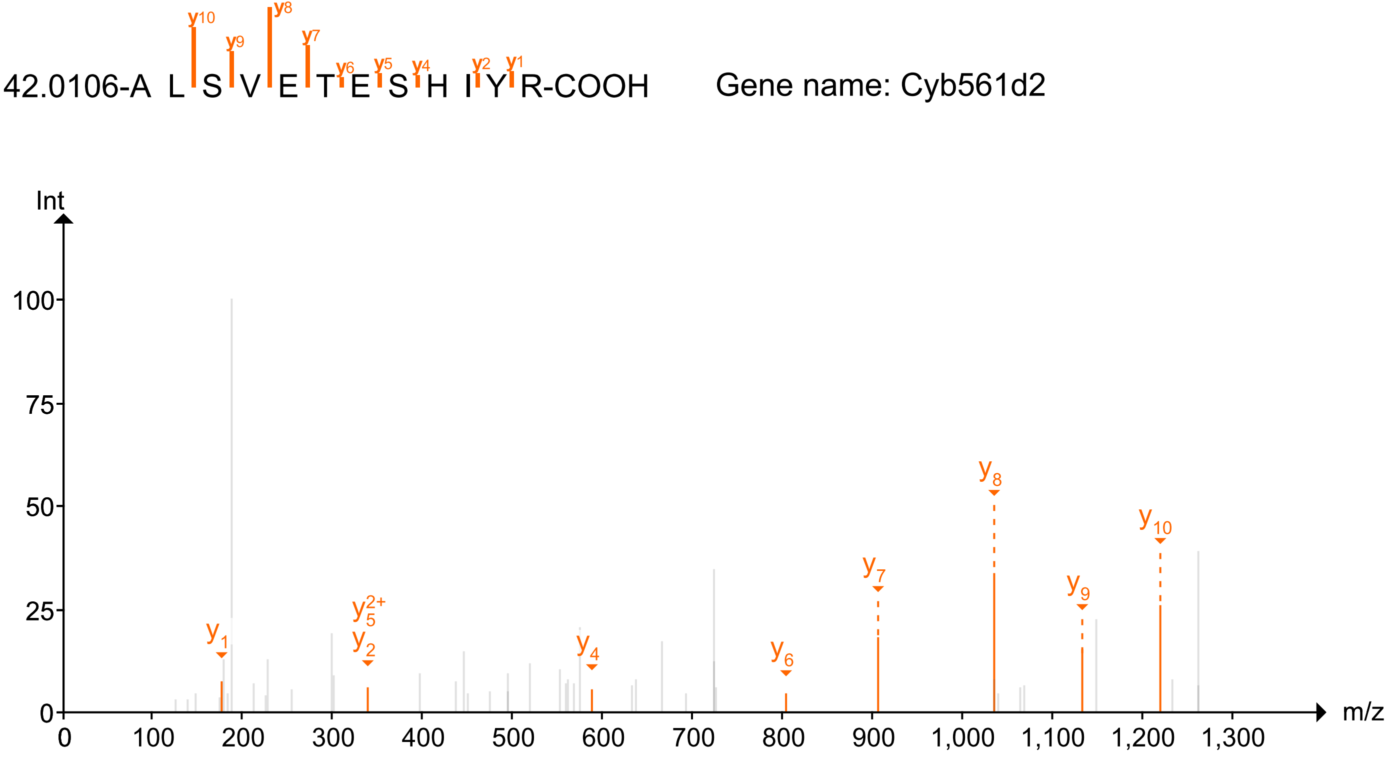
42.0106-A L S V E T E S H I Y R-COOH
Gene name: Cyb561d2
y10
y9
y8
y7
y6
y5
y4
y2
y1
Int
100
75
50
25
0
m/z
0
100
200
300
400
500
600
700
800
900
1,000
1,100
1,200
1,300
y1
y5 2+
y2
y4
y6
y7
y8
y9
y10

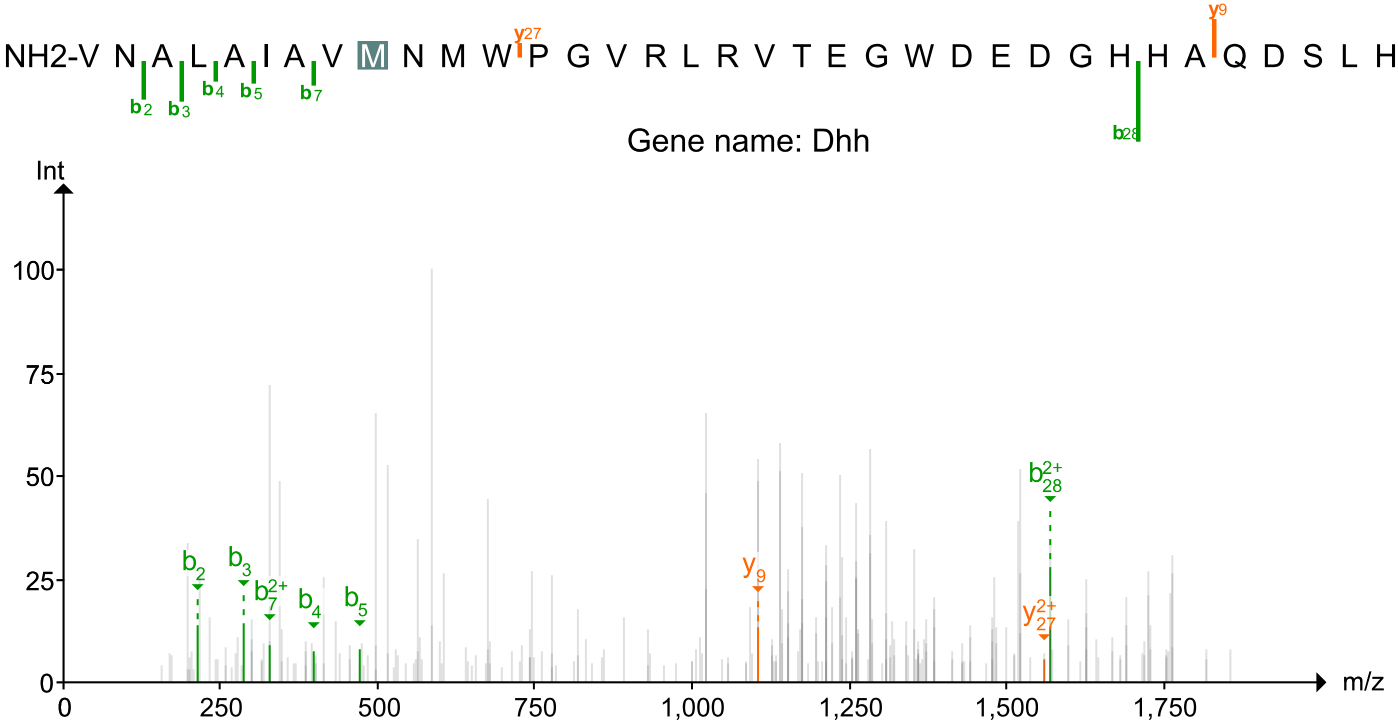

NH2-V N A L A I A V M N M W P G V R L R V T E G W D E D G H H A Q D S L H
b2 b3 b4 b5 b7 b28
y27 y9
Gene name: Dhh
Int
m/z
0
25
50
75
100
250
500
750
1,000
1,250
1,500
1,750
b2 b3 b7 2+ b4 b5 y9 b28 2+ y27 2+

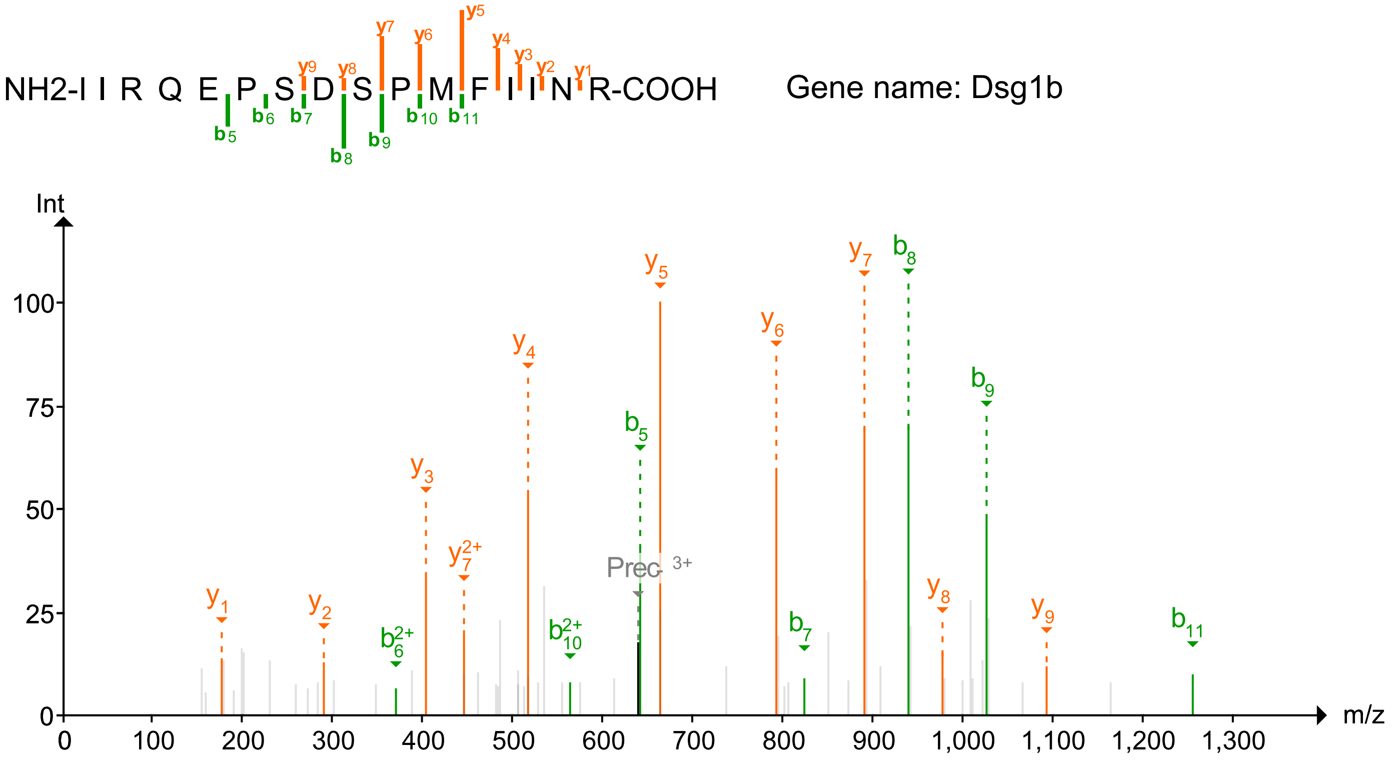

NH2-I I R Q E P S D S P M F I I N R-COOH
Gene name: Dsg1b
Int
m/z
Prec 3+

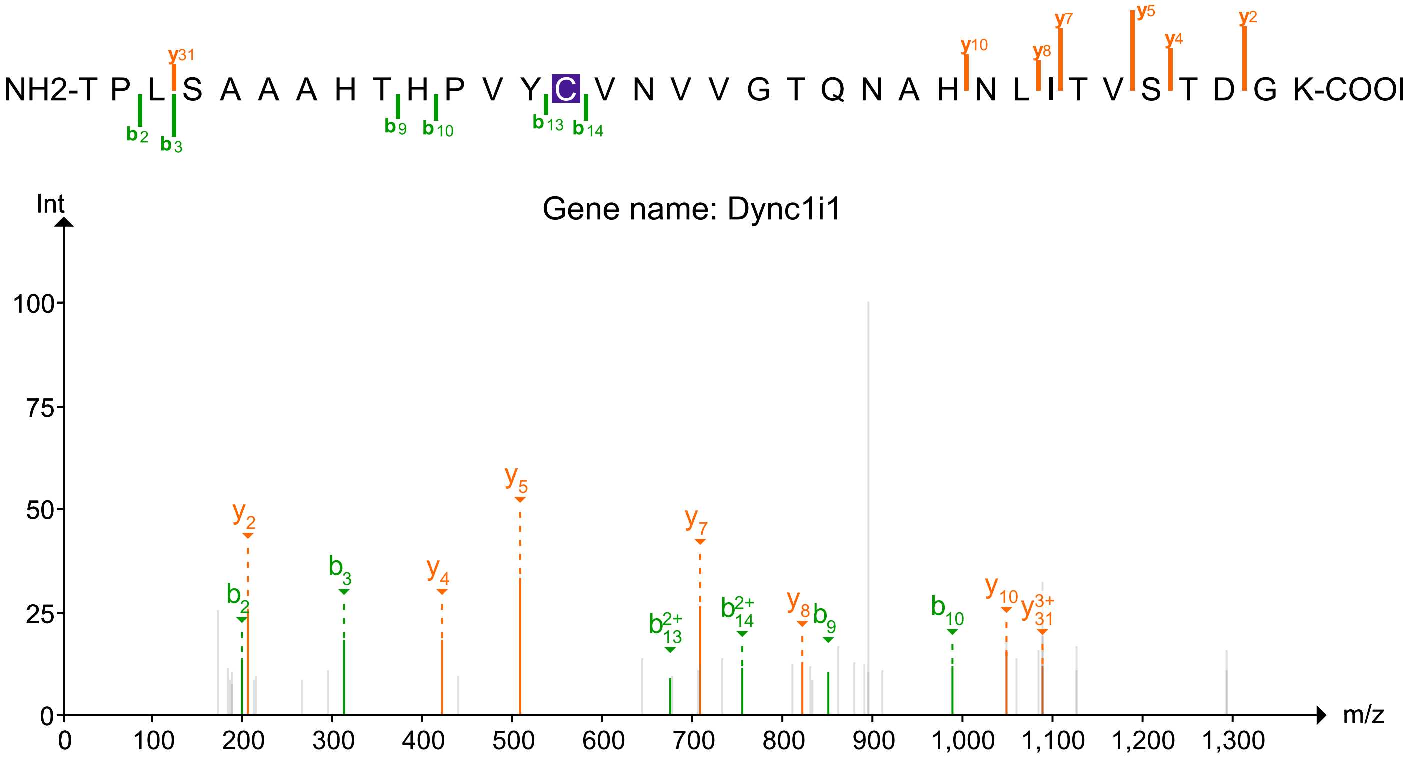

NH2-T P L S A A A H T H P V Y C V N V V G T Q N A H N L I T V S T D G K-COOH
y31 y10 y8 y7 y5 y4 y2
b2 b3 b9 b10 b13 b14
Gene name: Dync1i1
Int
m/z
0 25 50 75 100
0 100 200 300 400 500 600 700 800 900 1,000 1,100 1,200 1,300
y2 b2 b3 y4 y5 b13 2+ y7 b14 2+ y8 b9 b10 y10 y31 3+

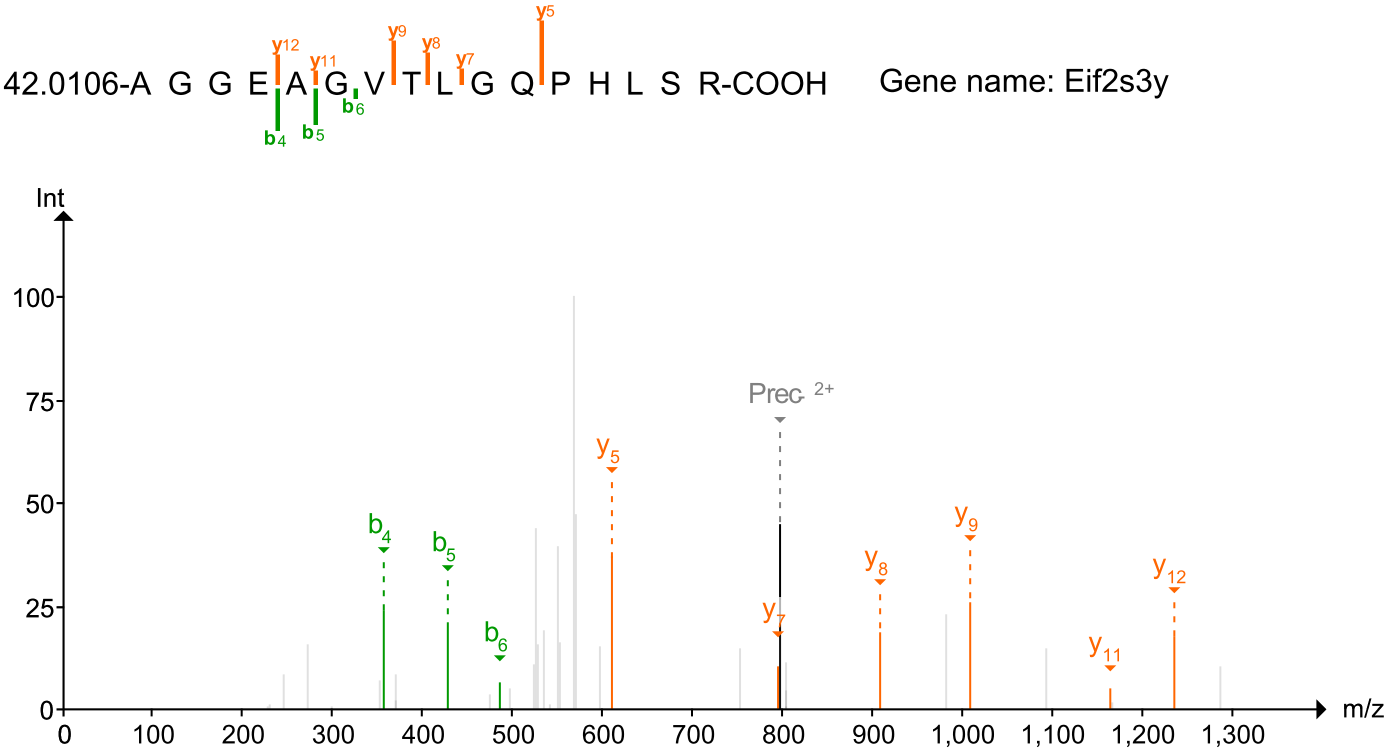

42.0106-A G G E A G V T L G Q P H L S R-COOH
Gene name: Eif2s3y
y12
y11
y9
y8
y7
y5
b4
b5
b6
Int
100
75
50
25
0
Prec 2+
m/z
0
100
200
300
400
500
600
700
800
900
1,000
1,100
1,200
1,300

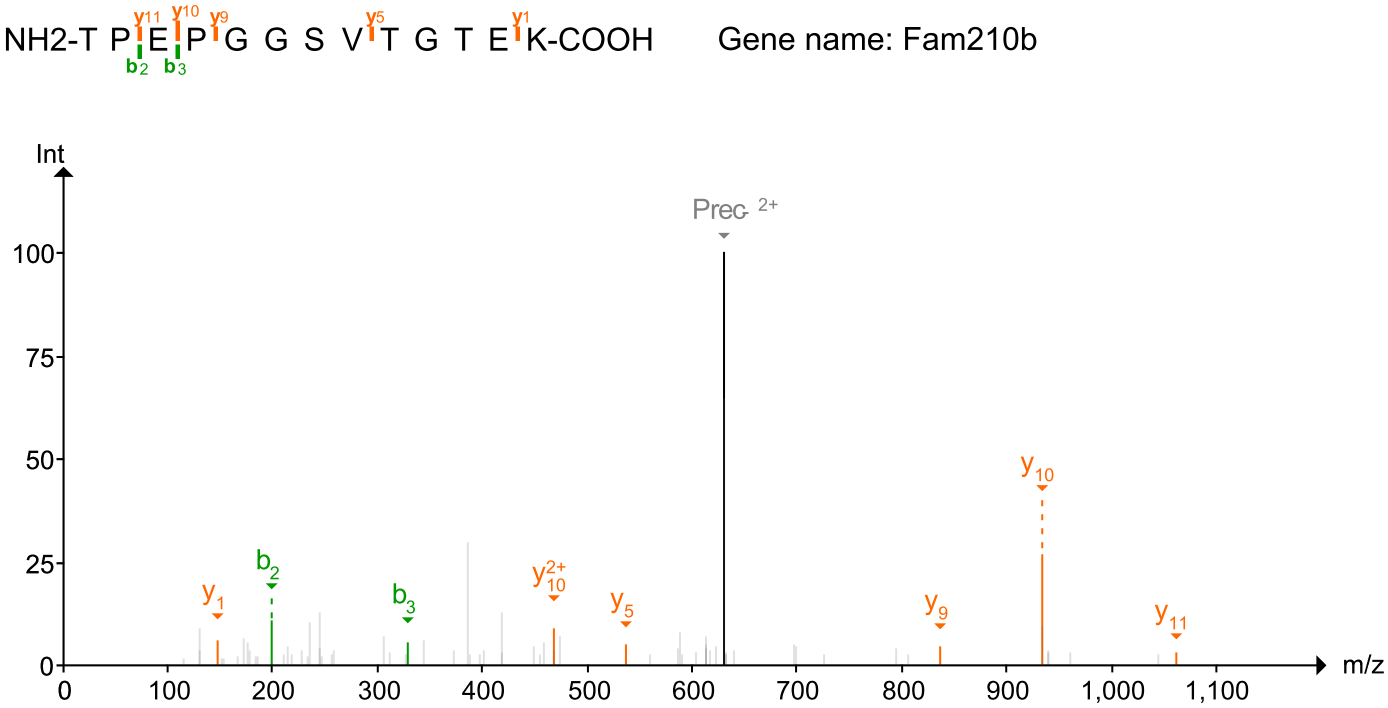

NH2-T P E P G G S V T G T E K-COOH
y11
y10
y9
y5
y1
b2
b3
Gene name: Fam210b
Int
Prec 2+
100
75
50
25
0
y10
y2+10
y1
b2
b3
y5
y9
y11
0
100
200
300
400
500
600
700
800
900
1,000
1,100
m/z

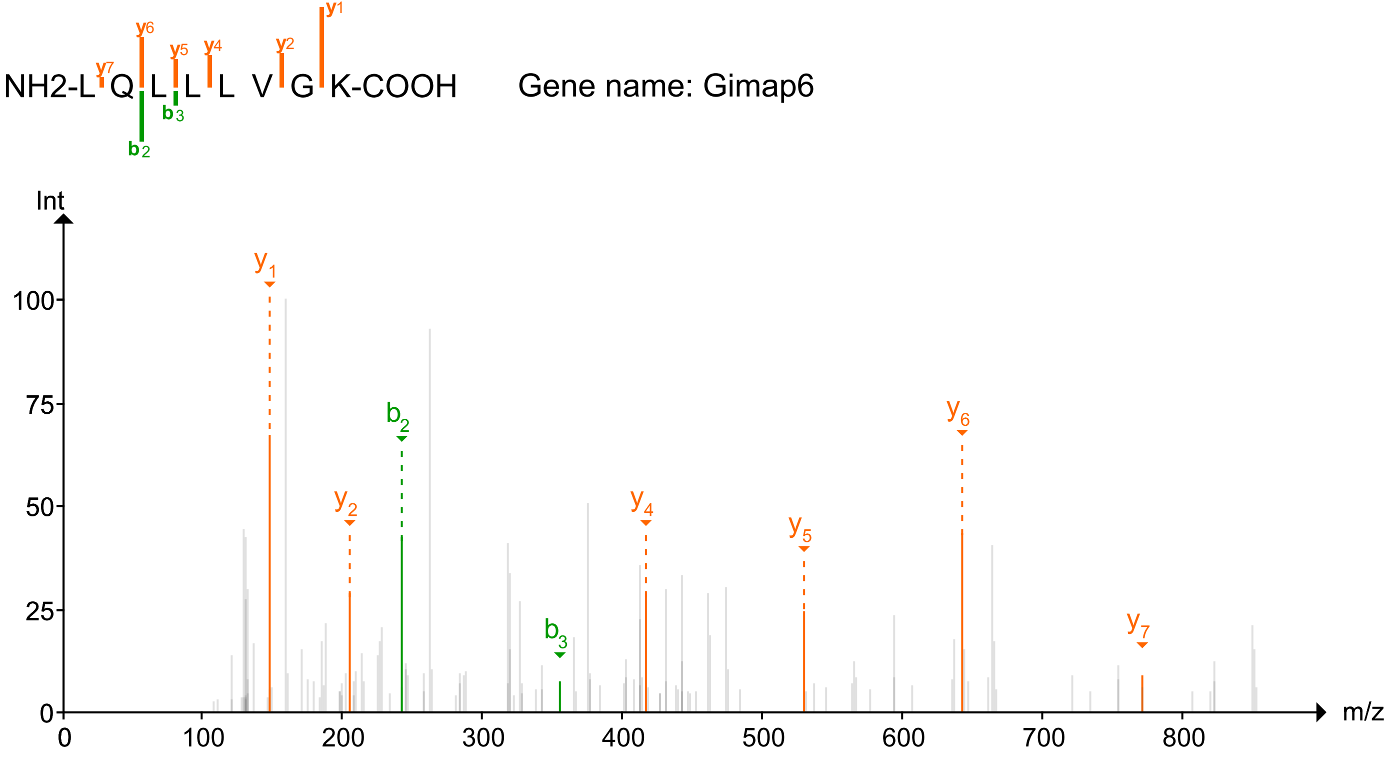

NH2-L Q L L L V G K-COOH
Gene name: Gimap6
y7 y6 y5 y4 y2 y1
b2 b3
Int
100
75
50
25
0
m/z
0 100 200 300 400 500 600 700 800

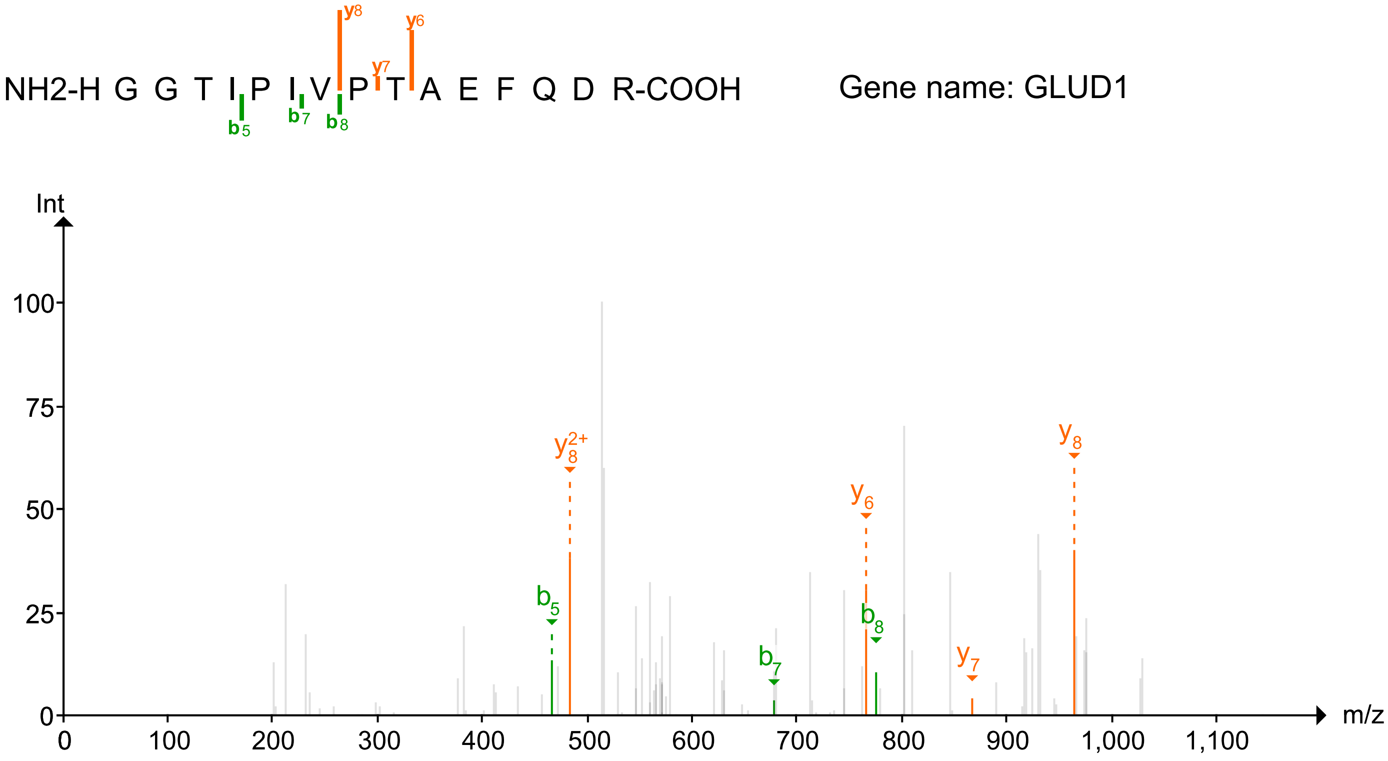

NH2-H G G T I P I V P T A E F Q D R-COOH
Gene name: GLUD1
y8
y7
y6
b5
b7
b8
Int
100
75
50
25
0
m/z
0
100
200
300
400
500
600
700
800
900
1,000
1,100
y_8^{2+}
b_5
b_7
y_6
b_8
y_7
y_8

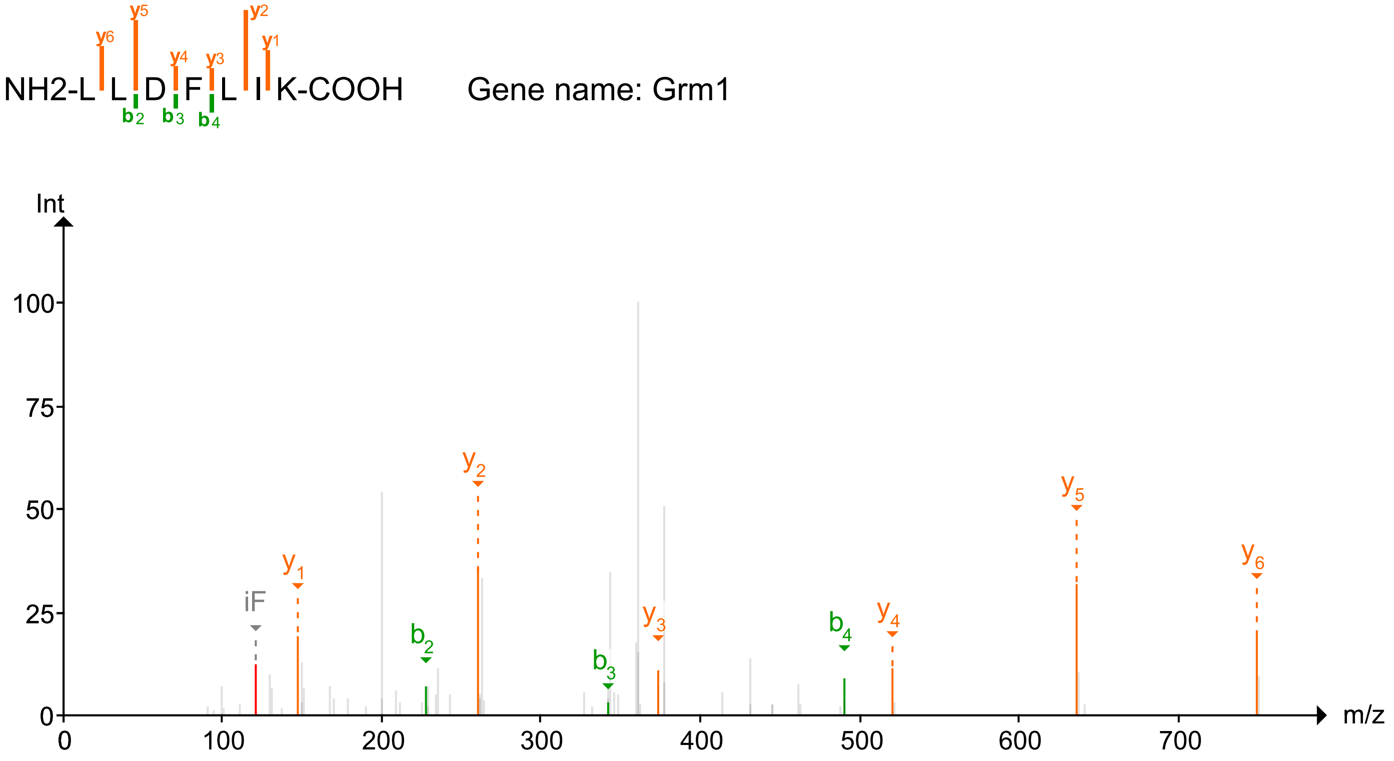

NH2-L L D F L I K-COOH
y6 y5 y4 y3 y2 y1
b2 b3 b4
Gene name: Grm1
Int
100
75
50
25
0
iF
y1
b2
y2
b3
y3
b4
y4
y5
y6
0
100
200
300
400
500
600
700
m/z

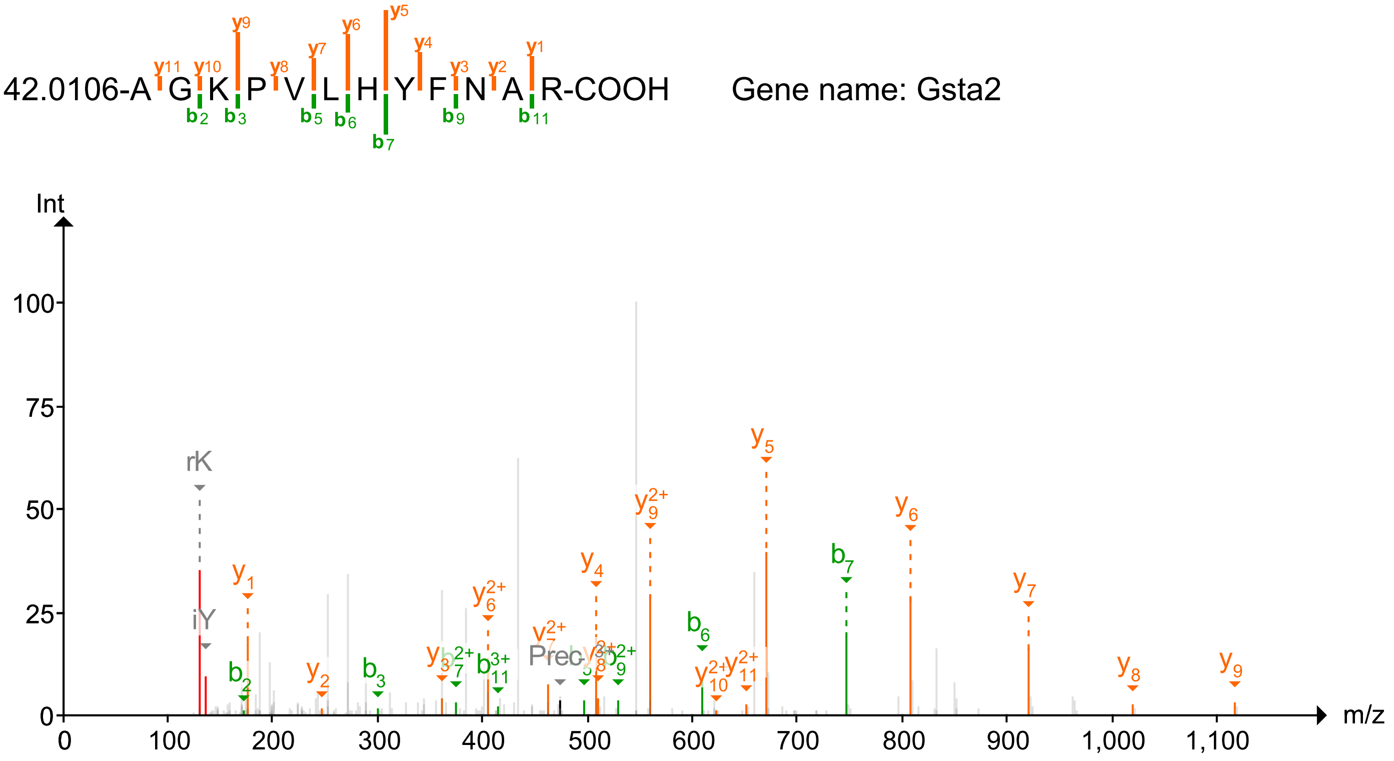

42.0106-A G K P V L H Y F N A R-COOH
Gene name: Gsta2
Int
m/z
0
25
50
75
100
100
200
300
400
500
600
700
800
900
1,000
1,100
rK
iY
Prec

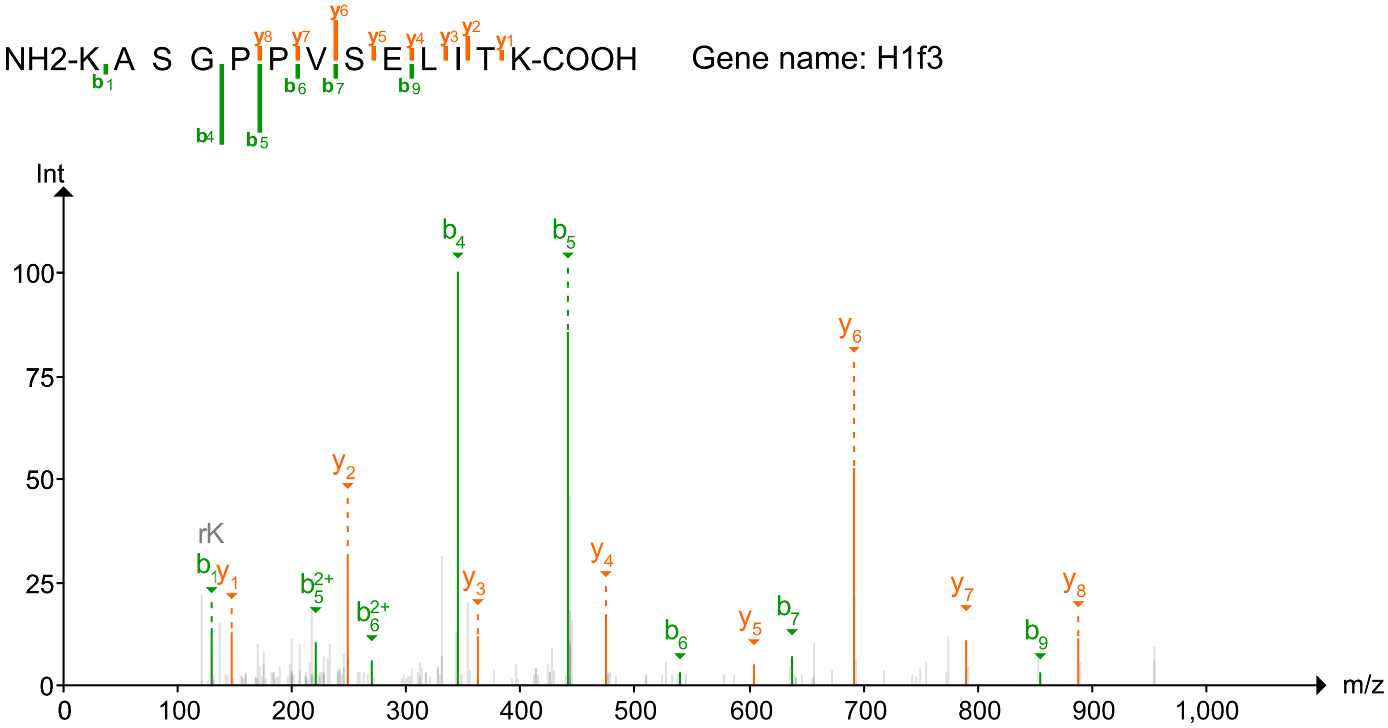
NH2-K A S G P P V S E L I T K-COOH
Gene name: H1f3
y8 y7 y6 y5 y4 y3 y2 y1
b1 b4 b5 b6 b7 b9
Int
100
75
50
25
0
b4
b5
y6
y2
rK
b1
y1
b5 2+
b6 2+
y3
y4
b6
y5
b7
y7
b9
y8
0 100 200 300 400 500 600 700 800 900 1,000
m/z

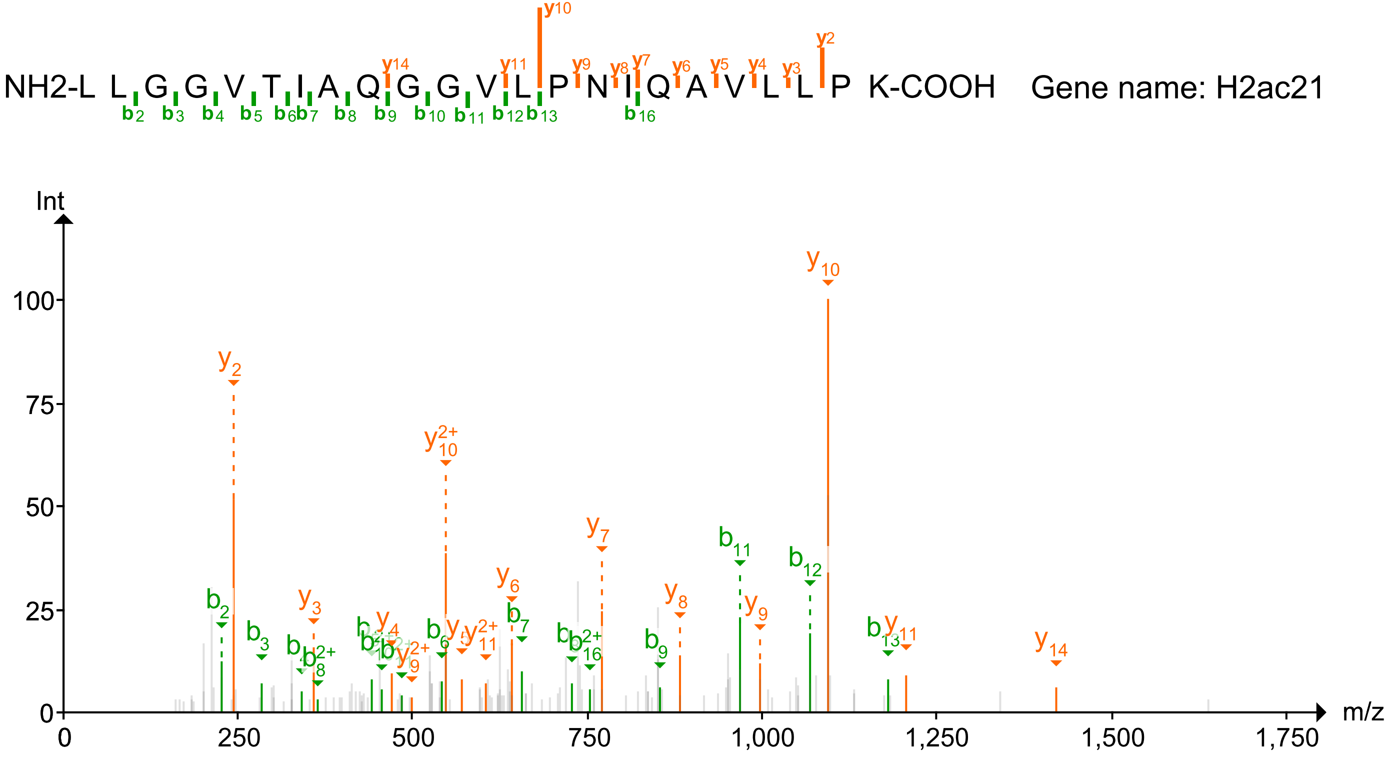
NH2-L L G G V T I A Q G G V L P N I Q A V L L P K-COOH
Gene name: H2ac21
Int
m/z
0
25
50
75
100
250
500
750
1,000
1,250
1,500
1,750
y10
y2
y2+10
y7
b11
b12
y6
y3
b2
y8
y9
b7
b3
y4
b6
y2+11
b2+16
b9
y11
y14
b13
b2+8

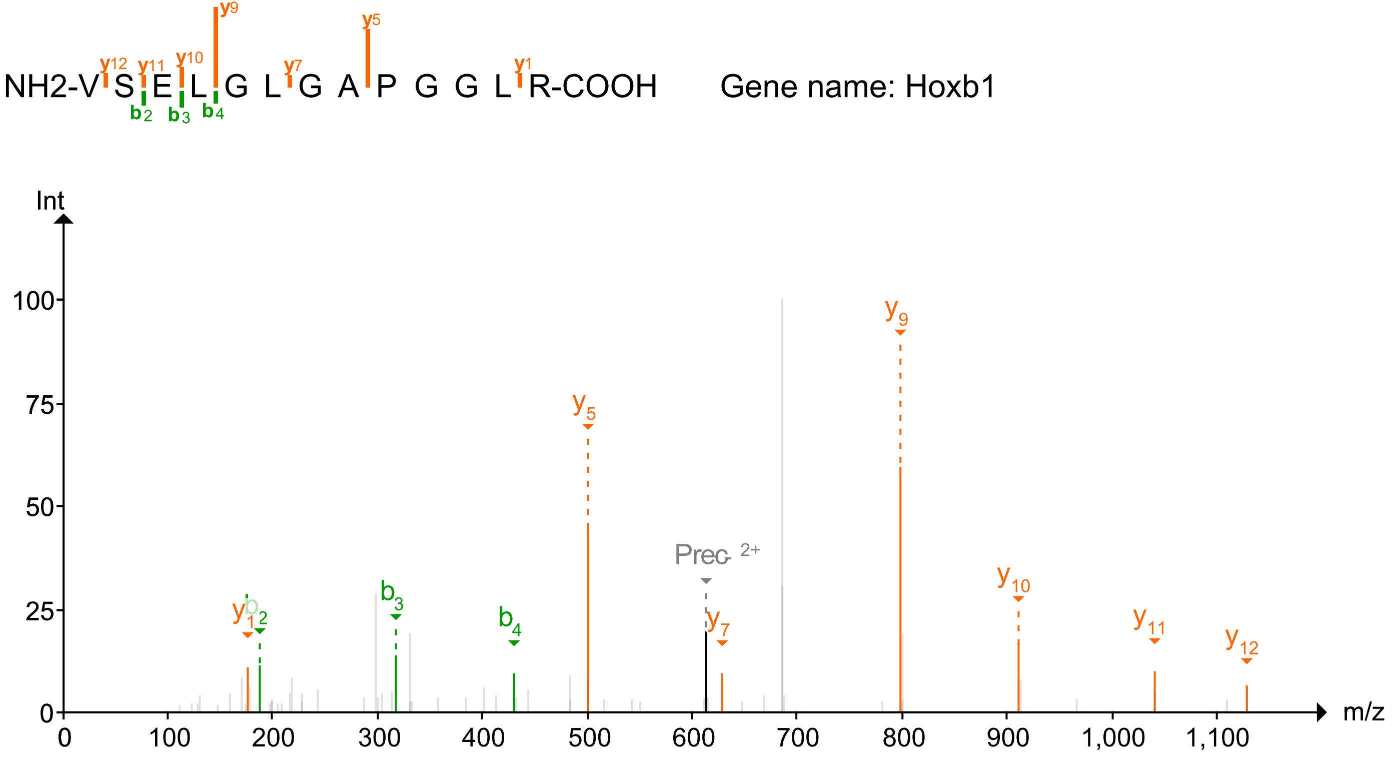
NH2-V S E L G L G A P G G L R-COOH
Gene name: Hoxb1
y12 y11 y10 y9 y7 y5 y1
b2 b3 b4
Int
m/z
Prec 2+
0
25
50
75
100
0
100
200
300
400
500
600
700
800
900
1,000
1,100

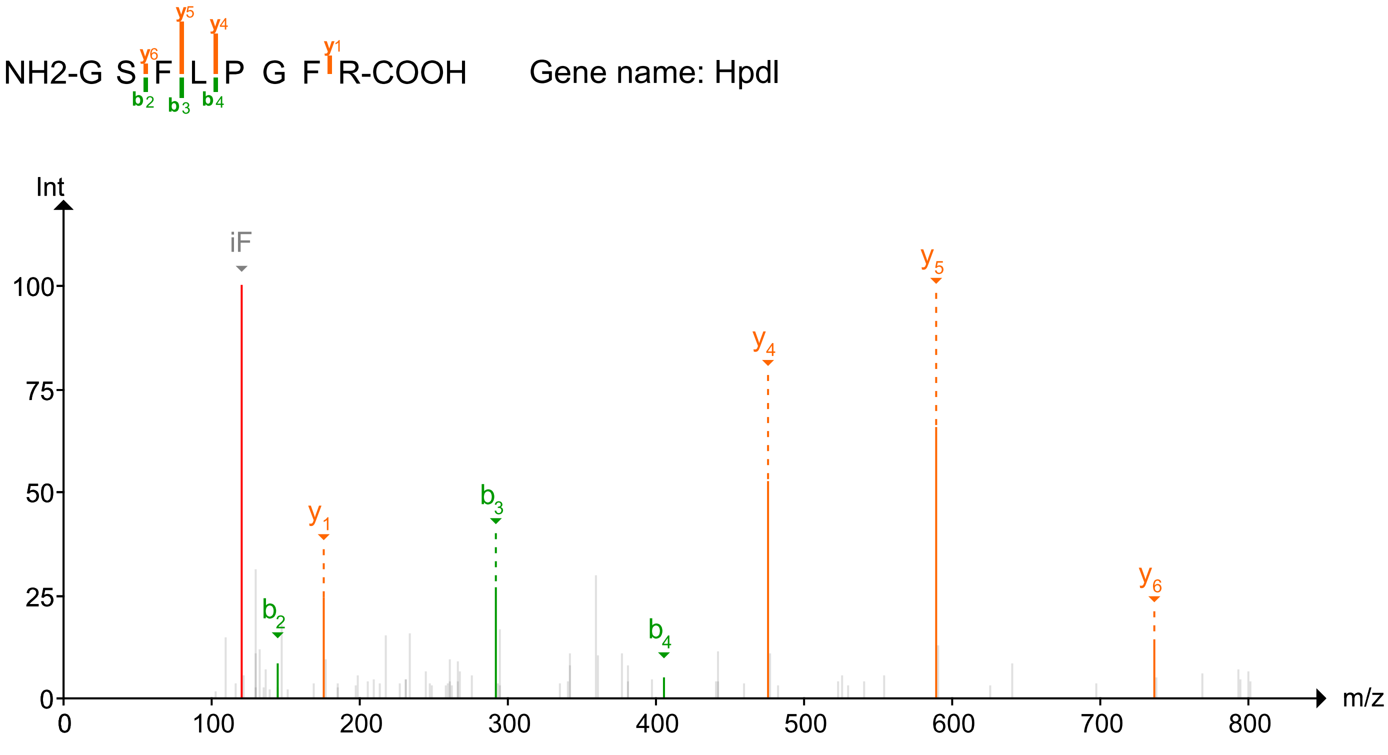

NH2-G S F L P G F R-COOH
y6
y5
y4
y1
b2
b3
b4
Gene name: Hpdl
Int
iF
y5
y4
b3
y1
b2
b4
y6
100
75
50
25
0
0
100
200
300
400
500
600
700
800
m/z

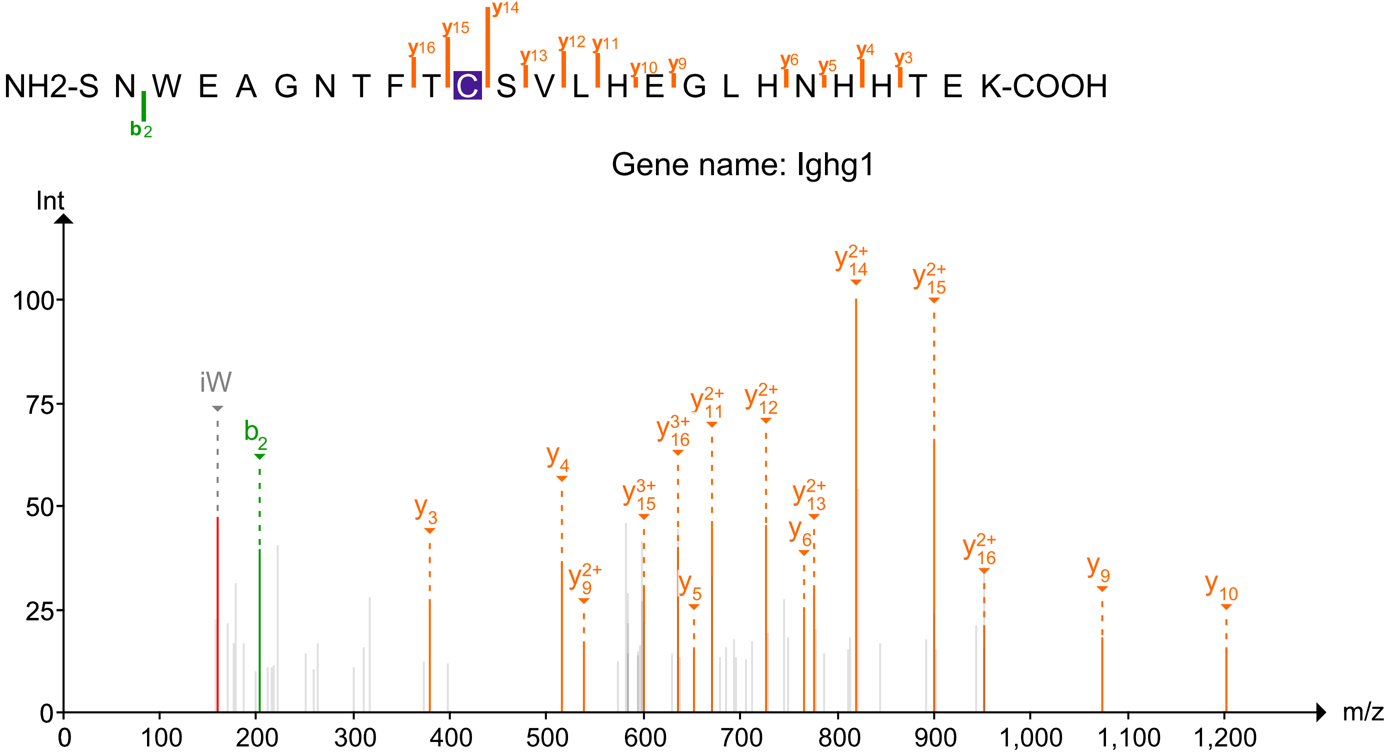

NH2-S N W E A G N T F T C S V L H E G L H N H H T E K-COOH
b2
y16 y15 y14 y13 y12 y11 y10 y9 y6 y5 y4 y3
Gene name: Ighg1
Int
m/z
iW
b2
y3
y4
y9 2+
y15 3+
y16 3+
y5
y11 2+
y12 2+
y6
y13 2+
y14 2+
y15 2+
y16 2+
y9
y10
0
25
50
75
100
0
100
200
300
400
500
600
700
800
900
1,000
1,100
1,200

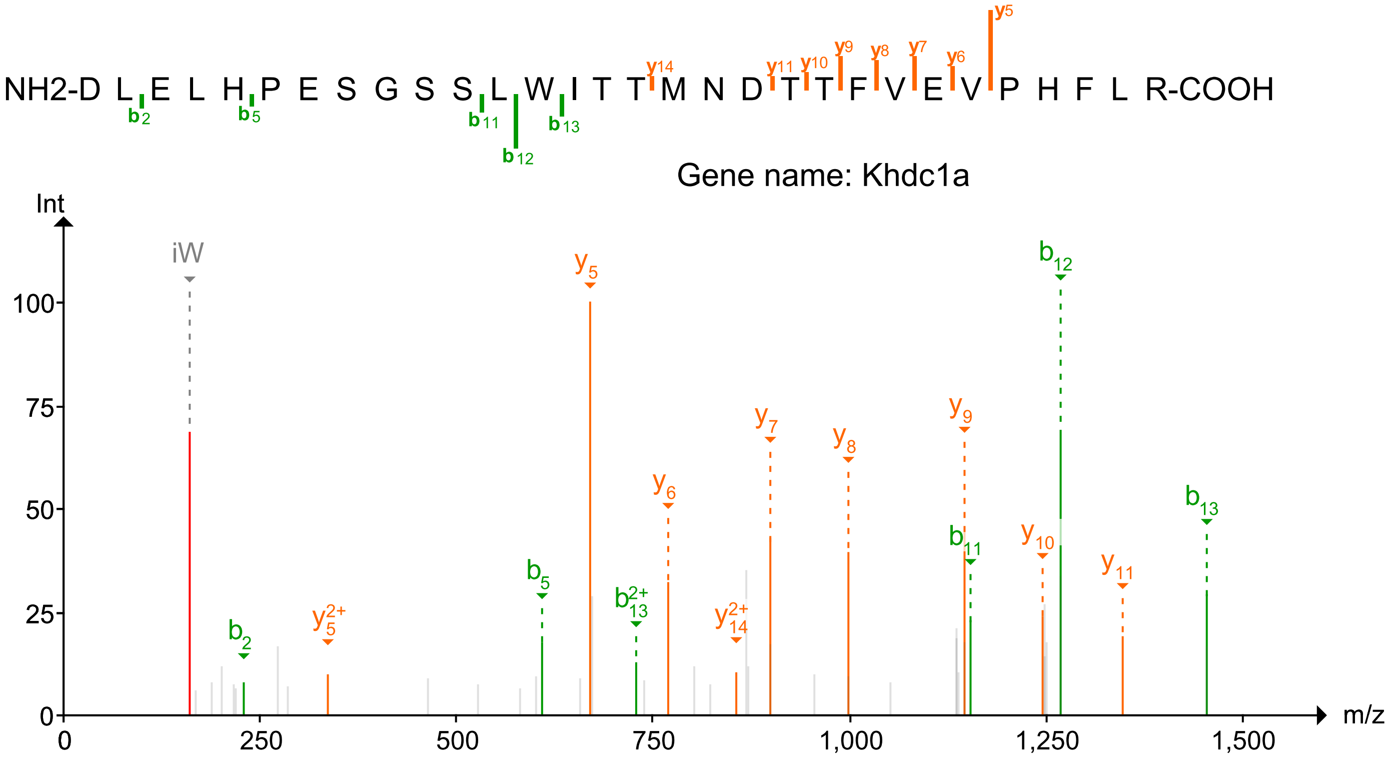

NH2-D L E L H P E S G S S L W I T T M N D T T F V E V P H F L R-COOH
b2 b5 b11 b12 b13
y14 y11 y10 y9 y8 y7 y6 y5
Gene name: Khdc1a
Int
iW
y5 y6 y7 y8 y9 y10 y11
y_5^{2+} y_{14}^{2+}
b2 b5 b11 b12 b13 b_{13}^{2+}
0 25 50 75 100
0 250 500 750 1,000 1,250 1,500
m/z

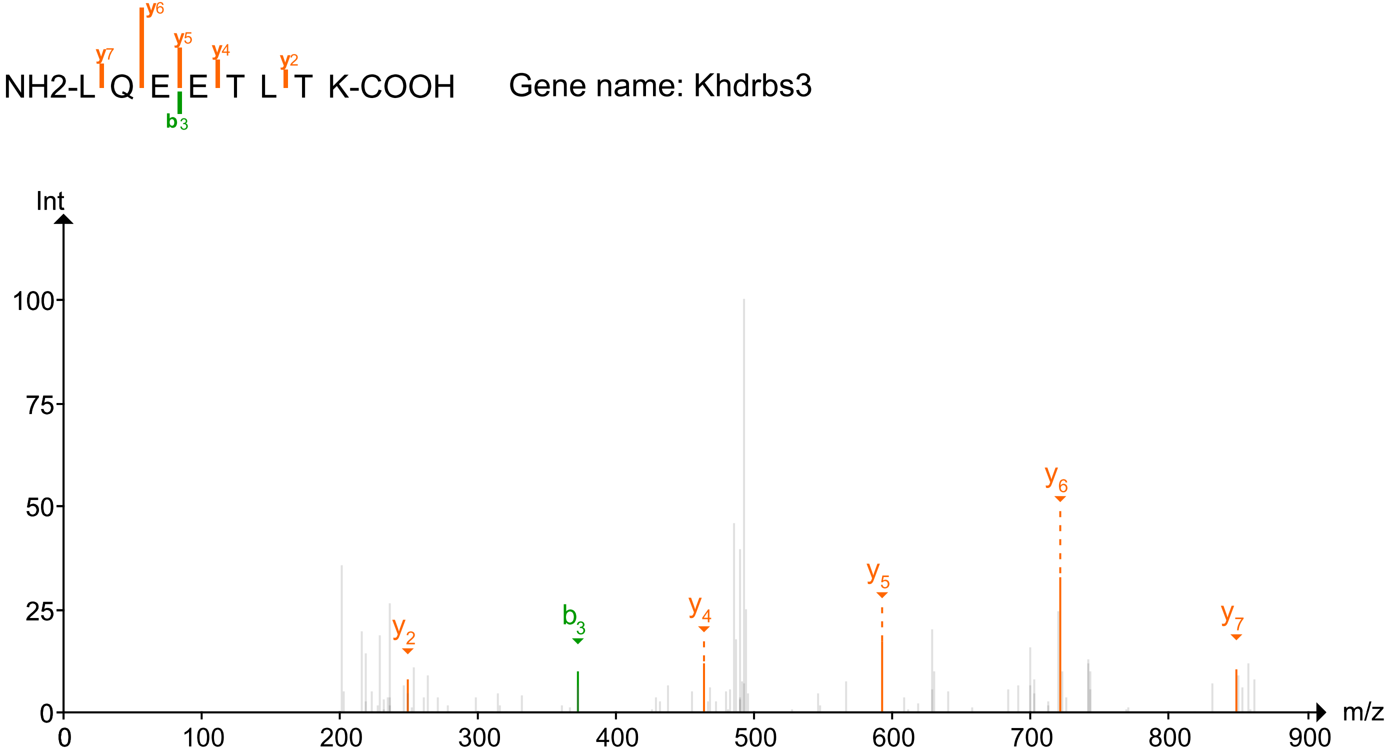

NH2-L Q E E T L T K-COOH
y7
y6
y5
y4
y2
b3
Gene name: Khdrbs3
Int
100
75
50
25
0
m/z
0
100
200
300
400
500
600
700
800
900
y2
b3
y4
y5
y6
y7

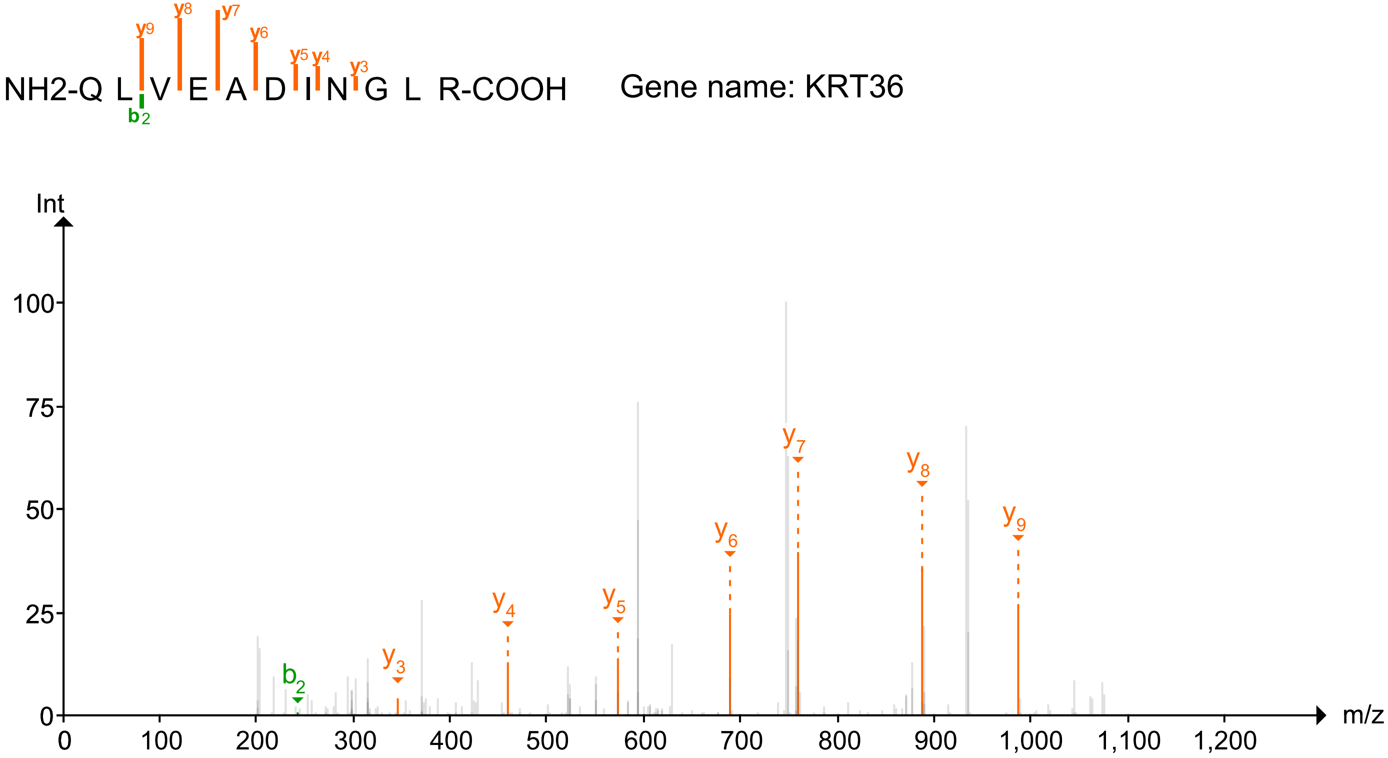

NH2-Q L V E A D I N G L R-COOH
Gene name: KRT36
y9 y8 y7 y6 y5 y4 y3
b2
Int
100
75
50
25
0
m/z
0 100 200 300 400 500 600 700 800 900 1,000 1,100 1,200
b2 y3 y4 y5 y6 y7 y8 y9

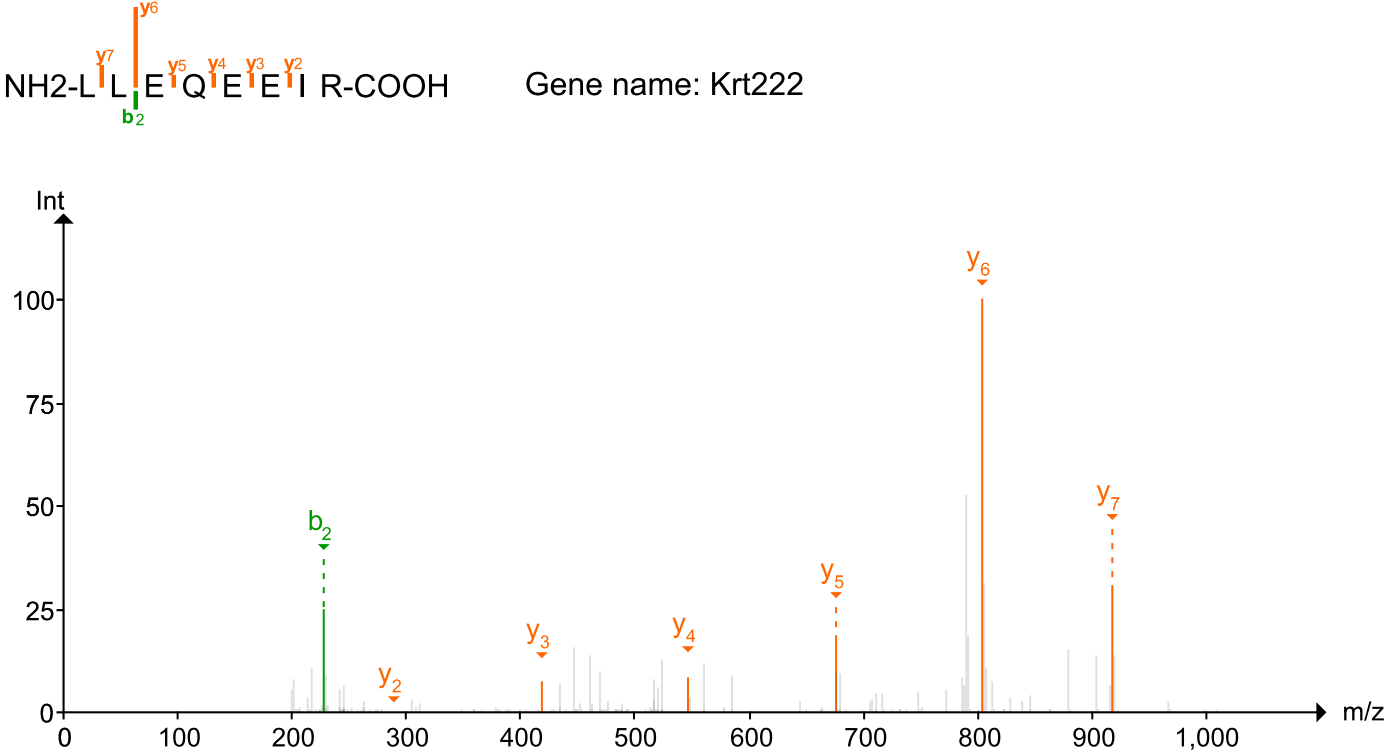
y6
y7
y5
y4
y3
y2
NH2-L L E Q E E I R-COOH
b2
Gene name: Krt222
Int
100
75
50
25
0
y6
y7
b2
y5
y3
y4
y2
0
100
200
300
400
500
600
700
800
900
1,000
m/z

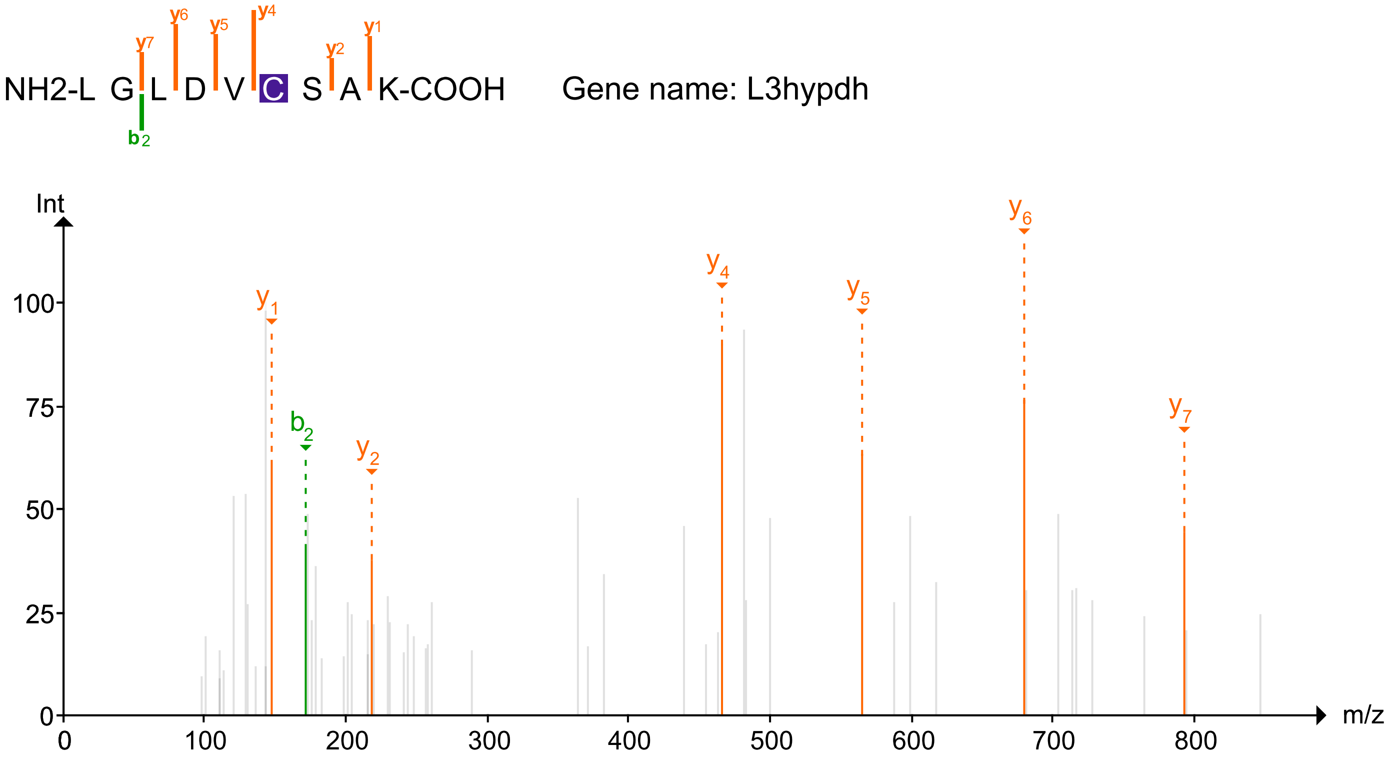

NH2-L G L D V C S A K-COOH
Gene name: L3hypdh
y7
y6
y5
y4
y2
y1
b2
Int
100
75
50
25
0
m/z
0
100
200
300
400
500
600
700
800
y1
b2
y2
y4
y5
y6
y7

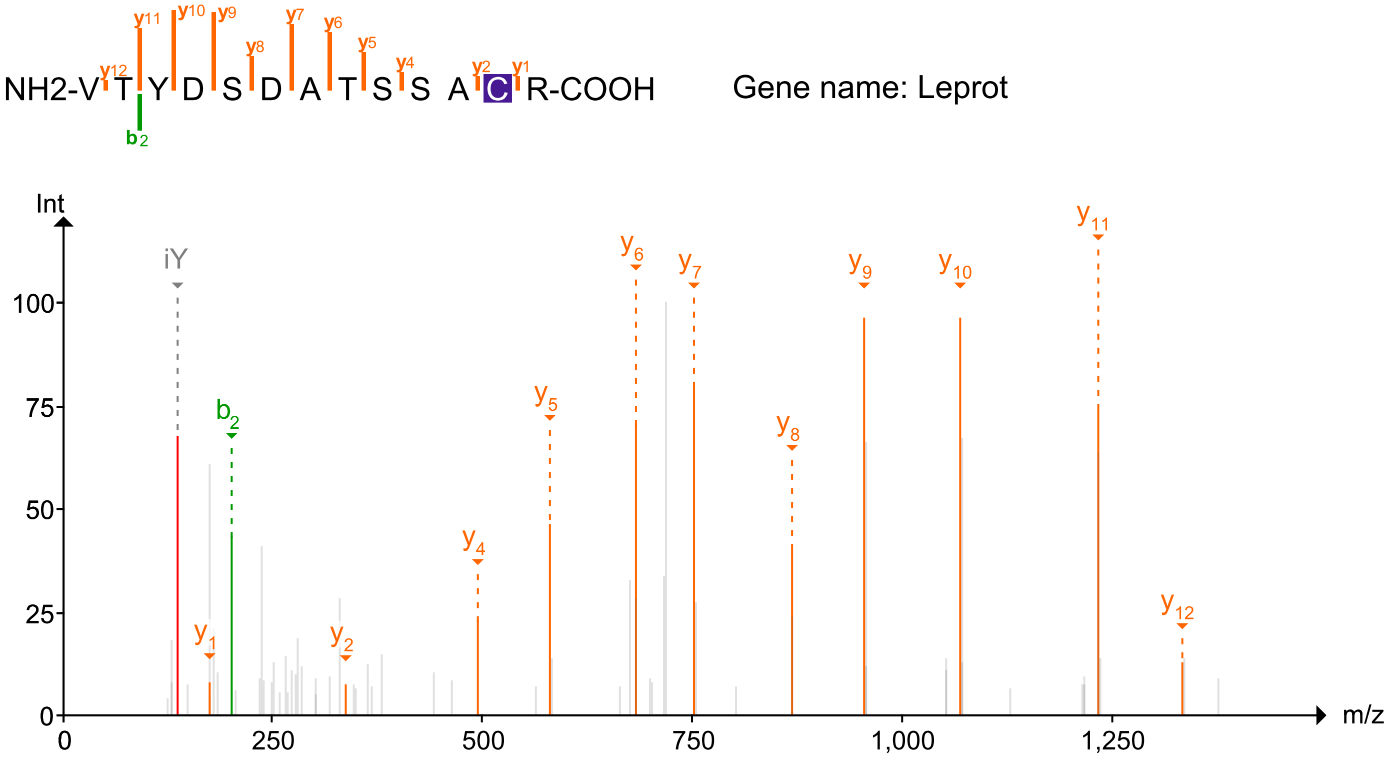
NH2-V T Y D S D A T S S A C R-COOH
Gene name: Leprot
y12 y11 y10 y9 y8 y7 y6 y5 y4 y2 y1
b2
Int
m/z
iY
100
75
50
25
0
0
250
500
750
1,000
1,250

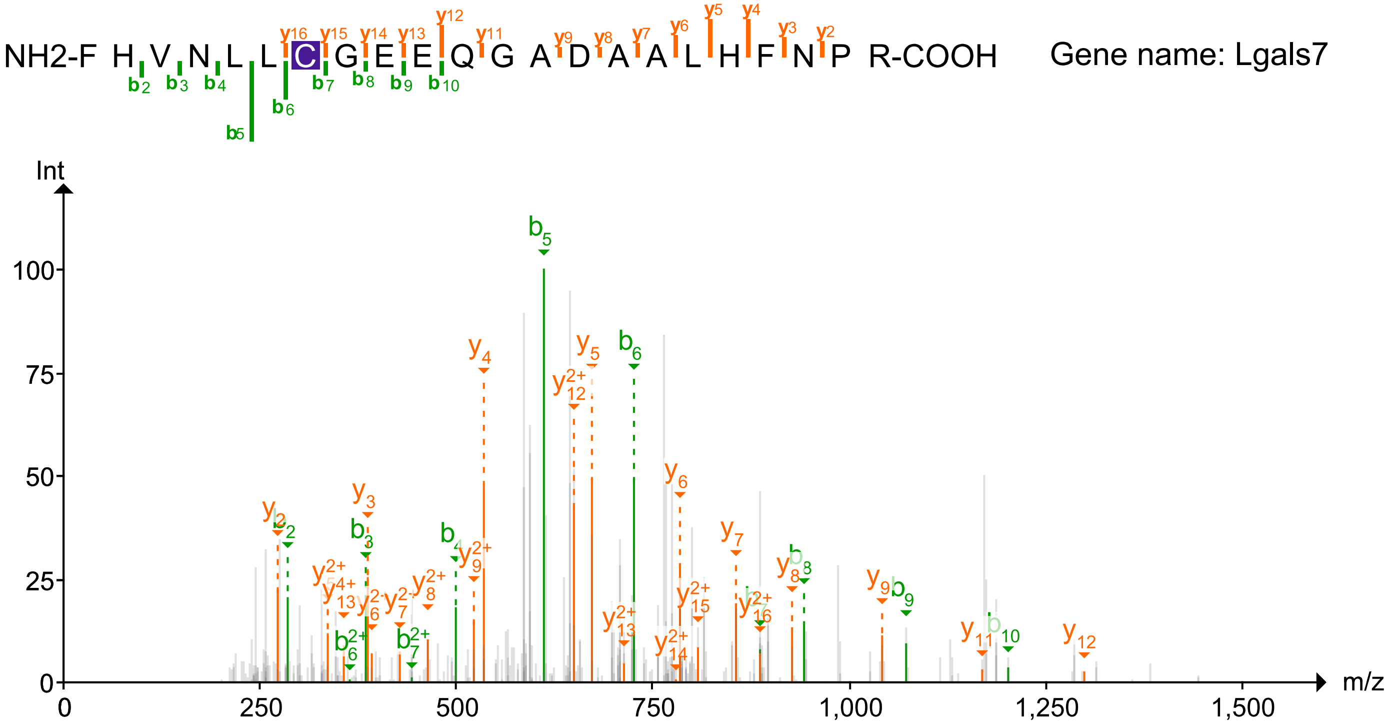

NH2-F H V N L L C G E E Q G A D A A L H F N P R-COOH
Gene name: Lgals7
Int
m/z
0
25
50
75
100
0
250
500
750
1,000
1,250
1,500

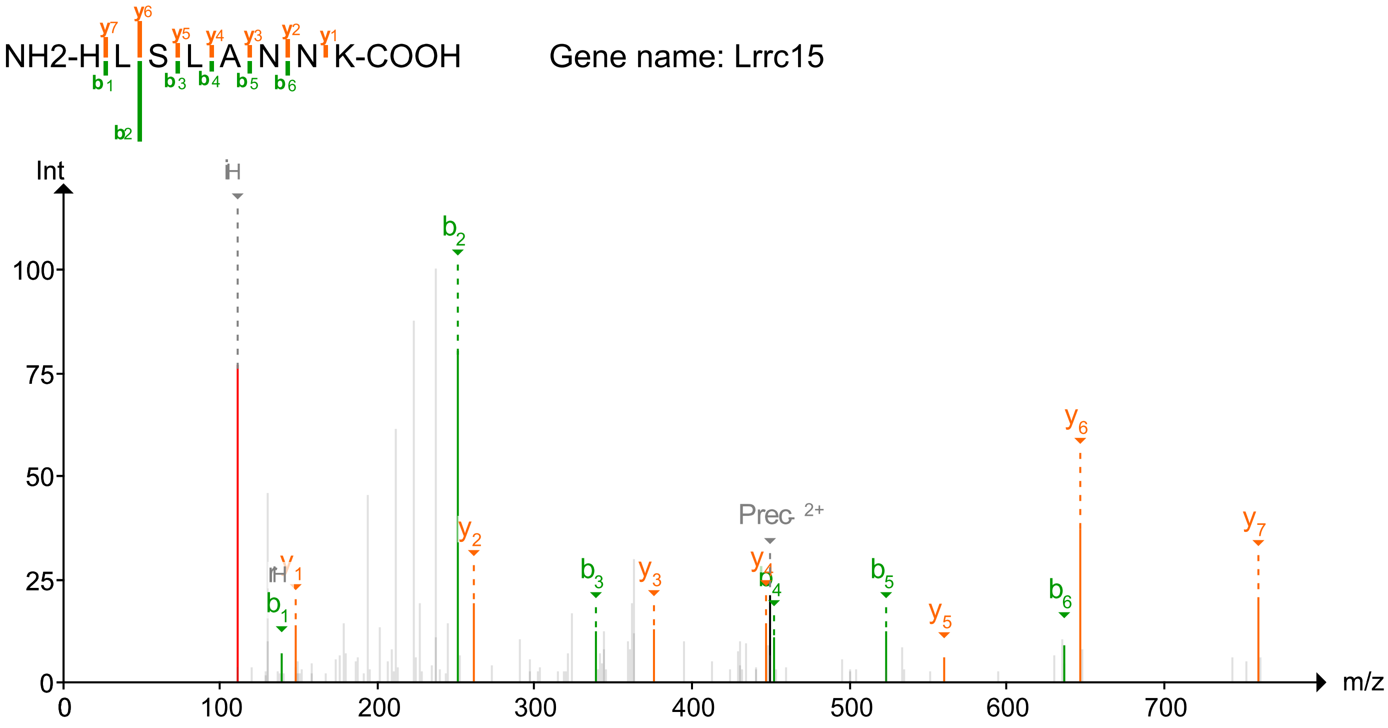

NH2-H L S L A N N K-COOH
y7 y6 y5 y4 y3 y2 y1
b1 b2 b3 b4 b5 b6
Gene name: Lrrc15
Int
m/z
100
75
50
25
0
0 100 200 300 400 500 600 700
iH
Prec 2+

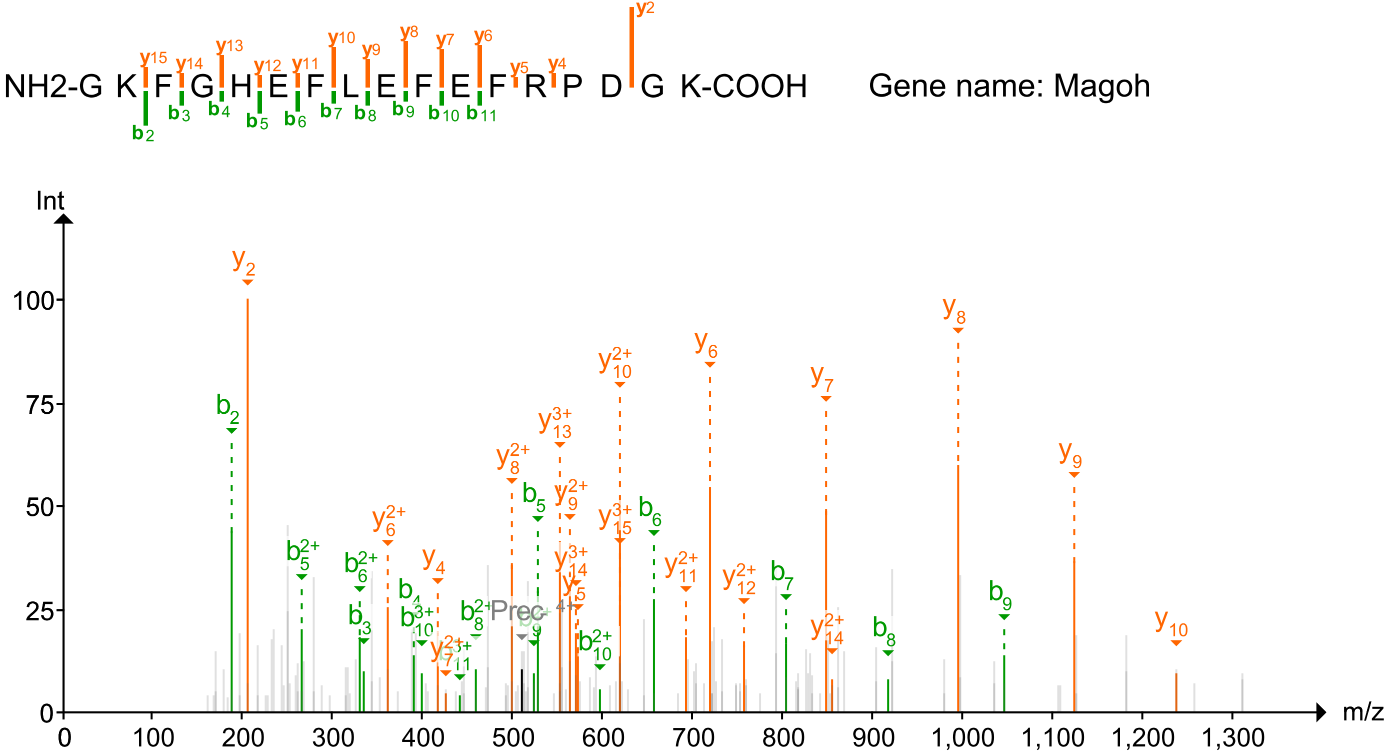
NH2-G K F G H E F L E F E F R P D G K-COOH
Gene name: Magoh
Int
m/z
0
25
50
75
100
0
100
200
300
400
500
600
700
800
900
1,000
1,100
1,200
1,300

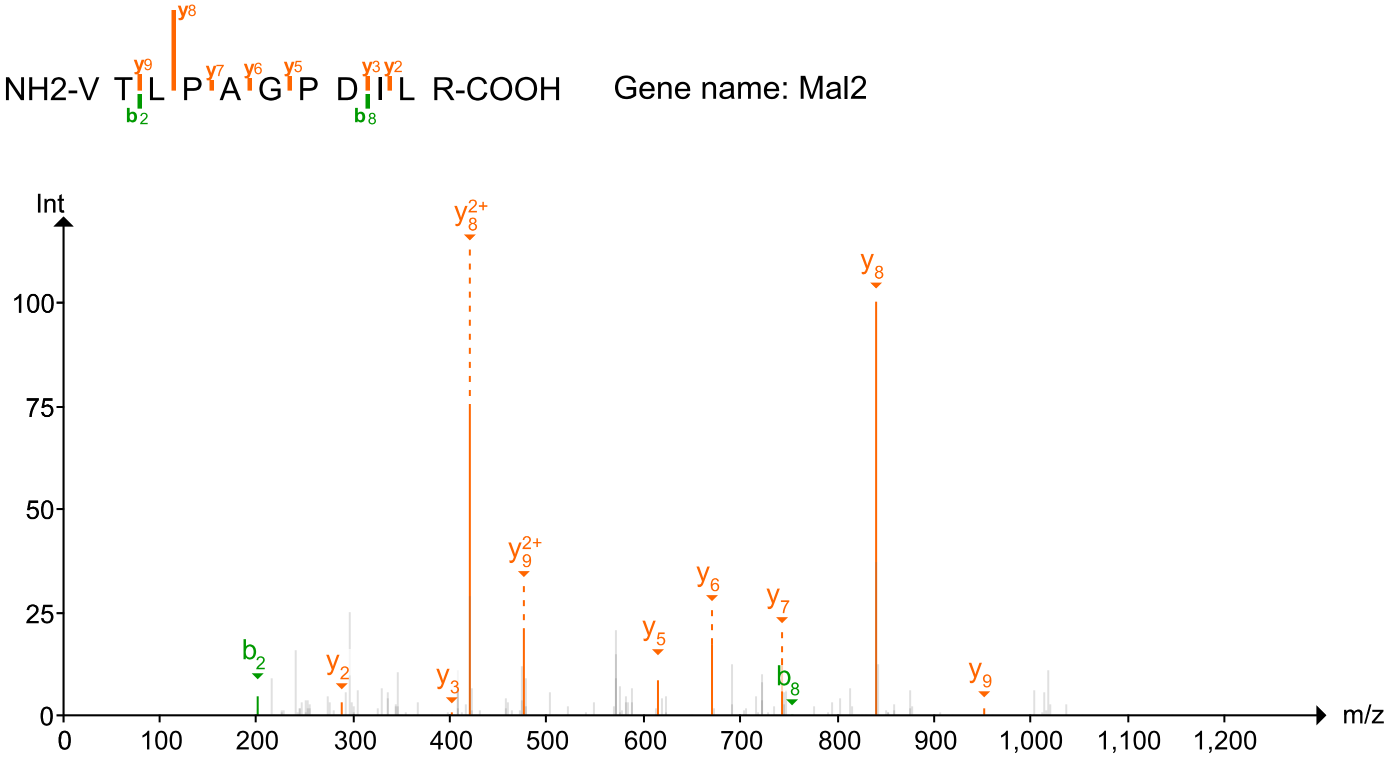

NH2-V T L P A G P D I L R-COOH
Gene name: Mal2
y8 y9 y7 y6 y5 y3 y2
b2 b8
Int
100
75
50
25
0
m/z
0 100 200 300 400 500 600 700 800 900 1,000 1,100 1,200
y8 2+
y8
y9 2+
y6
y7
y5
b2
y2
y3
b8
y9

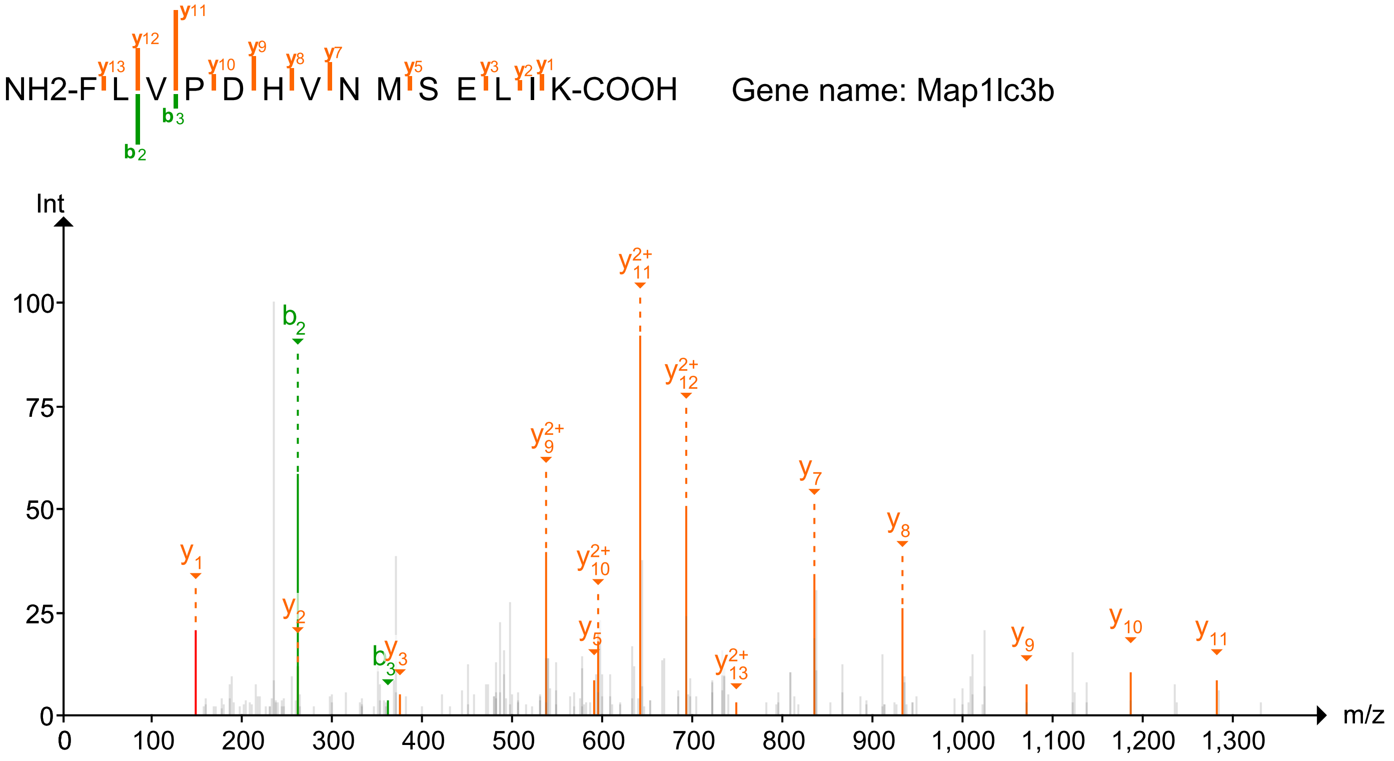
NH2-F L V P D H V N M S E L I K-COOH
Gene name: Map1lc3b
y13 y12 y11 y10 y9 y8 y7 y5 y3 y2 y1
b2 b3
Int
100
75
50
25
0
m/z
0 100 200 300 400 500 600 700 800 900 1,000 1,100 1,200 1,300
y1 b2 y2 b3 y3 y9 2+ y10 2+ y5 y11 2+ y12 2+ y13 2+ y7 y8 y9 y10 y11

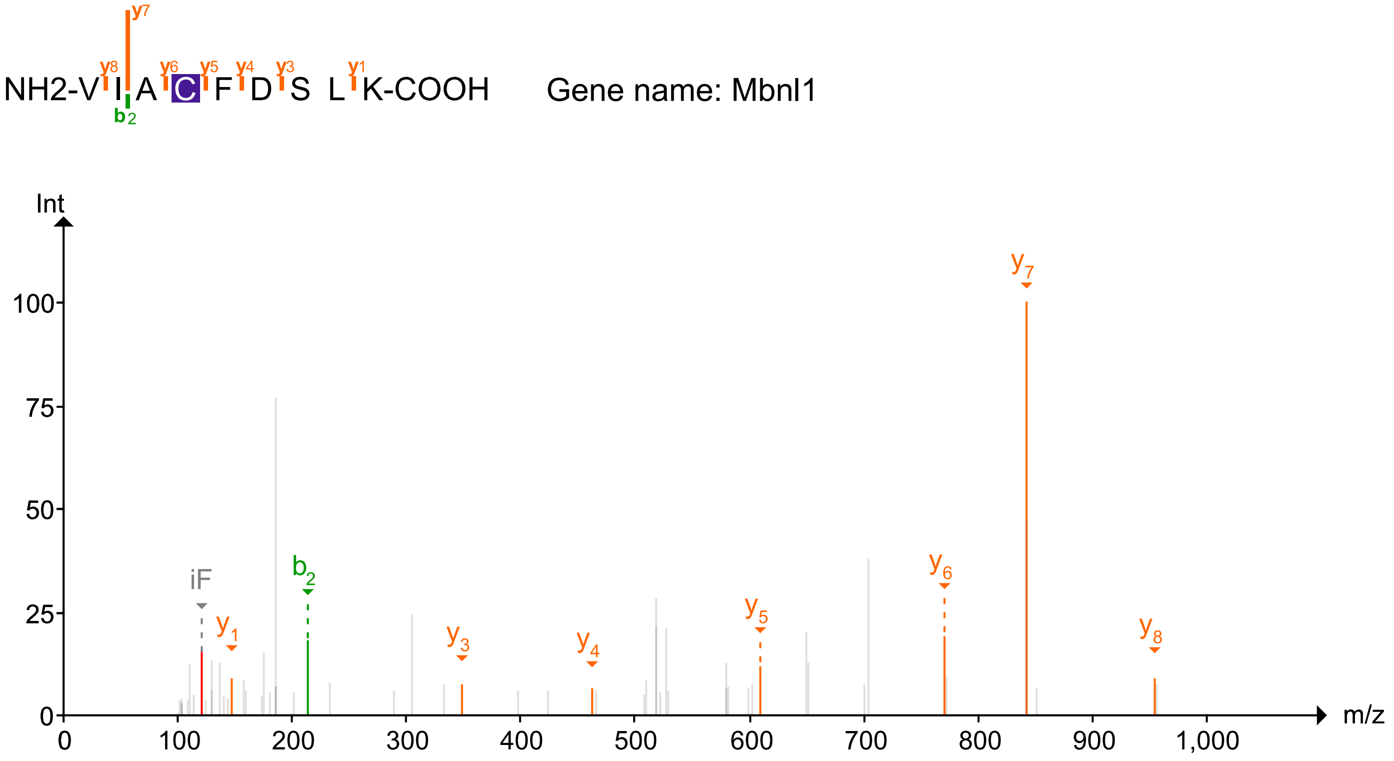

NH2-V I A C F D S L K-COOH
y8 y7 y6 y5 y4 y3 y1
b2
Gene name: Mbnl1
Int
100
75
50
25
0
iF
y1
b2
y3
y4
y5
y6
y7
y8
0
100
200
300
400
500
600
700
800
900
1,000
m/z

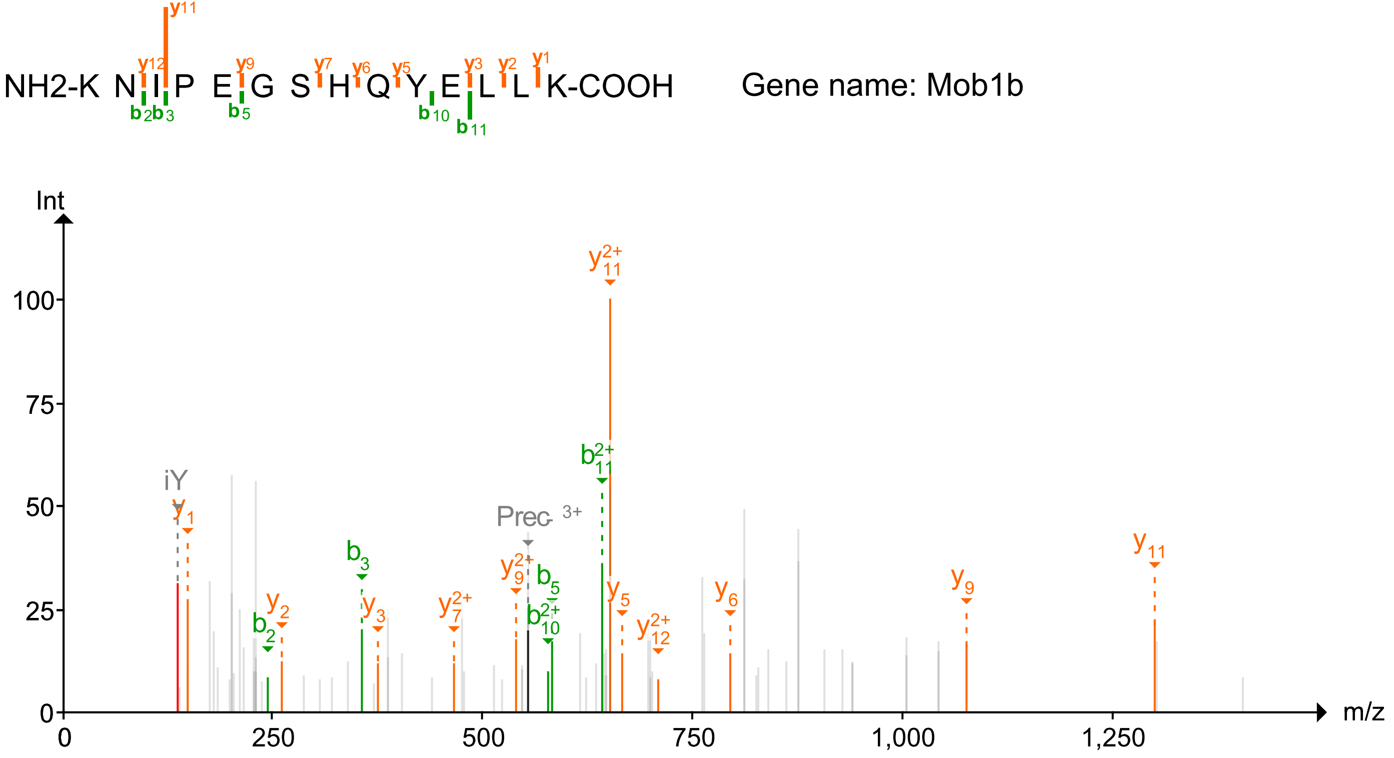

NH2-K N I P E G S H Q Y E L L K-COOH
Gene name: Mob1b
y11
y12
y9
y7
y6
y5
y3
y2
y1
b2
b3
b5
b10
b11
Int
100
75
50
25
0
m/z
0
250
500
750
1,000
1,250
iY
y1
b2
y2
b3
y3
y2+ 7
y2+ 9
Prec- 3+
b5
b2+ 10
b2+ 11
y2+ 11
y5
y2+ 12
y6
y9
y11

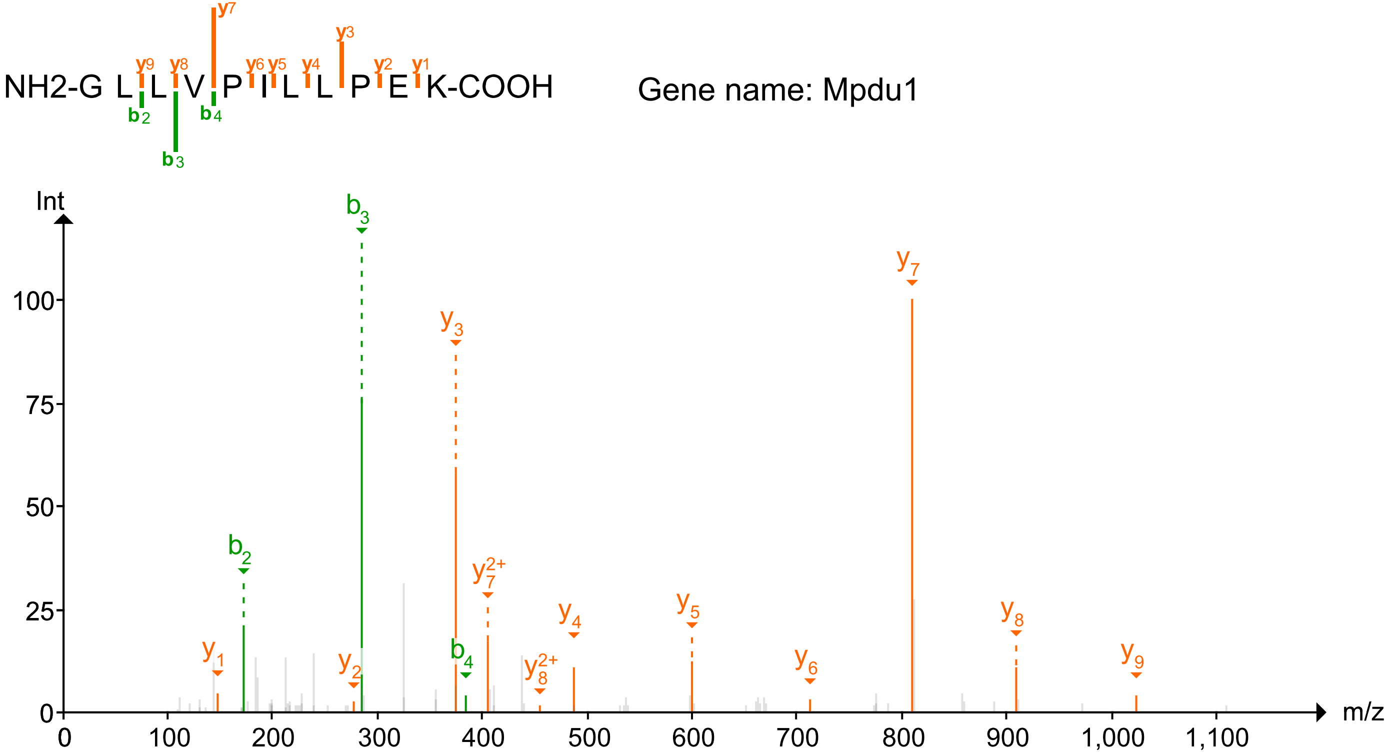

NH2-G L L V P I L L P E K-COOH
Gene name: Mpdu1
y9 y8 y7 y6 y5 y4 y3 y2 y1
b2 b3 b4
Int
m/z
100
75
50
25
0
0 100 200 300 400 500 600 700 800 900 1,000 1,100
b3
y7
y3
b2
y7 2+
y4
y5
y8
y1
y2
b4
y8 2+
y6
y9

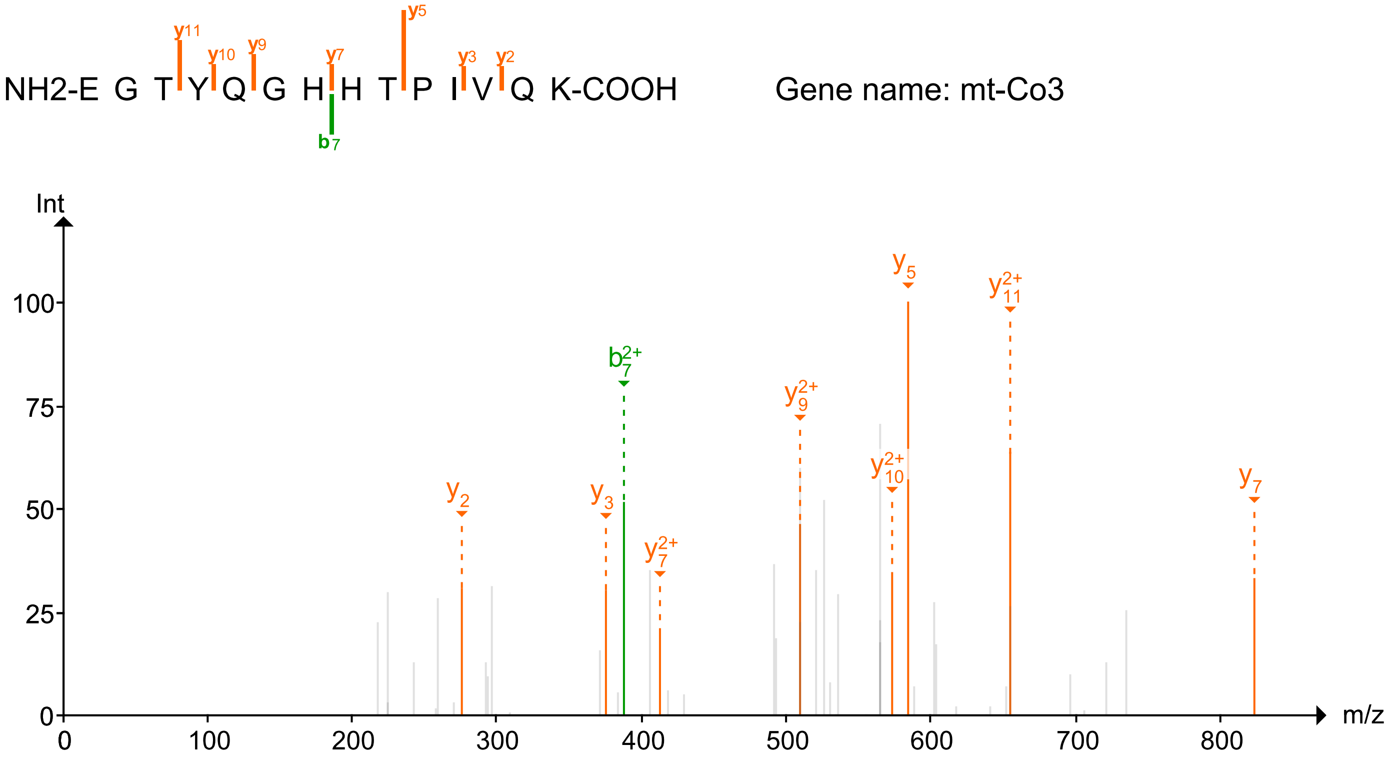

NH2-E G T Y Q G H H T P I V Q K-COOH
Gene name: mt-Co3
y11
y10
y9
y7
y5
y3
y2
b7
Int
m/z
100
75
50
25
0
0
100
200
300
400
500
600
700
800

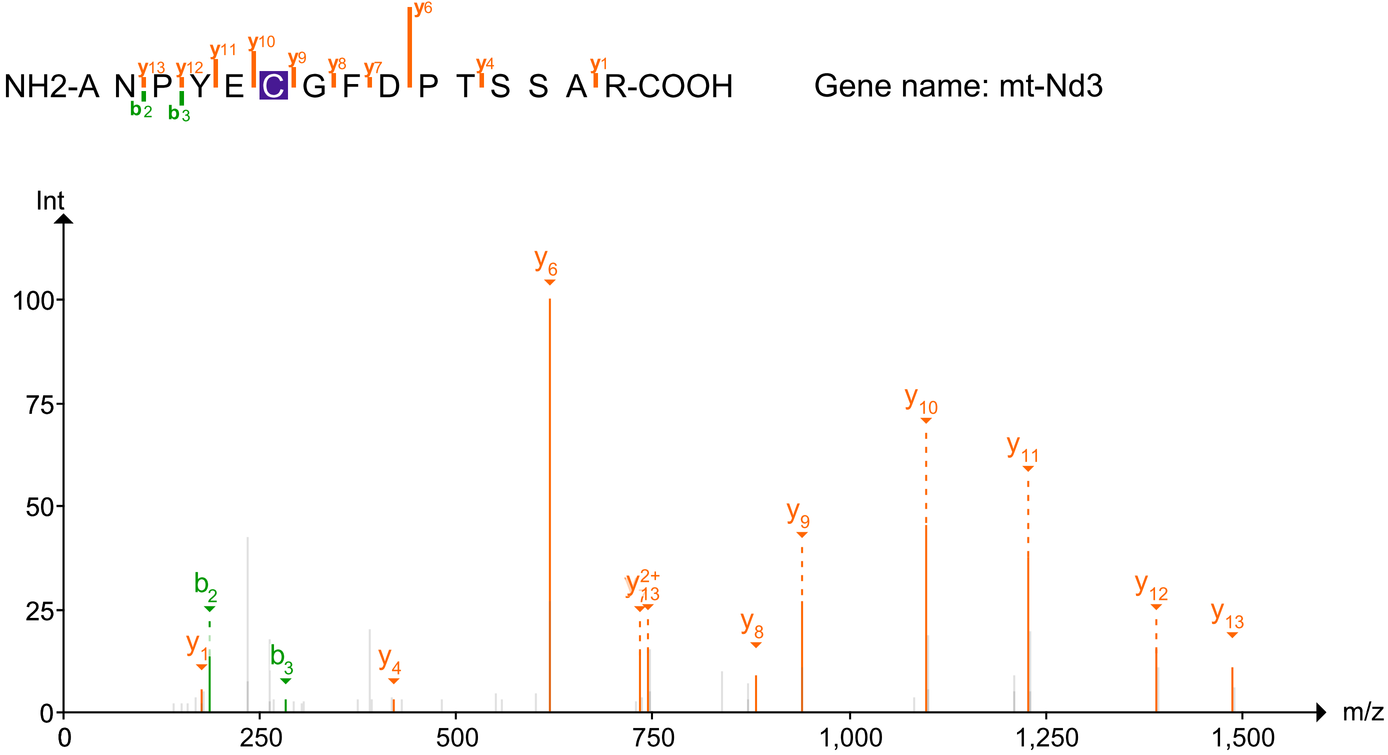

NH2-A N P Y E C G F D P T S S A R-COOH
Gene name: mt-Nd3
y13 y12 y11 y10 y9 y8 y7 y6 y4 y1
b2 b3
Int
100
75
50
25
0
0
250
500
750
1,000
1,250
1,500
m/z
y6
y10
y11
y9
y2+ 13
y12
y13
y8
b2
y1
b3
y4

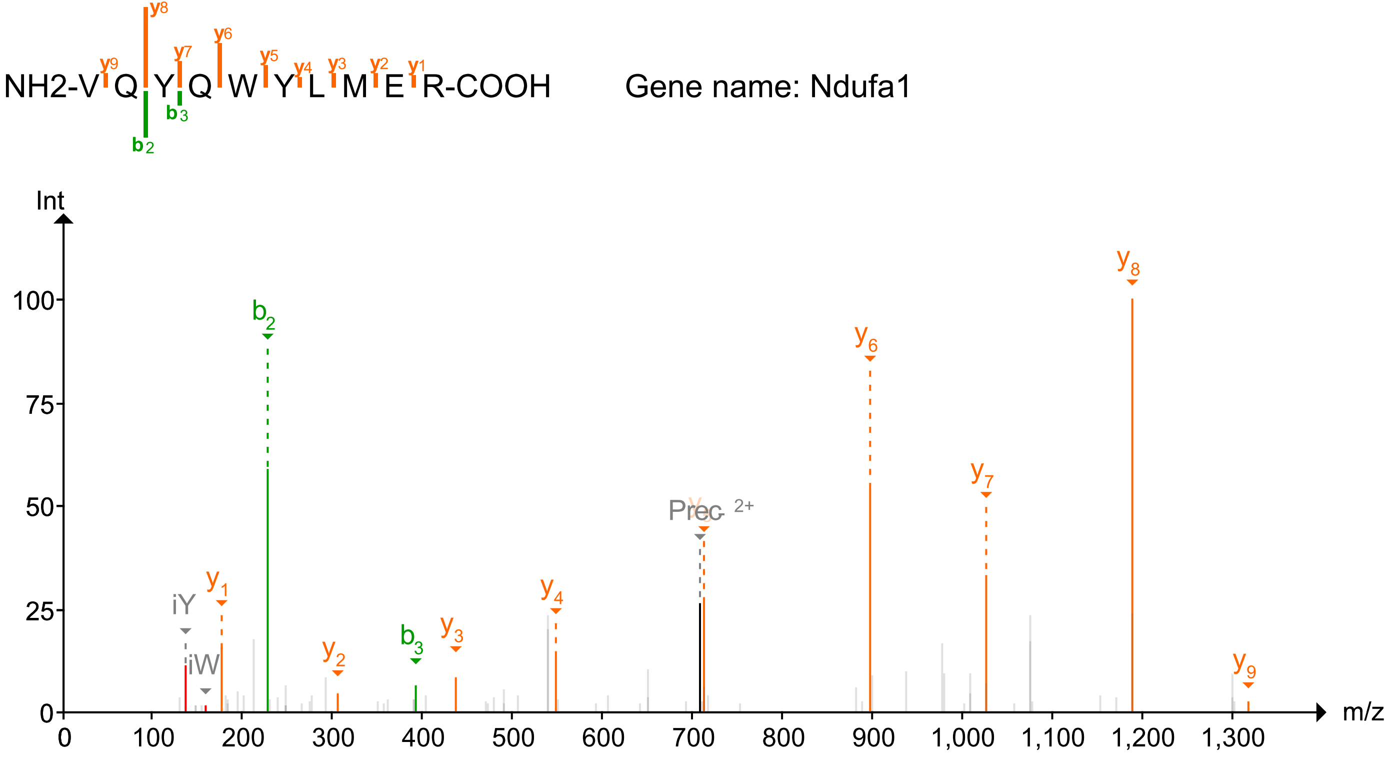

NH2-V Q Y Q W Y L M E R-COOH
Gene name: Ndufa1
y9 y8 y7 y6 y5 y4 y3 y2 y1
b2 b3
Int
m/z
100
75
50
25
0
0 100 200 300 400 500 600 700 800 900 1,000 1,100 1,200 1,300
b2
y8
y6
y7
Prec 2+
y1
iY
iW
y2
b3
y3
y4
y9

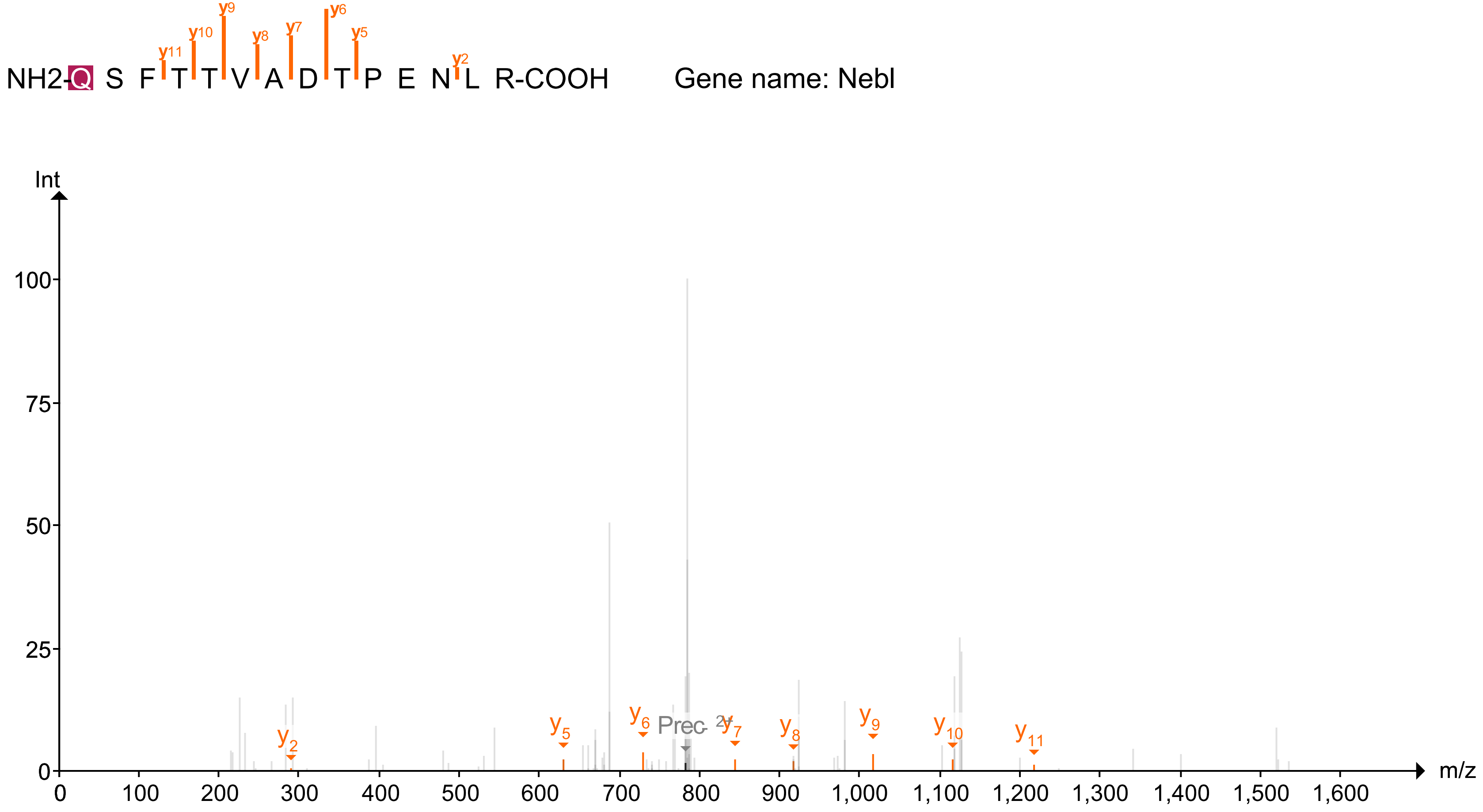

NH2-Q S F T T V A D T P E N L R-COOH
Gene name: Nebl
y11 y10 y9 y8 y7 y6 y5 y2
Int
100
75
50
25
0
y2 y5 y6 Prec- 2+ y7 y8 y9 y10 y11
0 100 200 300 400 500 600 700 800 900 1,000 1,100 1,200 1,300 1,400 1,500 1,600
m/z

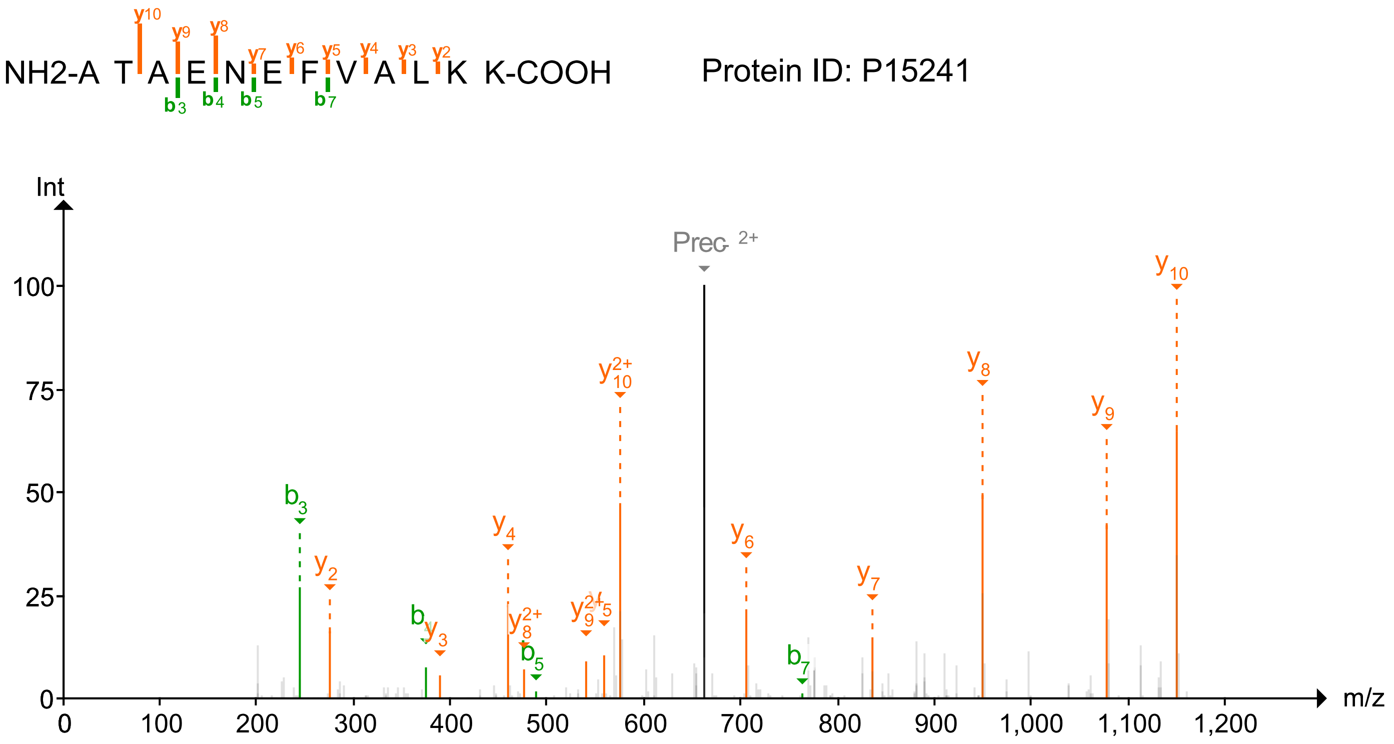
NH2-A T A E N E F V A L K K-COOH
Protein ID: P15241
Int
Prec 2+
m/z
0
25
50
75
100
0
100
200
300
400
500
600
700
800
900
1,000
1,100
1,200

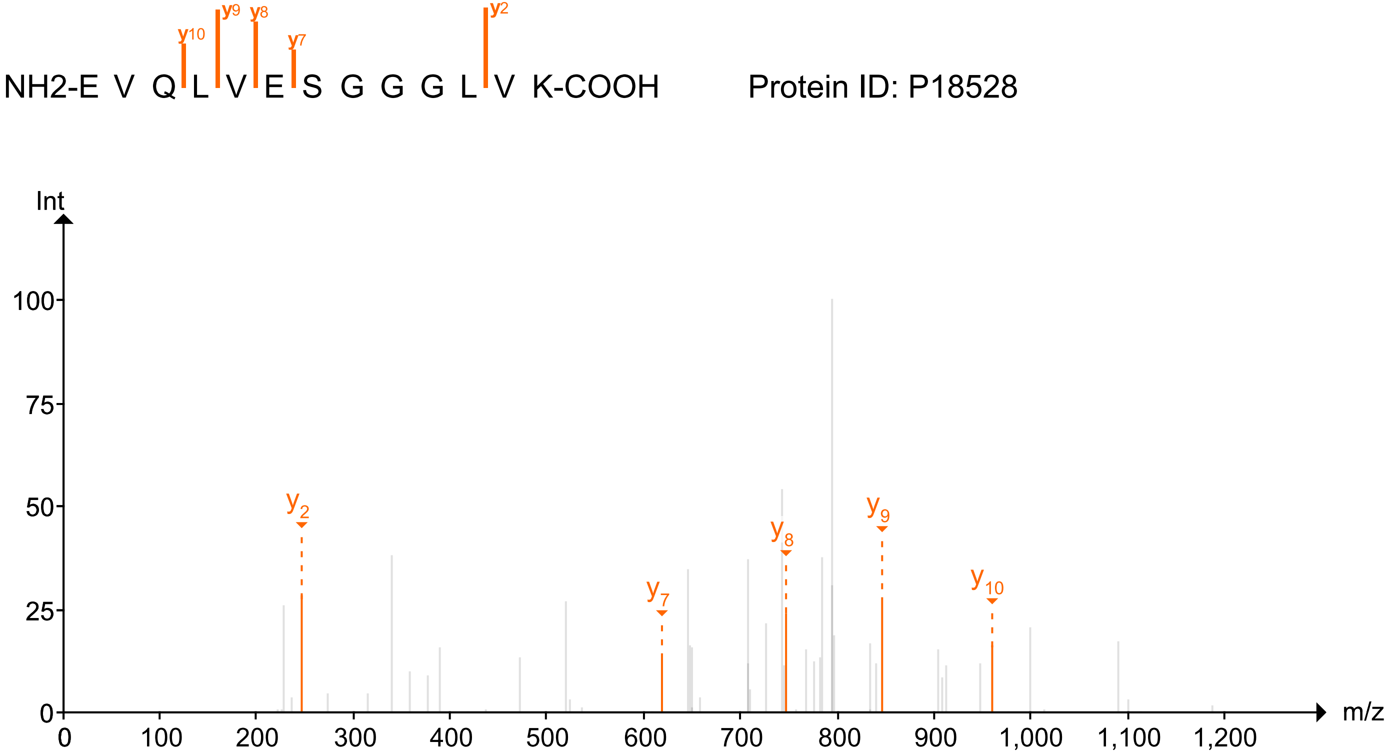

y10
y9
y8
y7
y2
NH2-E V Q L V E S G G G L V K-COOH
Protein ID: P18528
Int
100
75
50
25
0
y2
y7
y8
y9
y10
m/z
0
100
200
300
400
500
600
700
800
900
1,000
1,100
1,200

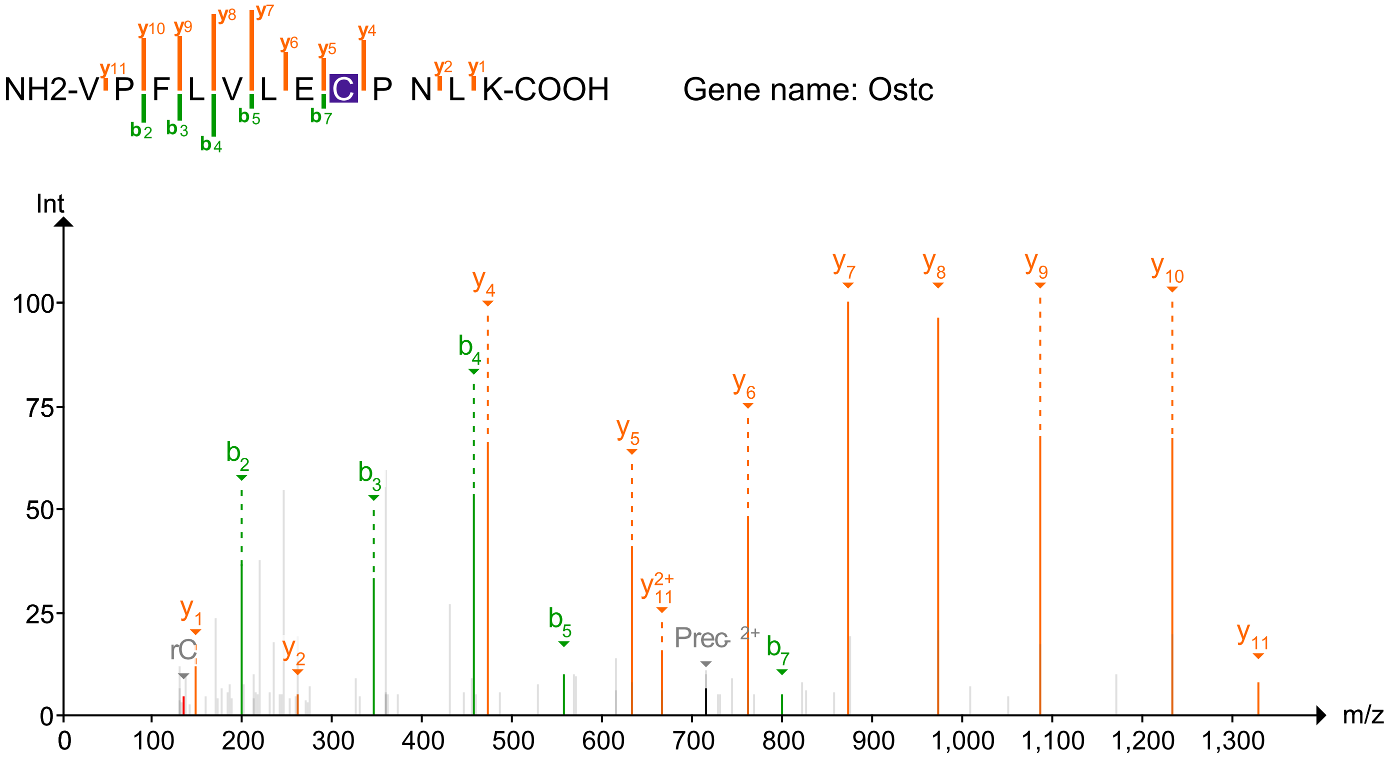

NH2-VPFLVLECPNLK-COOH
Gene name: Ostc
Int
m/z
y1 y2 y4 y5 y6 y7 y8 y9 y10 y11
b2 b3 b4 b5 b7
rC
Prec 2+

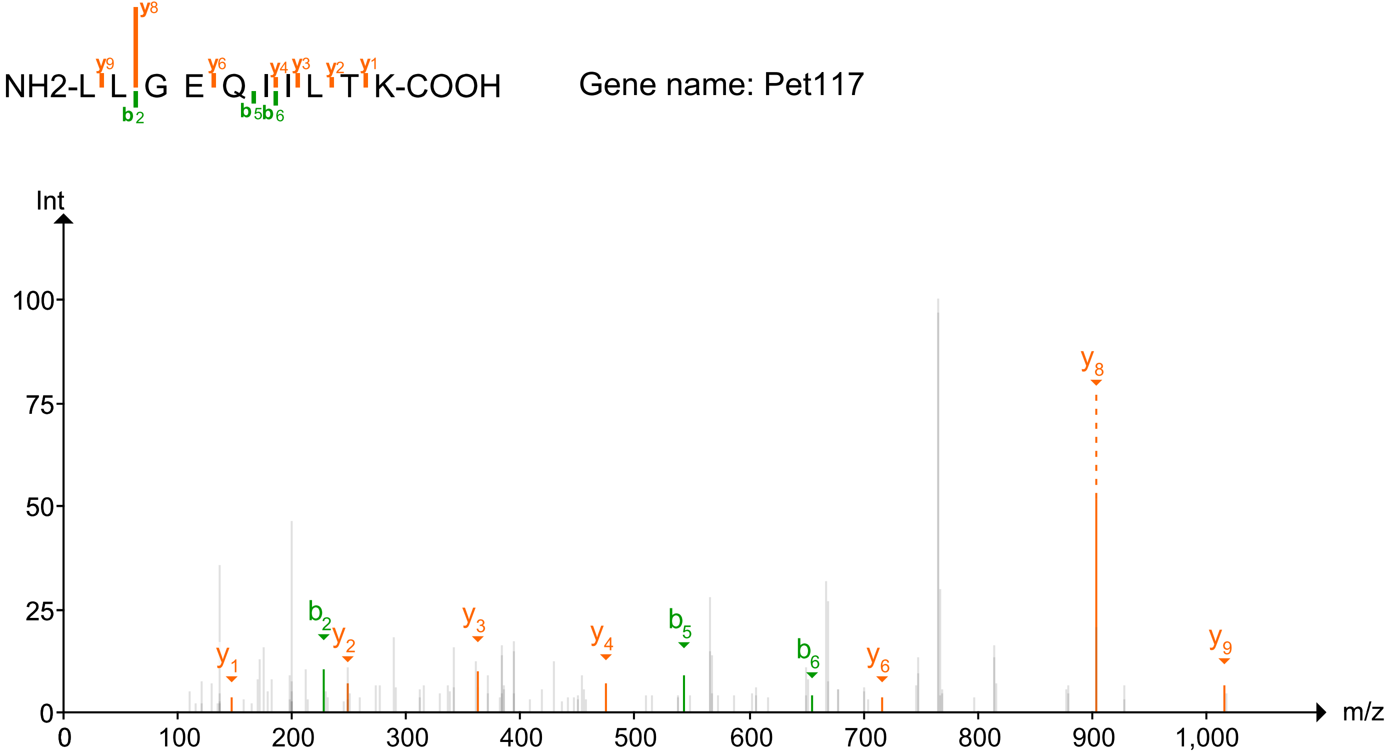

NH2-L L G E Q I I L T K-COOH
Gene name: Pet117
y8
y9
y6
y4
y3
y2
y1
b2
b5
b6
Int
m/z
0
25
50
75
100
0
100
200
300
400
500
600
700
800
900
1,000

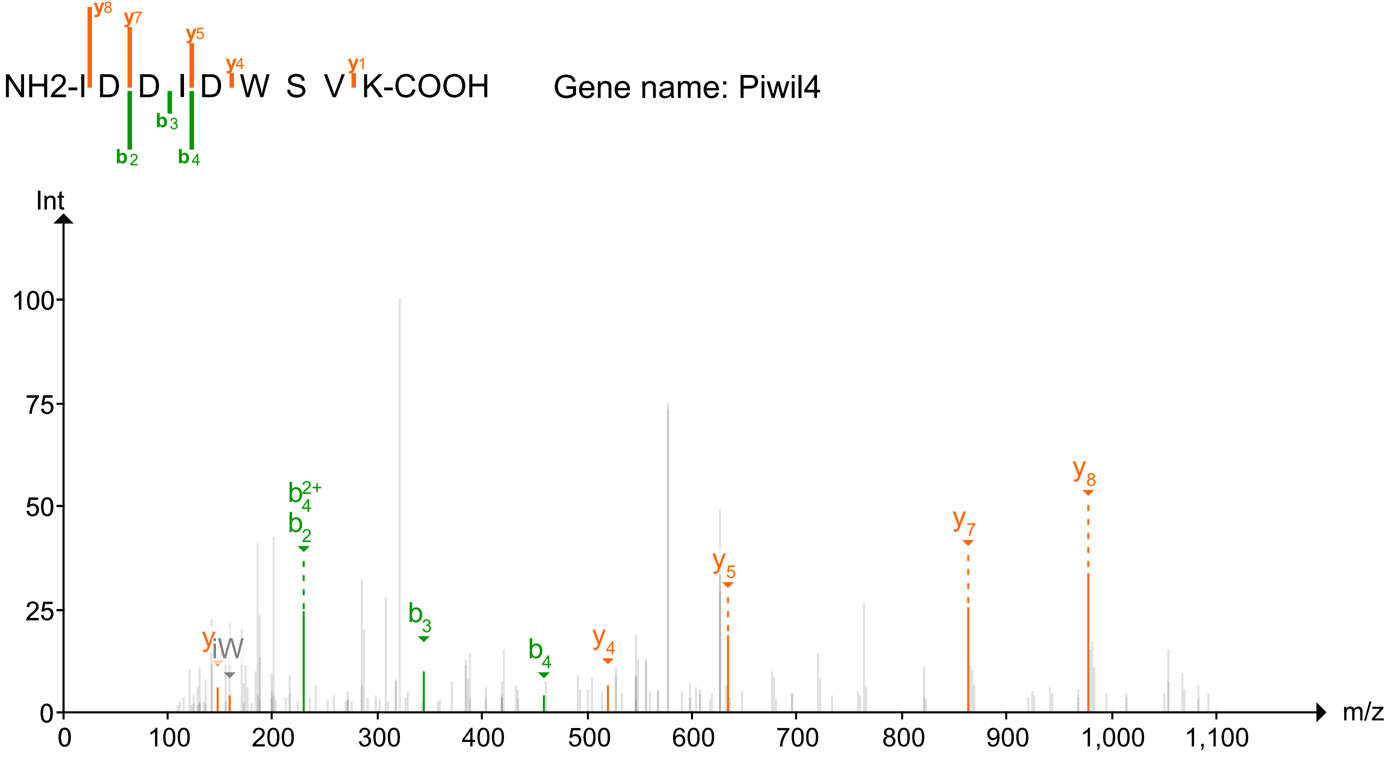

NH2-I D D I D W S V K-COOH
Gene name: Piwil4
y8
y7
y5
y4
y1
b3
b2
b4
Int
m/z
100
75
50
25
0
0
100
200
300
400
500
600
700
800
900
1,000
1,100
b_4^{2+}
b_2
b_3
b_4
y_4
y_5
y_7
y_8
iW

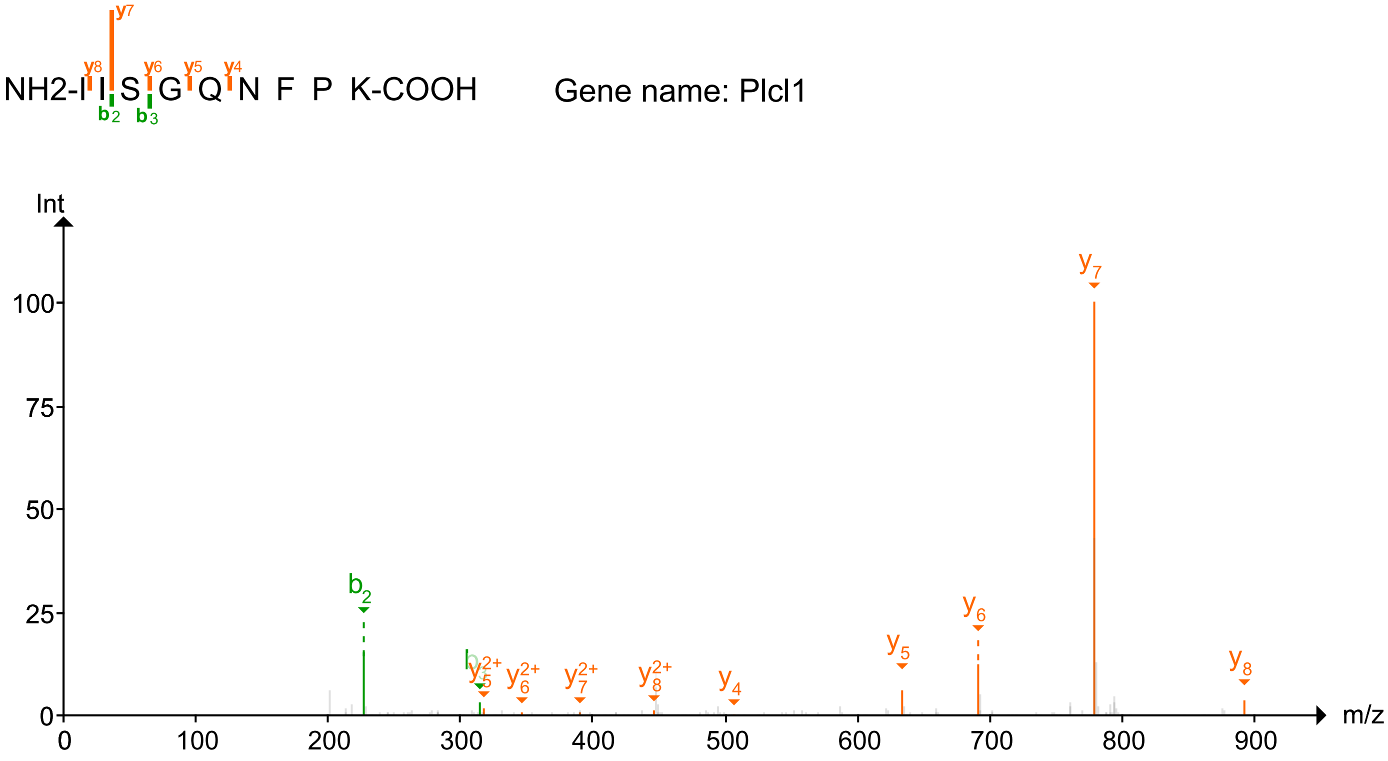

NH2-I I S G Q N F P K-COOH
Gene name: Plcl1
y7
y8
y6
y5
y4
b2
b3
Int
100
75
50
25
0
0
100
200
300
400
500
600
700
800
900
m/z
y7
b2
y5 2+
y6 2+
y7 2+
y8 2+
y4
y5
y6
y8

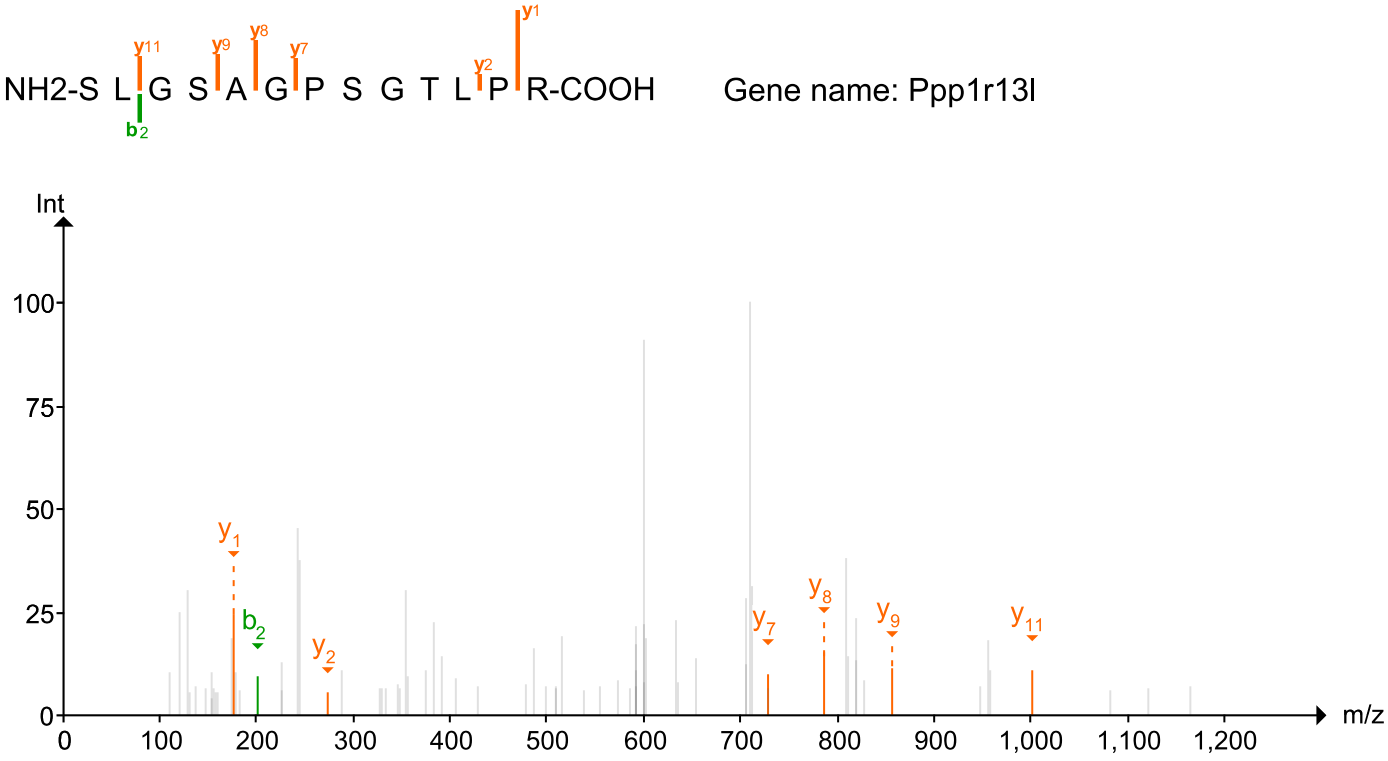

NH2-S L G S A G P S G T L P R-COOH
Gene name: Ppp1r13l
y11 y9 y8 y7 y2 y1
b2
Int
100
75
50
25
0
y1 b2 y2 y7 y8 y9 y11
0 100 200 300 400 500 600 700 800 900 1,000 1,100 1,200
m/z

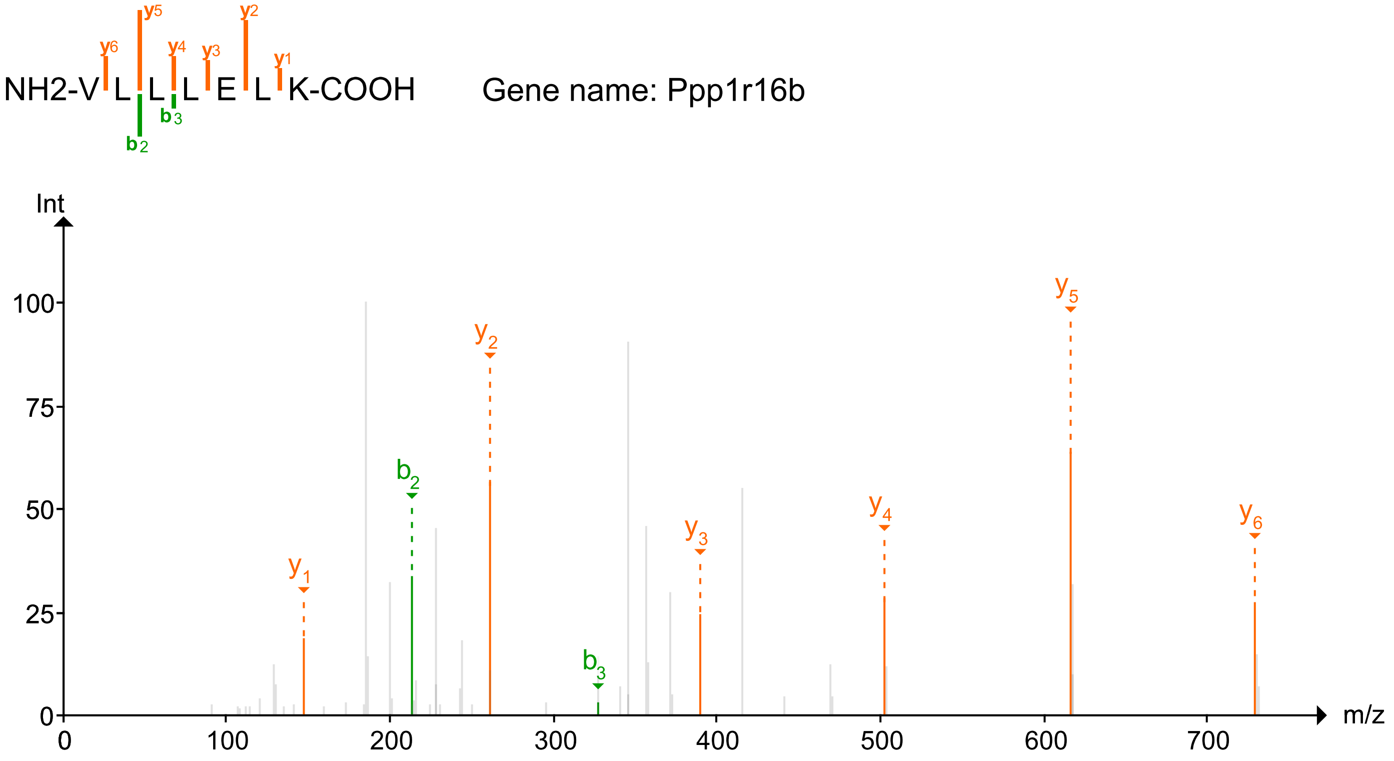
NH2-V L L L E L K-COOH
Gene name: Ppp1r16b
y6 y5 y4 y3 y2 y1
b2 b3
Int
100
75
50
25
0
m/z
0 100 200 300 400 500 600 700
y1 b2 y2 b3 y3 y4 y5 y6

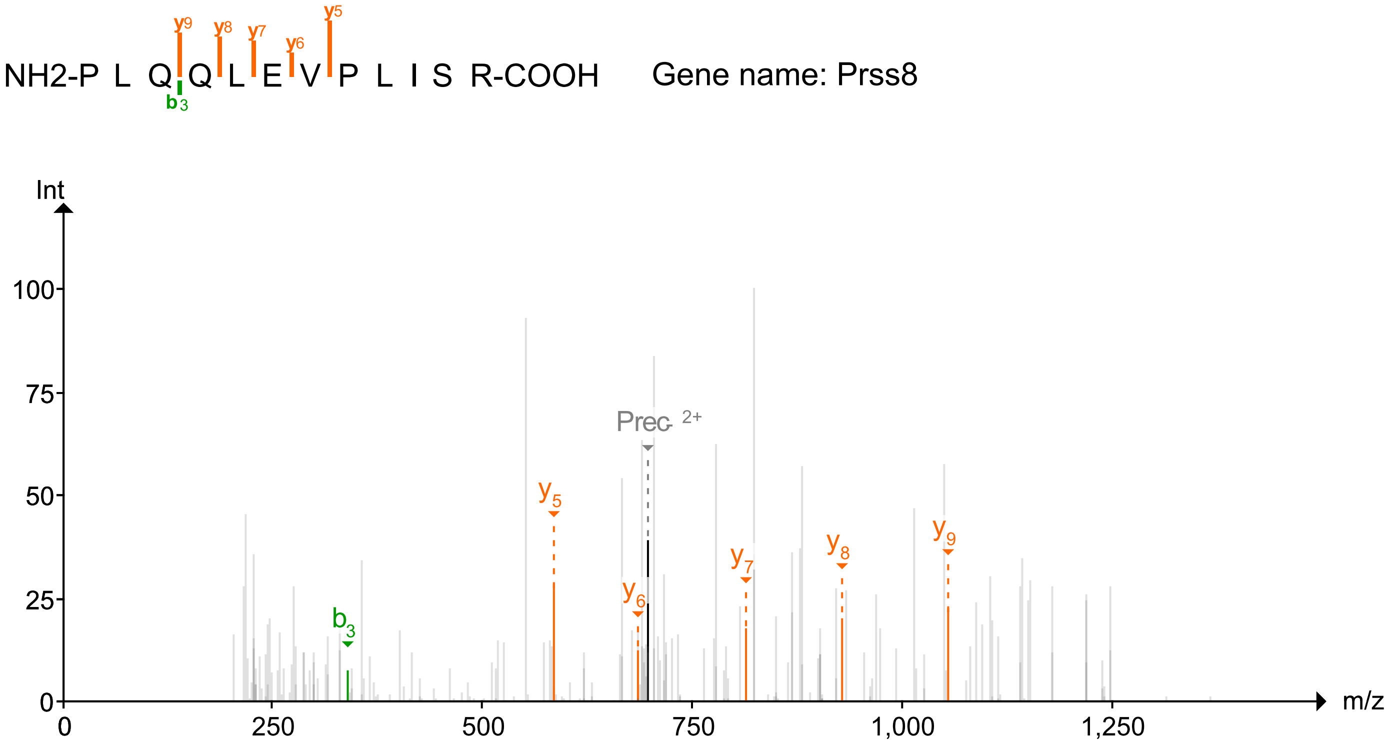

NH2-P L Q Q L E V P L I S R-COOH
Gene name: Prss8
y9 y8 y7 y6 y5
b3
Int
100
75
50
25
0
Prec 2+
y5
y6
y7
y8
y9
b3
0
250
500
750
1,000
1,250
m/z

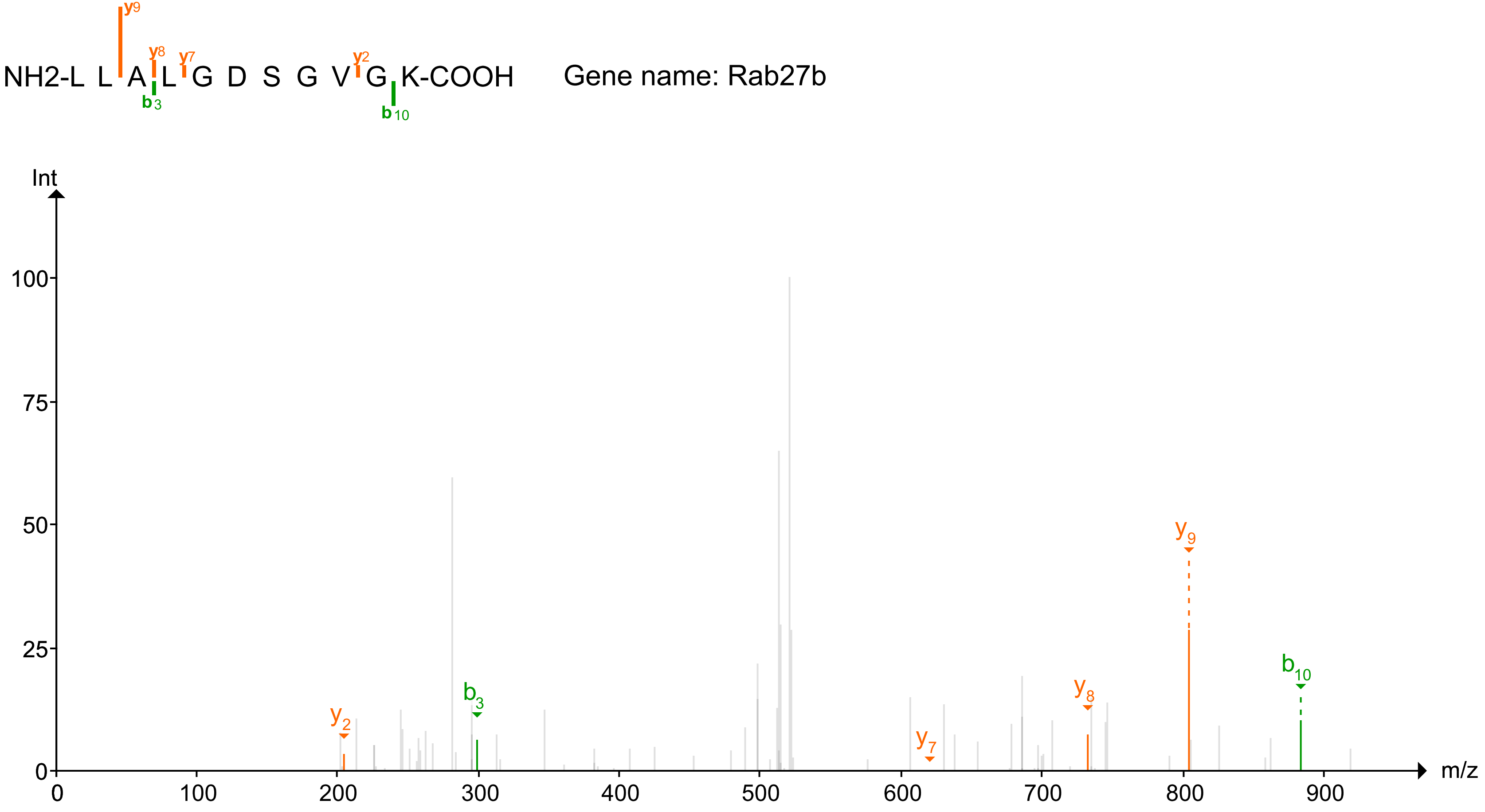

NH2-L L A L G D S G V G K-COOH
Gene name: Rab27b
y9
y8
y7
y2
b3
b10
Int
100
75
50
25
0
0
100
200
300
400
500
600
700
800
900
m/z
y2
b3
y7
y8
y9
b10

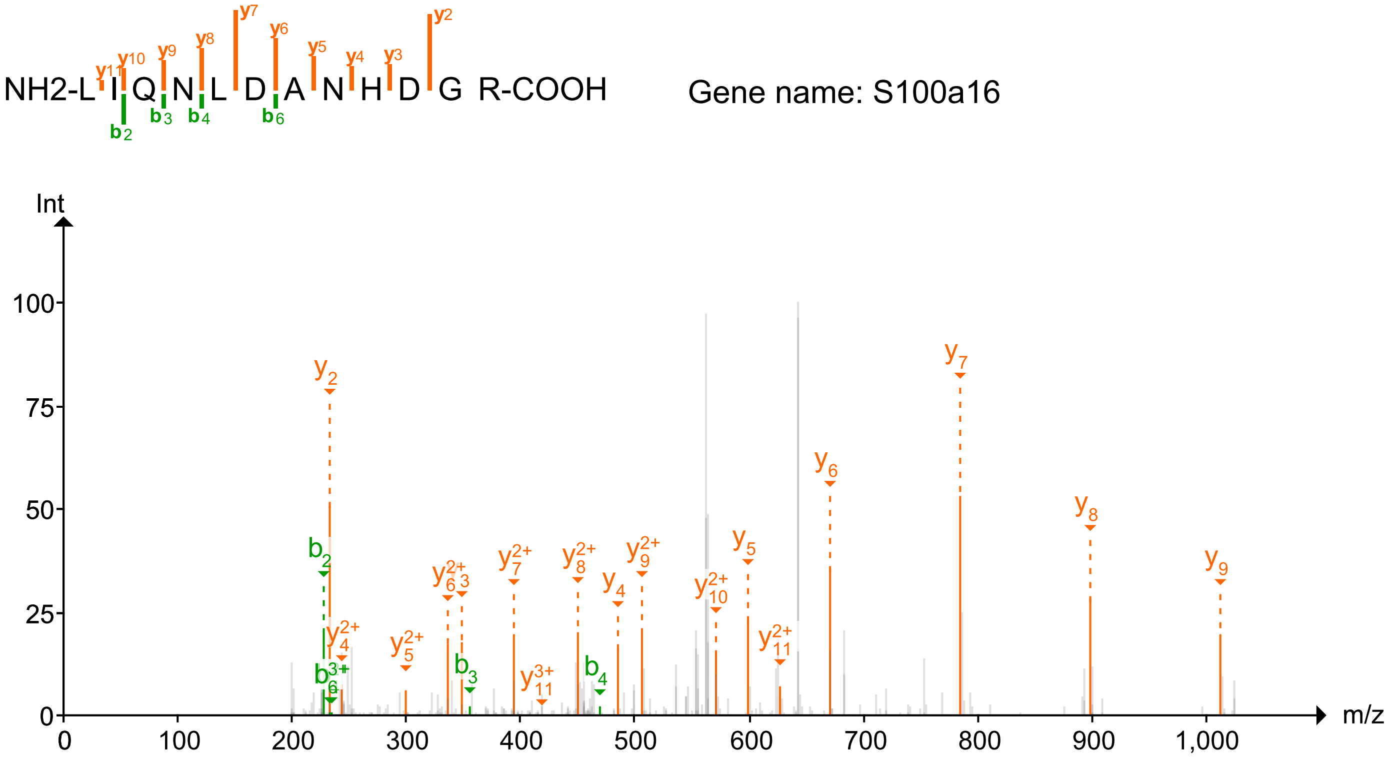
NH2-L I Q N L D A N H D G R-COOH
y11 y10 y9 y8 y7 y6 y5 y4 y3 y2
b2 b3 b4 b6
Gene name: S100a16
Int
100
75
50
25
0
m/z
0 100 200 300 400 500 600 700 800 900 1,000
y2 b2 y4 2+ b6 3+ y5 2+ y6 2+ y3 b3 y7 2+ y11 3+ y8 2+ y4 b4 y9 2+ y10 2+ y5 y11 2+ y6 y7 y8 y9

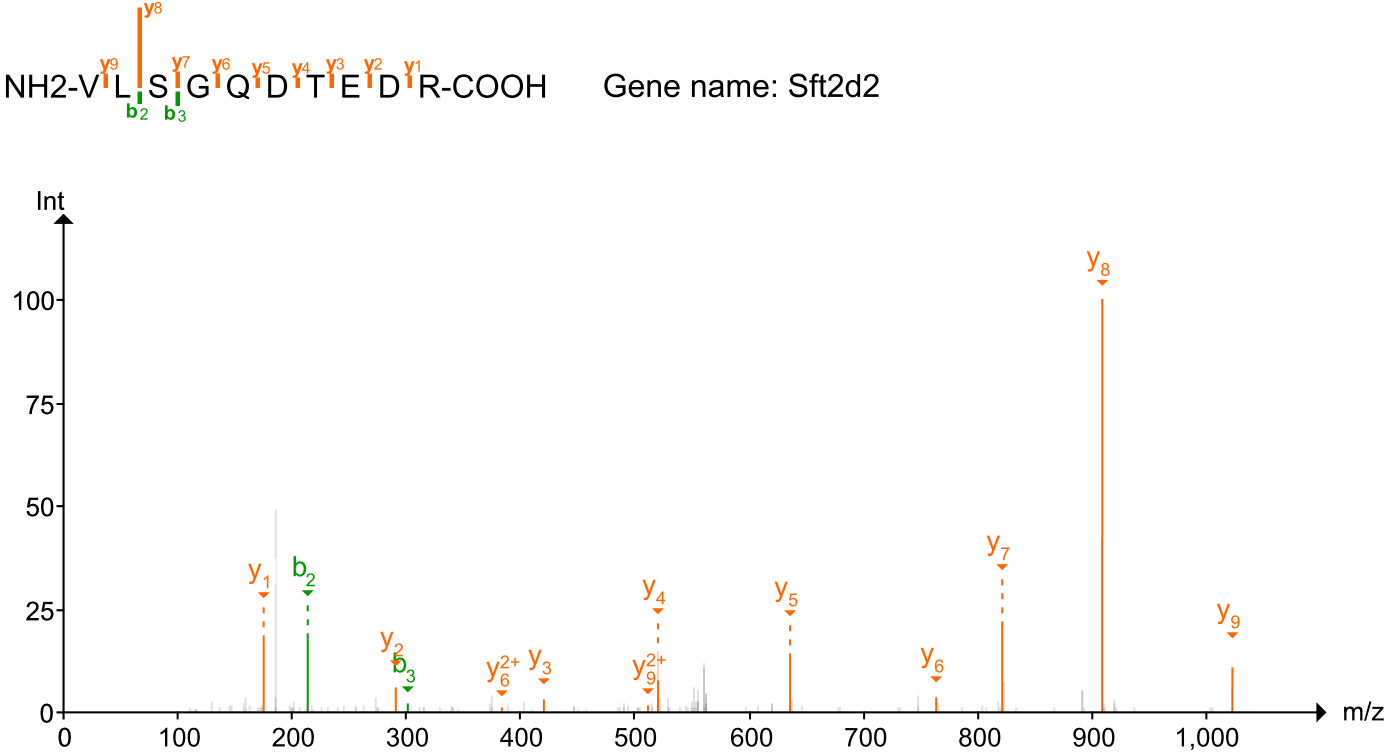

NH2-V L S G Q D T E D R-COOH
Gene name: Sft2d2
y9 y8 y7 y6 y5 y4 y3 y2 y1
b2 b3
Int
100
75
50
25
0
m/z
0
100
200
300
400
500
600
700
800
900
1,000
y8
y7
y9
y1
b2
y4
y5
y2
b3
y6 2+
y3
y9 2+
y6

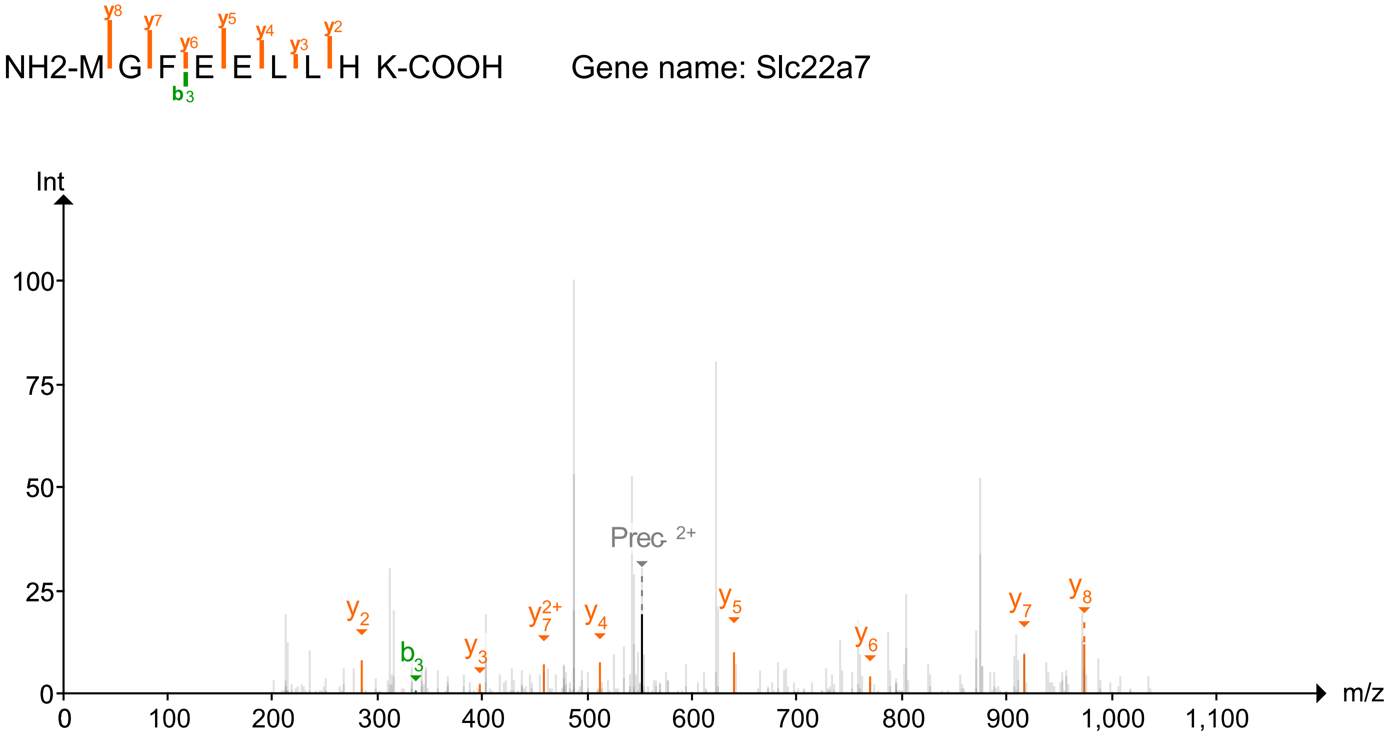

NH2-M G F E E L L H K-COOH
y8 y7 y6 y5 y4 y3 y2
b3
Gene name: Slc22a7
Int
100
75
50
25
0
m/z
0 100 200 300 400 500 600 700 800 900 1,000 1,100
Prec 2+
y2 b3 y3 y7 2+ y4 y5 y6 y7 y8

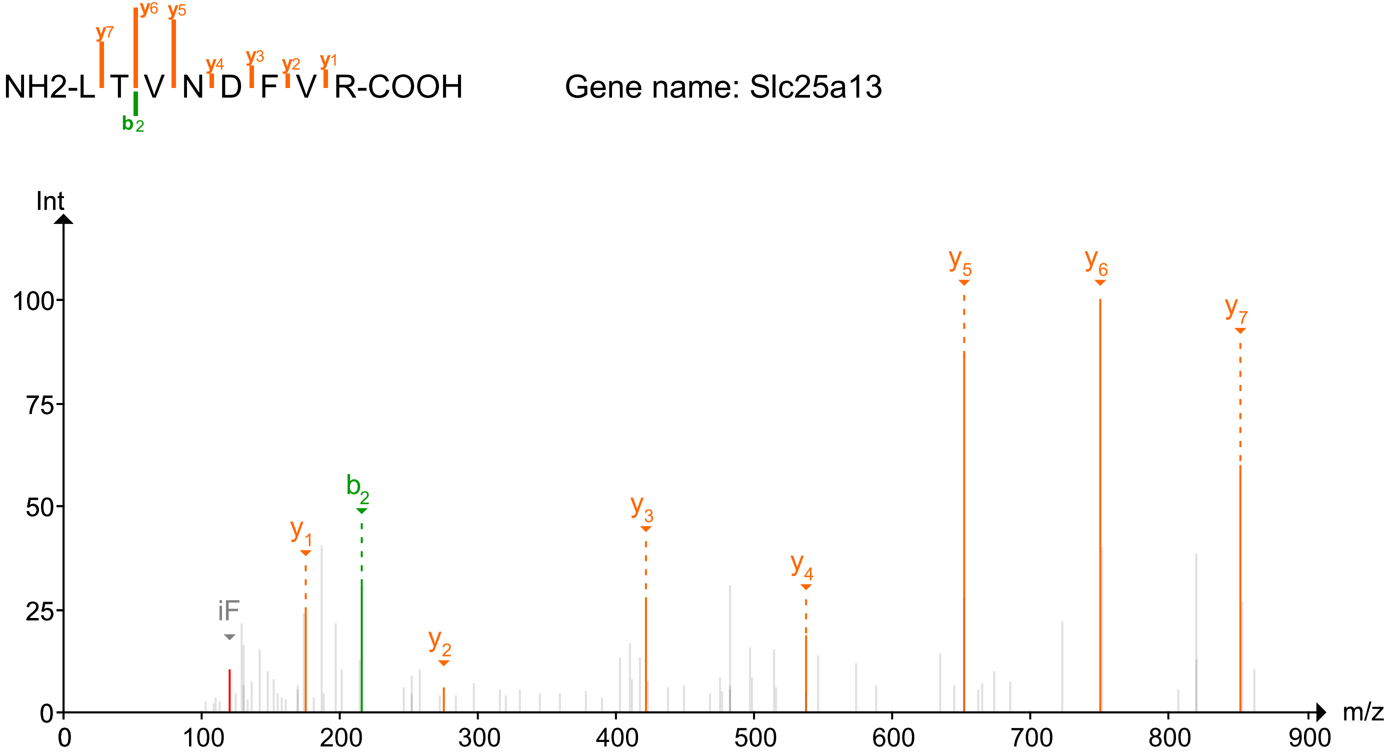

NH2-L T V N D F V R-COOH
y7 y6 y5 y4 y3 y2 y1
b2
Gene name: Slc25a13
Int
m/z
y5 y6 y7 b2 y1 y3 y4 y2 iF
0 25 50 75 100
0 100 200 300 400 500 600 700 800 900

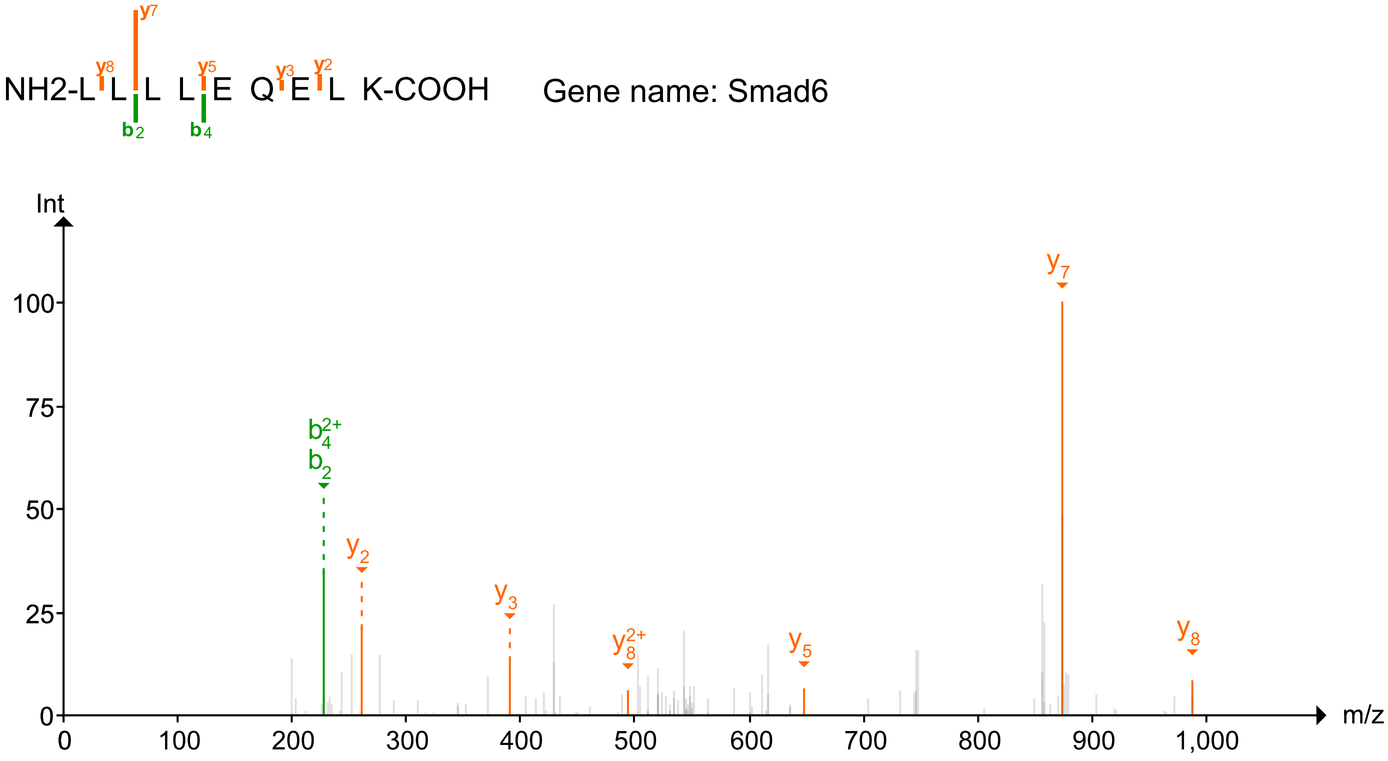
NH2-L L L L E Q E L K-COOH
y8 y7 y5 y3 y2
b2 b4
Gene name: Smad6
Int
100
75
50
25
0
m/z
0
100
200
300
400
500
600
700
800
900
1,000
b_4^{2+}
b_2
y_2
y_3
y_8^{2+}
y_5
y_7
y_8

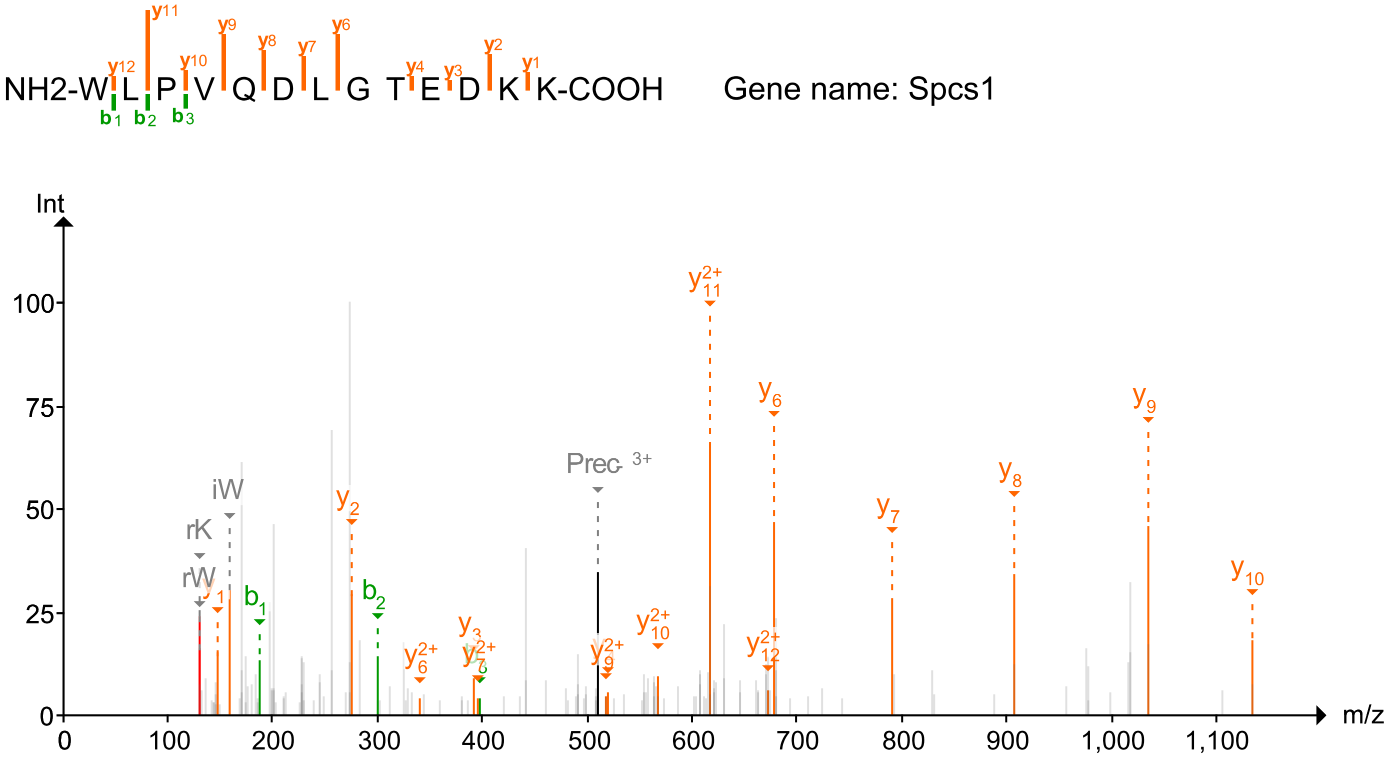
NH2-W L P V Q D L G T E D K K-COOH
Gene name: Spcs1
y12 y11 y10 y9 y8 y7 y6 y4 y3 y2 y1
b1 b2 b3
Int
m/z
0
25
50
75
100
0
100
200
300
400
500
600
700
800
900
1,000
1,100
rW
rK
iW
y_1
b_1
y_2
b_2
y_6^{2+}
y_3
y_7^{2+}
Prec 3+
y_9^{2+}
y_{10}^{2+}
y_{11}^{2+}
y_{12}^{2+}
y_6
y_7
y_8
y_9
y_{10}

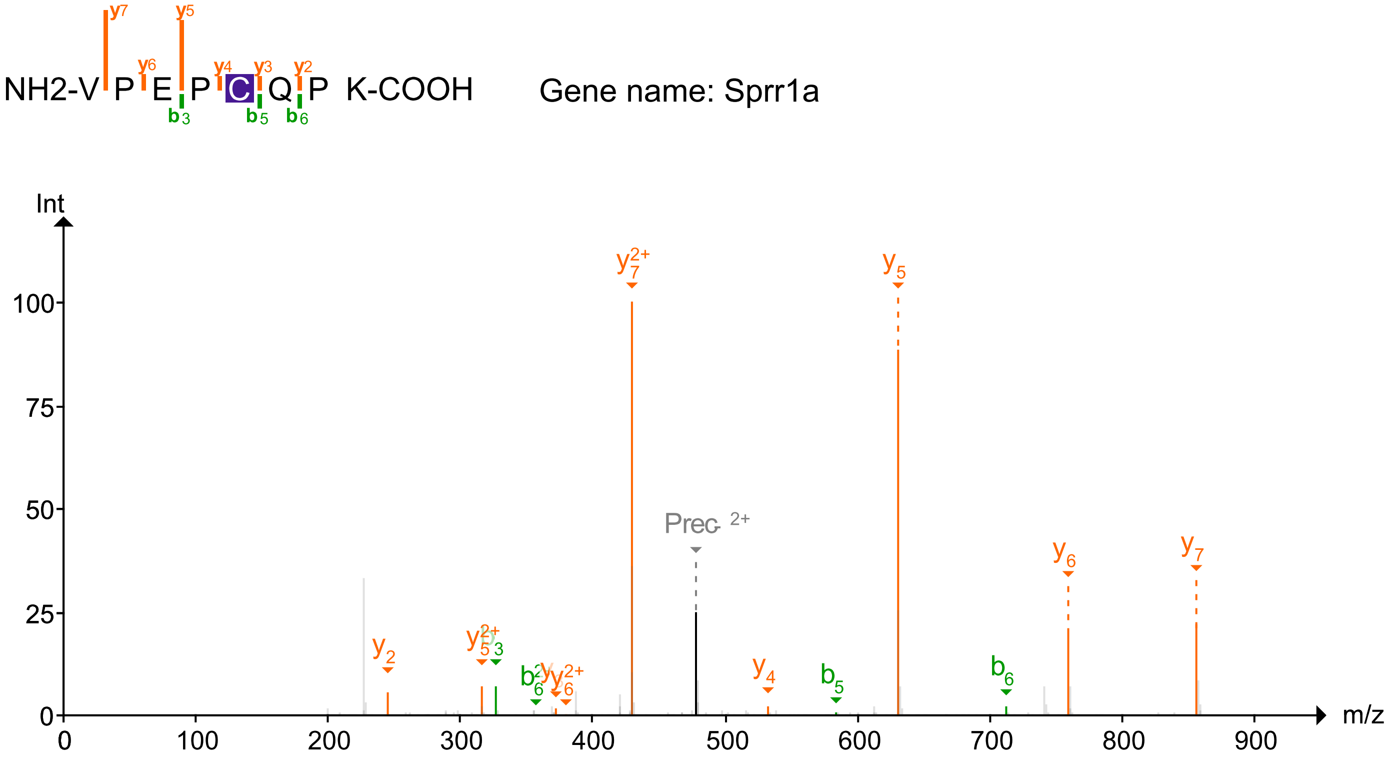
NH2-V P E P C Q P K-COOH
Gene name: Sprr1a
y7
y6
y5
y4
y3
y2
b3
b5
b6
Int
100
75
50
25
0
m/z
0
100
200
300
400
500
600
700
800
900
y7 2+
y5
Prec 2+
y6
y7
y2
y5 2+
b3
b6 2+
y6 2+
y4
b5
b6

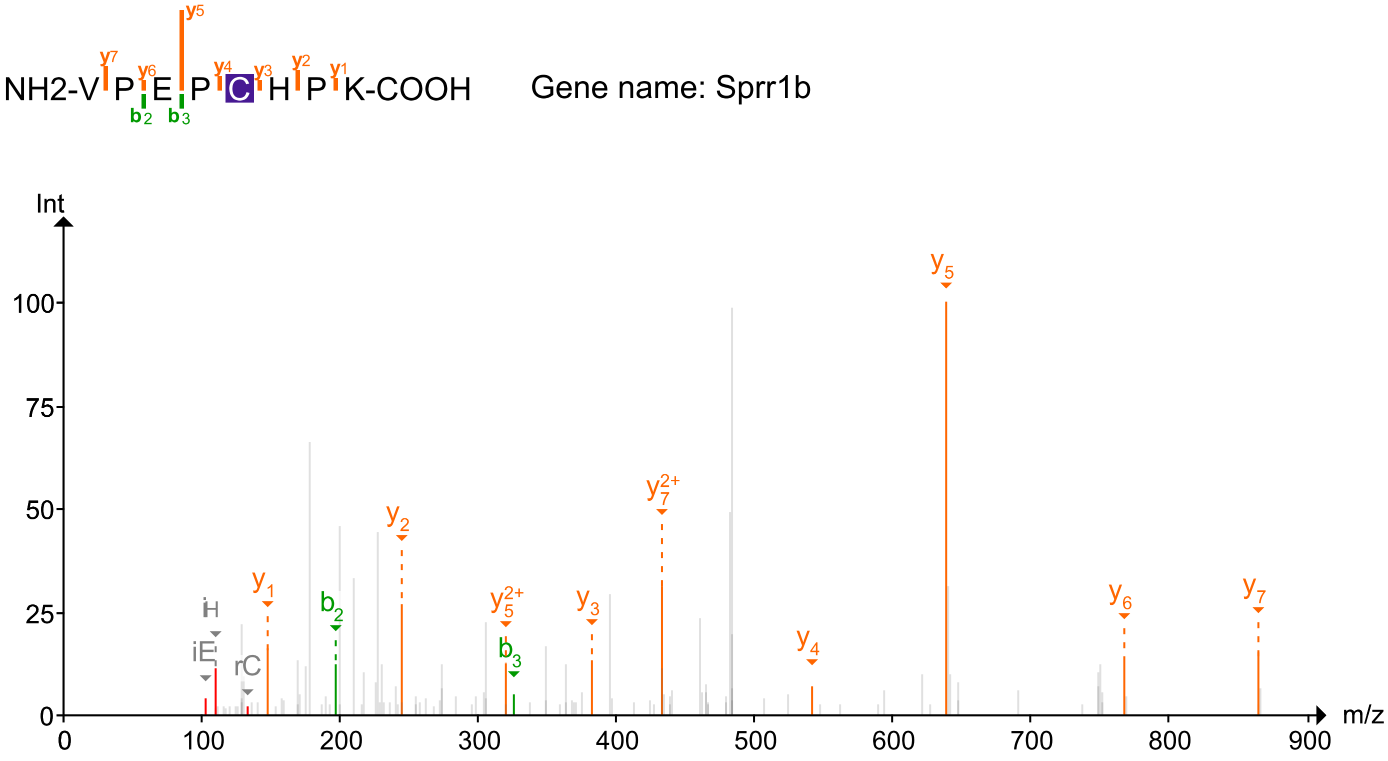
NH2-V P E P C H P K-COOH
y7 y6 y5 y4 y3 y2 y1
b2 b3
Gene name: Sprr1b
Int
m/z
0
25
50
75
100
0
100
200
300
400
500
600
700
800
900
y5
y7 2+
y2
y1
b2
y5 2+
y3
b3
y4
y6
y7
iH
iE
rC

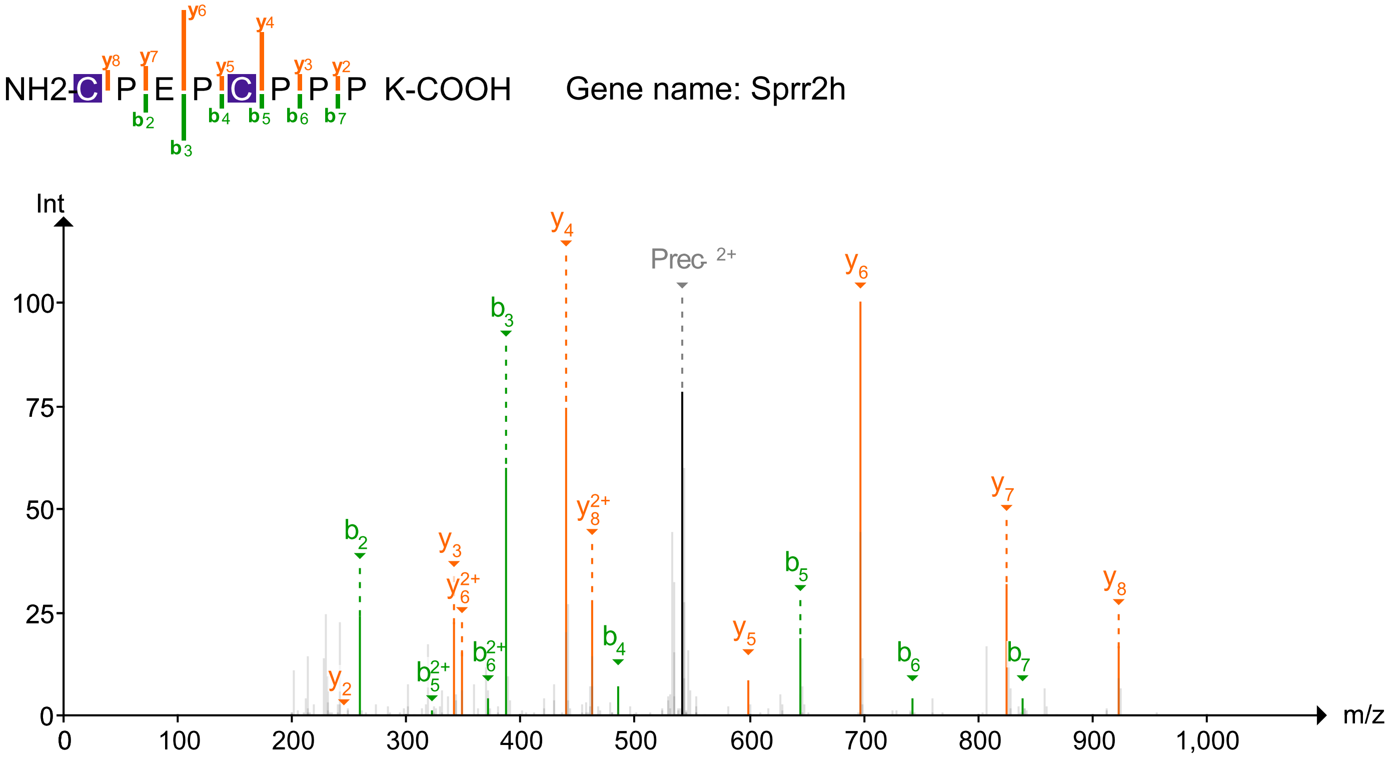
NH2-C P E P C P P P K-COOH
Gene name: Sprr2h
Int
m/z
Prec 2+
b2 b3 b4 b5 b6 b7
y2 y3 y4 y5 y6 y7 y8
y6 2+
y8 2+
b5 2+
b6 2+
0
25
50
75
100
0
100
200
300
400
500
600
700
800
900
1,000

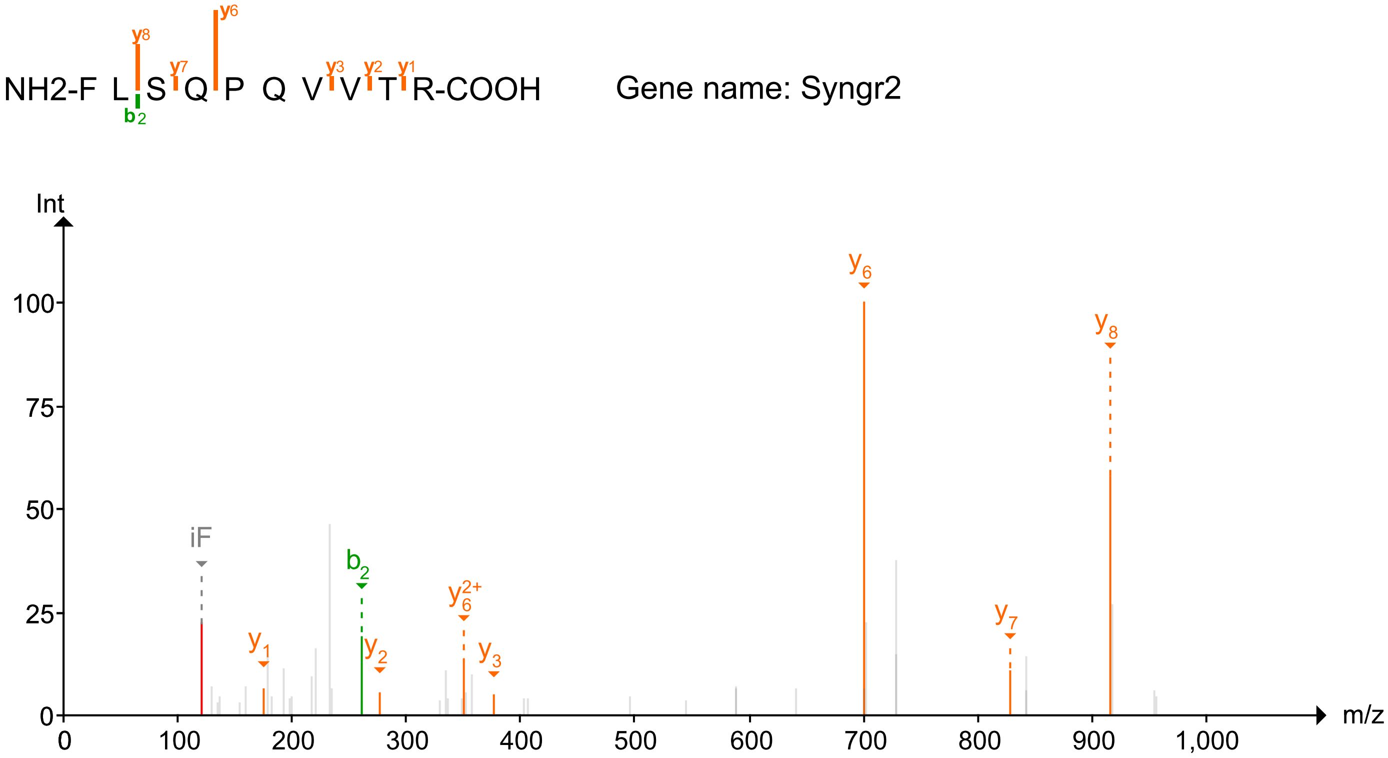

NH2-F L S Q P Q V V T R-COOH
y8
y7
y6
y3
y2
y1
b2
Gene name: Syngr2
Int
m/z
y6
y8
iF
b2
y1
y2
y6 2+
y3
y7
0
25
50
75
100
0
100
200
300
400
500
600
700
800
900
1,000

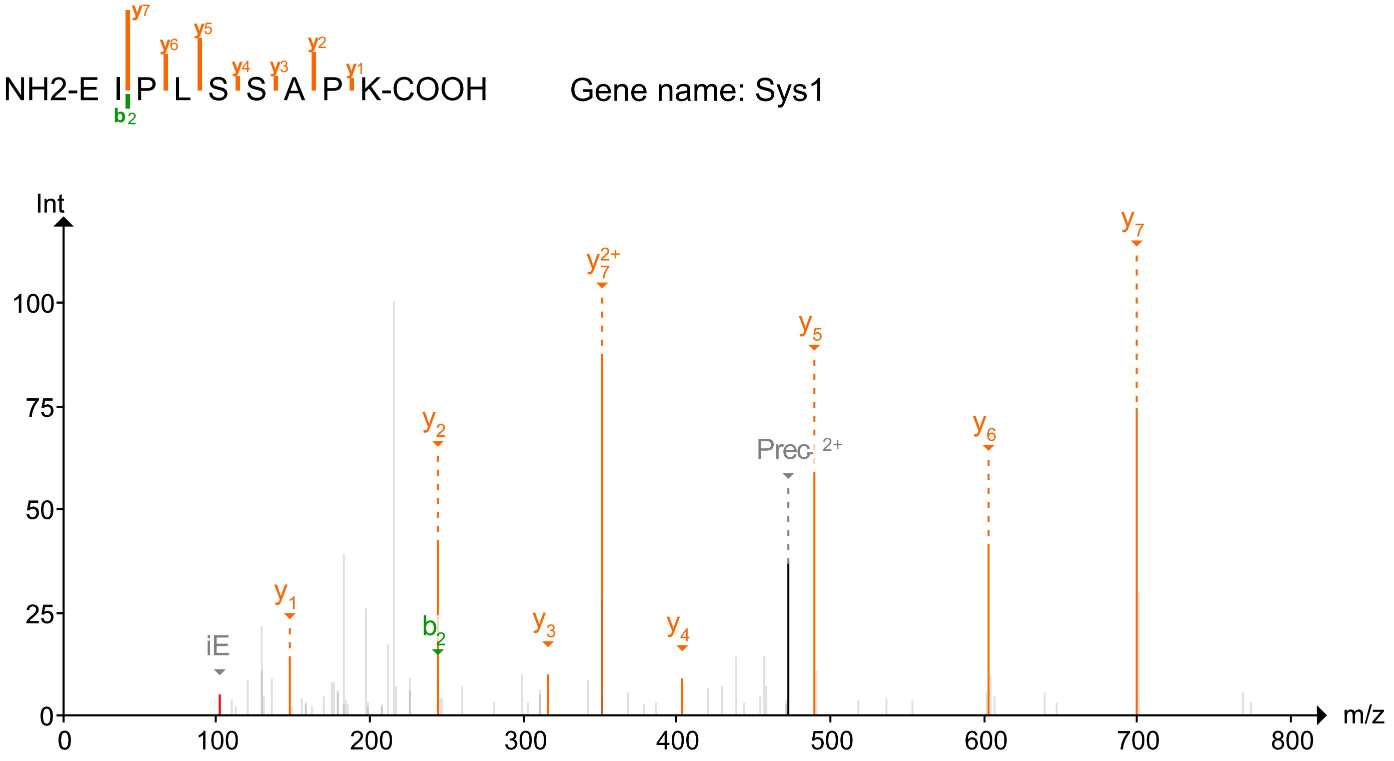

NH2-E I P L S S A P K-COOH
y7 y6 y5 y4 y3 y2 y1
b2
Gene name: Sys1
Int
m/z
100
75
50
25
0
0 100 200 300 400 500 600 700 800
y7
y7 2+
y5
y2
Prec 2+
y6
y1
b2
y3
y4
iE

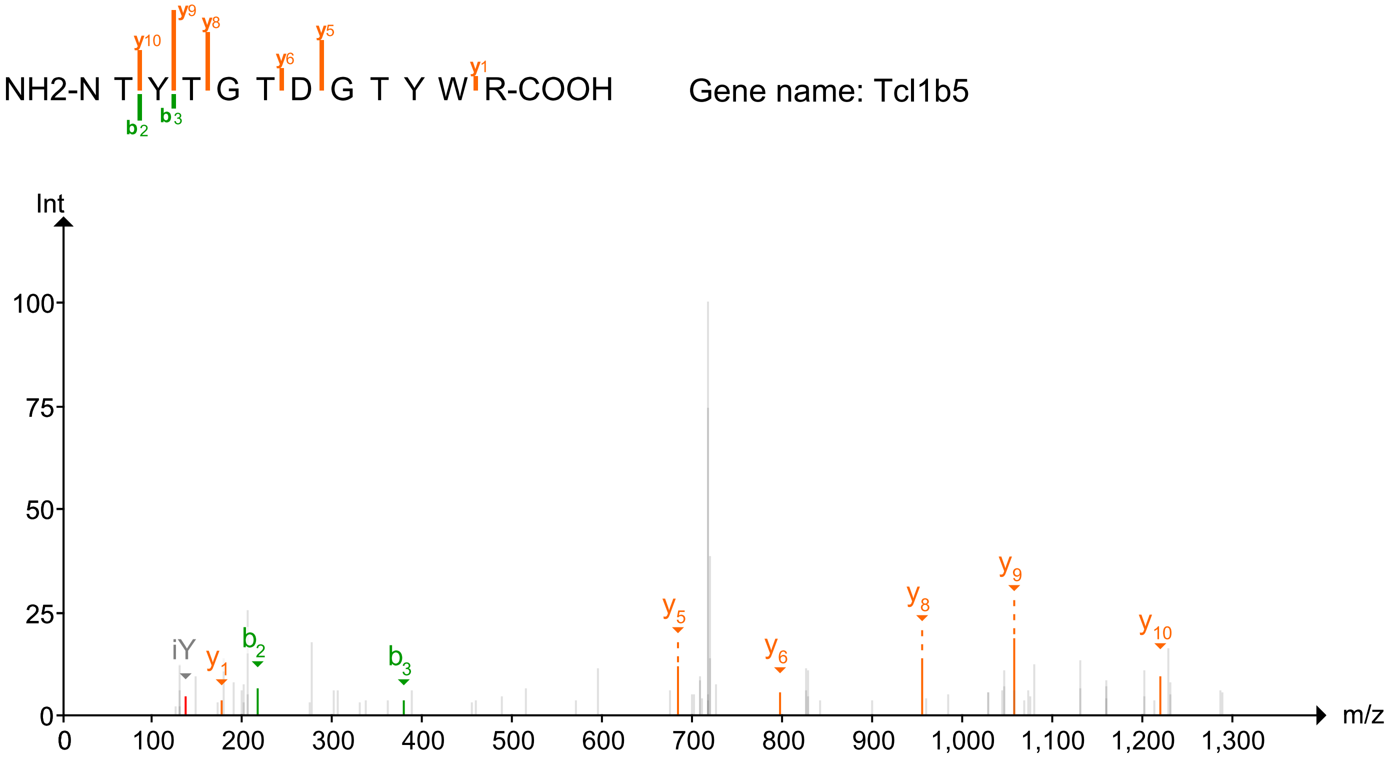
NH2-N T Y T G T D G T Y W R-COOH
y10 y9 y8 y6 y5 y1
b2 b3
Gene name: Tcl1b5
Int
100
75
50
25
0
m/z
0 100 200 300 400 500 600 700 800 900 1,000 1,100 1,200 1,300
iY
y1
b2
b3
y5
y6
y8
y9
y10

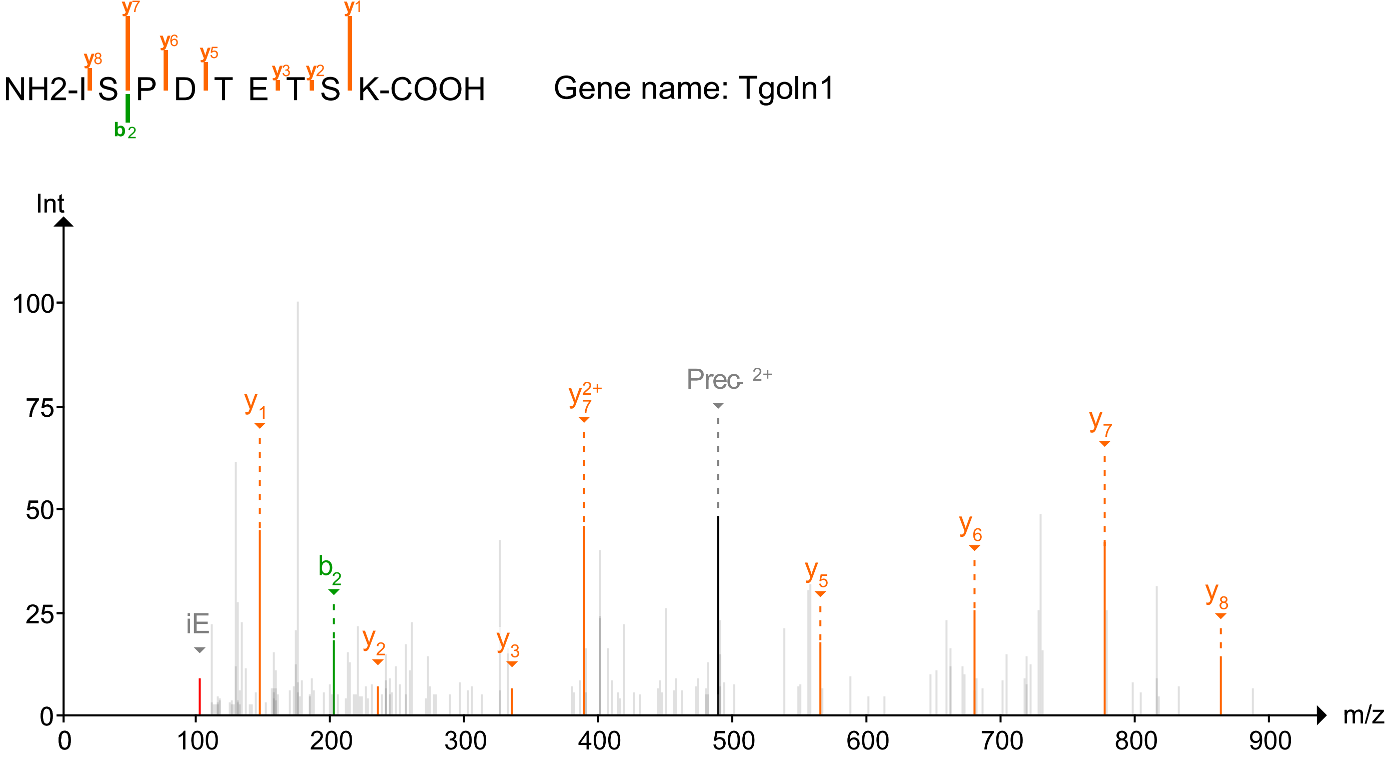

NH2-I S P D T E T S K-COOH
y8 y7 y6 y5 y3 y2 y1
b2
Gene name: Tgoln1
Int
m/z
y1 y2 y3 y5 y6 y7 y8
y7 2+
b2
iE
Prec 2+
0 25 50 75 100
0 100 200 300 400 500 600 700 800 900

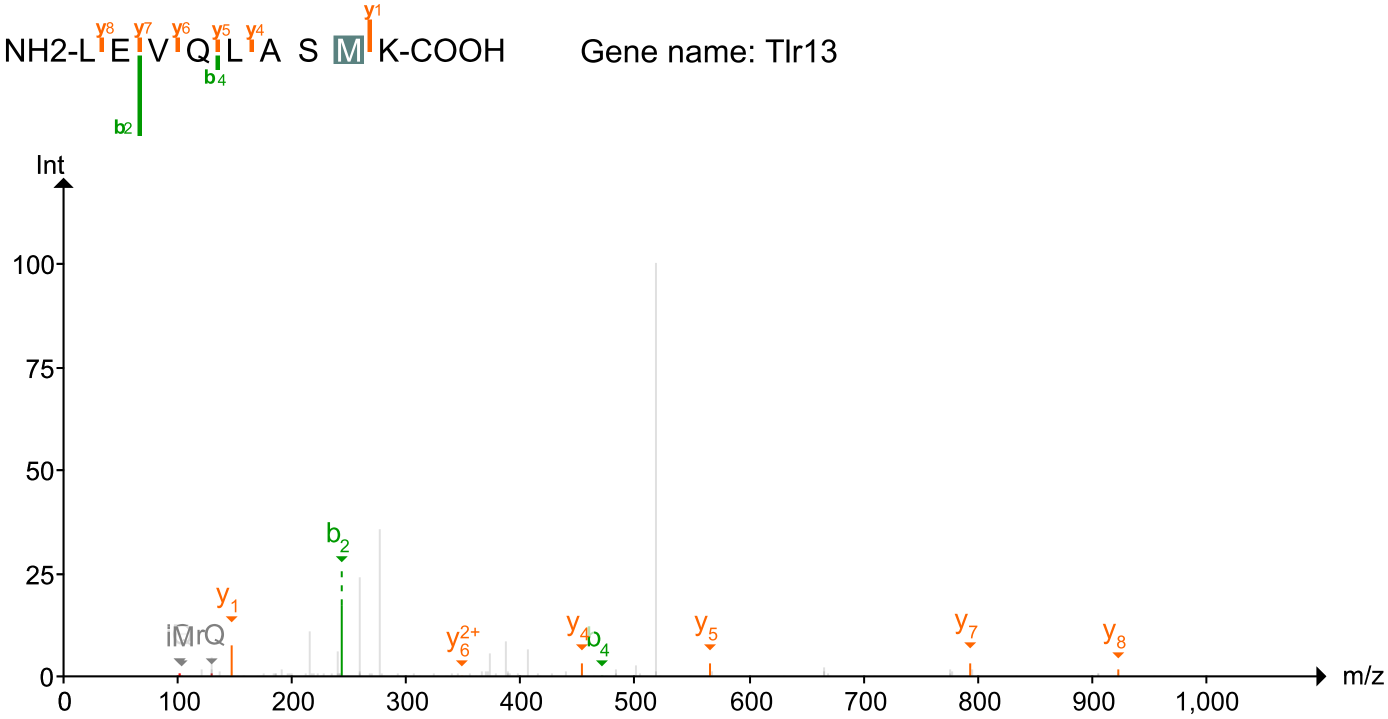

NH2-L E V Q L A S M K-COOH
Gene name: Tlr13
y8
y7
y6
y5
y4
y1
b2
b4
Int
100
75
50
25
0
m/z
0
100
200
300
400
500
600
700
800
900
1,000
b2
y1
iM
iC
rQ
y6 2+
y4
b4
y5
y7
y8

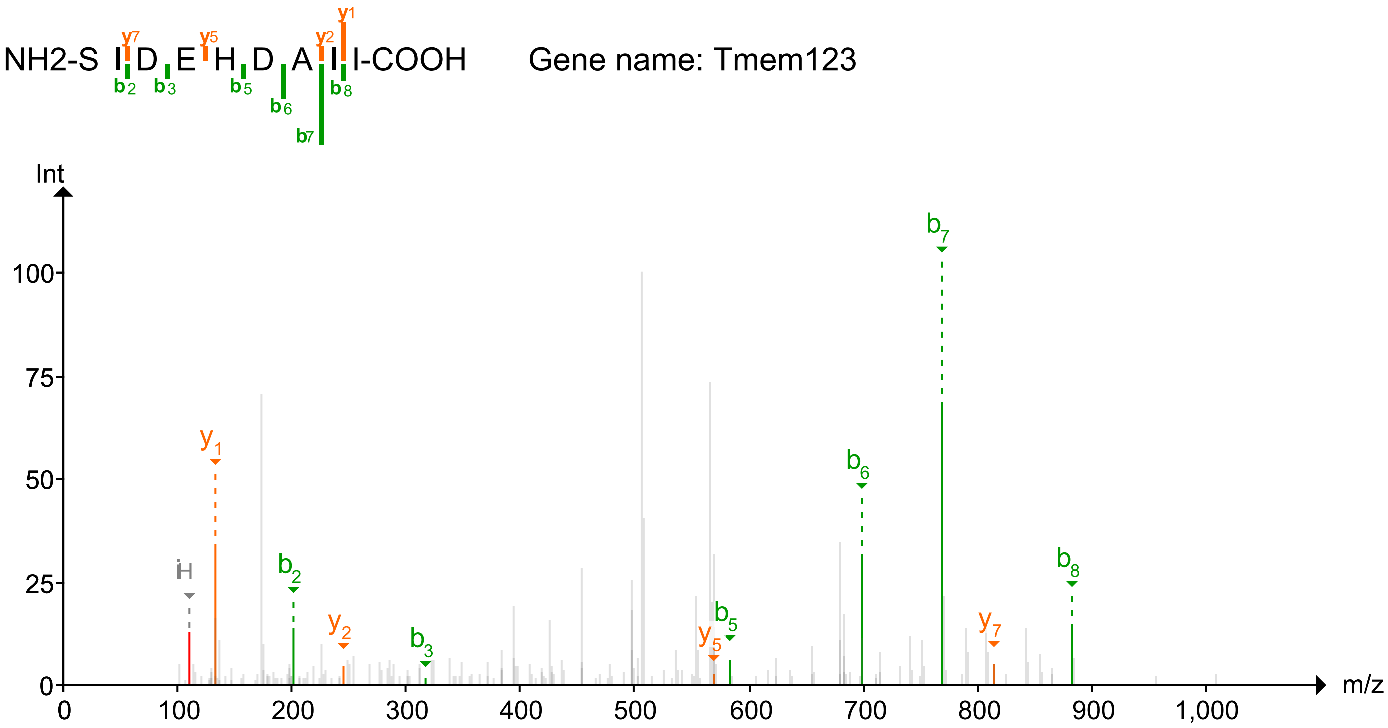

NH2-S I D E H D A I I-COOH
Gene name: Tmem123
Int
m/z
y1
b2
y2
b3
y5
b5
b6
b7
y7
b8
H

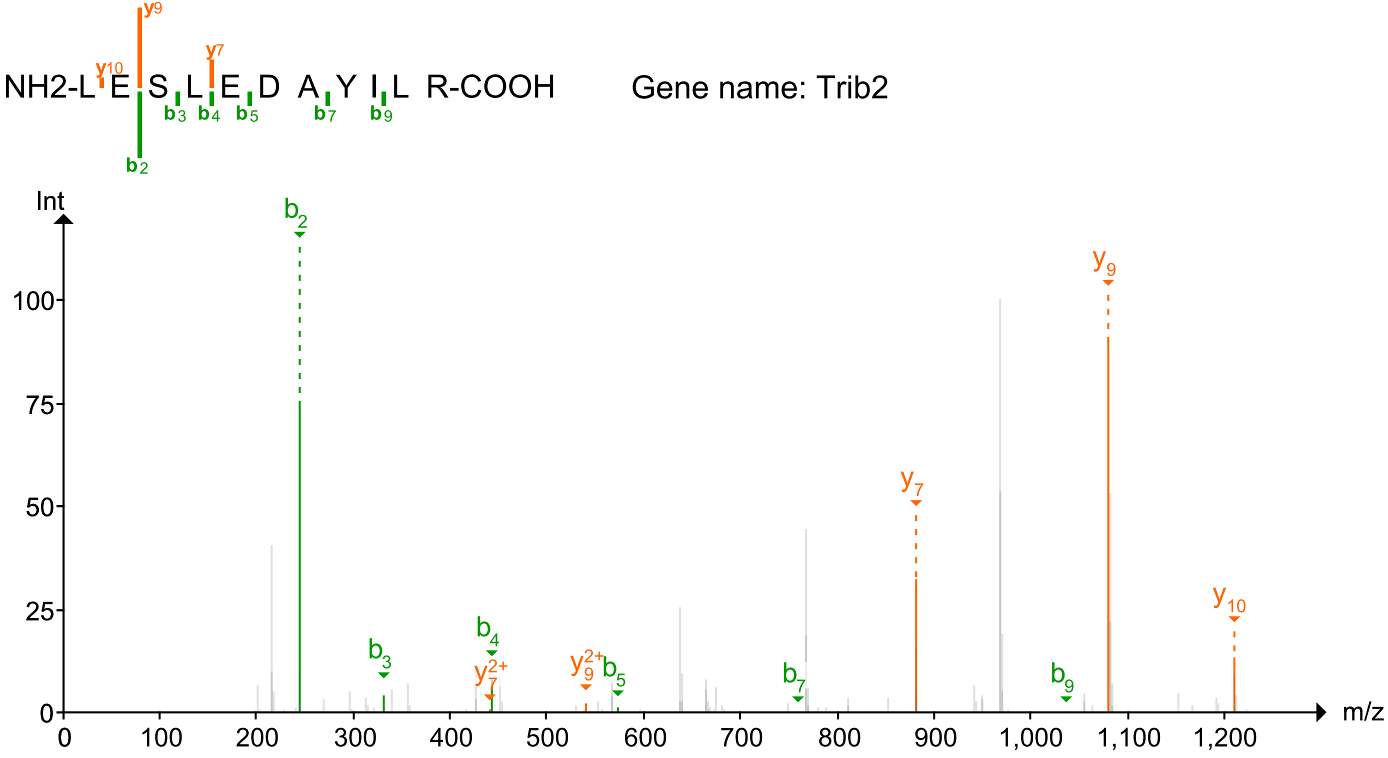

NH2-L E S L E D A Y I L R-COOH
y10
y9
y7
b2
b3
b4
b5
b7
b9
Gene name: Trib2
Int
m/z
0
25
50
75
100
0
100
200
300
400
500
600
700
800
900
1,000
1,100
1,200
b2
b3
b4
y7 2+
y9 2+
b5
b7
y7
b9
y9
y10

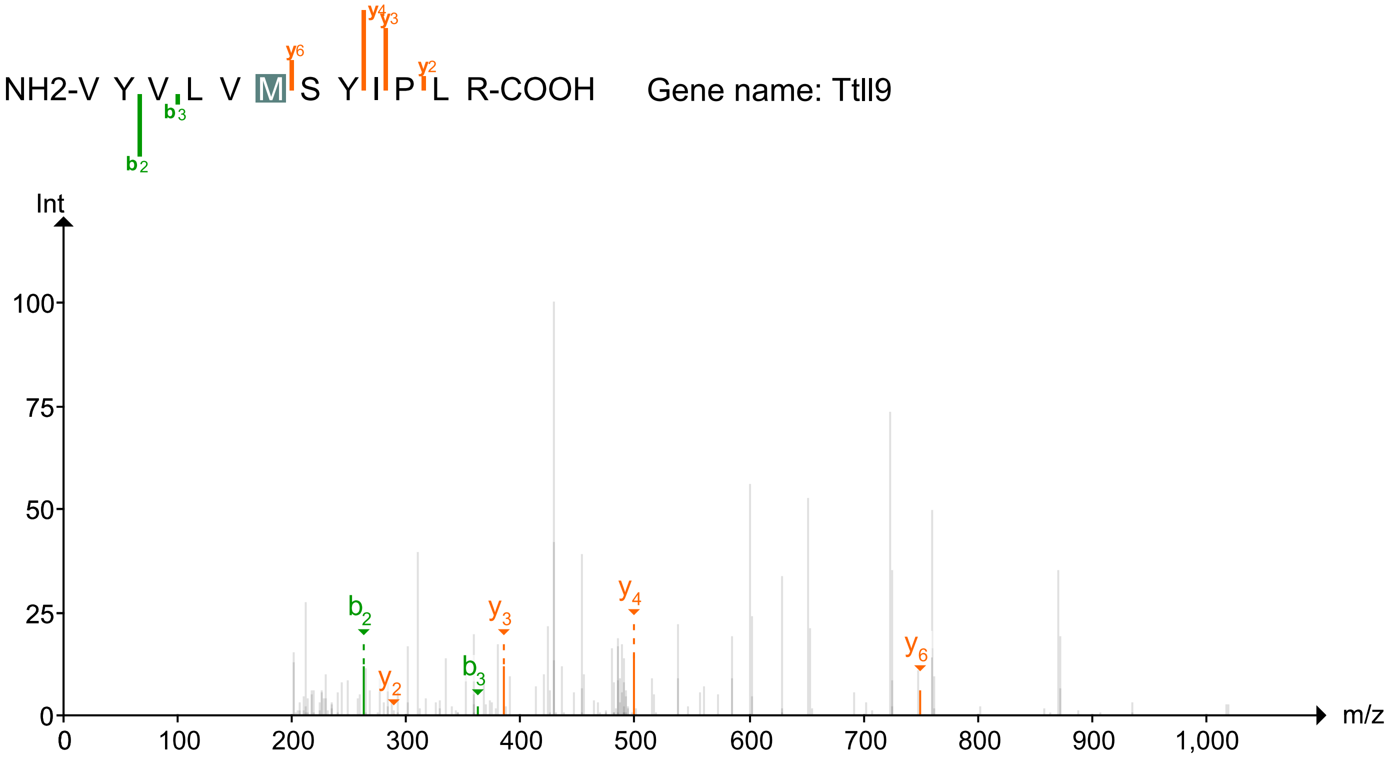
NH2-V Y V L V M S Y I P L R-COOH
Gene name: Ttll9
b2
b3
y6
y4
y3
y2
Int
m/z
0
25
50
75
100
0
100
200
300
400
500
600
700
800
900
1,000

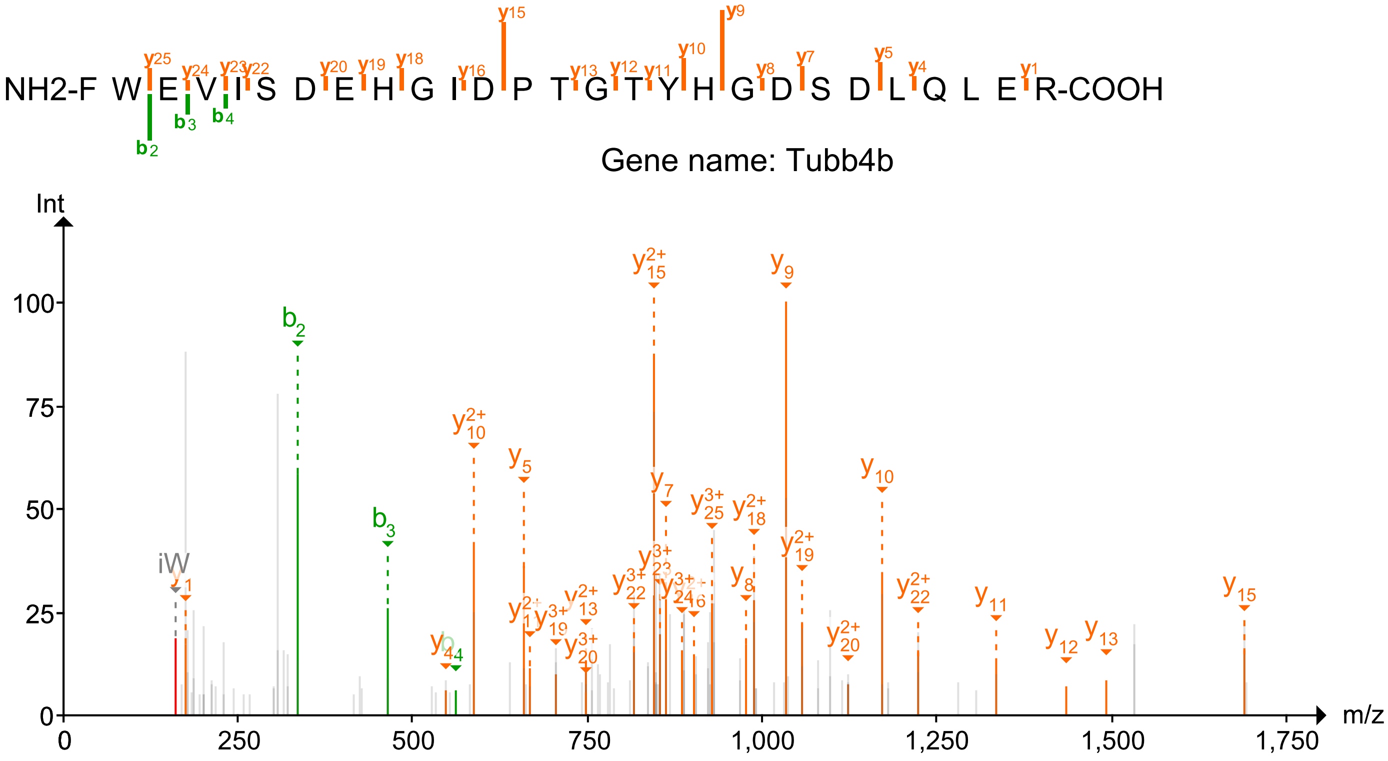

NH2-F W E V I S D E H G I D P T G T Y H G D S D L Q L E R-COOH
Gene name: Tubb4b
Int
m/z
0
25
50
75
100
250
500
750
1,000
1,250
1,500
1,750
b2
b3
b4
iW
y1
y5
y7
y8
y9
y10
y11
y12
y13
y15

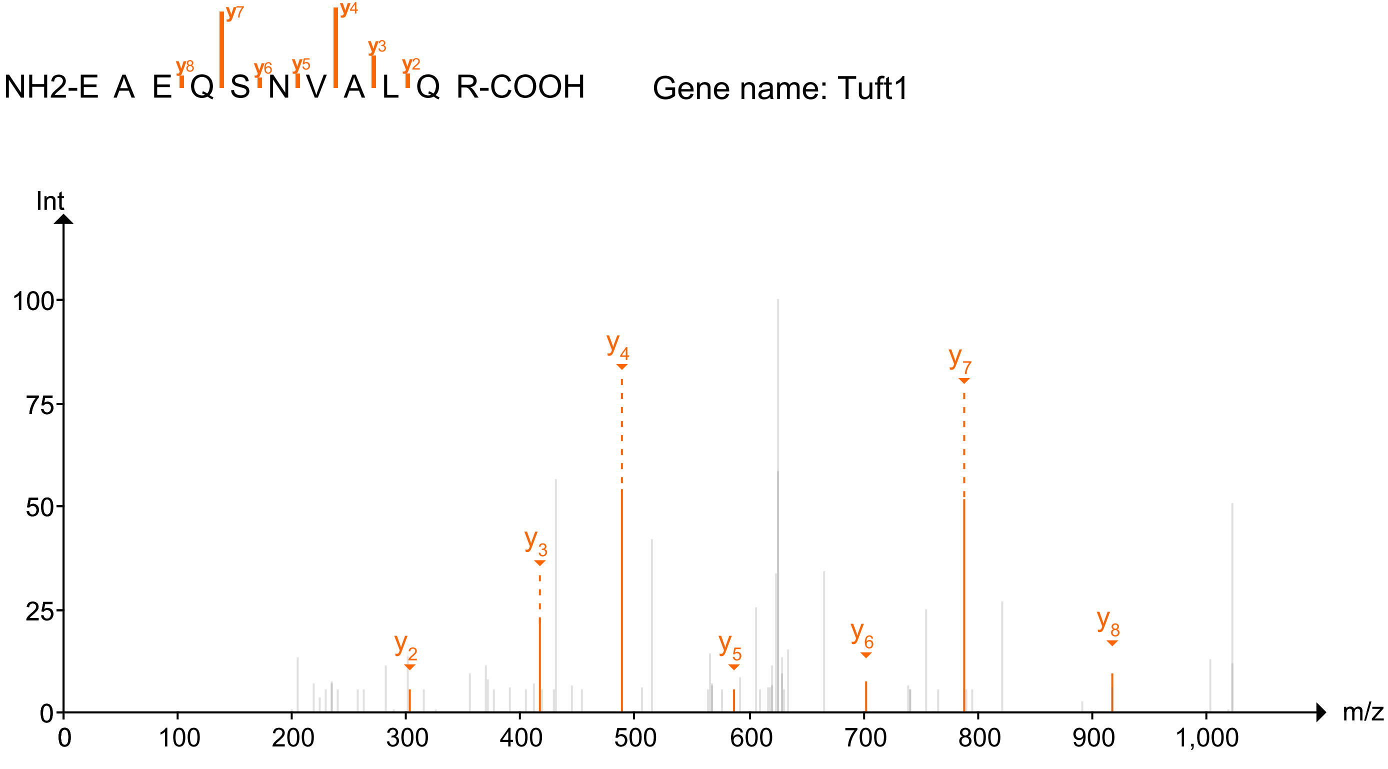

NH2-E A E Q S N V A L Q R-COOH
y8 y7 y6 y5 y4 y3 y2
Gene name: Tuft1
Int
100
75
50
25
0
y4
y7
y3
y2
y5
y6
y8
0
100
200
300
400
500
600
700
800
900
1,000
m/z

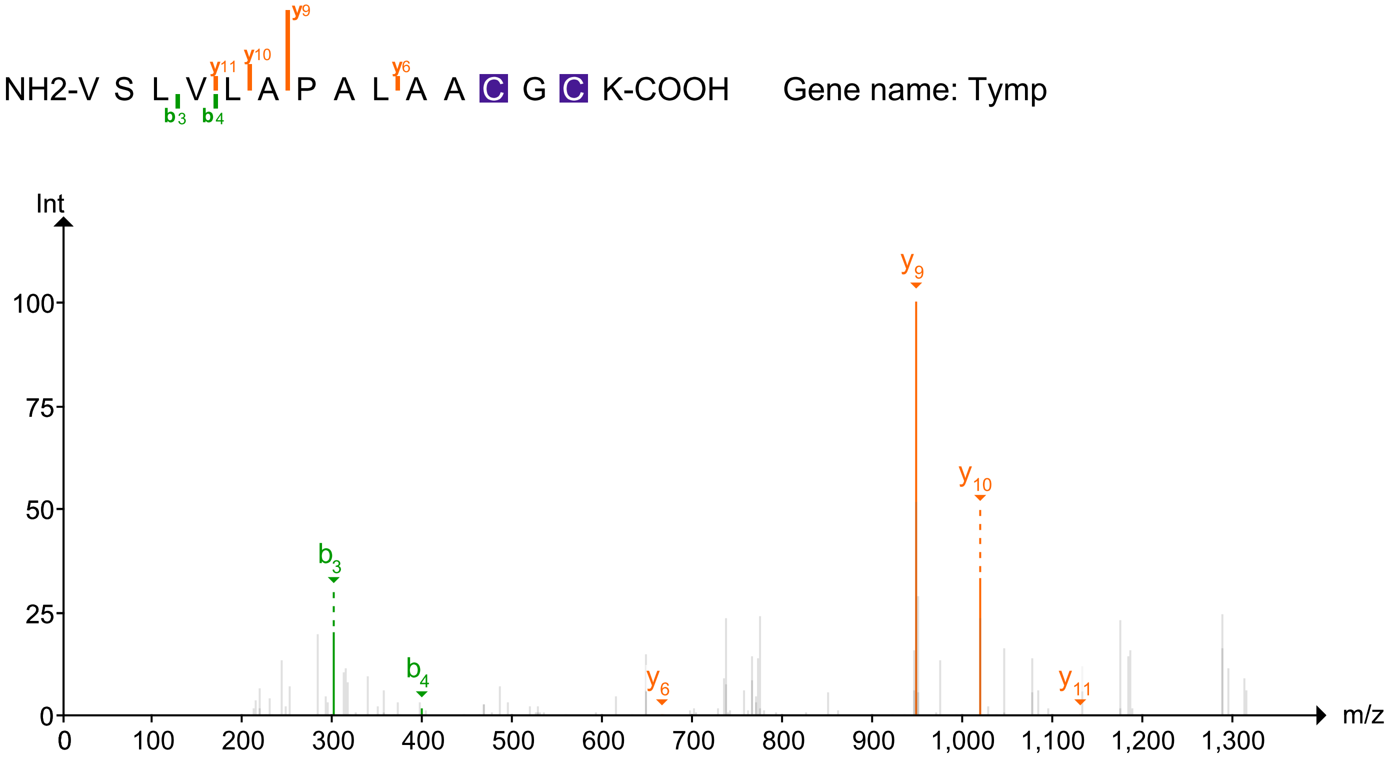
NH2-V S L V L A P A L A A C G C K-COOH
Gene name: Tymp
b3
b4
y11
y10
y9
y6
Int
m/z
y9
y10
b3
b4
y6
y11
0
25
50
75
100
0
100
200
300
400
500
600
700
800
900
1,000
1,100
1,200
1,300

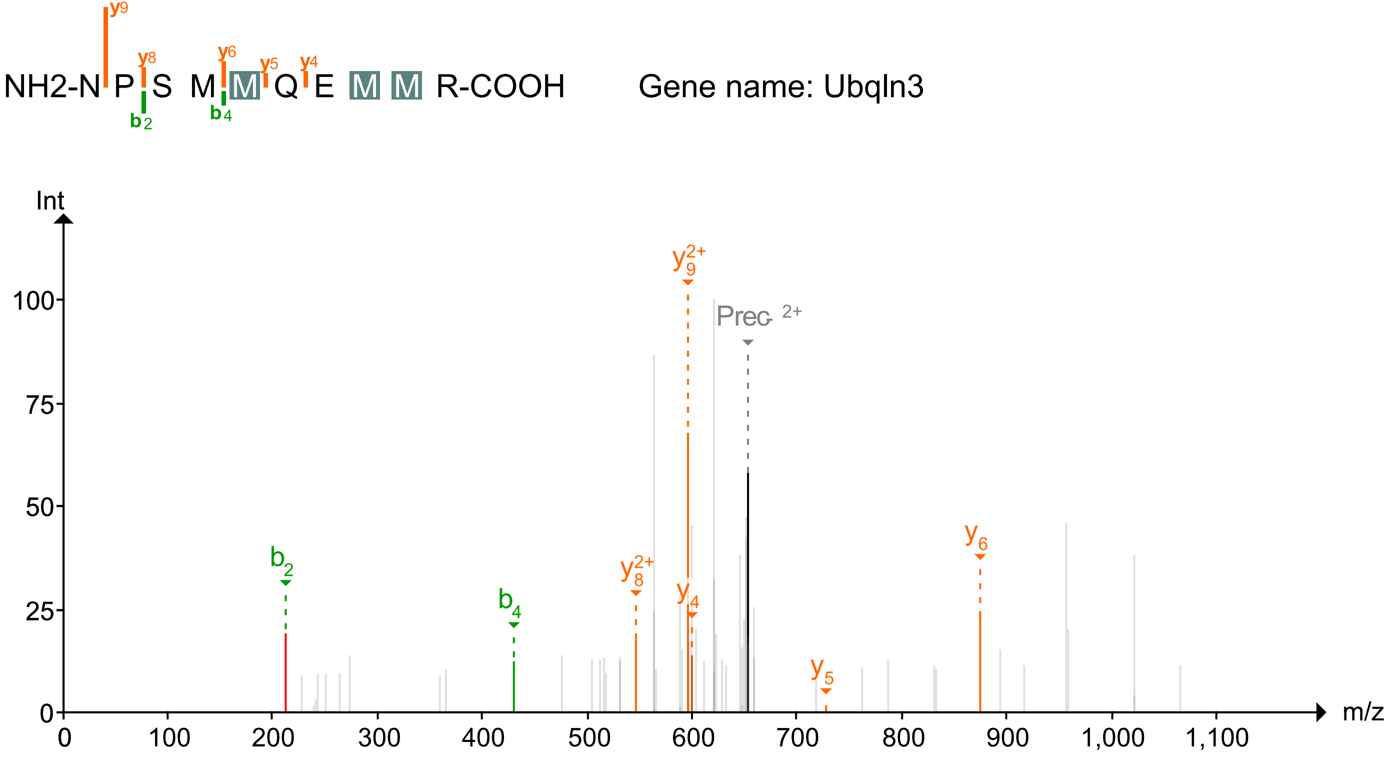

NH2-N P S M M Q E M M R-COOH
y9
y8
y6
y5
y4
b2
b4
Gene name: Ubqln3
Int
100
75
50
25
0
m/z
0
100
200
300
400
500
600
700
800
900
1,000
1,100
b2
b4
y8 2+
y9 2+
y4
y5
y6
Prec 2+

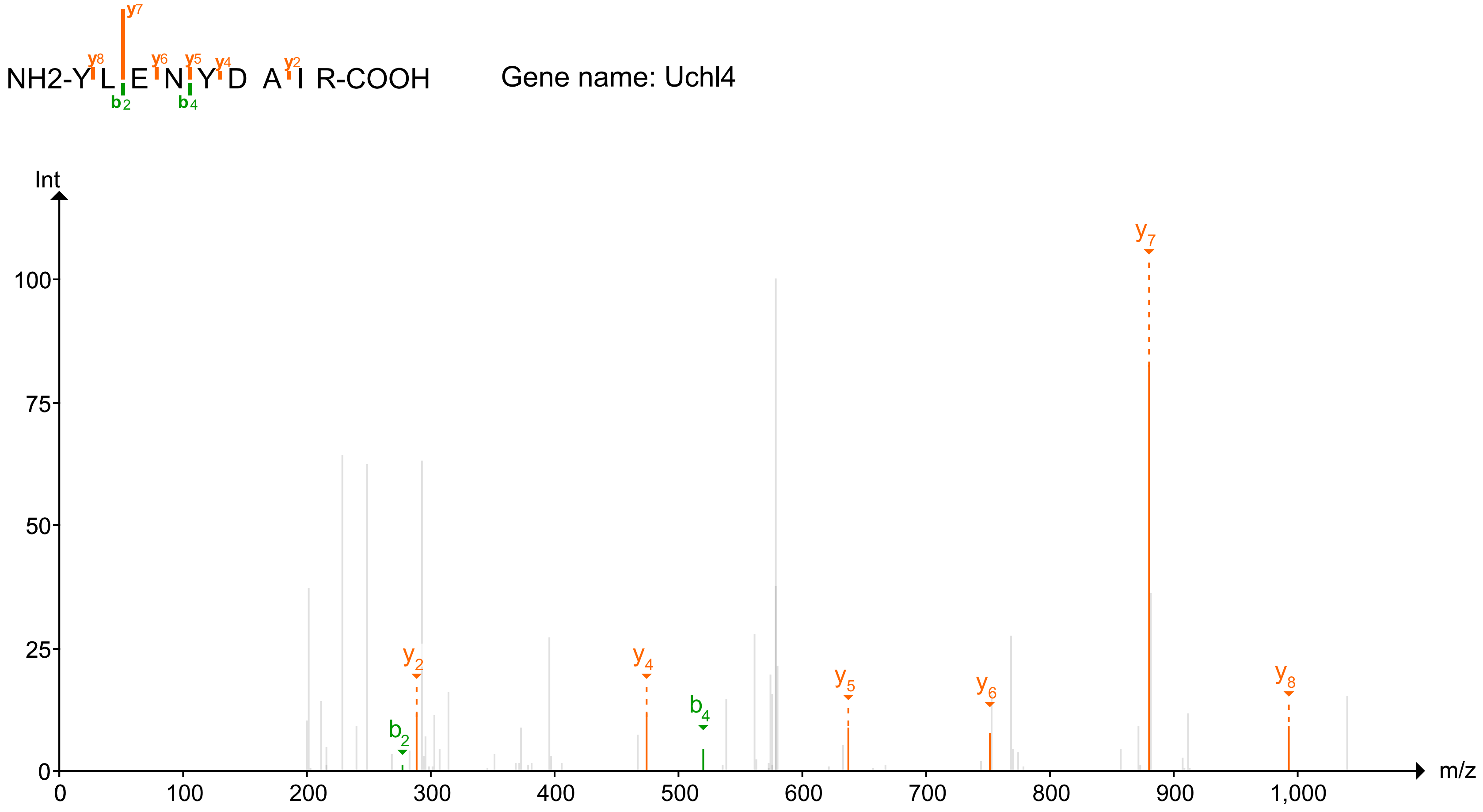

NH2-YLENYDAIR-COOH
Gene name: Uchl4
y8 y7 y6 y5 y4 y2
b2 b4
Int
100
75
50
25
0
m/z
0
100
200
300
400
500
600
700
800
900
1,000
y7
y2
y4
b4
y5
y6
y8
b2

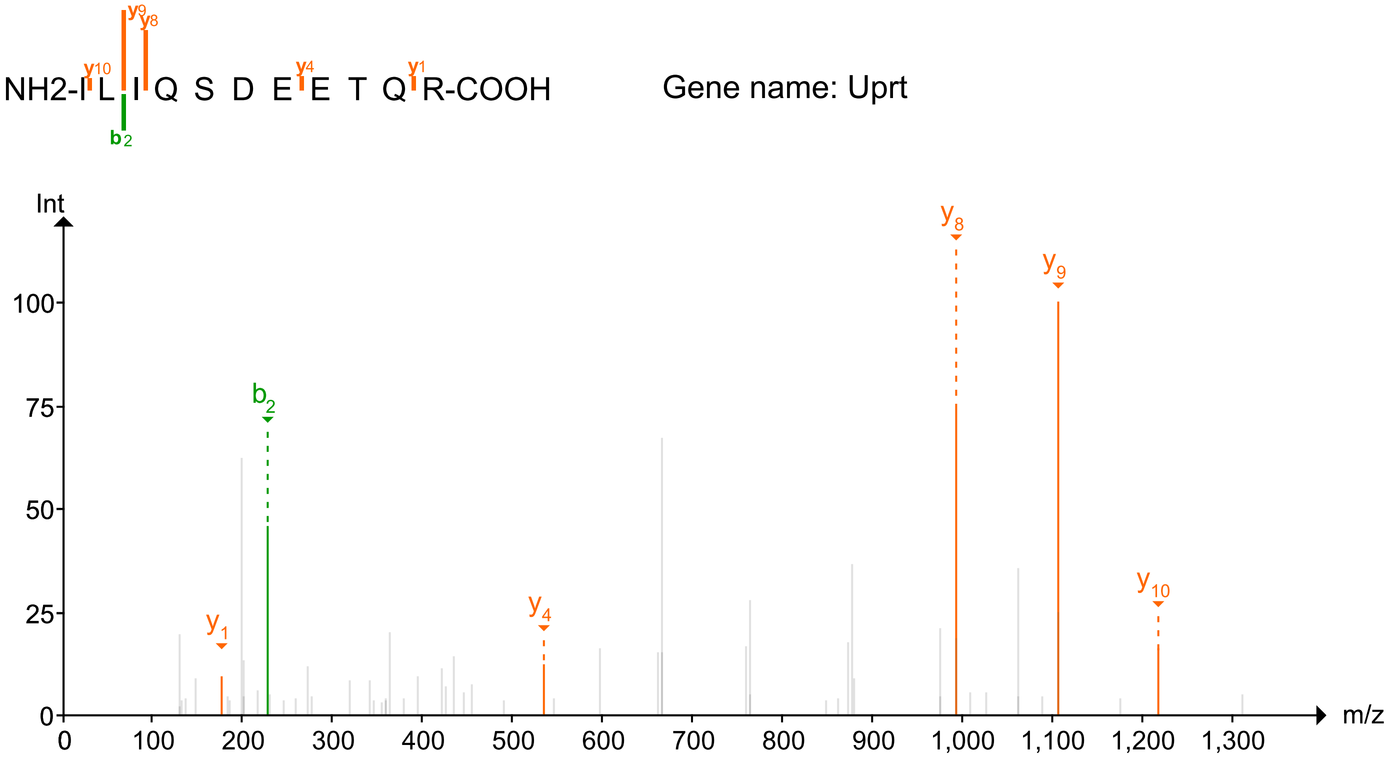

NH2-I L I Q S D E E T Q R-COOH
y10
y9
y8
y4
y1
b2
Gene name: Uprt
Int
100
75
50
25
0
m/z
0
100
200
300
400
500
600
700
800
900
1,000
1,100
1,200
1,300
y8
y9
b2
y1
y4
y10

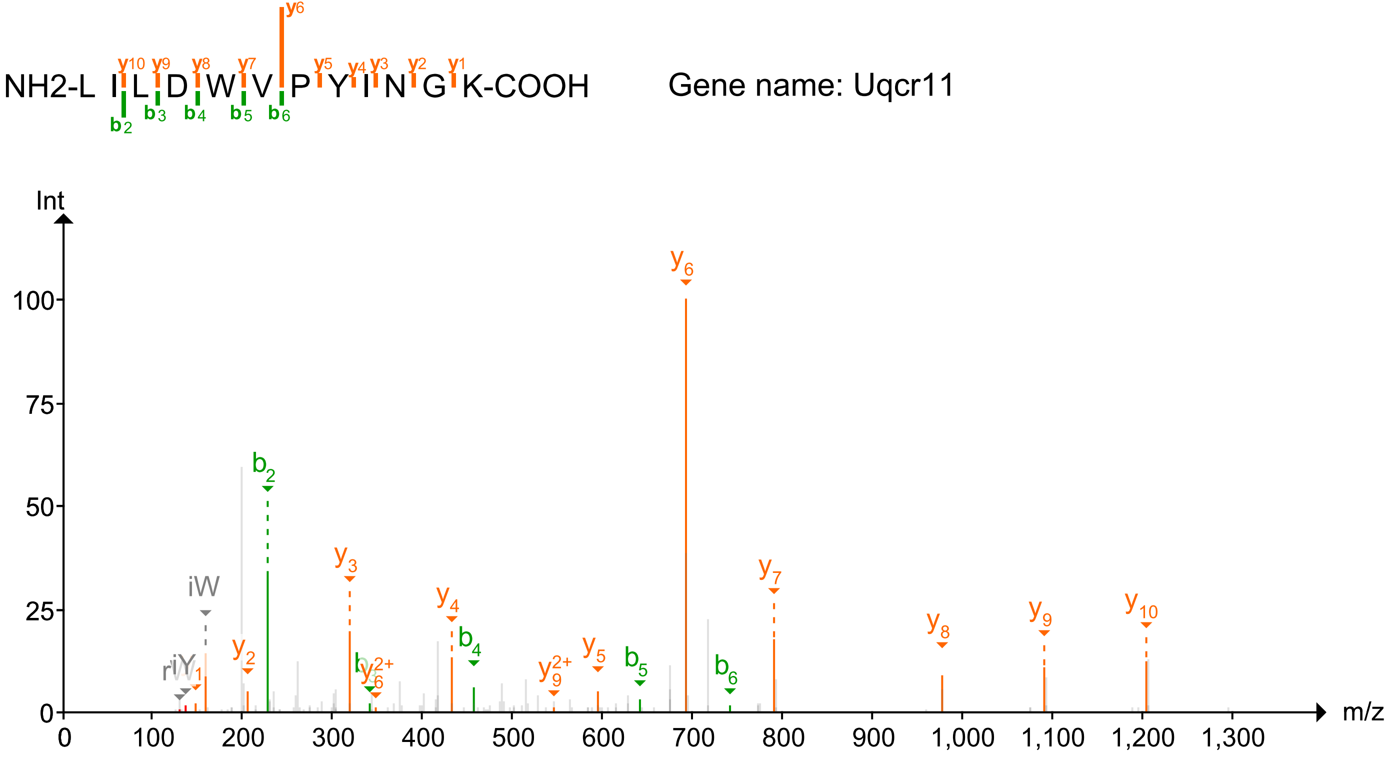

NH2-L I L D W V P Y I N G K-COOH
y10 y9 y8 y7 y6 y5 y4 y3 y2 y1
b2 b3 b4 b5 b6
Gene name: Uqcr11
Int
m/z
iW
iY
b2
y6
y3
y7
y4
b4
y2
y8
y9
y10
y5
b5
b6
y9 2+
y6 2+
y1

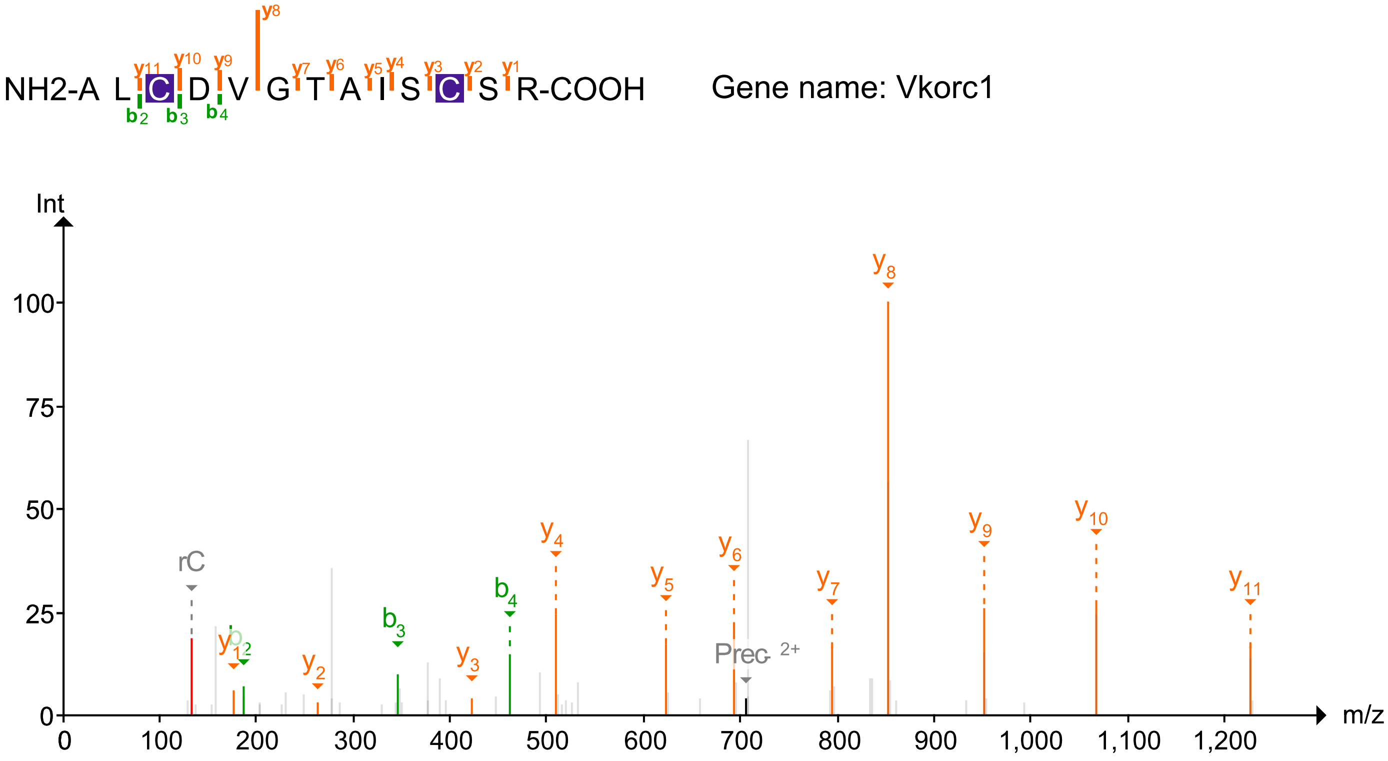

NH2-A L C D V G T A I S C S R-COOH
Gene name: Vkorc1
y11 y10 y9 y8 y7 y6 y5 y4 y3 y2 y1
b2 b3 b4
Int
100
75
50
25
0
rC
y1
b2
y2
b3
y3
b4
y4
y5
y6
Prec 2+
y7
y8
y9
y10
y11
0 100 200 300 400 500 600 700 800 900 1,000 1,100 1,200
m/z

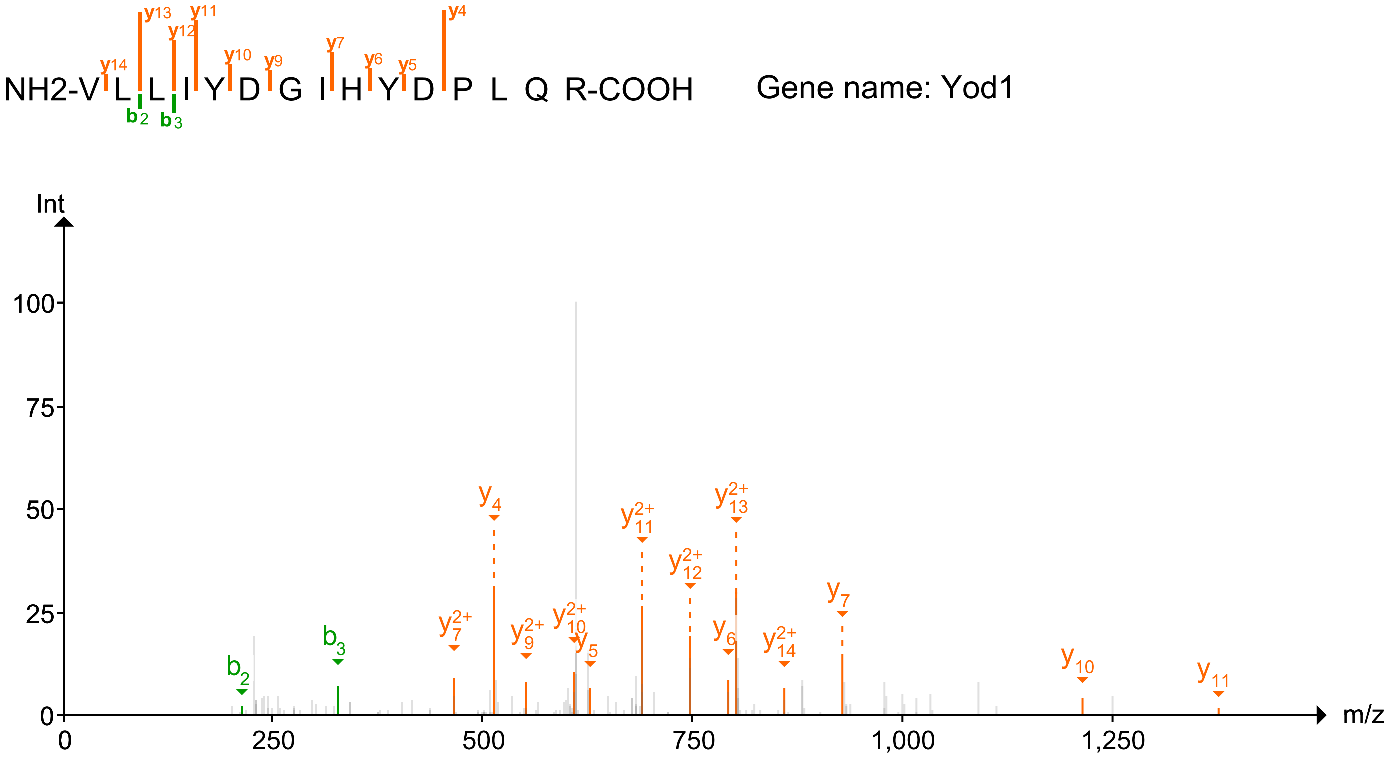
NH2-V L L I Y D G I H Y D P L Q R-COOH
Gene name: Yod1
y14 y13 y12 y11 y10 y9 y7 y6 y5 y4
b2 b3
Int
100
75
50
25
0
250
500
750
1,000
1,250
m/z
b2 b3 y7 2+ y4 y9 2+ y10 2+ y5 y11 2+ y12 2+ y6 y13 2+ y14 2+ y7 y10 y11

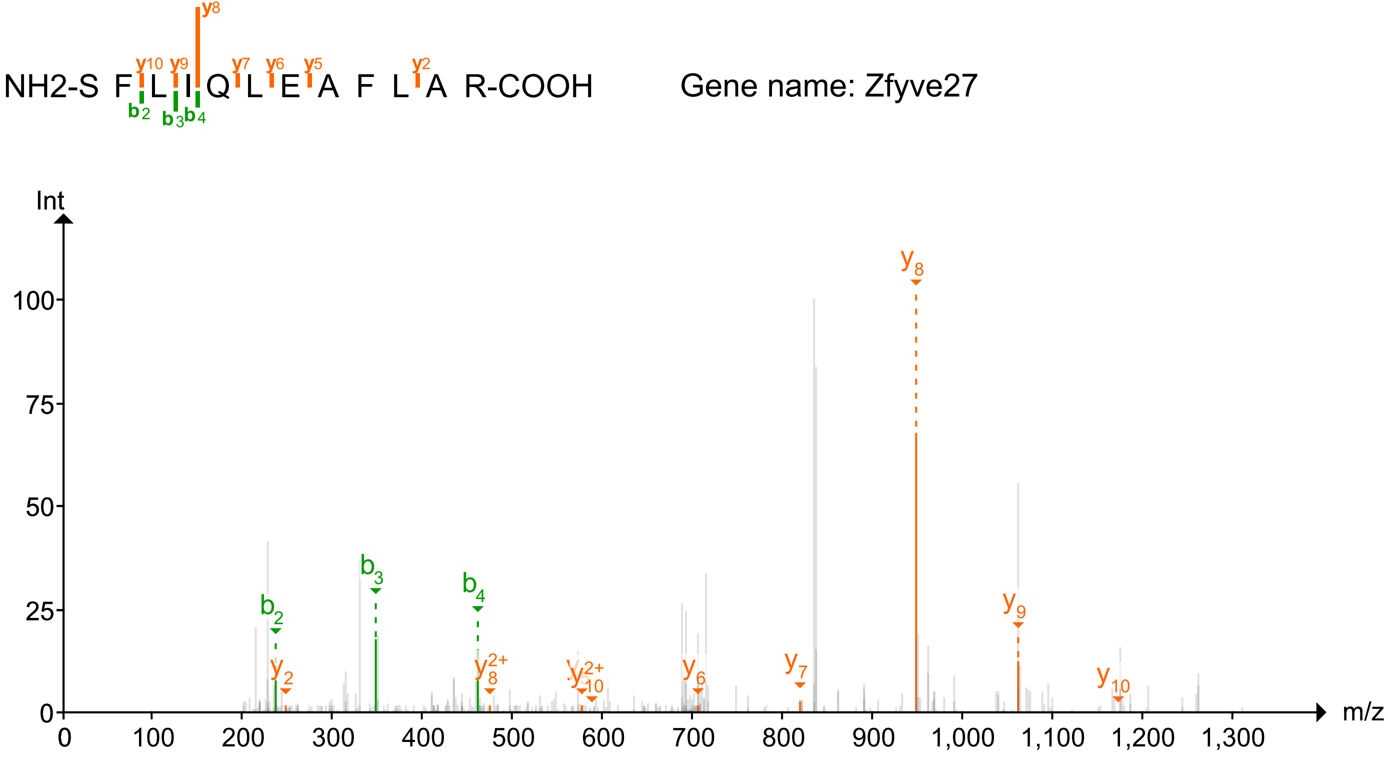
NH2-S F L I Q L E A F L A R-COOH
Gene name: Zfyve27
Int
m/z
100
75
50
25
0
0
100
200
300
400
500
600
700
800
900
1,000
1,100
1,200
1,300
y8
y10
y9
y7
y6
y5
y2
b2
b3
b4

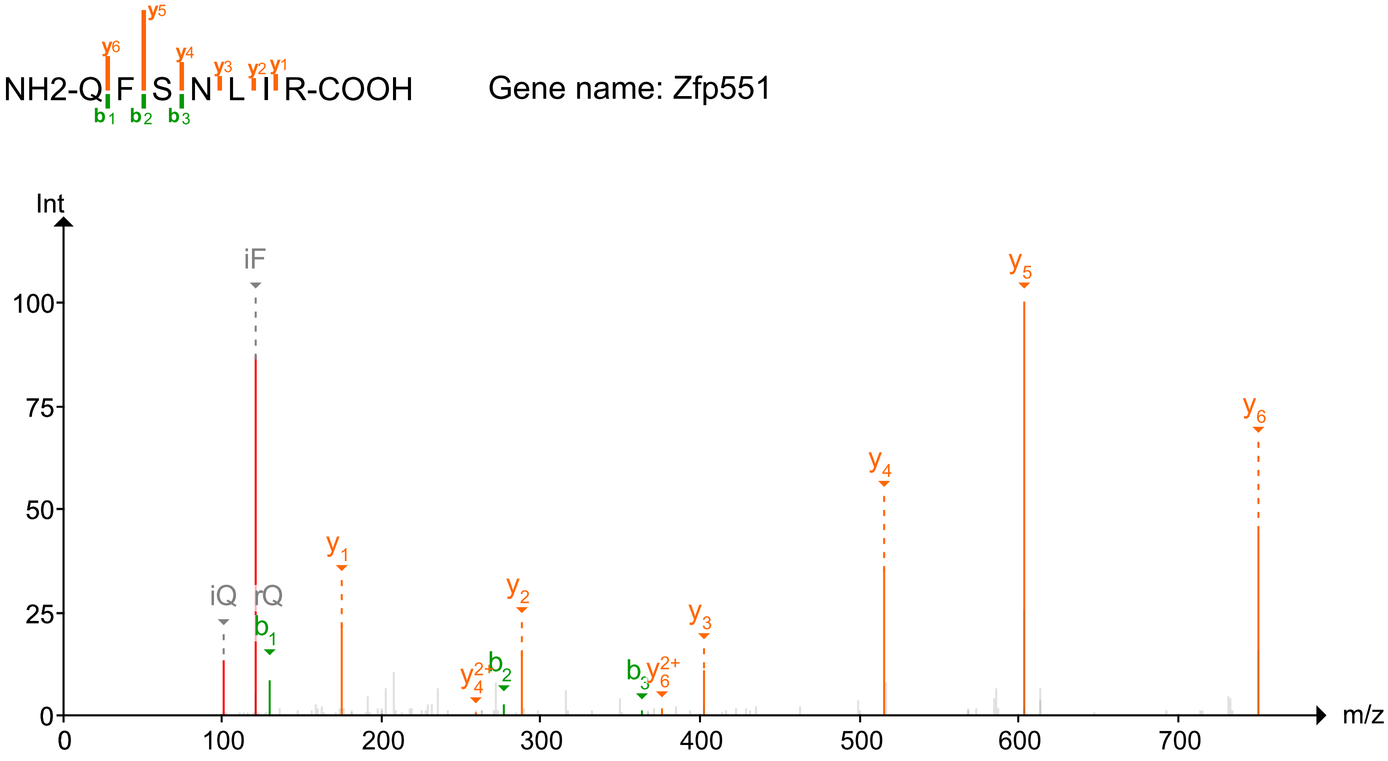

NH2-QFSNLIR-COOH
y6 y5 y4 y3 y2 y1
b1 b2 b3
Gene name: Zfp551
Int
100
75
50
25
0
iF
iQ
rQ
b1
y1
y2
y4 2+
b2
b3
y6 2+
y3
y4
y5
y6
0
100
200
300
400
500
600
700
m/z

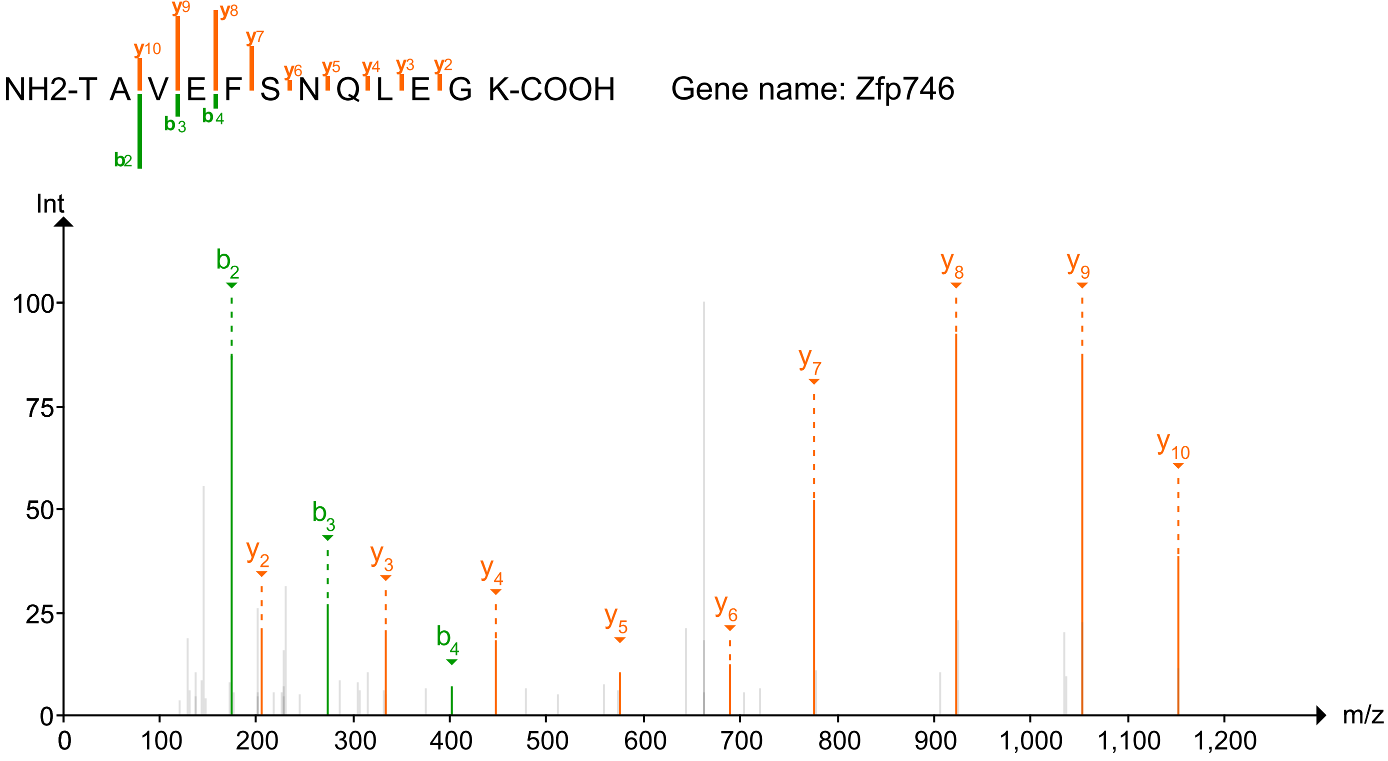

NH2-T A V E F S N Q L E G K-COOH
Gene name: Zfp746
y10
y9
y8
y7
y6
y5
y4
y3
y2
b2
b3
b4
Int
m/z
100
75
50
25
0
0
100
200
300
400
500
600
700
800
900
1,000
1,100
1,200
