# Supplementary material for: Single-Cell Multi-Omics Analysis of In Vitro Post-Ovulatory–Aged Oocytes Revealed Aging-Dependent Protein Degradation
Source: Mol Cell Proteomics. 2024 Nov 20;24(1):100882. doi: 10.1016/j.mcpro.2024.100882 (PMC11728983; doi:10.1016/j.mcpro.2024.100882)
Supplement: Supplementary Data 2 [file mmc3.pdf]

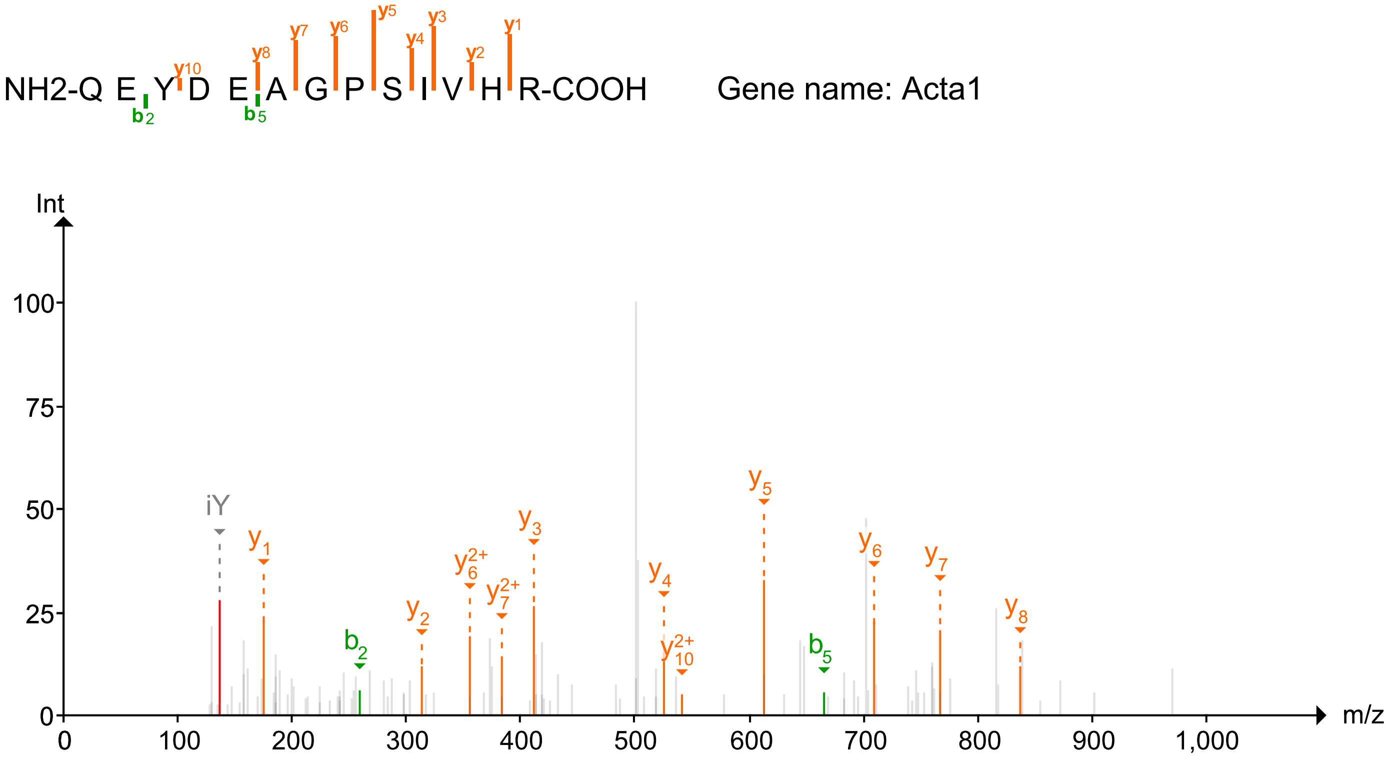

NH2-Q E Y D E A G P S I V H R-COOH
Gene name: Acta1
y10 y8 y7 y6 y5 y4 y3 y2 y1
b2 b5
Int
100
75
50
25
0
iY
y_1
b_2
y_2
y_6^{2+}
y_7^{2+}
y_3
y_4
y_{10}^{2+}
y_5
b_5
y_6
y_7
y_8
0 100 200 300 400 500 600 700 800 900 1,000
m/z

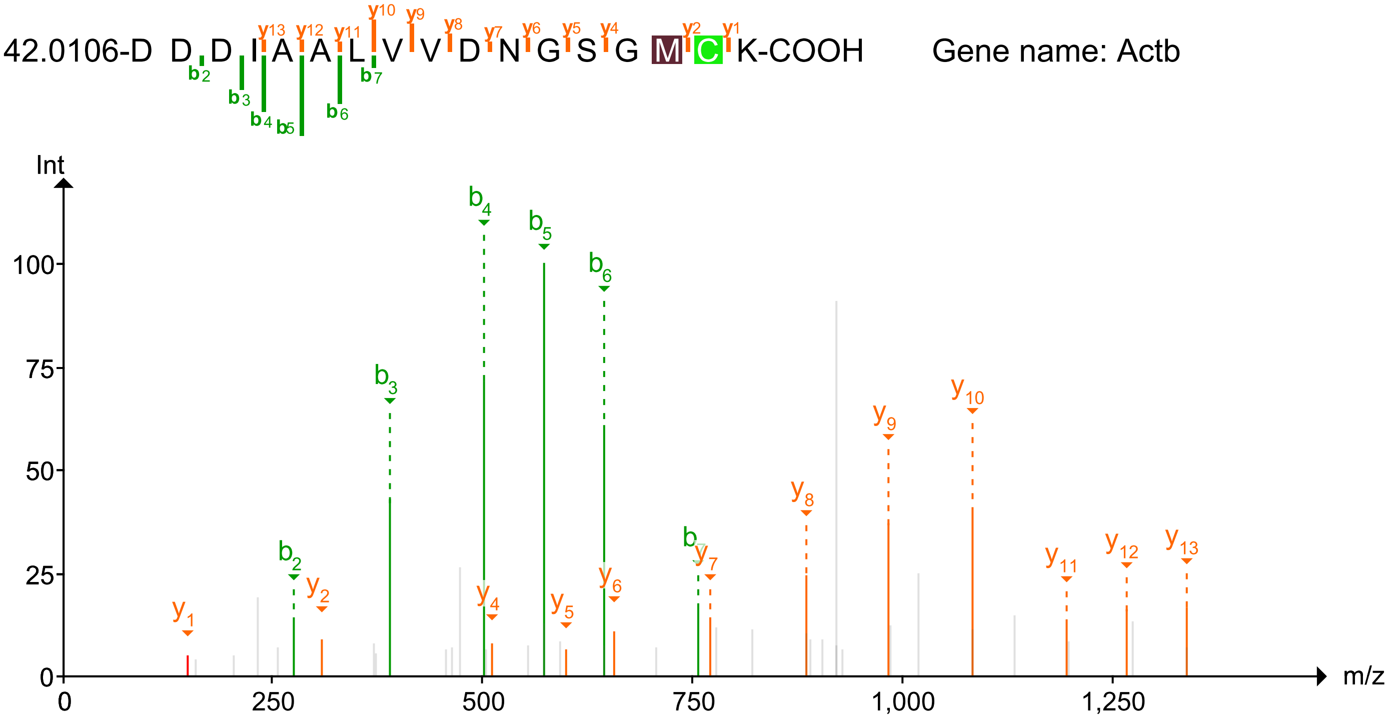

42.0106-D D D I A A L V V D N G S G M C K-COOH
Gene name: Actb
Int
m/z
0
25
50
75
100
250
500
750
1,000
1,250
b2
b3
b4
b5
b6
b7
y1
y2
y4
y5
y6
y7
y8
y9
y10
y11
y12
y13

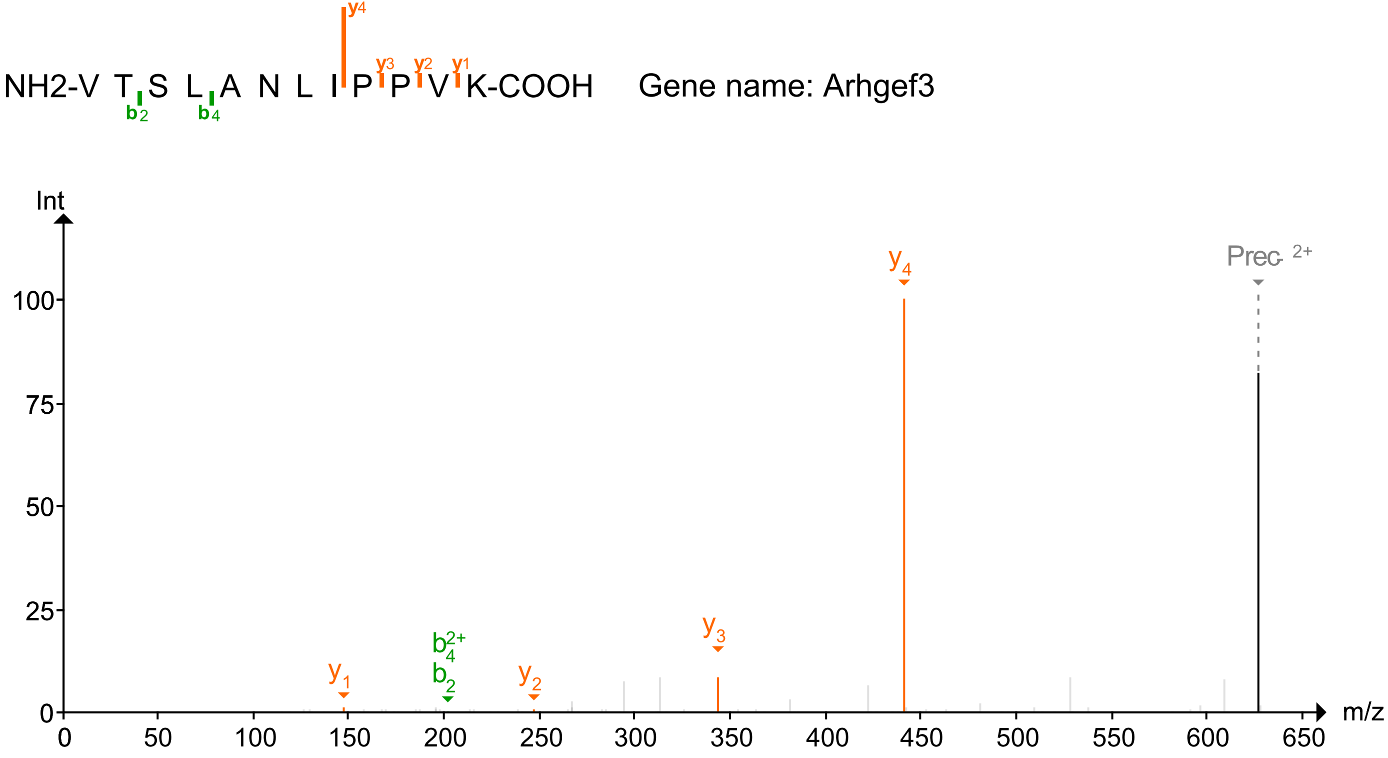

NH2-V T S L A N L I P P V K-COOH
Gene name: Arhgef3
b2
b4
y4
y3
y2
y1
Int
100
75
50
25
0
Prec 2+
y4
y3
y1
y2
b4 2+
b2
m/z
0
50
100
150
200
250
300
350
400
450
500
550
600
650

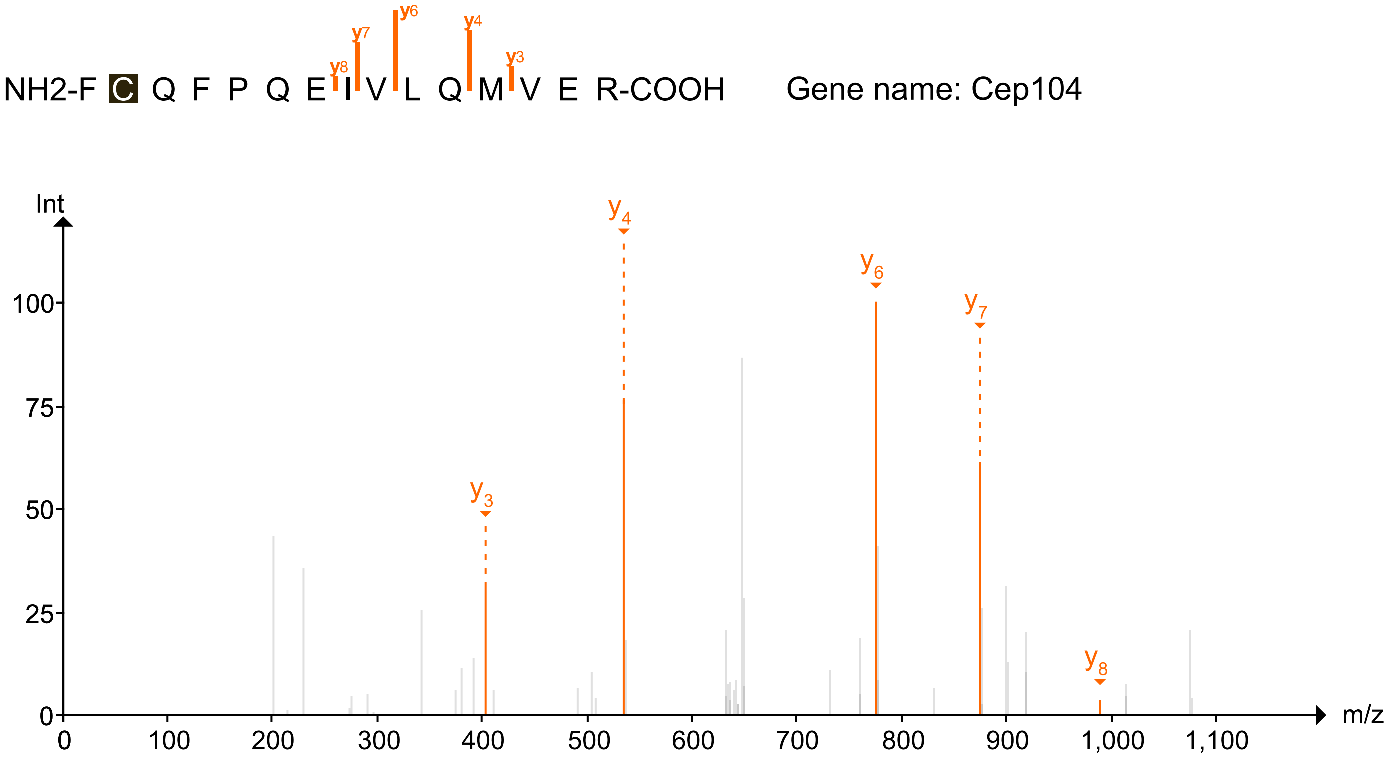

y8
y7
y6
y4
y3
NH2-F C Q F P Q E I V L Q M V E R-COOH
Gene name: Cep104
Int
y4
y6
y7
y3
y8
100
75
50
25
0
0
100
200
300
400
500
600
700
800
900
1,000
1,100
m/z

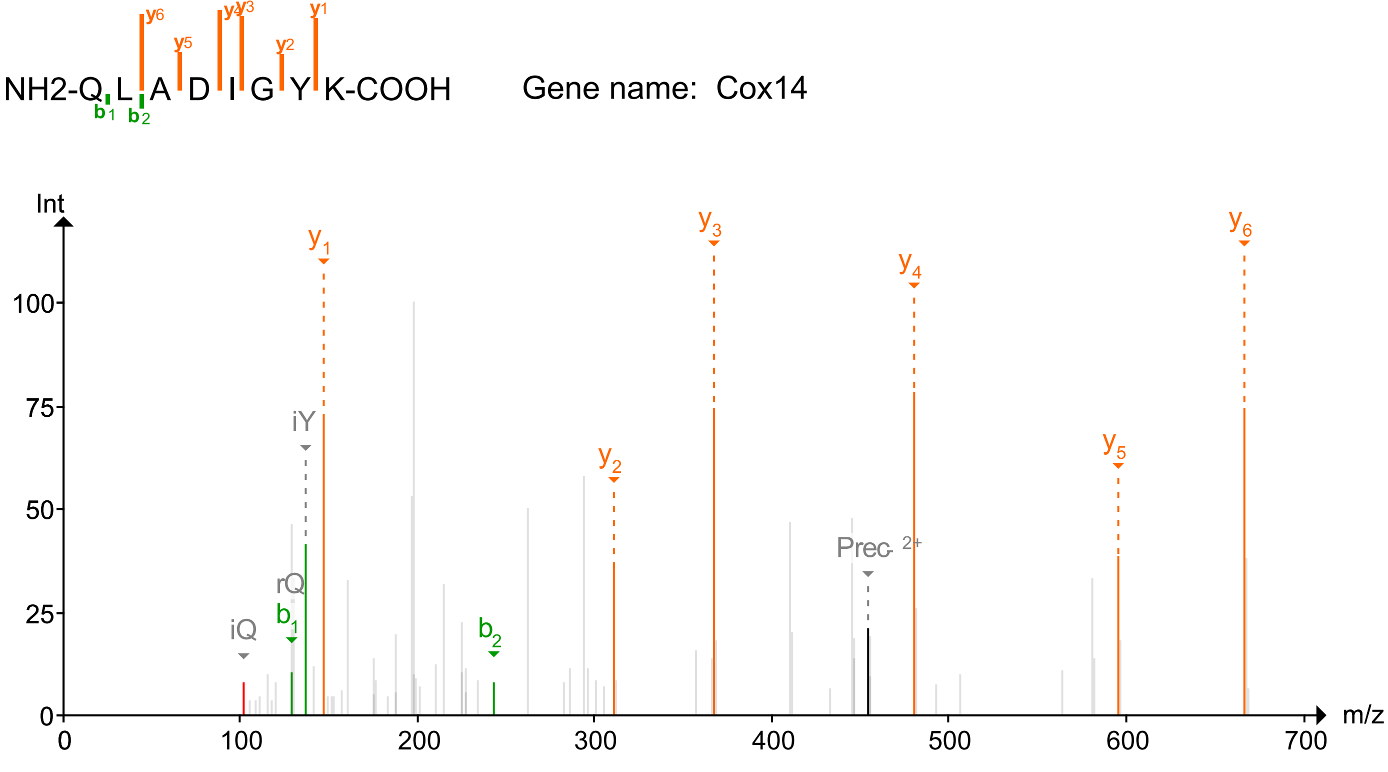

NH2-Q L A D I G Y K-COOH
Gene name: Cox14
b1
b2
y6
y5
y4
y3
y2
y1
Int
m/z
iQ
rQ
iY
Prec 2+
0
25
50
75
100
200
300
400
500
600
700

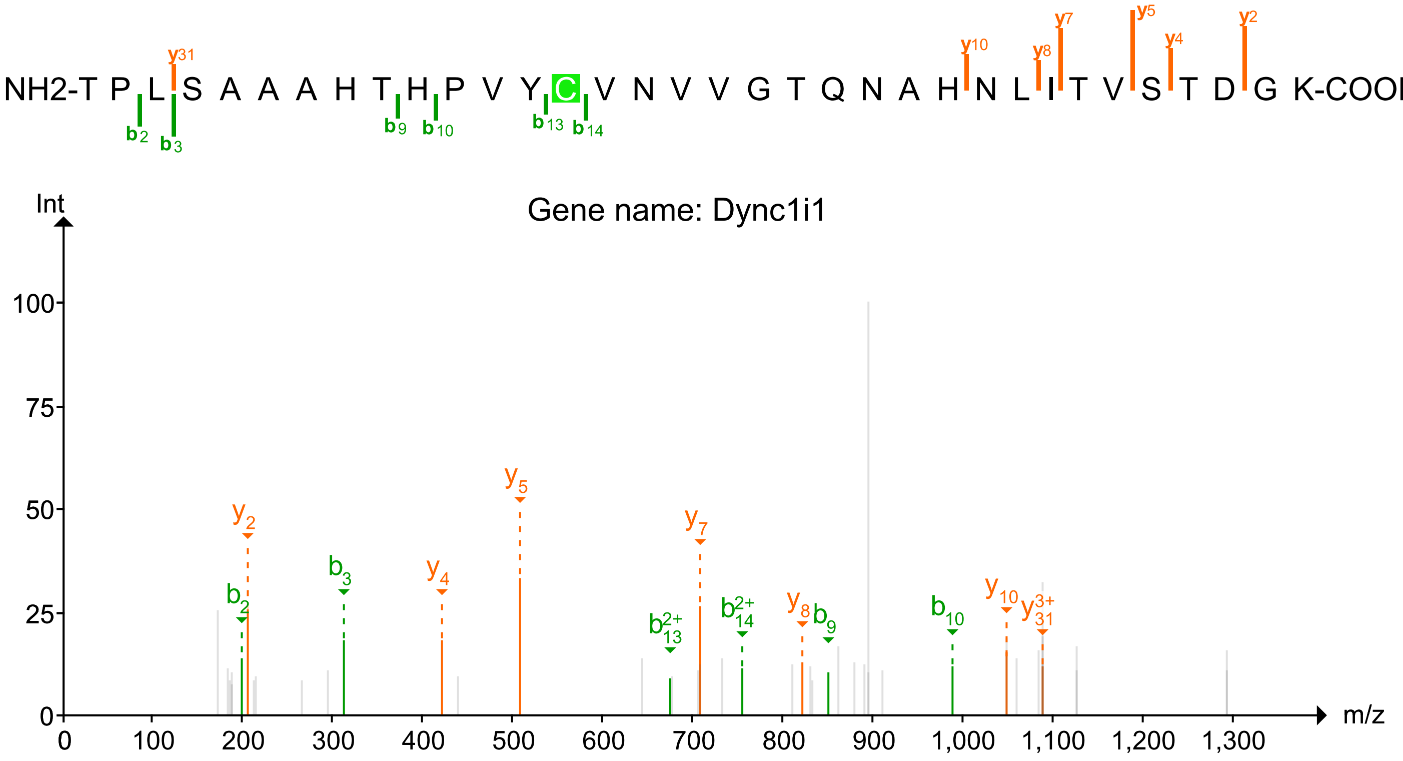
NH2-T P L S A A A H T H P V Y C V N V V G T Q N A H N L I T V S T D G K-COOH
Gene name: Dync1i1
Int
m/z
y2 b2 b3 y4 y5 b2+13 y7 b2+14 y8 b9 b10 y10 y3+31
y31 y10 y8 y7 y5 y4 y2
b2 b3 b9 b10 b13 b14

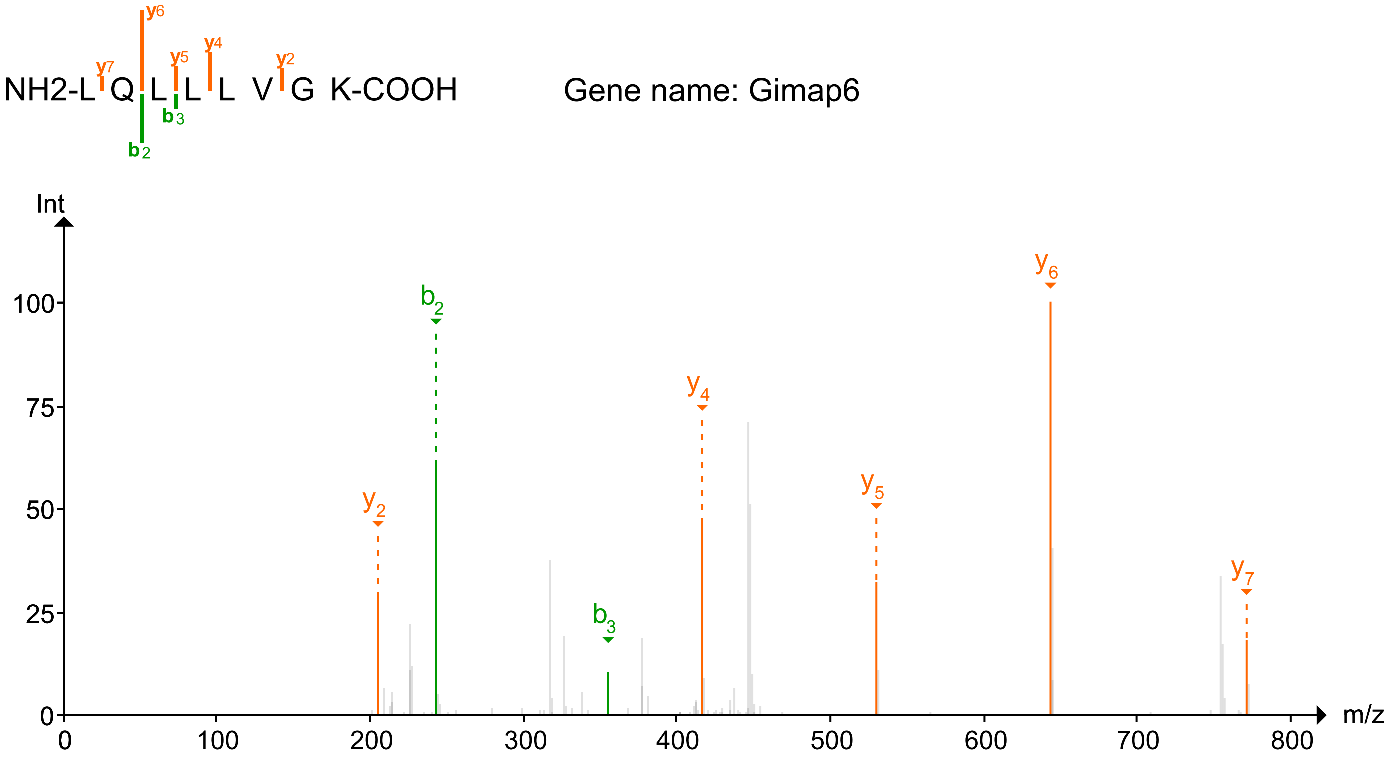

NH2-L Q L L L V G K-COOH
y7
y6
y5
y4
y2
b2
b3
Gene name: Gimap6
Int
m/z
0
25
50
75
100
0
100
200
300
400
500
600
700
800
y2
b2
b3
y4
y5
y6
y7

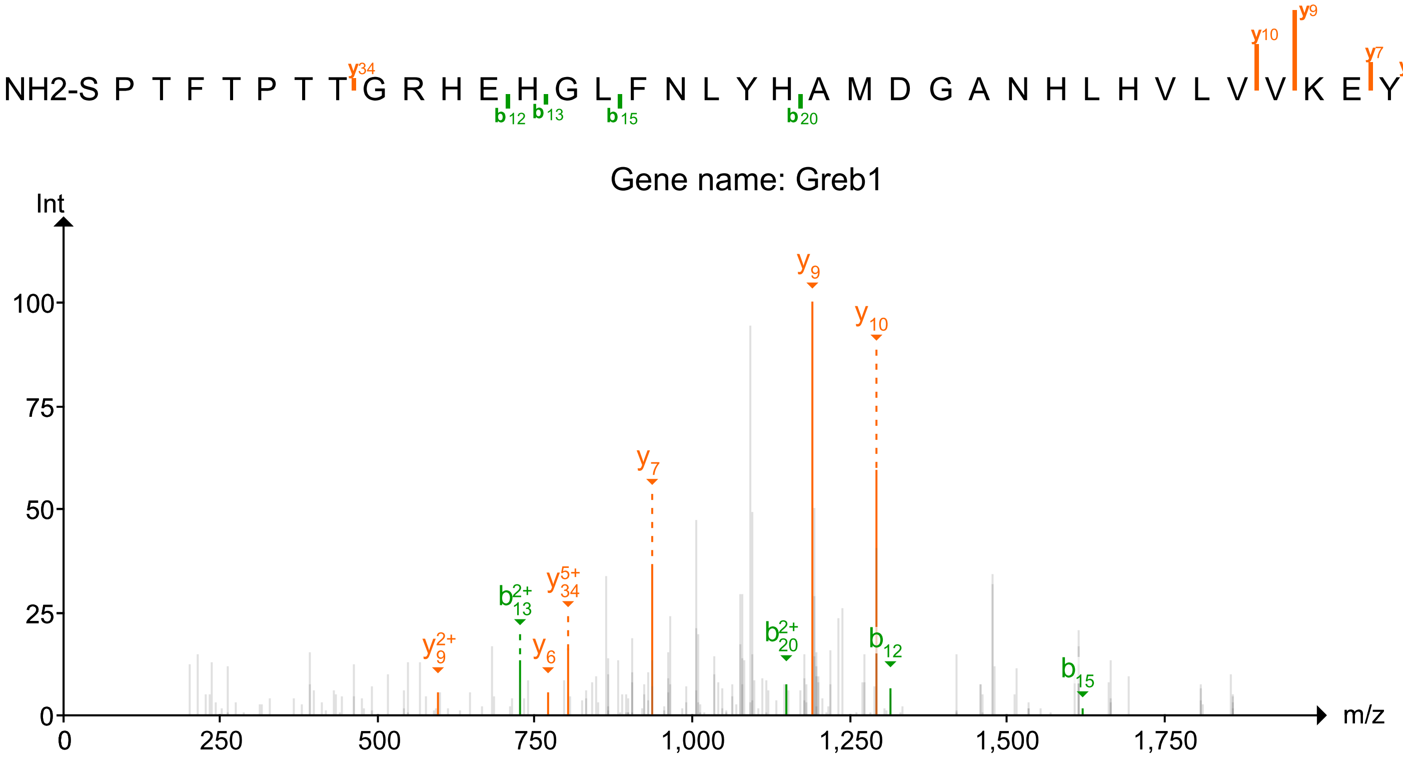

NH2-S P T F T P T T G R H E H G L F N L Y H A M D G A N H L H V L V V K E Y
y34 y10 y9 y7
b12 b13 b15 b20
Gene name: Greb1
Int
m/z
100
75
50
25
0
0
250
500
750
1,000
1,250
1,500
1,750
y9
y10
y7
y9 2+
b13 2+
y6
y34 5+
b20 2+
b12
b15

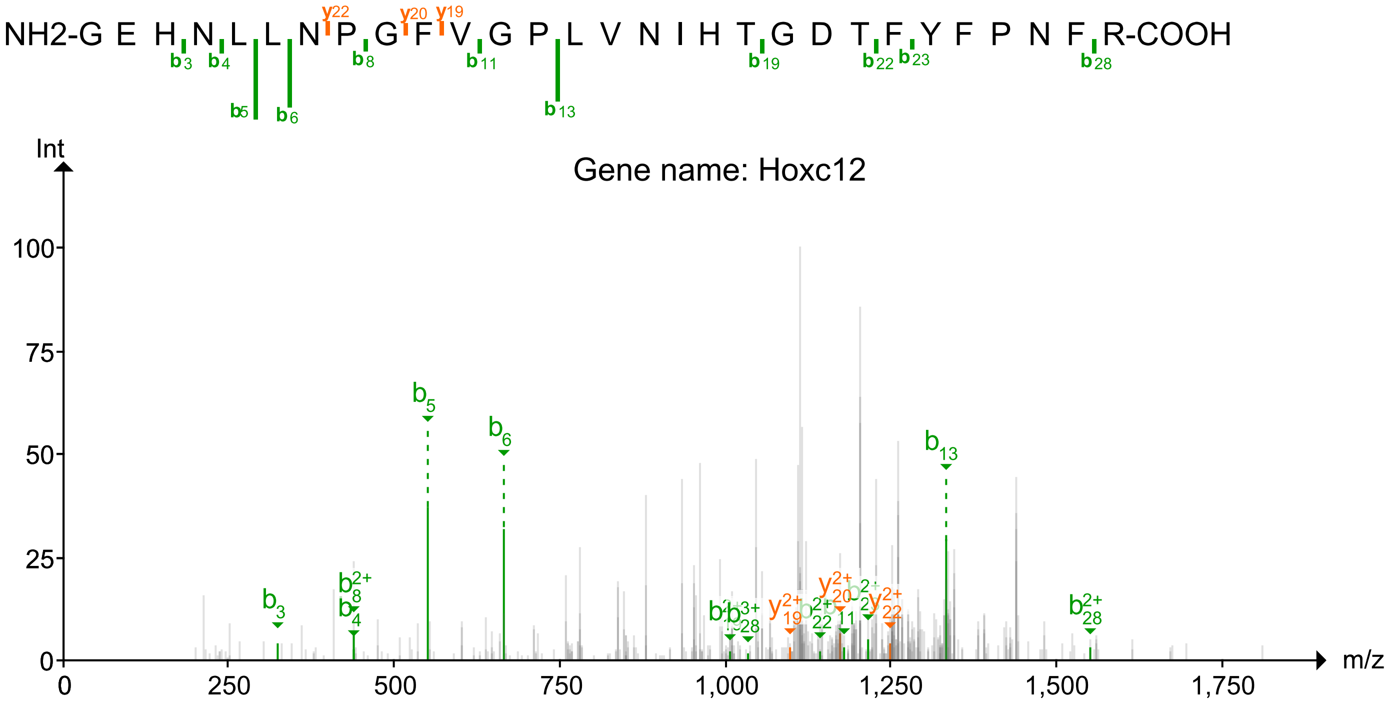
NH2-G E H N L L N P G F V G P L V N I H T G D T F Y F P N F R-COOH
Gene name: Hoxc12
Int
m/z

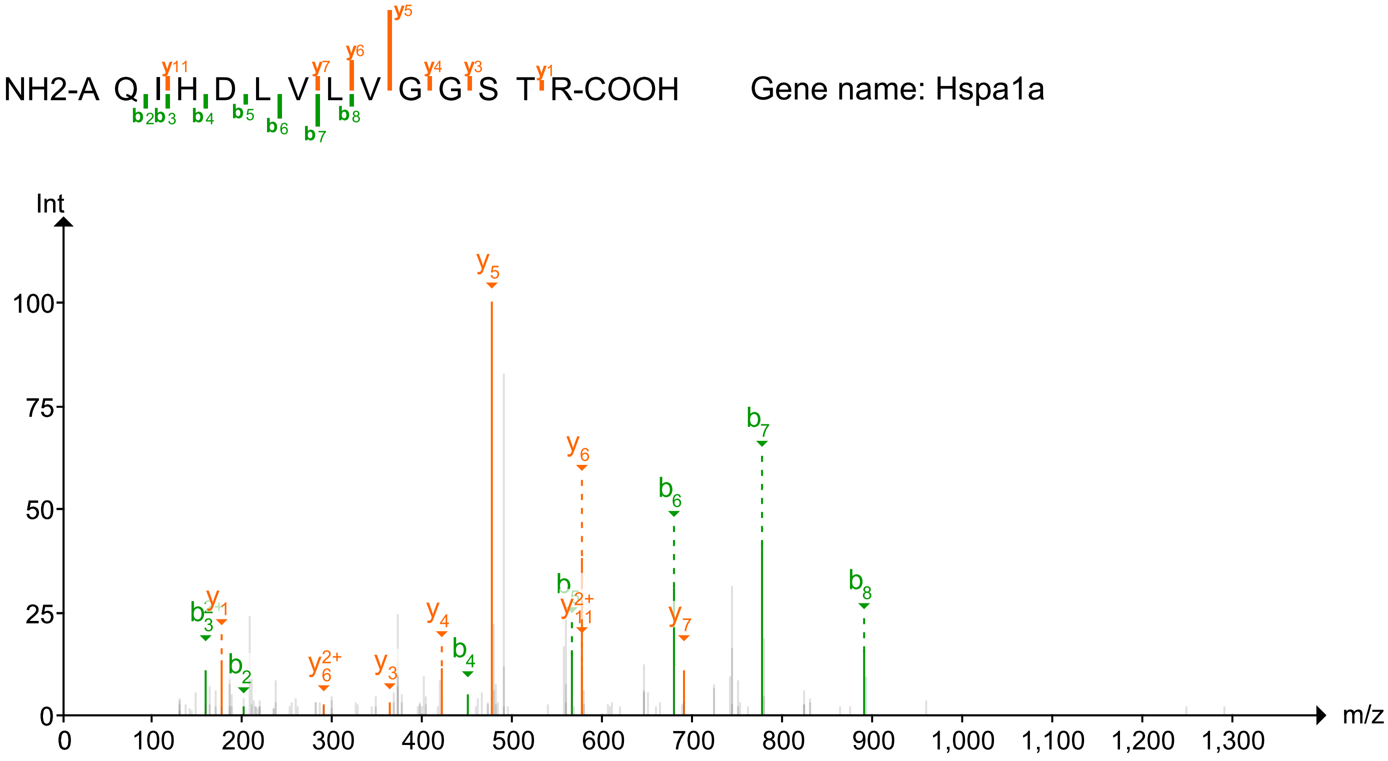

NH2-A Q I H D L V L V G G S T R-COOH
Gene name: Hspa1a
Int
m/z

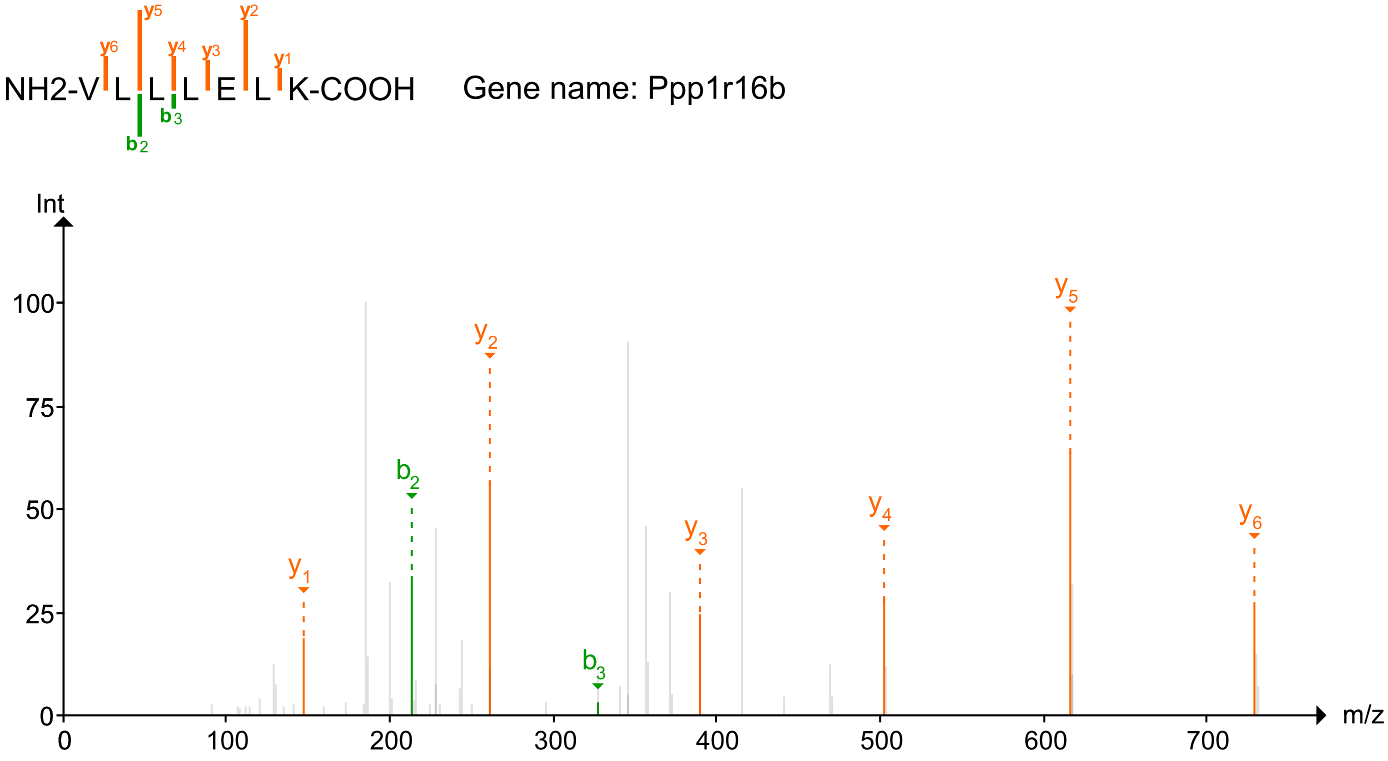
NH2-VLLLELK-COOH
Gene name: Ppp1r16b
y6
y5
y4
y3
y2
y1
b2
b3
Int
100
75
50
25
0
m/z
0
100
200
300
400
500
600
700

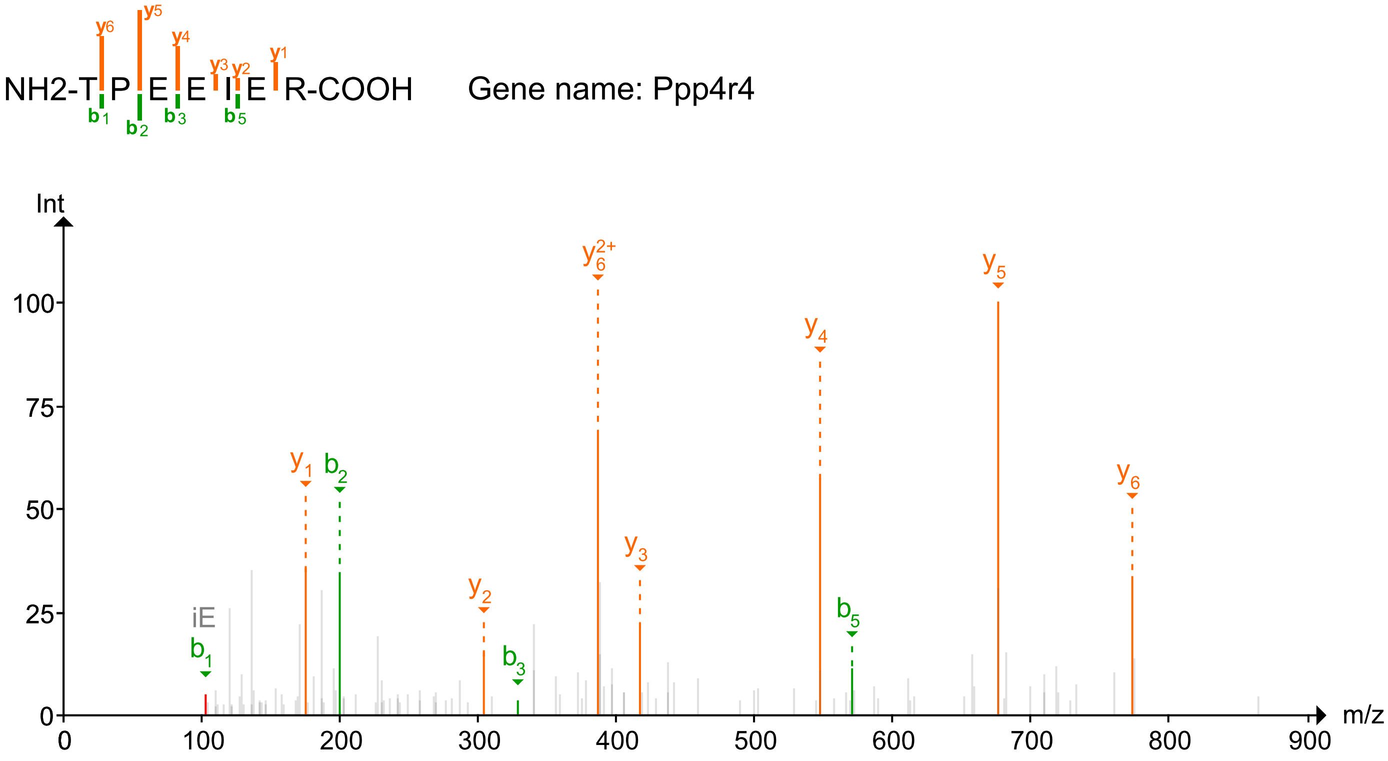
NH2-T P E E I E R-COOH
y6 y5 y4 y3 y2 y1
b1 b2 b3 b5
Gene name: Ppp4r4
Int
m/z
iE
0 25 50 75 100
0 100 200 300 400 500 600 700 800 900

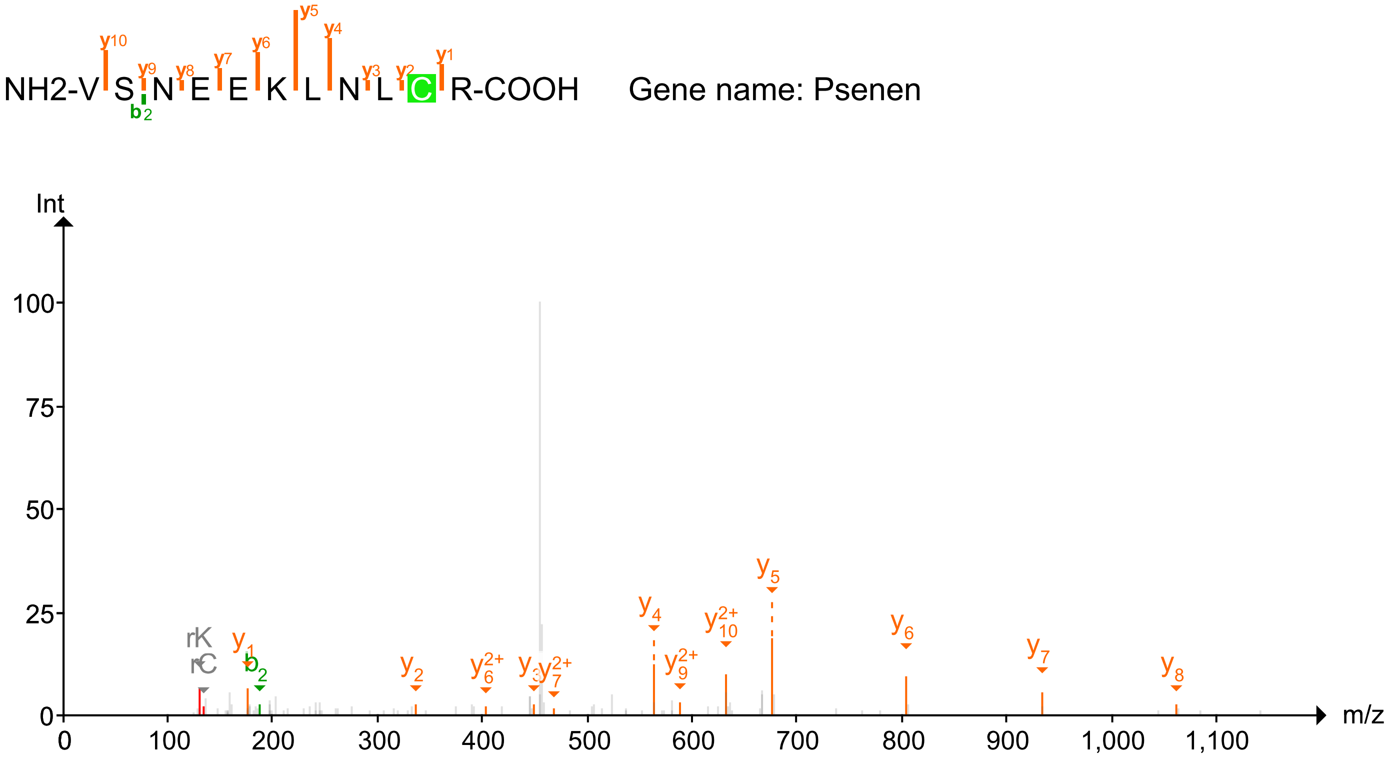
NH2-V S N E E K L N L C R-COOH
Gene name: Psenen
y10 y9 y8 y7 y6 y5 y4 y3 y2 y1
b2
Int
100
75
50
25
0
m/z
0 100 200 300 400 500 600 700 800 900 1,000 1,100
rK
rC
y1
b2
y2
y6 2+
y3
y7 2+
y4
y9 2+
y10 2+
y5
y6
y7
y8

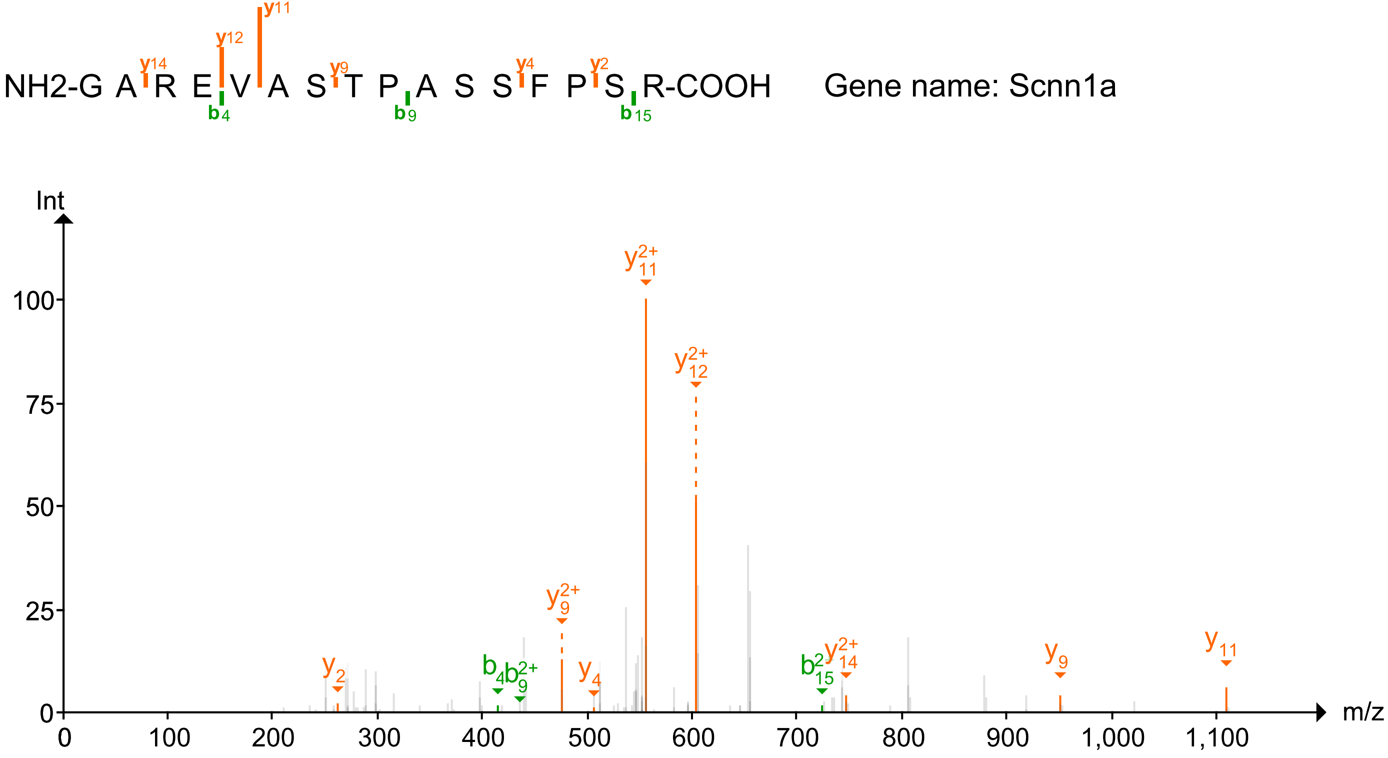
NH2-G A R E V A S T P A S S F P S R-COOH
Gene name: Scnn1a
y14
y12
y11
y9
y4
y2
b4
b9
b15
Int
m/z
0
25
50
75
100
0
100
200
300
400
500
600
700
800
900
1,000
1,100
y2+ 11
y2+ 12
y2+ 9
y2
b4
b2+ 9
y4
b2+ 15
y2+ 14
y9
y11

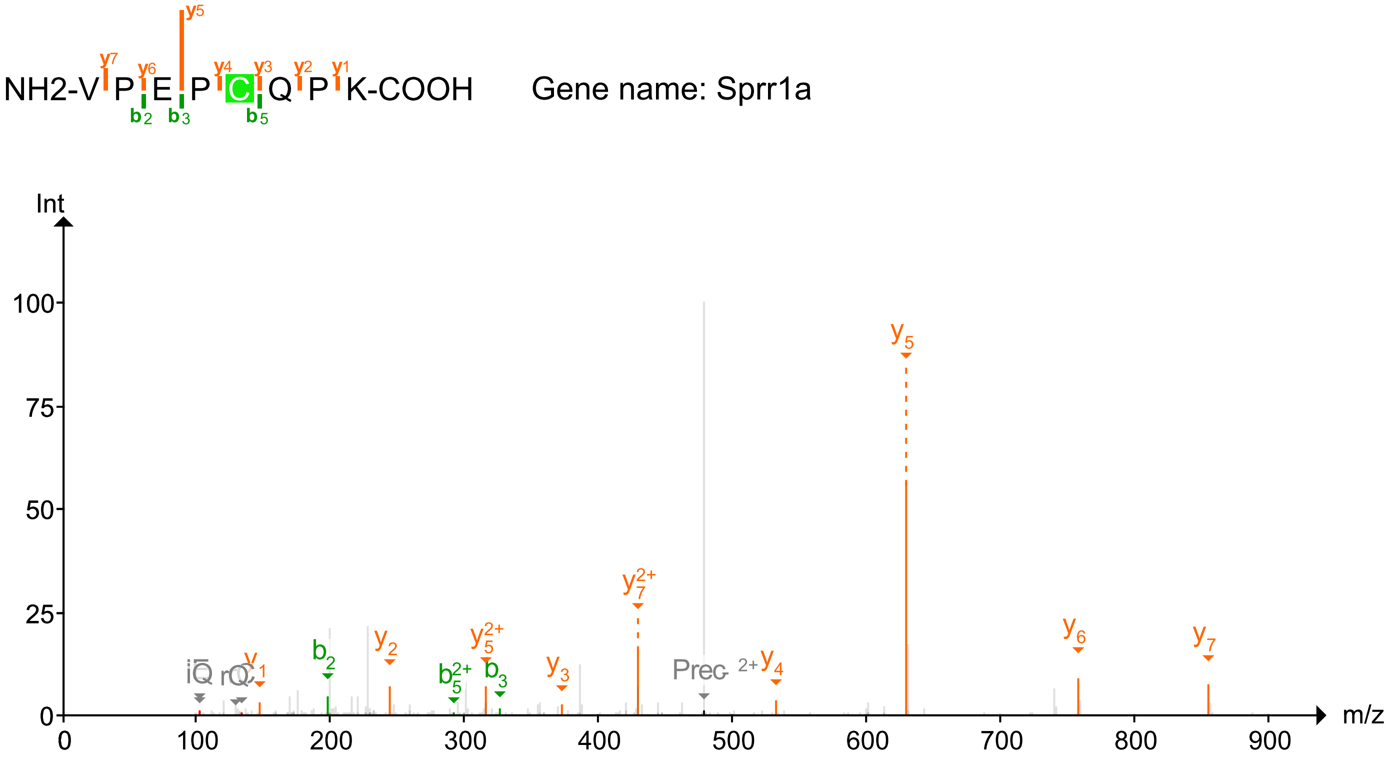

NH2-VPEPCQPK-COOH
Gene name: Sprr1a
y7 y6 y5 y4 y3 y2 y1
b2 b3 b5
Int
100
75
50
25
0
m/z
0 100 200 300 400 500 600 700 800 900
y5
y7 2+
y5 2+
y2
b2
b5 2+
b3
y3
Prec 2+
y4
y6
y7
y1
iQ
rC

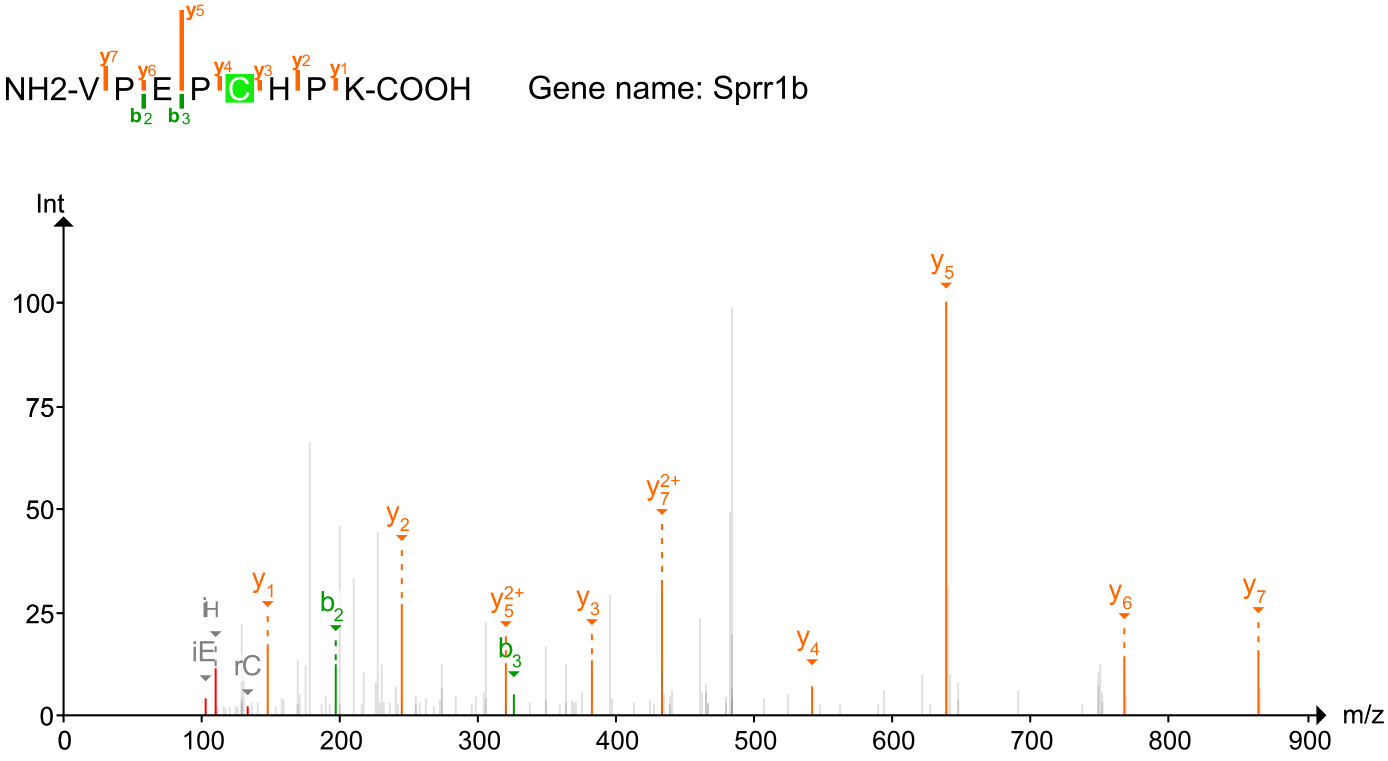
NH2-VPEPCHPK-COOH
Gene name: Sprr1b
Int
m/z
y5
y7
y6
y4
y3
y2
y1
b2
b3
iH
iE
rC
y7 2+
y5 2+
100
75
50
25
0
0
100
200
300
400
500
600
700
800
900

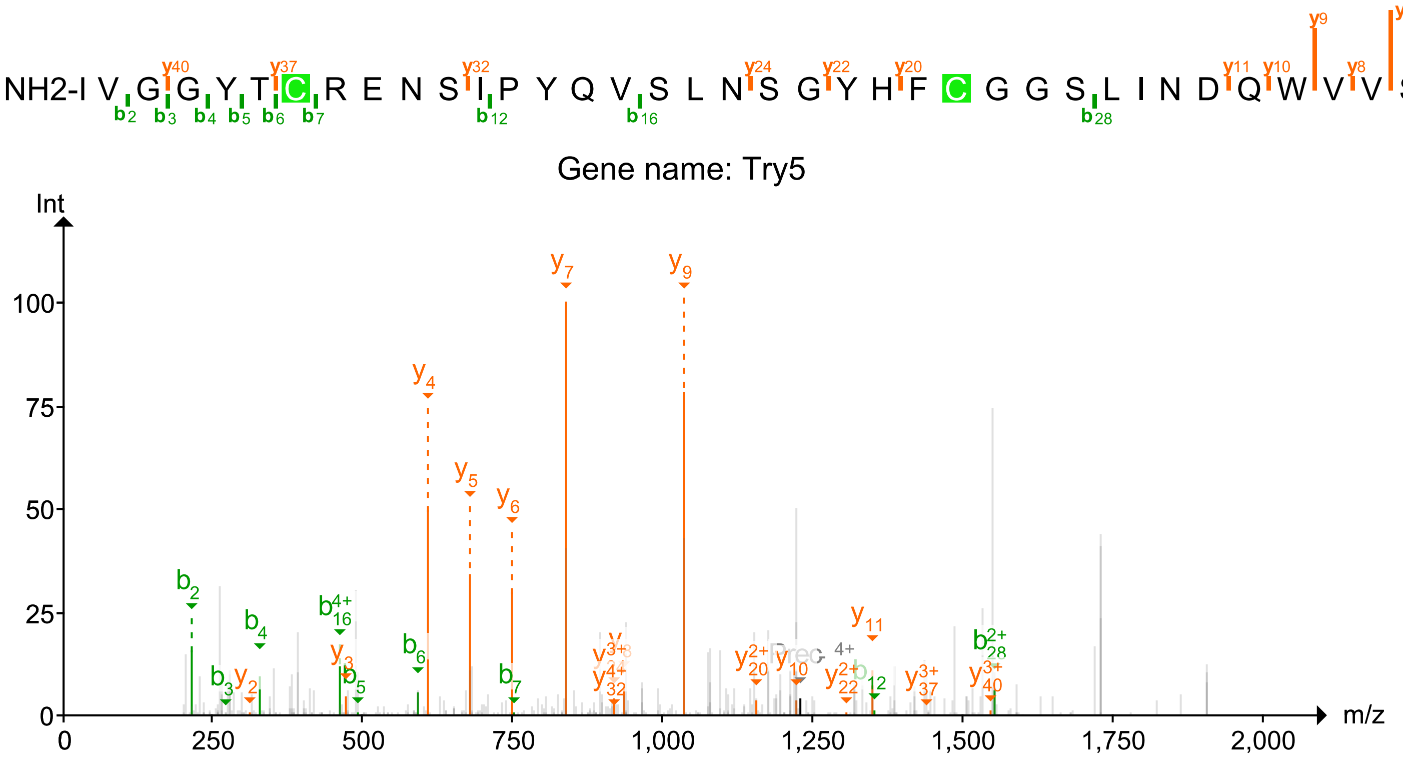

NH2-I V G G Y T C R E N S I P Y Q V S L N S G Y H F C G G S L I N D Q W V V S
Gene name: Try5
Int
m/z

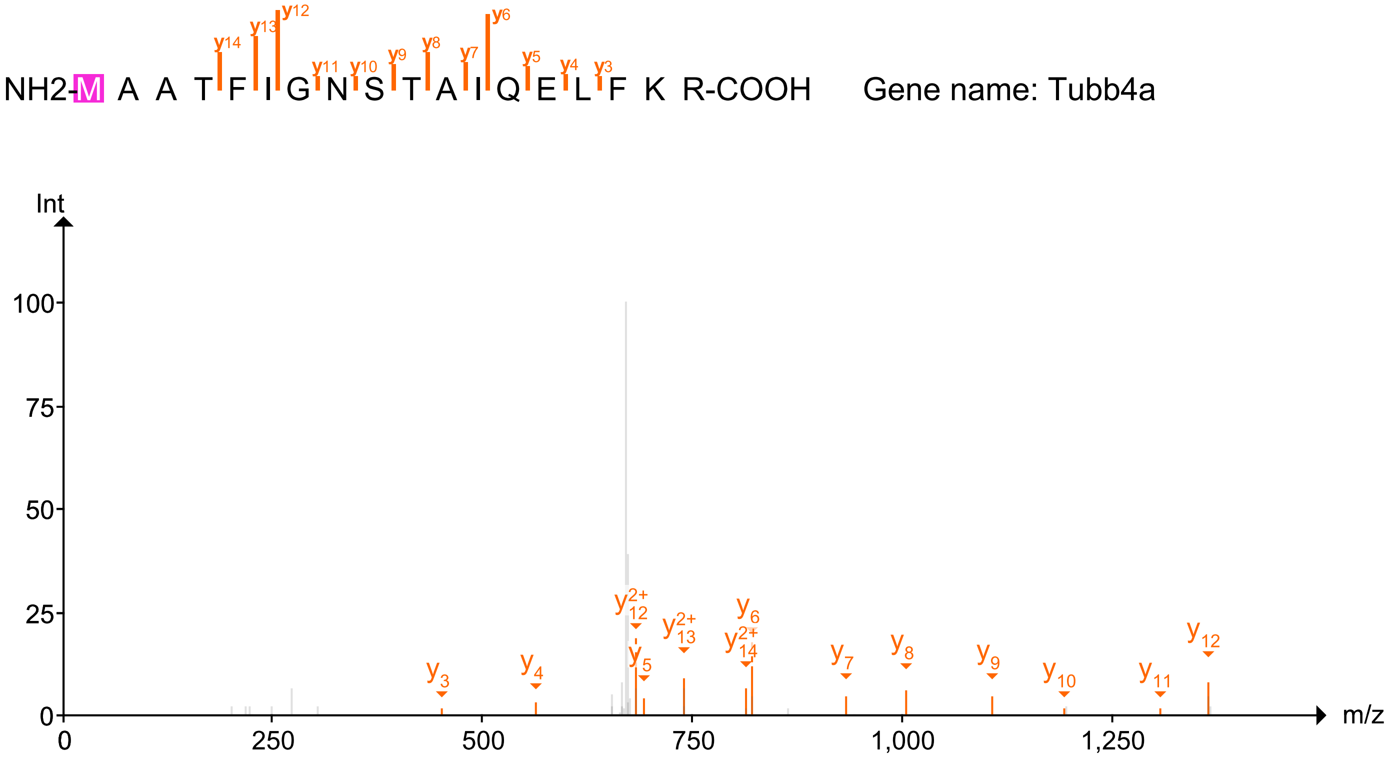

NH2-M A A T F I G N S T A I Q E L F K R-COOH
Gene name: Tubb4a
y14
y13
y12
y11
y10
y9
y8
y7
y6
y5
y4
y3
Int
100
75
50
25
0
m/z
0
250
500
750
1,000
1,250
y3
y4
y12 2+
y5
y13 2+
y14 2+
y6
y7
y8
y9
y10
y11
y12

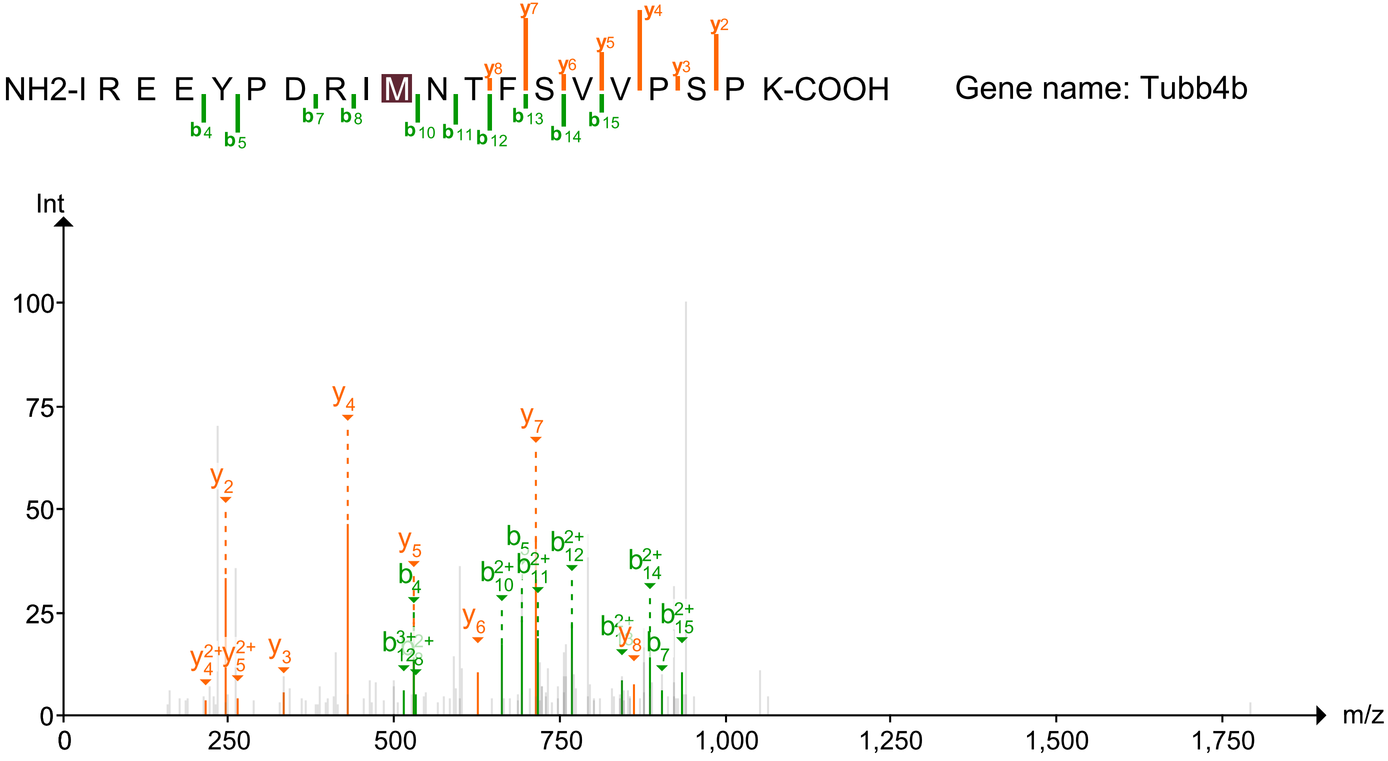

NH2-I R E E Y P D R I M N T F S V V P S P K-COOH
Gene name: Tubb4b
Int
m/z

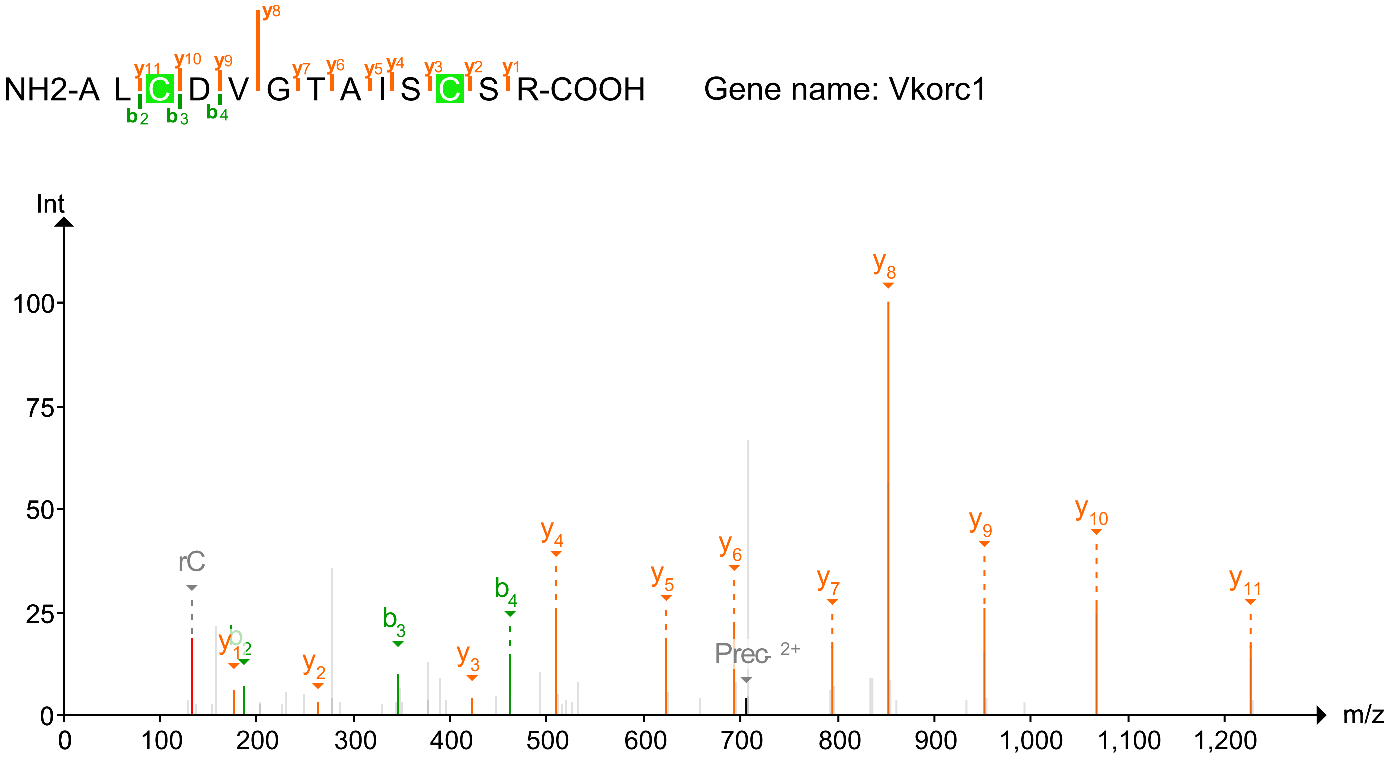

NH2-A L C D V G T A I S C S R-COOH
Gene name: Vkorc1
y11 y10 y9 y8 y7 y6 y5 y4 y3 y2 y1
b2 b3 b4
Int
m/z
0
25
50
75
100
0
100
200
300
400
500
600
700
800
900
1,000
1,100
1,200
rC
y1
b2
y2
b3
y3
b4
y4
y5
y6
Prec 2+
y7
y8
y9
y10
y11
